# Modeling antibiotic and cytotoxic effects of the dimeric isoquinoline IQ-143 on metabolism and its regulation in *Staphylococcus aureus*, *Staphylococcus epidermidis *and human cells

**DOI:** 10.1186/gb-2011-12-3-r24

**Published:** 2011-03-21

**Authors:** Alexander Cecil, Carina Rikanović, Knut Ohlsen, Chunguang Liang, Jörg Bernhardt, Tobias A Oelschlaeger, Tanja Gulder, Gerhard Bringmann, Ulrike Holzgrabe, Matthias Unger, Thomas Dandekar

**Affiliations:** 1 University of Würzburg, Theodor-Boveri Institute, Department of Bioinformatics, Am Hubland, 97074 Würzburg, Germany; 2 University of Würzburg, Institute for Pharmacy and Food Chemistry, Am Hubland, 97074 Würzburg, Germany; 3 University of Würzburg, Institute for Molecular Infection Biology, Josef-Schneider-Straße 2, 97080 Würzburg, Germany; 4 Ernst-Moritz-Arndt University, Institute for Microbiology, Greifswald, Friedrich- Ludwig- Jahn- Straße 15, 17487 Greifswald, Germany; 5 University of Würzburg, Institute for Organic Chemistry, Am Hubland, 97074 Würzburg, Germany; 6 Present address: RWTH Aachen, Institute of Organic Chemistry, Landoltweg 1, 52074 Aachen, Germany; 7 EMBL Heidelberg, BioComputing Unit, Meyerhofstraße 1, 69117 Heidelberg, Germany

## Abstract

**Background:**

Xenobiotics represent an environmental stress and as such are a source for antibiotics, including the isoquinoline (IQ) compound IQ-143. Here, we demonstrate the utility of complementary analysis of both host and pathogen datasets in assessing bacterial adaptation to IQ-143, a synthetic analog of the novel type *N*,*C*-coupled naphthyl-isoquinoline alkaloid ancisheynine.

**Results:**

Metabolite measurements, gene expression data and functional assays were combined with metabolic modeling to assess the effects of IQ-143 on *Staphylococcus aureus*, *Staphylococcus epidermidis *and human cell lines, as a potential paradigm for novel antibiotics. Genome annotation and PCR validation identified novel enzymes in the primary metabolism of staphylococci. Gene expression response analysis and metabolic modeling demonstrated the adaptation of enzymes to IQ-143, including those not affected by significant gene expression changes. At lower concentrations, IQ-143 was bacteriostatic, and at higher concentrations bactericidal, while the analysis suggested that the mode of action was a direct interference in nucleotide and energy metabolism. Experiments in human cell lines supported the conclusions from pathway modeling and found that IQ-143 had low cytotoxicity.

**Conclusions:**

The data suggest that IQ-143 is a promising lead compound for antibiotic therapy against staphylococci. The combination of gene expression and metabolite analyses with *in silico *modeling of metabolite pathways allowed us to study metabolic adaptations in detail and can be used for the evaluation of metabolic effects of other xenobiotics.

## Background

Antibiotic treatment of infectious diseases has become increasingly challenging as pathogenic bacteria have acquired a broad spectrum of resistance mechanisms. In particular, the emergence and spread of multi-resistant staphylococci has progressed to a global health threat [1]. They are not only resistant to almost all treatments, but also adapt very well to different conditions in the host, including persistence [2-4]. In the face of increasing resistance against antibiotics as well as persistence of staphylococci in the patient, an intensive search of new antibacterial lead compounds addressing new targets is urgently required.

Currently, several '-omics' techniques are available, but they are expensive and, in general, only limited information is available for each type of data [5]. We will show how different data sets for studying the metabolic effects of a xenobiotic can be efficiently combined to derive a maximum of information utilizing pathway modeling [6-8] while validating the latter by experimental data.

A new emerging paradigm for investigating drug effects and toxicity is followed here: instead of considering the body of the studied organism as a black box and just identifying toxic or antibiotic concentrations, genomics and post-genomics strategies are used to reveal affected pathways. This combination enables a more rapid understanding of metabolic effects and at the same time also reveals side effects in unprecedented detail, leading to a network paradigm: a substance is not just toxic or nontoxic but has, in general, stronger or weaker and concentration-dependent network effects.

**Figure 1:**
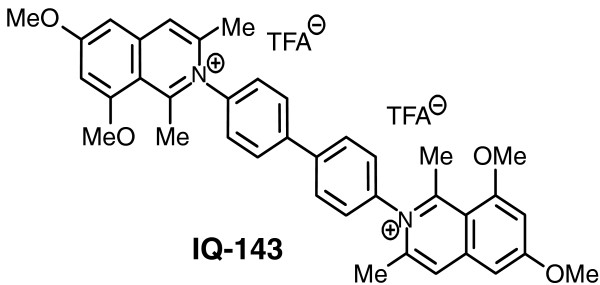
**Structure of IQ-143**. Shown is the structure of the environmental challenge and xenobiotic chosen, isoquinolinium salt IQ-143, a structurally simplified analogue of a new subclass of bioactive natural products, the *N*,*C*-coupled naphthyl-isoquinolines alkaloids.

In our studies we observed a drastic change in metabolic activity after administration of the isoquinolinium salt IQ-143 (Figure 1) and show for staphylococci that this compound is a xenobiotic with antibiotic properties. IQ-143 constitutes a structurally simplified analogue of a new subclass of bioactive natural products, the *N*,*C*-coupled naphthylisoquinoline alkaloids, which were first isolated from tropical lianas belonging to the Ancistrocladaceae plant family. Representatives of these alkaloids, such as ancistrocladinium A and B, exhibit excellent antiinfective activities - for example, against the pathogen *Leishmania major *- and thus serve as promising lead structures for the treatment of severe infectious diseases [9-13]. This class of compounds comprises complex natural products and newly developed synthetic analogues thereof [14-16] and provides a rich repertoire of representatives with a large potential against a number of infectious diseases, but potentially also bears the risk of toxic effects in humans.

Starting from publicly available genome sequences [17,18], genome annotation in the staphylococci strains was completed by sequence and domain analysis [19] to identify several previously unidentified metabolic enzymes of their central metabolism. The respective bioinformatic results obtained were validated by PCR analysis. The obtained gene expression data helped to monitor in detail the effect of different concentrations of the isoquinoline on staphylococci. Also, the combination with metabolic modeling allowed us to fill in missing information on all central metabolic enzymes, including those not affected by significant gene expression changes, and to obtain a complete view of the resulting metabolic adaptations of the staphylococci. These genome-scale predictions were further validated by direct metabolite measurements on specific nucleotides.

In general, the pathway modeling allows one to consider network effects besides target effects (for instance, on glycolysis, which decreases with increasing IQ-143 concentrations but is not a direct target of IQ-143) and to find areas that are comparatively resistant (for example, the pentose phosphate pathway). Gene expression data are complemented by the network modeling and from these counter regulation by higher gene expression can be identified. Only a few metabolite measurements are sufficient to validate the predictions regarding the involved pathways - for example, here regarding nucleotides as well as nucleotide-containing cofactors. We tested the independence of the data sets carefully and used them to also cross-validate the modeled pathway fluxes - for example, whether the network predictions from gene expression data fit measured nucleotide concentrations.

Metabolic responses in human cells were modeled considering measurements on cytochrome P450 (CYP) detoxification data. We extrapolated again for all effects on central pathways and compared the resulting predictions to cytotoxicity data on human cells.

## Results

### IQ-143 added to a *Staphylococcus epidermidis *culture: gene expression changes and metabolic model

IQ-143 has been identified by structure-activity relationship studies in a screening program for compounds with anti-staphylococcal activity [20]. To get a first hint of the mode of action of this substance, DNA-microarray experiments were conducted. The clinical *S. epidermidis *strain RP62A was grown in the presence of IQ-143 (concentrations of a quarter of the minimum inhibitory concentration and twice the minimum inhibitory concentration) as described in the Materials and methods section and hybridized to full genome arrays. Significant gene expression differences for *S. epidermidis *are shown in Tables 1 and 2 (details shown in Additional file 1: Table S5 lists gene expression differences for 1.25 μM of IQ-143, Table S6 for 0.16 μM of IQ-143). Overall, the expression of genes encoding proteins involved in the transport of macromolecules, such as the ATP-binding cassette (ABC) transporter, the peptide transporter, and the choline transporter, and metabolic enzymes of carbohydrate pathways were especially significantly affected.

**Table 1:** Gene expression changes measured after administration of IQ-143 in *S. epidermidis *RP62A

	Gene expression after IQ-143 administration		
	
Affected enzymes	**0.00 μM**^**a**^	0.16 μM	1.25 μM
OP_complex1	1.000	1.000	1.000
OP_complex2	1.000	1.000	1.000
OP_complex3	1.000	1.000	8.390
OP_complex4	1.000	1.000	1.000
OP_complex5a	1.000	1.000	1.000
SERP0290-zinc-transport_efflux	1.000	0.399	0.449
SERP0291-zinc-transporter_import	1.000	0.544	0.450
SERP0292-iron-dicitrate-transporter_import	1.000	0.544	0.430
SERP0389-EC:1.1.1.1-rn:R00754	1.000	1.000	3.070
SERP0653-EC:6.3.5.3-rn:R04463	1.000	1.000	0.491
SERP0655-EC:2.4.2.14-rn:R01072	1.000	1.000	0.436
SERP0656-EC:6.3.3.1-rn:R04208	1.000	1.000	0.424
SERP0657-EC:2.1.2.2-rn:R04325	1.000	1.000	0.426
SERP0658-EC:2.1.2.3-rn:R04560	1.000	1.000	0.439
SERP0659-EC:6.3.4.13-rn:R04144	1.000	1.000	0.392
SERP0686-spermidine/putrescine-transport_import	1.000	1.000	2.361
SERP0687-spermidine/putrescine-transport_import	1.000	1.000	2.208
SERP0688-spermidine/putrescine-transport_import	1.000	1.000	2.075
SERP0765-Uracil-permease-transport_import	1.000	1.000	2.765
SERP0831-EC:2.7.7.7-rn:R00375	1.000	1.000	2.202
SERP0831-EC:2.7.7.7-rn:R00376	1.000	1.000	2.202
SERP0831-EC:2.7.7.7-rn:R00378	1.000	1.000	2.202
SERP0831-EC:2.7.7.7-rn:R00379	1.000	1.000	2.202
SERP0841-EC:2.7.7.8-rn:R00437	1.000	1.000	2.867
SERP0841-EC:2.7.7.8-rn:R00439	1.000	1.000	2.867
SERP1403-MultiDrug-transport_efflux	1.000	1.000	2.063
SERP1802-cobalt/nickel-transport_efflux	1.000	1.000	2.401
SERP1803-cobalt/nickel-transport_efflux	1.000	1.000	2.301
SERP1944-MultiDrug-transport_efflux	1.000	1.000	2.075
SERP1951-lipoprotein-transport_efflux/import	1.000	1.000	0.457
SERP1952-macrolide-transport_efflux	1.000	1.000	0.386
SERP1997-formate/nitrite-transport_efflux/import	1.000	1.000	2.619
SERP2060-glyerol-transport_import	1.000	1.000	2.823
SERP2156-EC:1.1.1.27-rn:R00703	1.000	1.000	0.486
SERP2179-choline/betaine/carnitine-transp_efflux	1.000	7.071	2.389
SERP2186-EC:2.7.7.4-rn:R00529	1.000	1.000	0.349
SERP2283-phopsphonate-transport_import	1.000	1.000	2.680
SERP2289-MultiDrug-transport_efflux	1.000	1.000	1.971

This table shows the gene expression changes measured after administration of IQ-143. 1.0 denotes the standard activity without IQ-143. A value of 0.5 indicates that the activity of this enzyme was halved after administration of IQ-143, a value of 2.075 indicates that the activity was doubled (again after administration of IQ-143). ^a^Expression with no IQ-143 (0.00 μM column) is set to 1.000 for normalization purposes.

**Table 2:** Key effects of the measured gene expression differences after administration of IQ-143 compared to untreated *S. epidermidis *RP62A

Concentration of IQ-143 (μM)	**Enzymes affected**^**a**^	**Effect on enzymes**^**b**^	**Phenotypic effects**^**c**^
0.16 μM	SERP0290-zinc-transport_efflux	Down-regulated	
	SERP0291-zinc-transporter_import	Down-regulated	40% biofilm inhibition
	SERP0292-iron-dicitrate-transporter_import	Down-regulated	No growth inhibition
	SERP2179-choline/betaine/carnitine-transp_efflux	Up-regulated	
			
1.25 μM	SERP0290-zinc-transport_efflux	Down-regulated	
	SERP0291-zinc-transporter_import	Down-regulated	
	SERP0292-iron-dicitrate-transporter_import	Down-regulated	
	SERP0653-FGAM synthetase-rn:R04463	Down regulated	
	SERP0655-amidophosphoribosyltransferase-rn:R01072	Down-regulated	
	SERP0656-AIR synthetase-rn:R04208	Down-regulated	
	SERP0657-GAR formyltransferase-rn:R04325	Down-regulated	
	SERP0658-AICAR transformylase-rn:R04560	Down-regulated	~100% biofilm inhibition
	SERP0659-glycinamide ribonucleotide synthetase-rn:R04144	Down-regulated	~100% growth inhibition
	SERP0686-spermidine/putrescine-transport_import	Up-regulated	
	SERP0687-spermidine/putrescine-transport_import	Up-regulated	
	SERP0688-spermidine/putrescine-transport_import	Up-regulated	
	SERP0765-Uracil-permease-transport_import	Up-regulated	
	SERP0831-DNA polymerase-rn:R00375	Up-regulated	
	SERP0831-DNA polymerase-rn:R00376	Up-regulated	
	SERP0831-DNA polymerase-rn:R00378	Up-regulated	
	SERP0831-DNA polymerase-rn:R00379	Up-regulated	
	SERP0841-PNPase-rn:R00437	Up-regulated	
	SERP0841-PNPase-rn:R00439	Up-regulated	
	SERP1403-MultiDrug-transport_efflux	Up-regulated	
	SERP1802-cobalt/nickel-transport_efflux	Up-regulated	
	SERP1803-cobalt/nickel-transport_efflux	Up-regulated	
	SERP1944-MultiDrug-transport_efflux	Up-regulated	
	SERP1951-lipoprotein-transport_efflux/import	Down-regulated	
	SERP1952-macrolide-transport_efflux	Down-regulated	
	SERP1997-formate/nitrite-transport_efflux/import	Up-regulated	
	SERP2060-glyerol-transport_import	Up-regulated	
	SERP2179-choline/betaine/carnitine-transp_efflux	Up-regulated	
	SERP2186-ATP-sulfurylase;-rn:R00529	Down-regulated	
	SERP2283-phosphonate-transport_import	Up-regulated	
	SERP2289-MultiDrug-transport_efflux	Up-regulated	

^a^Locus tags are given first (SERP numbers), followed by abbreviated biochemical name and then KEGG reaction numbers (always starting with - m:R...). The effects on *S. aureus *USA300 were modeled (Table S20 in Additional file 1), are similar overall, and were validated by metabolite measurements. ^b^Down-regulated means that gene expression was halved (or more then halved); up-regulated means that gene expression was doubled (or more than doubled). Specific values are given in Tables S5 and S6 in Additional file 1. All the enzymes with key changes in expression are part of the complete simulated metabolic model. ^c^The phenotypes are combination effects of the complete networks, not of single modes (see also Figure S2 in Additional file 1).

**Figure 2:**
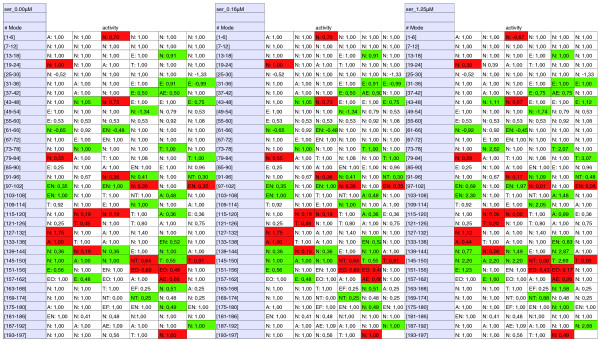
**Changes in extreme modes in *S. epidermidis *RP62A with three different concentrations of IQ-143**. Red shading indicates lower activities after IQ-143 administration, green shading indicates higher activities, and 'ser' denotes *S. epidermidis*. Each row displays the changes for six extreme modes (continuously numbered from 1 to 197); numbers given in the fields are the activities for each mode under different concentrations of IQ-143. Also given are the pathways in which the modes are involved. Abbreviations: A, amino acids; E, energy metabolism; F, fatty acids; N, nucleotide metabolism; O, oxidative phosphorylation; T, transporters. All details are also shown in Additional file 1 (Tables S10, S11, and S12; key changes in Tables S16 and S17).

**Figure 3:**
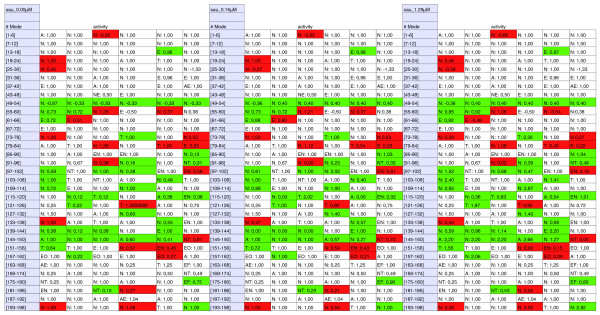
**Changes in extreme modes in *S. aureus *USA300 with three different concentrations of IQ-143**. Red shading indicates lower activities after IQ-143 administration, green shading indicates higher activities, and 'sau' denotes *S. aureus*. Each row displays six extreme modes (continuously numbered from 1 to 198); numbers given in the fields are the activities for each mode under different concentrations of IQ-143. Also given are the pathways in which the modes are involved. Abbreviations: A, amino acids; E, energy metabolism; F, fatty acids; N, nucleotide metabolism; O, oxidative phosphorylation; T, transporters. All details are also shown in Additional file 1 (Tables S7, S8, and S9; key changes in Tables S18 and S19).

To analyze pathway changes resulting from the mode of action of IQ-143, including identification of affected enzymes that are not already apparent from the transcriptome data, we applied YANAsquare [21,22] and a custom-made routine written in R [23] for calculating metabolic-flux changes after administration of IQ-143 (Figures 2 and 3).

The calculation of the pathway changes started from the metabolic model of *S. epidermidis *(details in Table S3 in Additional file 1) and applied the gene expression data with significant expression changes (Table 1) as flux constraints (Tables S10, S11 and S12 in Additional file 1; detailed changes in Tables S16 and S17 in Additional file 1).

We first prepared a stoichiometric matrix in which the rows and columns correspond to all the enzymes (for annotation and collection see next chapter in results and Materials and methods) in the network as well as the internal metabolites of the network. The 'internal' metabolites inside the network have to be balanced: tshould neither accumulate nor be lost over time. This condition permits calculation of all enzyme combinations that balance their metabolites inside the network. This yields a list of all metabolic pathways possible for this network [24]. In real situations, such as growth with or without IQ-143, these possible pathways are used quite differently. Next, we calculated the actual flux distribution with a specific program; to do this, direct experimental data are required. The significantly differentially expressed enzymes provide such data and constraints on the flux distribution. This is, of course, a simplification as enzyme activity is modulated allosterically and further factors are involved, such as stability of mRNA and translational regulation. However, the combined errors are strongly reduced by the high number of constraints introduced by the gene expression data. For the complete system of enzymes with significant gene expression changes, the squared deviation between the predicted enzyme activity according to the estimated flux distribution and the observed enzyme activity was minimized (least-square minimization combining the genetic algorithm of YANAsquare with a custom written R routine; see Materials and methods).

From the complete set of flux calculations, several enzyme changes that were not detected by the transcriptome data became apparent (Table 1). Certainly, these are only predictions taking the network effects into account. However, they were subsequently re-checked using metabolite measurements (see below). Numerous repetitions of the transcriptome measurements may also have detected them, as more subtle differences then become significant. On the other hand, the amount of enzyme and activity is likely to be different from subtle transcriptional changes. As an example, combined effects on nucleotide and energy metabolism are described in several extreme pathway modes (Table 1; see, for example, modes 127 and 161 in Tables S7, S8, S9, S10, S11, and S12 in Additional file 1). These flux changes pertain to the enzymes (with EC numbers in parentheses) PNPase (2.4.2.1), glucokinase (2.7.1.2), deoxycytidine kinase (2.7.1.74), DNA-directed RNA polymerase (2.7.7.6), deoxycytidine deaminase (3.5.4.14), alpha-D-Glucose-1-epimerase (5.1.3.3), and glucose-6-phosphate isomerase (5.3.1.9). Furthermore, changes in amino acid metabolism became apparent from the flux changes for modes 35 and 154. Enzymes involved in energy and amino acid metabolism change their activity after administration of IQ-143. This included citric synthase (2.3.3.1), aconitate hydratase (4.2.1.3) and acetyl-CoA synthetase (6.2.1.1) as well as enzymes involved in the conversion of acetyl-CoA to L-valine and the conversion of serine to cysteine.

### Annotation of metabolic enzymes and flux balance metabolic model for *S. epidermidis *and *Staphylococcus aureus*

**Figure 4:**
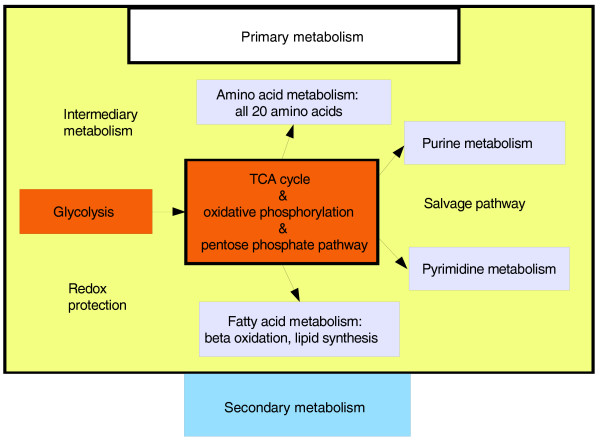
**Simplified view of the metabolic chart for *S. aureus *and *S. epidermidis*, focusing on central metabolic pathways of interest**. This flow chart illustrates which pathways of the primary metabolism are incorporated into our models. Note that the secondary metabolism is not a part of our model. TCA, tricarboxylic acid.

To establish an accurate model of the enzymes involved in the response of staphylococci to IQ-143, we started from the available genome sequences for *S. epidermidis *[Genbank:CP000029, Genbank:CP000028] [17] and *S. aureus *USA300 [Genbank:CP000730 and Genbank:CP000255] [18] and applied biochemical data on staphylococci according to the KEGG database [25]. We considered all pathways of primary metabolism: amino acid, carbohydrate, lipid, and nucleotide synthesis and degradation, salvage pathways and energy metabolism (Figure 4). We established models for both *S. aureus *and *S. epidermidis*; *S. aureus *is well known as a dangerous pathogen, but infections by *S. epidermidis *(normally a commensal of the skin) are increasingly common due to the biofilm-forming capacity of this pathogen and its development of resistance to a broad spectrum of antibacterial agents [26].

We performed sequence and domain analyses [19] to identify several enzymes that had escaped previous annotation efforts, such as nucleoside-triphosphate diphosphatase and thymidine phosphorylase in both strains (Table S1 in Additional file 1), and verified their occurrence in the cDNA of total RNA from *S. epidermidis *by PCR (Figure 6S in Additional file 1). The genome sequences were meticulously analyzed by sequence analysis. In addition, we searched in available data banks for enzyme repertoires of both organisms, and different enzyme reading frames were validated by PCR on the mRNAs from these organisms. Any verified discrepancies by these different checks were next incorporated into the generated metabolic models so that pathways with different enzyme repertoires are different in the two models. For instance, *S. aureus *USA300 has only one AMP-pyrophosphorylase and one GMP-pyrophosphorylase, whereas *S. epidermidis *RP62A has two of each. On the other hand *S. aureus *USA300 has a XMP-ligase, whereas *S. epidermidis *RP62A does not.

Our complete models (reactions in Tables S2 and S3 in Additional file 1) of metabolism in staphylococci systematically included all pathways for which gene expression data pointed to major changes (Tables 1 and 2) in individual enzyme expression after applying different concentrations of IQ-143. Furthermore, the metabolic capabilities of these models were calculated applying YANA [21].

### Changes in reactions and enzyme activity of *S. aureus *and *S. epidermidis *after administration of IQ-143

**Figure 5:**
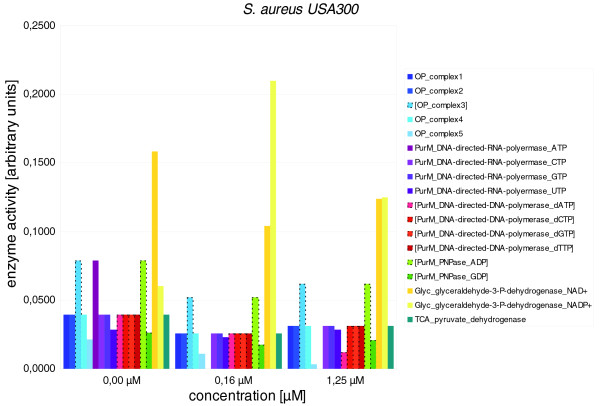
**Effects of IQ-143 on metabolic enzymes of *S. aureus***. Detailed data are given in Table 4. The insert shows the different enzyme color codes. Many differences are apparent after applying metabolic modeling; bars with dotted outlines and brackets around the enzyme name highlight those enzymes in which the different gene expression values already indicate a significant change after administration of IQ-143.

**Figure 6:**
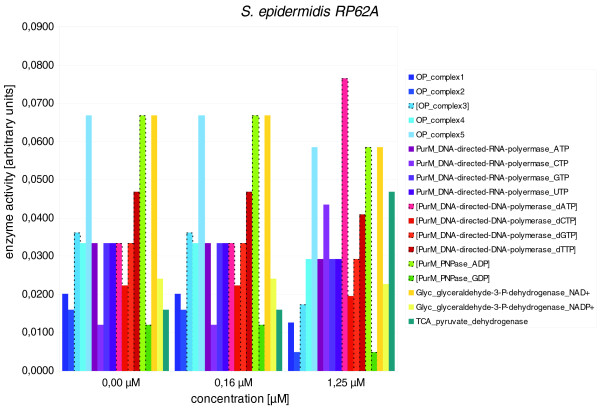
**Effects of IQ-143 on metabolic enzymes of *S. epidermidis***. Detailed data are given in Table 4. The insert shows the different enzyme color codes. Many differences are apparent after applying metabolic modeling; bars with dotted outlines and brackets around the enzyme name highlight those enzymes in which the different gene expression values already indicate a significant change after administration of IQ-143.

Using the above experimental data and the two strain-specific metabolic models, we compared standard growth to the reduced growth after administration of IQ-143 (see Materials and methods). Several species-specific differences with regards to reactions were observed after administration of IQ-143 in *S. aureus *compared to *S. epidermidis*. These are summarized in Figures 2 and 3 (details in Tables S7, S8, S9, S10, S11 and S12). Thus, some modes are only up-regulated (for example, modes 49 and 54 for pyrimidine metabolism in *S. aureus*, but not in *S. epidermidis*) or only down-regulated (for example, modes 44 and 193 for pyrimidine metabolism in *S. epidermidis*, but not changed in *S. aureus*). Some metabolic modes are oppositely regulated in the two strains. For example, mode 122 (involving several transporter proteins for choline, carnithin and betaine) is up-regulated in *S. aureus *but down-regulated in *S. epidermidis*. Nevertheless, most of the calculated metabolic fluxes were similar to those obtained for *S. epidermidis *applying the gene expression data as constraints (Tables S18 and S19 in Additional file 1 detail further changes). Several enzyme changes in *S. epidermidis *and *S. aureus *that were not observable from the transcriptome data became apparent only after applying the metabolic modeling (Figures 5 and 6; bars with dotted outlines indicate changes already indicated by the gene expression data). For example, DNA-directed RNA-polymerases do not change significantly in their respective gene expression, but have clearly different activities under the influence of different concentrations of IQ-143.

The combination of all data with the strain-specific metabolic models showed an effect of IQ-143 on energy metabolism, DNA and RNA elongation as well as bacterial growth for both species (Figure S2 in Additional file 1).

The activity increase in extreme pathway mode 61 (Table S18 in Additional file 1) for the enzymes glucose-6-phosphate isomerase (5.3.1.9), alpha/beta D-glucokinase (2.7.1.1), adenylate kinase (2.7.4.10), and D-glucose-1-epimerase (5.1.3.3) is only visible in *S. aureus*.

### Pathway effects of different concentrations of IQ-143 in *S. epidermidis *and *S. aureus*

Metabolic modeling took advantage of enzyme gene expression changes from the array data by using these data as constraints for the metabolic flux calculations. This allowed us to estimate the effects of different degrees of environmental change after the administration of different concentrations of IQ-143 on not only the metabolism of individual enzymes but also on entire pathways. Using the gene expression changes as constraints in a metabolite flux model to estimate the changes in individual metabolic fluxes after administration of IQ-143, YANAsquare allowed us to calculate the resulting change for each flux and all enzymes in the network [22]. The constraints on the gene expression of several enzymes are of course only a simple first-order estimate of enzyme activity. However, it turned out that the given number (31) of constraints in the model, which were estimated according to significant gene expression changes as well as the tight connections between different pathways in the metabolic network, are sufficient for optimized flux estimates. In particular, the estimated fluxes are in accordance with the measured experimental metabolite concentrations and their changes (see below).

One could expect a general stress response from the administered IQ-143. In fact, we identified stress response mechanisms of *S. epidermidis *RP62A against IQ-143 (Table 3). However, we found significant up-regulation of stress response genes only for two genes after looking at all genes that were up-regulated: SERP2244 and SERP1998. SERP2244 encodes a bacterial capsule synthesis protein (PGA_cap), which may help the bacteria to resist high salt concentrations and may also be involved in virulence [27,28]. SERP1998 is a putative activator of the Hsp90 ATPase homolog 1-like protein. Up-regulation of Hsp90 results in higher survival under conditions of increased stress [29,30]. However, genes belonging to the sigmaB-dependent stress regulon are not affected by IQ-143. Furthermore, the transcriptome data show that several ABC transporters are up-regulated by IQ-143. ABC transporters are often involved in multi-drug resistance as they function as trans-membrane efflux pumps for active transport of several xenobiotics, including anti-infective substances [31]. In staphylococci, several ABC transporters, such as MsrA (conferring resistance to macrolides, lincosamides, streptogramins), TetK (conferring resistance to tetracycline), NorA (conferring resistance to fluoroquinolones), VgaAB (conferring resistance to streptogramins), and FusB (conferring resistance to fusidic acid), have been shown to be involved in antibiotic resistance [32]. However, the ABC transporters deregulated by IQ-143 in this study have not been documented to be involved in resistance to xenobiotics yet. Further studies are needed to clarify the exact role of these transporters in resistance.

**Table 3:** Identification of stress response mechanisms in *S. epidermidis *RP62A1^1^

	Hit					
	
Query	Family	Description	Entry type	Clan	Bit score	E-value
SERP2244	PGA_cap	Bacterial capsule synthesis protein PGA_cap	Domain	CL0163	233.2	2.3e-69
SERP1998	AHSA1	Activator of Hsp90 ATPase homolog 1-like protein	Family	CL0209	67.8	6.9e-19

This table provides BLAST [48] results of the two putative stress response mechanisms of *S. epidermidis *RP62A we detected by iterative sequence search. PGA_cap encodes a poly-gamma-glutamate capsule, which could improve the survivability under salt stress. AHSA1 encodes an activator of the Hsp90 ATPase homolog 1-like protein, which results in an increase of efficiency of the Hsp90 function and thus leads to higher survivability under stress conditions.

Gene expression differences (Table 1) and detailed modeling of metabolism suggest that key changes are not located in just one particular subnetwork: DNA and RNA elongation is up-regulated (two-fold), and oxidative phosphorylation complex 3 is up-regulated (eight-fold). By contrast, glycolysis as well as lactate dehydrogenase (1.1.1.27) are down-regulated (by 50%).

In particular, enzymes of the oxidative phosphorylation and purine pathways are primarily affected upon application of IQ-143 (Table 4). In purine metabolism, the enzymes utilizing inosine monophosphate (IMP) are impeded as well as complex 1 and 3 (Figures 5 and 6) of oxidative phosphorylation. Also, there is a drop in activity of some DNA and RNA polymerases. Figures 2 and 3 provide detailed information on the complete metabolic effects calculated from the data using YANAsquare [22].

**Table 4:** Effects of IQ-143 on diverse enzymes of oxidative phosphorylation and energy and nucleotide metabolism of *S. aureus *USA300 and *S. epidermidis *RP62A

	**Concentration of IQ-143 (μM)**^**b**^		
	
**Enzyme**^**a**^	0.00	0.16	1.25
*S. aureus *USA300			
OP_complex1	0.0396	0.0260	0.0310
OP_complex2	0.0396	0.0260	0.0310
[OP_complex3]	0.0791	0.0520	0.0619
OP_complex4	0.0396	0.0260	0.0310
OP_complex5	0.0214	0.0109	0.0031
PurM_DNA-directed-RNA-polymerase_ATP	0.0791	0.0000	0.0000
PurM_DNA-directed-RNA-polymerase_CTP	0.0396	0.0260	0.0310
PurM_DNA-directed-RNA-polymerase_GTP	0.0396	0.0260	0.0310
PurM_DNA-directed-RNA-polymerase_UTP	0.0285	0.0229	0.0285
[SERP0831-PurM_DNA-directed-DNA-polymerase_dATP]	0.0396	0.0260	0.0121
[SERP0831-PurM_DNA-directed-DNA-polymerase_dCTP]	0.0396	0.0260	0.0310
[SERP0831-PurM_DNA-directed-DNA-polymerase_dGTP]	0.0396	0.0260	0.0310
[SERP0831-PurM_DNA-directed-DNA-polymerase_dTTP]	0.0396	0.0260	0.0310
[SERP0841-PurM_PNPase_ADP]	0.0791	0.0520	0.0619
[SERP0841-PurM_PNPase_GDP]	0.0265	0.0174	0.0207
Glyc_glyceraldehyde-3-P-dehydrogenase_NAD+	0.1582	0.1040	0.1238
Glyc_glyceraldehyde-3-P-dehydrogenase_NADP+	0.0601	0.2102	0.1251
TCA_pyruvate_dehydrogenase	0.0396	0.0260	0.0310
			
*S. epidermidis *RP62A			
OP_complex1	0.0201	0.0201	0.0126
OP_complex2	0.0161	0.0161	0.0050
[OP_complex3]	0.0361	0.0361	0.0175
OP_complex4	0.0334	0.0334	0.0292
OP_complex5	0.0669	0.0669	0.0585
PurM_DNA-directed-RNA-polymerase_CTP	0.0334	0.0334	0.0292
PurM_DNA-directed-RNA-polymerase_GTP	0.0120	0.0120	0.0436
PurM_DNA-directed-RNA-polymerase_UTP	0.0334	0.0334	0.0292
PurM_DNA-directed-RNA-polymerase_ATP	0.0334	0.0334	0.0292
[SERP0831-PurM_DNA-directed-DNA-polymerase_dATP]	0.0334	0.0334	0.0766
[SERP0831-PurM_DNA-directed-DNA-polymerase_dCTP]	0.0224	0.0224	0.0196
[SERP0831-PurM_DNA-directed-DNA-polymerase_dGTP]	0.0334	0.0334	0.0292
[SERP0831-PurM_DNA-directed-DNA-polymerase_dTTP]	0.0468	0.0468	0.0409
[SERP0841-PurM_PNPase_ADP]	0.0669	0.0669	0.0585
[SERP0841-PurM_PNPase_GDP]	0.0120	0.0120	0.0050
Glyc_glyceraldehyde-3-P-dehydrogenase_NAD+	0.0669	0.0669	0.0585
Glyc_glyceraldehyde-3-P-dehydrogenase_NADP+	0.0241	0.0241	0.0228
TCA_pyruvate_dehydrogenase	0.0161	0.0161	0.0468

This table lists the effects of three different concentrations of IQ-143 on the activity of diverse enzymes of the described pathways and reactions in *S. aureus *USA300 and *S. epidermidis *RP62A. ^a^Enzymes in brackets were also detected by their gene expression change in *S. epidermidis *RP62A. ^b^Concentrations tested were no IQ-143, 0.16 μM IQ-143 and 1.25 μM IQ-143. PurM, purine metabolism.

The changes in complexes 1 and 3 are of particular interest. These significant changes in activity suggest two possible modes of action for IQ-143: either NADH is not produced in a sufficient quantity any more due to various effects of IQ-143, or the compound competes in a direct way with NADH in certain enzymes. Regarding the first possibility, IMP-utilizing enzymes are also affected by IQ-143 if administered at a concentration of at least 1.25 μM (Tables S20 and S21 in Additional file 1). In particular, *S. epidermidis *and *S. aureus *have to use enzymes located in the glycolysis and pentose phosphate pathway to produce enough ribosylamine-5-phosphate, the initial step in IMP production. Some of these reactions use NAD^+ ^and produce NADH as a co-substrate (for example, glyceraldehyde-3-phosphate dehydrogenase in lower glycolysis). NAD^+^-utilizing enzymes are significantly down-regulated by 10 to 15% (see Tables S20 and S21 in Additional file 1). One scenario of drug action for IQ-143 predicts that if IMP synthesis is impaired (at least 1.25 μM IQ-143), there is less NADH available. This, in turn, is responsible for the drop in efficiency of complex 1 of oxidative phosphorylation, which thus also impedes complex 3. This theory is supported by the results shown in Table 5 for higher concentrations of IQ-143, where the changes in nucleotide concentrations after application of IQ-143 to *S. aureus *are shown. Whereas 0.16 μM IQ-143 reduced AMP concentration by approximately 70% (control, 0.42 μg/ml; 0.16 μM IQ-143, 0.12 μg/ml), an almost 50-fold increase in AMP concentration was observed with 1.25 μM IQ-143 (Table 6). Such an accumulation of AMP is most likely the consequence of decreased production of ATP by oxidative phosphorylation.

**Table 5:** Concentrations of CMP, AMP, GMP, XMP, and TMP

	Control		0.16 μM IQ-143			1.25 μM IQ-143		
	
	Mean (μg/ml)	SD	Mean (μg/ml)	SD	% of control	Mean (μg/ml)	SD	% of control
CMP	21.03	0.96	24.41*	0.24	116.07	3.86**	0.19	18.35
TMP	1.61	0.12	1.67	0.11	103.76	8.81*	0.24	547.20
AMP	0.42	0.06	0.12*	0.02	28.57	20.37**	0.80	4850.00
GMP	1.51	0.05	1.44**	0.05	95.36	3.66*	0.21	242.38
XMP	2.62	0.20	3.96**	0.16	151.15	3.44**	0.11	131.30

Direct measurement of CMP, AMP, GMP, XMP, and TMP concentrations in *S. aureus *(in μg/ml and in percentage of control value depending on the applied IQ-143 concentration). Statistically significant differences are indicated (*P < 0.05,**P < 0.01). SD, standard deviation.

**Table 6:** Concentration of NAD, NADH, NADP, and NADPH

	Control		0.16 μM IQ-143			1.25 μM IQ-143		
	
	Mean (μg/ml)	SD	Mean (μg/ml)	SD	% of control	Mean (μg/ml)	SD	% of control
NAD	42.19	2.44	35.68**	0.92	84.57	27.89**	0.95	66.11
NADH	3.71	0.31	2.63**	0.28	70.89	1.95**	0.21	52.56
NADP	3.47	0.06	3.24**	0.05	93.37	2.42**	0.05	69.74
NADPH	2.87	0.12	2.25**	0.02	78.40	5.56**	0.22	193.73

Direct measurement of NAD, NADH, NADP, and NADPH concentrations in *S. aureus *(in μg/ml and percentage of control value depending on the applied IQ-143 concentration). Statistically significant differences are indicated (*P < 0.05,**P < 0.01). SD, standard deviation.

A second potential mode of action for IQ-143 would be that it directly acts as a NADH competitor and impairs the production of NAD^+^. This again leads to the effects described above, although this time the reduced pool of NAD^+ ^and not the inhibition of NADH-producing enzymes is responsible for the calculated effects.

### Metabolite measurements in *S. aureus*

To better examine these possibilities, we conducted direct metabolite measurements by HPLC-UV and quantitatively measured the metabolic changes due to the administered xenobiotic IQ-143 (that is, metabonomics as defined by Nicholson [33]). In contrast to the modeled metabolic fluxes (see the previous section of Results), these are direct measurements and are used to validate and re-test the predictions regarding the resulting metabolite levels.

**Figure 7:**
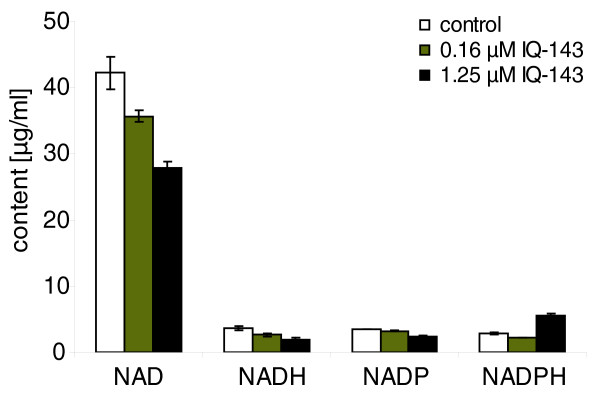
**Measured changes of nucleotides upon addition of 0.16 μM and 1.25 μM IQ-143**. The results represent mean values of triplicate measurements (± standard deviation). For further details see Tables 5 and 6.

The data show a complex pattern of cell alterations upon administration of IQ-143: the nicotinamide-adenine dinucleotides NAD^+^, NADH and NADP show substantial decreases of between 10 and 30% and between 30 and 50% dependent on the applied concentration of the inhibitor (0.16 μM and1.25 μM IQ-143, respectively; Figure 7). In turn, the reduced phosphate form NADPH undergoes a two-fold increase (with 1.25 μM IQ-143). Table 6 gives an overview of the metabolite measurements and shows significant differences in these with various concentrations of applied IQ-143.

**Figure 8:**
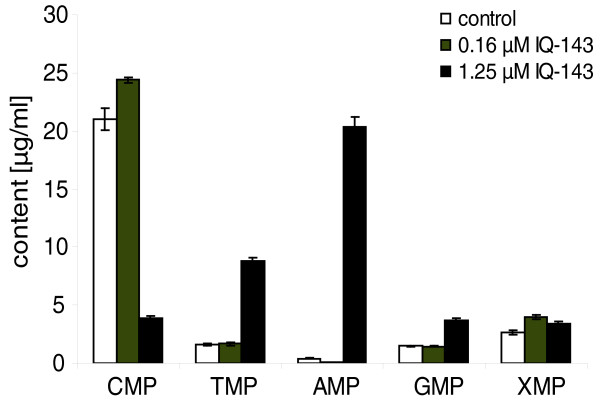
**Measured changes of energy metabolism upon addition of 0.16 μM and 1.25 μM IQ-143**. The results represent mean values of triplicate measurements (± standard deviation). For further details see Tables 5 and 6.

Additionally, strong changes occurred in the metabolite profile of purine metabolism. Pathway modeling of these data suggests down-regulation of purine metabolism as well as further effects also on the pyrimidine metabolism. Thymidine-5'-monophosphate (TMP) and cytidine-5'-monophosphate (CMP) show statistically significant changes: the concentration of TMP increased five-fold upon treatment with 1.25 μM IQ-143, and CMP production was reduced to 20% compared to the control. The lower inhibitor concentration (0.16 μM) resulted in only a slight increase in CMP. The concentrations of all nucleotides increase at high concentrations of IQ-143 (Figure 8, Table 5). By contrast, the changes with low IQ-143 concentrations are more heterogeneous.

### Metabolic effects of different concentrations of IQ-143 on human cells

The combined effects of IQ-143 are bacteriostatic and, at higher concentrations, bactericidal on *S. aureus *and *S. epidermidis*. This supports the use of IQ-143 as a potential antibiotic lead compound for the development of a novel class of antibiotics against staphylococcal infections. However, for clinical application, the therapeutic width and toxic effects in human cells have to be considered. As a start in this direction, we combined direct measurements in cultured human cells with *in vitro *measurements of enzyme activity of the CYP oxidase system involved in xenobiotic detoxification (Table 7). The direct measurements in cultured human cells revealed IC_50 _values that indicate that IQ-143 has toxic effects (see Materials and methods) on human kidney 293T human embryonal cells and macrophage (J774.1) cells at a dose of approximately 40 μM.

**Table 7:** Effects on human cytochrome P450 function *in vitro*

Cytochrome enzyme	Percentage of control activity		
	
	1 μM IQ-143	10 μM IQ-143	100 μM IQ-143
1A2	106.9	127.3	126.2
2C8	142.8	144.0	146.0
2C9	122.7	130.0	142.0
2C19	102.2	101.8	90.1
2D6	99.1	100.9	58.7
3A4	98.6	95.3	6.6

CYP enzymes derived from baculovirus-infected insect cells were incubated with the corresponding substrates and IQ-143 at concentrations of 1 μM, 10 μM, and 100 μM as described by Unger and Frank [34]. As shown here (and in Figure S3), only very high doses of IQ-143 reduced the activity of the CYP isoenzymes 2C19, 2D6, and 3A4. At concentrations below 100 μM IQ-143, the activities were even higher compared to those of the control group.

With regard to the *in vitro *measurements, we experimentally investigated the effects of IQ-143 on the six main human liver drug metabolizing CYP enzymes, 1A2, 2C8/9/19, 2D6 and 3A4, using a previously developed and well-established *in vitro *test system [15,34,35]. Only at high concentrations of IQ-143 (100 μM) was inhibition apparent: CYP 3A4 is strongly inhibited (Table 7; Figure S3 in Additional file 1); and two other enzymes are partially inhibited, CYP2C19 only slightly (5% loss of activity) and CYP2D6 moderately (40% loss of activity).

Human cells and staphylococcal cells show only few differences in their core enzyme composition of primary metabolism. For the human cell model the enzymes were compared in both organisms according to the KEGG database, extended by our own sequence analysis (Tables S1 and S4 in Additional file 1). After taking minor differences into account, we assumed as a worst-case scenario that the effects (and metabolic or gene expression changes) of IQ-143 administration in staphylocci are comparable to those in human cells. We thus applied similar constraints (gene expression changes for key enzymes) to the modified metabolic model for the human cells. To the human model we also added the detoxification pathways of the six human CYP enzymes. Applying the constraints apparent for no, low (1 μM or 10 μM), or high concentrations of IQ-143 (100 μM) - three CYP enzymes partially or fully inhibited - we tested next whether this is predicted to affect other pathway fluxes in the human cell model (for calculations see Materials and methods). With the exception of the partial block of the cytochrome isoenzymes at high concentrations of IQ-143, there was no other block predicted for any of the other pathways modeled. In accordance with the experimental observations, this would imply low toxicity of IQ-143 in cell culture.

## Discussion

### Modeling adaptation processes

There is an urgent need to find new antibiotics against staphylococci due to the emergence and alarming spread of resistant strains not only in hospitals but, more recently, also in the community. In particular, drugs belonging to novel chemical classes are of broad interest as it is assumed that resistance development against such substances will be minimized. In addition, the identification of novel targets may accelerate the finding of new lead substances combating multi-drug-resistant pathogens [36]. The compound IQ-143 has no cytotoxic effects at low concentrations in human cells compared to other isoquinoline compounds. In this study, we have included systems-wide approaches coupled with bioinformatic modeling and host-detoxification enzyme effects to elucidate the mode of action of the antimicrobial compound IQ-143 in different staphylococci; it shows direct application of systems biology in antibiotic research [37].

Our combination of theoretical modeling, analysis of enzyme activity, measurement of metabolite concentrations as well as the incorporation of gene expression data allowed us to describe in large-scale models the diverse effects of the antibiotic compound IQ-143 on the metabolism of both pathogens (*S. aureus *and *S. epidermidis*) and human host cells. These approaches are complementary to each other: direct toxicity data were only partially available and metabolite measurements covered only a range of nucleotides, including NAD(P)(H). Our work demonstrates how metabolic modeling can help to fill in missing information and how this allows predictions on the enzyme activities of the complete network, which subsequently can be verified by the experimental measurements. As a requirement for the modeling, the genome sequences were partly re-annotated.

Such a combined approach is of general use in metabonomics [33] to model, for instance, the effects of various different isoquinolines and other drugs, the effects of genetic mutations, or even more complex interactions between hosts and pathogens (for example, the metabolism of *S. aureus *under persistence in the host).

Our results suggest that IQ-143 targets the energy metabolism of *S. epidermidis *and *S. aureus *(Table 4) and we observed severely limited growth of *S. epidermidis *and *S. aureus *when IQ-143 was applied. On the other hand (as shown by array data here), gene expression for DNA and RNA polymerases was not down-regulated by IQ-143, but was instead up-regulated (up to two-fold). Our modeling can explain both findings. IQ-143 does not affect the DNA and RNA polymerase chain as initially suspected (Table 2), but rather interferes with the energy metabolism.

An example is mode 102, which consists of a pyruvate-phosphotransferase and a PEP-carboxylase. The activity of this particular mode is reduced by one-half after administration of IQ-143 in both staphylococci strains. In general, the metabolism of sugars and alcohols is reduced by IQ-143 and the investigated pathogens counteract this effect by expressing more DNA and RNA polymerases (and other enzymes) in order to maintain appropriate turnover in these pathways.

The general stress response is not strongly activated in *S. epidermidis *after administration of IQ-143 (only two genes are turned on). Several ABC transporter genes, which probably encode multiple drug efflux pumps, are turned on in the presence of IQ-143. These are typical responses of *S. epidermidis *against toxic agents [38]. However, for IQ-143 the specific pathway effects are more important and stronger.

### Metabolic implications

By analyzing CYP enzyme activity, this study enables the inhibitory potential of IQ-143 towards the major human drug metabolizing CYP enzymes to be assessed. In contrast to several previously tested naphthylisoquinoline alkaloids [15], which showed extraordinarily strong and selective inhibition of CYP2D6, IQ-143 did not show a remarkable inhibition of CYP2D6 or other tested isoenzymes at the relevant concentrations of 1 and 10 μM. Owing to the low inhibitory activity of the compound, the possibility of drug-drug interactions is very small. Even for CYP3A4, the major human CYP isoenzyme in the gut and liver, inhibition is unlikely because its activity is significantly reduced only at a concentration of 100 μM, which will not be achieved in the human body. This also reveals that certain structural characteristics might be avoided when developing new drugs from IQ-143 in order to minimize toxic effects occurring through protein inhibition.

The two investigated *Staphylococcus *species use NAD(H) as an energy source for oxidative phosphorylation. In accordance with this, the results of metabolite measurements show lower concentrations of NAD^+ ^and NADH. However, NADPH levels increased at the highest concentrations of IQ-143. Therefore, we believe that NADPH-producing enzymes (for example, of the pentose-phosphate pathway) and NADH-producing enzymes (including glyceraldehyde-3P-dehydrogenase, which can use both NADH as well as NADPH) are probably not the primary targets of the inhibition. Instead, IQ-143 has to directly affect NADH consumption. By inhibiting complex 1 of oxidative phosphorylation, NADH consumption is severely affected even at low concentrations of IQ-143; NADH is consumed at a significantly reduced rate, which leads to a smaller quantity of available NAD^+^. Glyceraldehyde-3P-dehydrogenase, however, is not affected by this, and nor are the NADPH-using enzymes. As modeling shows, this leads to much higher production of NADPH since less and less NAD^+ ^is available.

Our theory is supported by experimental findings (see 'Results: Pathway effects of different concentrations of IQ-143 in *S. epidermidis *and *S. aureus*' as well as Tables 5 and 6) and data from the literature. Aromatic substances with a quaternary nitrogen, such as the quinolinium-derived drug dequalinium chloride, tend to accumulate in mitochondria [39,40]. Also, the interference of the mitochondrial respiratory chain, especially complex I, by quaternary isoquinoline derivatives such as *N*-methylisoquinolinium ions or *N*-methyl-1,2,3,4-tetrahydroisoquinoline is well known [41,42]. Since IQ-143 is structurally related to dequalinium chloride, interaction of this newly identified antimicrobial compound with the mitochondrial respiratory chain is possible. Additionally, our findings are supported by the results in Table 5, which lists the changes in nucleotide concentrations after application of IQ-143 to *S. aureus*. Whereas 0.16 μM IQ-143 reduced the AMP concentration by approximately 70% (control, 0.42 μg/ml; 0.16 μM IQ-143, 0.12 μg/ml), an almost 50-fold increase in AMP concentration was observed using a concentration of 1.25 μM IQ-143 (Table 5). Increased AMP concentration due to a breakdown of the labile ATP molecule can be excluded because the control incubation was processed in the same way as the samples treated with IQ-143. Presumably, the accumulation of AMP points to direct inhibition of NADH oxidation by complex I of the respiratory chain because blocking electron transport leads directly to the breakdown of the chemoosmotic potential and, subsequently, oxidative phosphorylation.

The effects of secondary metabolites of the compound, host-pathogen interactions and more complex system effects have not been investigated in this work. However, since the first mouse experiments suggested that IQ-143 is toxic, this substance should currently only be considered as a lead structure for future drug development based on the promising results regarding antibiosis in staphylococci and to negate toxic effects in the host. Certainly this theoretical suggestion requires further experimental tests.

## Conclusions

Utilizing our model, the apparent bacteriostatic and, at higher concentrations, bactericidal effects of IQ-143 in *S. aureus *and *S. epidermidis *can now be described in detail according to its effects on the activity of specific enzymes and pathways in these organisms, in particular on energy metabolism and DNA/RNA elongation. IQ-143 administration affects oxidative phosphorylation but also has an impact on purine metabolism, including direct effects on purine metabolism and other nucleotide-producing enzymes at higher concentrations as well as pathway effects observable, for example, in glycolysis. These effects can be explained by the drug interfering with the NAD(H) pool and the multi-enzyme complexes of oxidative phosphorylation. The network effects can only be seen through modeling since measurements of metabolites are able to show only a small part of the whole metabolome. The metabolic effects are also not observable in the gene expression data either unless they lead to significant changes in gene expression. By applying data gathered from the metabolite measurements, the models can be fitted and thus made more accurate than when based on gene expression data alone.

This permits improvement of the lead substance (for example, pro-drug or testing of further modifications). Our combination of modeling and experimental data is generally suited to elucidate organism-wide metabolic adaptations to xenobiotics in a comparative way. Future extensions will include further data sets, such as additional data on toxicity and enzyme kinetics.

## Materials and methods

### Microarray analysis

Total RNA was isolated from *S. epidermidis *strain RP62A grown in the presence of 0.16 μM (one-quarter of the minimal inhibitory concentration) and 1.25 μM (twice the minimal inhibitory concentration) IQ-143 and without the drug. For the analysis of gene expression with subinhibitory concentrations of IQ-143, an overnight culture of *S. epidermidis *RP62A was diluted to an optical density OD_600nm _of 0.05 in a 50 ml flask. To this culture 0.16 μM IQ-143 was added and the culture was grown with agitation (200 rpm) until OD_600nm _reached 1.0. To analyzing the impact of inhibitory concentrations of IQ-143, 1.25 μM of the substance was added to the cultures in the exponential growth phase (OD_600nm _of 1.0) and the cultures were grown for an additional 10 minutes. Bacteria were harvested with the addition of RNA Protect (QIAGEN, Hilden, Germany) according to the manufacturer's instructions. Prior to RNA isolation, bacteria were lysed using glass beads in a Fast Prep shaker (Qbiogene, Heidelberg, Germany) for 45 s at a speed of 6.5 units. RNA was isolated using a QIAGEN RNeasy kit according to the standard QIAGEN RNeasy protocol.

*S. epidermidis *RP62A full genome microarrays containing PCR products of 2,282 genes/open reading frames were used for microarray analysis (Scienion, Berlin, Germany). DNA expression data have been deposited in the public databank repository Protecs [43,44] (accession [PROTECS:IQ-143]).

Total RNA (10 μg) for DNA microarray analysis isolated from cultures in the exponential growth phase was used for reverse transcription and fluorescent labeling reactions using random primers and Superscript III reverse transcriptase (Invitrogen, Darmstadt, Germany). cDNA was concomitantly labeled using the dyes Cy3 and Cy5 according to the manufacturer's instructions (Scienion, Dortmund, Germany). RNA obtained from twelve (0.16 μM) and six (1.25 μM) different biological experiments was utilized, and a reverse labeling (dye switch) experiment was performed to minimize bias due to differential dye bleaching or incorporation of the Cy3 and Cy5 dyes during the reverse transcription reaction. Microarray hybridization (16 h at 50°C) and washing of the slides were performed according to the manufacturer's instructions. Hybridized slides were scanned using a Genepix 4000B laser scanner (Axon Instruments Inc., Union City, CA, USA). Bioinformatic analyses on the slide hybridization results of each single experiment were performed using Genepix Pro3.0 (Axon Instruments Inc.). Data for each image were normalized to the mean ratio of means of all features.

### Reconstruction of metabolic networks

To model involved metabolic pathways, we used the KEGG database [24]. Additional genome annotation of missing enzyme activities for the central pathways was determined using iterative sequence and domain analysis methods [19]. Subsequent experimental verification by PCR complemented this (Tables S1, S2, S3, and S4, and Figure S6 in Additional file 1). The model of central metabolism included lipid, amino acid, and central carbohydrate metabolism as well as nucleotide and salvage pathways.

### Metabolic flux modeling

Extreme pathways possible in the annotated enzyme network were calculated first [24]. To identify actual flux strengths, we used YANAsquare [21,22] and a custom written program in R [23]. We modeled flux strengths in the metabolic webs of *S. aureus *USA300 and *S. epidermidis *RP62A according to gene expression data obtained for the purpose (Tables S5 and S6 in Additional file 1). A least square fit used first YANAsquare and next the improved R routine to calculate optimal pathway fluxes that best matched the constraints for key enzyme activities as estimated according to significant elevated or lowered enzyme expression in the above data sets (Table 1; Tables S5 and S6 in Additional file 1). Additional metabolite measurements (Figures 7 and 8) probed whether the metabolite concentrations were correctly predicted. Measured CYP activity data were considered next in the model to test whether inhibition of CYP enzymes affected other pathways in their fluxes.

Detailed input files for the pathway models are provided in Additional file 1 (for *S. aureus *USA300 in Table S2; for *S. epidermidis *RP62A inTable S3). The calculated activities of the different extreme pathway modes for no IQ-143 and two different concentrations of it are listed in Tables S7, S8, and S9 (*S. aureus*), S10, S11, and S12 (*S. epidermidis*), and S12, S13, S14, and S15 (human).

### Cell culture

Cells of *S. aureus *USA300 and *S. epidermidis *RP62A were cultured in Luria-Bertani-Medium at 30°C and shaken at 170 rpm. After 2 hours, IQ-143 was added: 0.8 μl of a 20 mM stock solution of IQ-143 in dimethyl sulfoxide was added per 100 ml cell culture to attain a concentration of 0.16 μM. For a concentration of 1.25 μM, 6.25 μl per 100 ml cell culture were added. The cells were harvested when an OD of 1.0 was reached and the metabolites were extracted. Toxicity assays in human cells were conducted according to [15]. Concentrations tested included 0.16 μM and 1.25 μM IQ-143, and a control with no antibiotic added.

### *In vitro *inhibitory activity of IQ-143 on CYP enzymes

To test the inhibitory activity of IQ-143 on the six main human drug-metabolizing CYP enzymes, we applied the method described by Unger and Frank [34]. The enzymes CYP1A2, 2C8/2C9/2C19, 2D6 and 3A4 were derived from baculovirus-infected insect cells and were incubated with different concentrations of IQ-143 (1, 10, and 100 μM).

### IC_50 _determination for human cells

J774.1 macrophages were cultured in complete medium (RPMI with NaHCO_3_, 10% fetal calf serum, 2 mM glutamine, 10 mM Hepes pH 7.2, 100 U/ml penicillin, 50 μg/ml gentamicin, 50 μM 2-mercaptoethanol) without phenol red in the absence or presence of increasing concentrations of the compounds at a cell density of 1 × 10^5 ^cells/ml (200 μl) for 24 h at 37°C, 5% CO_2 _and 95% humidity. Following the addition of 20 μl of Alamar Blue, the plates were incubated and the ODs measured at 24 h, 48 h, and 72 h. The standard Alamar blue assay was performed as previously described [45].

Kidney epithelial 293T cells (2 × 10^4 ^cells/ml) were tested in the same manner as the macrophages except that complete DMEM medium was used: 4.5 g/l solution of DMEM high D-glucose solution with sodium pyruvate but without L-glutamine, fetal bovine serum superior at a final concentration of 20%, 200 mM L-glutamine 100x.

### Commercial sources of standards for the metabolite measurements

The standards were obtained from the following suppliers. AppliChem (Darmstadt, Germany): β-nicotinamide adenine dinucleotide (NAD), β-nicotinamide adenine dinucleotide phosphate sodium salt (NADP), β-nicotinamide adenine dinucleotide reduced dipotassium salt (NADH), and β-nicotinamide adenine dinucleotide 2'-phosphate reduced tetrasodium salt (NADPH). Sigma (Taufkirchen, Germany): adenosine 5'-monophosphate sodium salt, cytidine 5'-monophosphate disodium salt, dextromethorphan, imipramine, inosine 5'-monophosphate disodium salt, guanosine 5'-monophosphate disodium salt hydrate, midazolam, paclitaxel, reserpine, tacrine, tolbutamide, thymidine 5'-monophosphate disodium salt hydrate, xanthosine 5'-monophosphate disodium salt, and sodium chloride. Fluka (Buchs, Switzerland): tributylamine and formic acid (purissimum grade). Fisher Scientific (Schwerte, Germany): methanol and acetonitrile. Natutec (Frankfurt, Germany): recombinant CYP1A2, CYP2C8, CYP2C9, CYP2C19, CYP2D6 and CYP3A4 from baculovirus-infected insect cells co-expressed with P450 reductase and cytochrome b5.

### Cell culture harvesting and HPLC

Cultivated cells (*S. aureus *and *S. epidermidis*) were quenched by adding methanol 50% (v/v). After washing the cell pellet with 0.9% sodium chloride it was extracted with methanol 80% (v/v) by means of ultrasonic treatment. After centrifugation the supernatants were directly analyzed by HPLC using an Agilent System 1100 LC (Waldbronn, Germany) consisting of a vacuum degasser, a binary pump, an autosampler, a thermostatted column compartment and an UV-visible diode array detector. System control and data processing were performed using the Agilent ChemStation Software revision A.10.01.

### Determination of purine and pyrimidine nucleotides (CMP, AMP, IMP, GMP, TMP and XMP)

The HPLC methods for the analysis of the purine and pyrimidine nucleotides were adapted from Schmitz *et al. *[46]. A sample volume of 10 μl was injected onto a 150 × 4.6 mm internal diameter, 4 μm Synergi Fusion RP column (Phenomenex, Aschaffenburg, Germany). The mobile phase consisted of water (A) and acetonitrile (B), both containing 5 mM tributylamine and 0.1% formic acid. The following gradient (percentage B) was applied: 0 to 5 minutes, 5%; 15 minutes, 20%; 18 minutes, 20%. After 18 minutes the column was flushed with 100% B for 3 minutes and re-equilibrated with 5% B. The flow rate was set to 1 ml/minute and the temperature for the column was set to 25°C. As all nucleotides show high UV absorption at about 260 nm, this wavelength was chosen for detection. All measurements were performed in triplicate. The external calibration for quantification was carried out through measurement of a mixture of the corresponding nucleotides covering a range between 0.5 and 100 μg/ml.

### Determination of nicotinamide derivatives (NAD, NADH, NADP, NADPH)

For the HPLC analysis of the nicotinamide derivatives the same method as described for the nucleotides was applied. However, the gradient (percentage B) was slightly varied: 0 to 5 minutes, 5%; 15 minutes, 50%; 18 minutes, 50%. After 18 minutes the column was flushed with 100% B for 3 minutes and re-equilibrated with 5% B.

### Statistical analysis

Statistical analysis was performed using the Mann-Whitney U-test by means of the software Statistica 8.0 (StatSoft (Europe) GmbH, 20253 Hamburg, Germany); *P*-values were calculated in relation to corresponding controls (pooled values).

### Additional data and scripts

As well as Additional file 1, other files are available from [47], containing: an introduction to pathway modeling in general and a tutorial for working with YANAsquare; input files for YANAsquare needed to calculate the extreme modes; scripts for R for calculation of the effects of changing gene expression after administration of IQ-143 (these are also used for a statistical evaluation of said effects); and scripts for PERL to import the results from R to YANAsquare.

## Abbreviations

ABC: ATP-binding cassette; CMP: cytidine-5'-monophosphate; CYP: cytochrome P450; DMEM: Dulbecco/Vogt modified Eagle's minimal essential medium; HPLC: high-performance liquid chromatography; IMP: inosine monophosphate; IQ: isoquinoline; IQ-143: synthetic analogue of the novel-type *N*:*C*-coupled naphthyl-isoquinoline alkaloid ancisheynine; KEGG: Kyoto Encyclopedia of Genes and Genomes; OD: optical density; PCR: polymerase chain reaction; SERP: *Staphylococcus epidermidis *RP62A;TMP: thymidine-5'-monophosphate; XMP: xanthosine-5'-monophosphate.

## Competing interests

The authors declare that they have no competing interests.

## Authors' contributions

AC did the genome re-annotation, the set up and calculation of the different metabolic models, culturing and harvesting of cells and was involved in data analysis of all data sets. CR conducted all metabolite measurements and cytochrome assays, and was involved in data analysis. CL was involved in programming tasks (PERL/R) and JB in database management (Protecs). KO did all gene expression analysis experiments and provided infection biology expertise. TAO did all cell toxicity tests. TG and GB selected and synthesized IQs and provided chemical expertise. UH and MU supervised CR and provided pharmaceutical expertise. In addition, MU was involved in the cytochrome assays and led and guided the metabolite measurements. TD led and guided the study, supervised AC, and was involved in the data analysis of all data sets. All authors participated in the writing of the manuscript and approved its final version.

## Supplementary Material

Additional file 1 **Supplementary materials**. Additional file 1 is a Word document containing additional data on sequence comparisons, pathway models, synthesis and effects of the IQ-143 compound, gene expression data, and nucleotide and NAD measurements, as reported in the manuscript. Click here for file
