# Supplementary material for: Modeling antibiotic and cytotoxic effects of the dimeric isoquinoline IQ-143 on metabolism and its regulation in Staphylococcus aureus, Staphylococcus epidermidis and human cells
Source: Genome Biol. 2011 Mar 21;12(3):R24. doi: 10.1186/gb-2011-12-3-r24 (PMC3129674; doi:10.1186/gb-2011-12-3-r24)

**Additional file 1 - supplementary materials:**

1. Enzymes found by iterative sequence analysis:
   1. Table S1: List of enzymes used to build metabolic webs found to be not part of KEGG data.
2. Full list of enzymes of YANAsquare modes:
   1. Table S2: List of enzmyes used to build metabolic web for *S. aureus* USA300*.*
   2. Table S3: List of enzmyes used to build metabolic web for *S. epidermidis* RP62A.
   3. Table S4: List of enzmyes used to build metabolic web for *H. sapiens.*
3. Synthesis and effects of the naptho-iso-quinoline IQ-143.
   1. Figure S1: Synthesis of IQ-143.
   2. Figure S2: Effects of IQ-143 on *S. epidermidis* RP62A.
   3. Figure S3*:* Effects on human Cytochrome P450 enzymes.
4. Gene expression data gathered by Ohlsen et al:
   1. Table S5: Gene expression data of *S. epidermidis* RP62A with 1,25µM IQ-143 added.
   2. Table S5: Gene expression data of *S. epidermidis* RP62A with 0,16µM IQ-143 added.
5. Extreme modes calculated by YANAsquare models:
   1. Table S7 to S9: Extreme modes of *S. aureus* USA300 calculated for different concentrations of IQ-143.
   2. Table S10 to S12: Extreme modes of *S. epidermidis* RP62A calculated for different concentrations of IQ-143.
   3. Table S13 to S15: Extreme modes of man calculated for different concentrations of IQ-143.
6. Extreme modes with changed activity after administration of IQ-143
   1. Table S16 and S17: Higher (S16) and lower (S17) activity for *S. epidermidis* RP62A*.*
   2. Table S18 and S19: Higher (S18) and lower (S19) activity for *S. aureus* USA300.
7. Measured concentrationsof nucleotides and NAD(P)H/NAD(P)^+^.
   1. Figure S4: Measured concentrations of NAD(P)H/NAD(P)^+^.
   2. Figure S5: Measured concentrations of nucleotides.
8. Calculated enzyme activities as calculated by R and YANAsquare:
   1. Table S20: Calculated enzyme activities for *S. aureus* USA300.
   2. Table S21: Calculated enzyme activities for *S. epidermidis* RP62A.
9. PCR Analysis
   1. Figure S6: Results for PCR detection of *in silico* predicted additional enzymes for *S. epidermidis.*
10. Mode of action: Complex 1&3 of the oxidative phosphorylation
    1. Figure S7: Mode of action of complex 1 of the oxidative phosphorylation
    2. Figure S8: Mode of action of complex 1 of the oxidative phosphorylation

**I: Enzymes found by iterative sequence analysis:**

Table S1: List of enzymes used to build metabolic webs found to be not part of KEGG data^1^.

|  | ***H. sapiens*** |  |  |  |  |
| --- | --- | --- | --- | --- | --- |
|  |  |  |  |  |  |
| EC- Number | Enzymname | Query Sequence: Brenda/NCBI | Hit: exPASY/NCBI | e- Value | Bit- Score |
| 3.6.1.5 | Apyrase | P49961 | ENTP1_HUMAN | 0,00E+000 | 1013 |
| 3.6.1.19 | nucleoside-triphosphate diphosphatase | Q9BY32 | ITPA_HUMAN | 1,00E-110 | 400 |
| 3.6.1.14 | adenosine-tetraphosphatase | Q4UK18 | ATPB_HUMAN | 0,00E+000 | 649 |
| 4.6.1.2 | guanylate cyclase | P33402 | GCYA2_HUMAN | 0,00E+000 | 1308 |
| 2.4.2.8 | inosinate pyrophosphorylase | P00492 | HPRT_HUMAN | 1,00E-123 | 442 |
| 2.4.2.4 | thymidine phosphorylase | P19971 | TYPH_HUMAN | 0,00E+000 | 868 |
| 2.7.1.74 | deoxycytidine kinase | P27707 | DCK_HUMAN | e-152 | 539 |
| 3.5.2.5 | Allantoinase | P77671 | ref|NP_001376.1| | 2,00E-032 | 136 |
| 3.6.1.41 | bis(5'-nucleosyl)-tetraphosphatase | P05637 | ref|NP_006230.2| | 8,00E-005 | 44,3 |
| 2.7.4.4 | nucleoside-phosphate kinase | NP_036606 | NP_036606 | 2,00E-147 | 518 |
| 4.2.1.70 | pseudouridylate synthase | Q96K56 | Q96K56_HUMAN | 0,00E+000 | 730 |
|  |  |  |  |  |  |
|  | ***S. epidermidis* RP62A** |  |  |  |  |
|  |  |  |  |  |  |
| EC- Number | Enzymename | Query Sequence: Brenda/NCBI | Hit: exPASY/NCBI | e- Value | Bit- Score |
| 3.6.1.19 | nucleoside-triphosphate diphosphatase | Q9BY32 | Q5HQ23 | 8.7e-16 | 177 |
| 3.6.1.3 | Adenosinetriphosphatase | A2QUY7 | Q5HME0 | 7.3e-51 | 508 |
| 2.7.1.74 | deoxycytidine kinase | P27707 | Q5HRJ1 | 1.2e-10 | 136 |
| 3.1.3.5 | 5'-nucleotidase | Q2YUP8 | Q5HKQ5 | 1.5e-19 | 244 |
| 2.4.2.22 | xanthine phosphoribosyltransferase | Q04IV9 | Q5HRX4 | 4.2e-48 | 486 |
| 2.4.2.4 | thymidine phosphorylase | Q301G5 | Q5HM85 | 2.4e-116 | 1126 |
| 2.7.1.76 | epoxyadenosine kinase | Q48VP1 | Q5HRJ0 | 2.5e-27 | 286 |
|  |  |  |  |  |  |
|  | ***S. aureus* USA300** |  |  |  |  |
| EC- Number | Enzymename | Query Sequence: Brenda/NCBI | Hit: exPASY/NCBI | e- Value | Bit- Score |
| 2.4.2.3 | uridine phosphorylase | P12758 | ref|NC_007793.1 | 2,00E-022 | 100 |
| 3.2.2.3 | uridine nucleosidase | Q88S31 | ref|NC_007793.1 | 3,00E-048 | 186 |
| 2.7.4.4 | uridine-cytidine kinase | ref|NP_036606.2 | ref|NC_007793.1 | 9,00E-042 | 163 |
| 2.7.4.10 | nucleoside-triphosphate-adenylate kinase | Q9UIJ7 | ref|NC_007793.1 | 2,00E-043 | 169 |
| 3.6.1.19 | nucleoside-triphosphate diphosphatase | Q9BY32 | ref|NC_007793.1 | 3,00E-012 | 65 |
| 2.7.7.6 | DNA-directed RNA polymerase | Q2FER5 | ref|NC_007793.1 | 1,00E-159 | 553 |
| 3.2.2.8 | riboyslpyrimidine nucleosidase | A7ZNY5 | ref|NC_007793.1 | 3,00E-060 | 225 |
| 2.7.1.74 | deoxycytidine kinase | P27707 | ref|NC_007793.1 | 3,00E-008 | 52 |
| 3.5.4.14 | deoxycytidine deaminase | O74047 | ref|NC_007793.1 | 5,00E-021 | 94 |
| 3.5.2.2 | Dihydropyrimidinase | A2UD01 | ref|NC_007793.1 | 4,00E-024 | 106 |
| 4.2.1.70 | pseudouridylate synthase | A5ISF5 | ref|NC_007793.1| | 1,00E-168 | 583 |
| 2.4.2.4 | thymidine phosphorylase | A5IUT1 | ref|NC_007793.1 | 0 | 716 |

^1^ Enzymes added through iterative sequence analysis to the enzymelist derived from KEGG.

**II: Full list of enzymes of YANAsquare modes**

Table S2: List of enzmyes used to build metabolic web for *S. aureus* USA300 ^1^.

| Enzyme name | reversible? | Reaction equation |
| --- | --- | --- |
| ADP-energy_to_ADP-metabolism | true | ADP-energy = ADP-metabolism |
| AMP-energy_to_AMP-metabolism | true | AMP-energy = AMP-metabolism |
| AS_Acetyl-CoA_to_L-Leucine | false | Acetyl-CoA + L-Glutamate + NAD+ = CO2 + L-Leucine + NADH + Oxo-Glutarate |
| AS_Acetyl-CoA_to_L-Valine | true | Acetyl-CoA + L-Glutamate = CoA-SH + Oxo-Glutarate |
| AS_Alanine_to_Pyruvate | true | Pyruvate + beta_Alanine = L-Alanine + Malonate_semi_aldehyde |
| AS_Aspartate_to_Alanine | false | L-Aspartate = CO2 + L-Alanine |
| AS_Aspartate_to_Arginine | false | ATP-energy + L-Aspartate = ADP-energy + Fumarate + L-Arginine |
| AS_Aspartate_to_Asparagine | false | ATP-energy + L-Aspartate + L-Glutamine = ADP-energy + L-Asparagine + L-Glutamate + Orthophosphate |
| AS_Aspartate_to_beta-Alanine | false | L-Aspartate = CO2 + beta_Alanine |
| AS_Aspartate_to_Homoserine | true | ATP-energy + L-Aspartate + 2 NADPH = ADP-energy + Homoserine + 2 NADP+ + Phosphate |
| AS_Glutamate_to_Glutamine | false | ATP-energy + L-Glutamate + NH3 = ADP-energy + L-Glutamine + Phosphate |
| AS_Glutamate_to_Proline | false | ATP-energy + L-Glutamate + 2 NADPH = ADP-energy + L-Proline + 2 NADP+ + 2 Phosphate |
| AS_Histidine_to_Glutamate | false | 2 H2O + L-Histidine + THF = Formamido-THF + L-Glutamate + NH3 |
| AS_Homoserine_to_Threonine | false | ATP-energy + H2O + Homoserine = ADP-energy + L-Threonine + Phosphate |
| AS_Isoleucine | false | ATP-energy + Isoleucine_ext = ADP-energy + iso-Leucine |
| AS_Leucine | false | ATP-energy + Leucine-ext = ADP-energy + L-Leucine |
| AS_Phenylalanin_to_Tyrosine | false | L-Phenylalanine + NADPH + O2 = H2O + L-Tyrosine + NADP+ |
| AS_Saccharopine_to_Lysine | true | H2O + NAD+ + Saccharopine = L-Lysine + NADH + Oxo-Glutarate |
| AS_Serine_to_Cysteine | false | Acetyl-CoA + H2S + L-Serine = Acetate + CoA-SH + L-Cysteine |
| AS_Serine_to_Glycine | true | L-Serine + THF = 5-10-Methylene-THF + H2O + L-Glycine |
| AS_Serine_to_Methionine | false | 5-10-Methylene-THF + L-Serine = L-Methionine + NH3 + Pyruvate + THF |
| AS_Serine_to_Pyruvate | false | L-Serine = NADPH + Pyruvate |
| AS_Threonine | false | ATP-energy + Threonine_ext = ADP-energy + L-Threonine |
| AS_Tryptophan_to_Tryptamine | false | L-Tryptophan = CO2 + Tryptamine |
| AS_Valine | false | ATP-energy + Valine-ext = ADP-energy + L-Valine |
| ATP-energy_to_ATP-metabolism | true | ATP-energy = ATP-metabolism |
| DNA-extern_to_DNA-intern | false | DNA-extern = DNA |
| DNA_to_DNA-blocked | false | DNA + IQ-143 = DNA_blocked + IQ-143_used |
| FA_Deg_C16_to_Acetyl-CoA | false | C16 + 6 FAD + 6 NAD+ = 6 Acetyl-CoA + 6 CoA-SH + 6 FADH2 + 6 NADH |
| FA_Syn_Acetyl-CoA_to_C16 | false | 6 Acetyl-CoA + 6 NADPH = 6 ACP + C16 + 6 CO2 + 6 H2O + 6 NADP+ |
| IQ-143-extern_to_IQ-143 | false | IQ-143_extern = IQ-143 |
| Glyc_2-Phospho-D-glycerate-2.3-phosphomutase | true | 2-Phospho-D-glycerate = 3-Phospho-D-glycerate |
| Glyc_2-phospho-D-glycerate-hydro-lyase | true | 2-Phospho-D-glycerate = H2O + Phosphoenolpyruvate |
| Glyc_6-phospho-beta-glucosidase | true | H2O + Salicin6-phosphate = Salicylalcohol + beta-D-Glucose6-phosphate |
| Glyc_6-phosphofructokinase | true | ATP-energy + beta-D-Fructose6-phosphate = ADP-energy + beta-D-Fructose16-bisphosphate |
| Glyc_acetaldehyde-dehydrogenase_NAD+ | true | Acetaldehyde + H2O + NAD+ = Acetate + H+ + NADH |
| Glyc_Actetate-CoA-ligase | true | ATP-energy + Acetate + CoA = ADP-energy + Acetyl-CoA + Pyrophosphate |
| Glyc_alpha-D-Glucose-6-phosphate-ketol-isomerase | true | alpha-D-Glucose6-phosphate = beta-D-Glucose6-phosphate |
| Glyc_alpha-D-Glucose-6-phosphate-ketol-isomerase2 | true | alpha-D-Glucose6-phosphate = beta-D-Fructose6-phosphate |
| Glyc_ATP-alpha-D-glucokinase | true | ATP-energy + alpha-D-Glucose = ADP-energy + alpha-D-Glucose6-phosphate |
| Glyc_ATP-beta-D-glucokinase | true | ATP-energy + beta-D-Glucose = ADP-metabolism + beta-D-Glucose6-phosphate |
| Glyc_beta-D-Glucose-6-phosphate-ketol-isomerase | true | beta-D-Glucose6-phosphate = beta-D-Fructose6-phosphate |
| Glyc_D-Glucose-1-epimerase | true | alpha-D-Glucose = beta-D-Glucose |
| Glyc_D-Glucose-1-epimerase-ketol-isomerase | true | (2R)-2-Hydroxy-3-(phosphonooxy)-propanal = Glyceronephosphate |
| Glyc_dihydrolipoamide-dehydrogenase | false | EnzymeN6-(dihydrolipoyl)lysine + NAD+ = EnzymeN6-(lipoyl)lysine + H+ + NADH |
| Glyc_fructose-bisphosphat-aldolase | true | beta-D-Fructose16-bisphosphate = (2R)-2-Hydroxy-3-(phosphonooxy)-propanal + Glyceronephosphate |
| Glyc_fructose-bisphosphatase | false | H2O + beta-D-Fructose16-bisphosphate = Orthophosphate + beta-D-Fructose6-phosphate |
| Glyc_glyceraldehyde-3-P-dehydrogenase_NAD+ | false | (2R)-2-Hydroxy-3-(phosphonooxy)-propanal + NAD+ + Orthophosphate = 3-Phospho-D-glyceroylphosphate + H+ + NADH |
| Glyc_glyceraldehyde-3-P-dehydrogenase_NADP+ | false | (2R)-2-Hydroxy-3-(phosphonooxy)-propanal + NADP+ + Orthophosphate = 3-Phospho-D-glyceroylphosphate + H+ + NADPH |
| Glyc_lipoic_acetyltransferase | true | Acetyl-CoA + EnzymeN6-(dihydrolipoyl)lysine = CoA + Dihydrolipoyllysine-residueacetyltransferaseS-acetyldihydrolipoyllysine |
| Glyc_phosphoglycerate-kinase | true | 3-Phospho-D-glycerate + ATP-energy = 3-Phospho-D-glyceroylphosphate + ADP-energy |
| Glyc_PTS-permease1 | true | D-Glucose + ProteinN(pi)-phospho-L-histidine = Proteinhistidine + alpha-D-Glucose6-phosphate |
| Glyc_PTS-permease2 | true | Arbutin + ProteinN(pi)-phospho-L-histidine = Arbutin6-phosphate + Proteinhistidine |
| Glyc_pyruvate_dehydrogenase | false | Pyruvate + Thiamindiphosphate = 2-(alpha-Hydroxyethyl)thiaminediphosphate + CO2 |
| Glyc_Succinate-CoA-ligase | true | ATP-energy + CoA + Succinate = ADP-energy + Orthophosphate + Succinyl-CoA |
| N-acylneuraminate-9-phosphatase | true | H2O + alpha-D-ribose-5P = AMP-metabolism + alpha-D-Ribose1-phosphate |
| OP_complex1 | false | 2 H+ + NADH + Ubichinon = 4 H+_ext + NAD+ + Ubihydrochinon |
| OP_complex2 | false | 4 H+ + Succinate + Ubichinon = Fumarate + Ubihydrochinon |
| OP_complex3 | false | 2 H+ + Ubihydrochinon + cytochrome_BC = 4 H+_ext + Ubichinon + cytochrome_C |
| OP_complex4 | false | 2 H+ + O- + cytochrome_C = 2 H+_ext + H2O |
| OP_complex5 | false | ADP-energy + 3 H+_ext + Phosphate = ATP-energy + 3 H+ + H2O |
| PurM_5-Hydroxyisourate-amidohydrolase | false | 5-Hydroxyisourate + H2O = (S)(+)-Allantoin |
| PurM_5-nucleotidase_AMP | false | AMP-metabolism + H2O = Adenosine + Orthophosphate |
| PurM_5-nucleotidase_CMP | false | CMP + H2O = Cytidine + Orthophosphate |
| PurM_5-nucleotidase_dAMP | false | H2O + dAMP = Deoxyadenosine + Orthophosphate |
| PurM_5-nucleotidase_dCMP | false | H2O + dCMP = Deoxycytidine + Orthophosphate |
| PurM_5-nucleotidase_dGMP | false | H2O + dGMP = Deoxyguanosine + Orthophosphate |
| PurM_5-nucleotidase_dTMP | false | H2O + dTMP = Orthophosphate + Thymidine |
| PurM_5-nucleotidase_GMP | false | GMP + H2O = Guanosine + Orthophosphate |
| PurM_5-nucleotidase_IMP | false | H2O + IMP = Inosine + Orthophosphate |
| PurM_5-nucleotidase_UMP | false | H2O + UMP = Orthophosphate + Uridine |
| PurM_5-nucleotidase_XMP | false | H2O + Xanthosine5-phosphate = Orthophosphate + Xanthosine |
| PurM_adenylate-kinase_AMP | true | AMP-metabolism + ATP-energy = 2 ADP-metabolism |
| PurM_adenylate-kinase_dAMP | true | ATP-energy + dAMP = ADP-energy + dADP |
| PurM_adenylosuccinate-lyase | true | N6-(12-Dicarboxyethyl)-AMP = AMP-metabolism + Fumarate |
| PurM_adenylosuccinate-lyase2 | true | 1-(5-Phosphoribosyl)-5-amino-4-(N-succinocarboxamide)-imidazole = 1-(5-Phosphoribosyl)-5-amino-4-imidazolecarboxamide + Fumarate |
| PurM_adenylylsulfate-kinase | true | ATP-energy + Adenylylsulfate = 3-phosphoadenylylsulfate + ADP-energy |
| PurM_ADP-ribose-ribophosphohydrolase | false | ADP-ribose + H2O = AMP-metabolism + D-Ribose5-phosphate |
| PurM_AICAR-pyrophosphate-phosphoribosyltransferase | true | 1-(5-Phosphoribosyl)-5-amino-4-imidazolecarboxamide + Pyrophosphate = 5-Amino-4-imidazolecarboxyamide + 5-Phospho-alpha-D-ribose1-diphosphate |
| PurM_AIR-carboxylase | true | 1-(5-Phospho-D-ribosyl)-5-amino-4-imidazolecarboxylate = Aminoimidazoleribotide + CO2 |
| PurM_allantoinase | false | Allantoate + H2O = Allantoine |
| PurM_AMP-pyrophosphorylase2 | true | AMP-metabolism + Pyrophosphate = 5-Phospho-alpha-D-ribose1-diphosphate + Adenine |
| PurM_ATP-phosphohydrolase | false | ATP-metabolism + H2O = ADP-metabolism + Orthophosphate |
| PurM_ATP_CDP-phosphotransferase | true | ATP-energy + CDP = ADP-energy + CTP |
| PurM_ATP_dADP-phosphotransferase | true | ATP-energy + dADP = ADP-energy + dATP |
| PurM_ATP_dCDP-phosphotransferase | true | ATP-energy + dCDP = ADP-energy + dCTP |
| PurM_ATP_dGDP-phosphotransferase | true | ATP-energy + dGDP = ADP-energy + dGTP |
| PurM_ATP_dIDP-phosphotransferase | true | ATP-energy + dIDP = ADP-energy + dITP |
| PurM_ATP_dTDP-phosphotransferase | true | ATP-energy + dTDP = ADP-energy + dTTP |
| PurM_ATP_dUDP-phosphotransferase | true | ATP-energy + dUDP = ADP-energy + dUTP |
| PurM_ATP_GMP-guanylate-kinase | true | ATP-energy + GMP = ADP-energy + GDP |
| PurM_ATP_GMP_guanylate-kinase | true | ATP-energy + dGMP = ADP-energy + dGDP |
| PurM_ATP_GTP-phosphotransferase | true | ATP-energy + GDP = ADP-energy + GTP |
| PurM_ATP_IDP-phosphotransferase | true | ATP-energy + IDP = ADP-energy + ITP |
| PurM_ATP_UTP-phosphotransferase | true | ATP-energy + UDP = ADP-energy + UTP |
| PurM_carbamate-kinase_ATP | true | ATP-energy + CO2 + NH3 = ADP-energy + Carbamoylphosphate |
| PurM_D-Ribose-1,5-phosphomutase | false | alpha-D-Ribose1-phosphate = D-Ribose5-phosphate |
| PurM_deoxyadenosine-kinase_ATP | false | ATP-energy + Deoxyadenosine = ADP-energy + dAMP |
| PurM_deoxycytidine-kinase_ATP | false | ATP-energy + Adenosine = ADP-energy + AMP-metabolism |
| PurM_deoxycytidine-kinase_ATP2 | false | ATP-energy + Deoxycytidine = ADP-energy + dCMP |
| PurM_dGTP-diphosphohydrolase | false | H2O + dGTP = Pyrophosphate + dGMP |
| PurM_dITP-diphosphohydrolase | false | H2O + dITP = 2-Deoxyinosine-5-phosphate + Pyrophosphate |
| PurM_DNA-directed-RNA-polyermase_ATP | false | ATP-metabolism + RNA = Pyrophosphate + RNA-A |
| PurM_DNA-directed-RNA-polyermase_CTP | false | CTP + RNA = Pyrophosphate + RNA-C |
| PurM_DNA-directed-RNA-polyermase_GTP | false | GTP + RNA = Pyrophosphate + RNA-G |
| PurM_DNA-directed-RNA-polyermase_UTP | false | RNA + UTP = Pyrophosphate + RNA-U |
| PurM_GDP-reductase | false | GMP + H+-intern + NADPH = IMP + NADP+ + NH3 |
| PurM_GMP-pyrophosphorylase2 | true | GMP + Pyrophosphate = 5-Phospho-alpha-D-ribose1-diphosphate + Guanine |
| PurM_GTP-diphosphohydrolase | false | GTP + H2O = GMP + Pyrophosphate |
| PurM_GTP-pyrophosphokinase | false | ATP-energy + GTP = ADP-energy + Guanosine3-diphosphate5-triphosphate |
| PurM_IMP-cyclohydrolase | true | H2O + IMP = 1-(5-Phosphoribosyl)-5-formamido-4-imidazolecarboxamide |
| PurM_IMP-dehydrogenase | false | H2O + IMP + NAD+ = H+-intern + NADH + Xanthosine5-phosphate |
| PurM_IMP-pyrophosphorylase | true | IMP + Pyrophosphate = 5-Phospho-alpha-D-ribose1-diphosphate + Hypoxanthine |
| PurM_IMP_L-aspartate-ligase | true | GTP + IMP + L-Aspartate = GDP + N6-(12-Dicarboxyethyl)-AMP + Orthophosphate |
| PurM_ITP-diphosphohydrolase | false | H2O + ITP = IMP + Pyrophosphate |
| PurM_metaphosphatase | true | Guanosine3-diphosphate5-triphosphate + H2O = Guanosine_3',5'-bis(diphosphate) + Orthophosphate |
| PurM_nucleoside-diphosphate-phosphotransferase_ATP | true | ADP-metabolism + ATP-energy = ADP-energy + ATP-metabolism |
| PurM_nucleotide-phosphatase_Adenine | true | Adenosine + Orthophosphate = Adenine + alpha-D-Ribose1-phosphate |
| PurM_nucleotide-phosphatase_Deoxyadenosine | true | Deoxyadenosine + Orthophosphate = 2-Deoxy-D-ribose1-phosphate + Adenine |
| PurM_nucleotide-phosphatase_Deoxyguanosine | true | Deoxyguanosine + Orthophosphate = 2-Deoxy-D-ribose1-phosphate + Guanine |
| PurM_nucleotide-phosphatase_Deoxyinosine | true | Deoxyinosine + Orthophosphate = 2-Deoxy-D-ribose1-phosphate + Hypoxanthine |
| PurM_nucleotide-phosphatase_Deoxyuridine | true | Deoxyuridine + Orthophosphate = 2-Deoxy-D-ribose1-phosphate + Uracil |
| PurM_nucleotide-phosphatase_Guanosine | true | Guanosine + Orthophosphate = Guanine + alpha-D-Ribose1-phosphate |
| PurM_nucleotide-phosphatase_Inosine | true | Inosine + Orthophosphate = Hypoxanthine + alpha-D-Ribose1-phosphate |
| PurM_nucleotide-phosphatase_Xanthosine | true | Orthophosphate + Xanthosine = Xanthine + alpha-D-Ribose1-phosphate |
| PurM_PRPP-synthetase | true | ATP-energy + D-Ribose5-phosphate = 5-Phospho-alpha-D-ribose1-diphosphate + ADP-energy |
| PurM_pyruvate-phosphotransferase_ATP | false | ATP-metabolism + Pyruvate = ADP-metabolism + Phosphoenolpyruvate |
| PurM_pyruvate-phosphotransferase_dATP | false | Pyruvate + dATP = Phosphoenolpyruvate + dADP |
| PurM_pyruvate-phosphotransferase_dGTP | false | Pyruvate + dGTP = Phosphoenolpyruvate + dGDP |
| PurM_pyruvate-phosphotransferase_GTP | false | GTP + Pyruvate = GDP + Phosphoenolpyruvate |
| PurM_SAICAR-synthetase | true | 1-(5-Phospho-D-ribosyl)-5-amino-4-imidazolecarboxylate + ATP-metabolism + L-Aspartate = 1-(5-Phosphoribosyl)-5-amino-4-(N-succinocarboxamide)-imidazole + ADP-metabolism + Orthophosphate |
| PurM_thioredoxin-oxidoreductase_dADP | false | ADP-metabolism + Thioredoxin = H2O + Oxidizedthioredoxin + dADP |
| PurM_thioredoxin-oxidoreductase_dATP | false | ATP-metabolism + Thioredoxin = H2O + Oxidizedthioredoxin + dADP |
| PurM_thioredoxin-oxidoreductase_dCDP | false | CDP + Thioredoxin = H2O + Oxidizedthioredoxin + dCDP |
| PurM_thioredoxin-oxidoreductase_dCTP | false | CTP + Thioredoxin = Oxidizedthioredoxin + dCTP |
| PurM_thioredoxin-oxidoreductase_dGDP | false | GDP + Thioredoxin = H2O + Oxidizedthioredoxin + dGDP |
| PurM_thioredoxin-oxidoreductase_dGTP | false | GTP + Thioredoxin = H2O + Oxidizedthioredoxin + dGTP |
| PurM_thioredoxin-oxidoreductase_dUDP | false | Thioredoxin + UDP = H2O + Oxidizedthioredoxin + dUDP |
| PurM_thioredoxin-oxidoreductase_dUTP | false | Thioredoxin + UTP = Oxidizedthioredoxin + dUTP |
| PurM_urea-amidohydrolase | false | H2O + Urea = CO2 + 2 NH3 |
| PurM_UTP-diphosphohydrolase | false | H2O + UTP = Pyrophosphate + UMP |
| PurM_xanthosine-phosphoribosyltransferase | true | Pyrophosphate + Xanthosine5-phosphate = 5-Phospho-alpha-D-ribose1-diphosphate + Xanthine |
| PurM_XMP-ligase | false | ATP-energy + NH3 + Xanthosine5-phosphate = ADP-energy + GMP + Pyrophosphate |
| PurM_XMP-pyrophosphorylase | true | Pyrophosphate + Xanthosine5-phosphate = 5-Phospho-alpha-D-ribose1-diphosphate + Xanthine |
| PurM_XMP_L-glutamine-amide-ligase | false | ATP-energy + H2O + L-Glutamine + Xanthosine5-phosphate = ADP-energy + GMP + L-Glutamate + Pyrophosphate |
| PurM_XTP-diphosphohydrolase | false | H2O + XTP = Pyrophosphate + Xanthosine5-phosphate |
| PyrM_2,3-cyclic-nucleotidase_CMP | false | 23-CyclicCMP + H2O = 3-CMP |
| PyrM_2,3-cyclic-nucleotidase_UMP | false | 23-CyclicUMP + H2O = 3-UMP |
| PyrM_AMP-pyrophosphorylase | true | AMP-metabolism + Pyrophosphate = 5-Phospho-alpha-D-ribose1-diphosphate + Adenine |
| PyrM_aspartate-carbamoyltransferase | false | Carbamoylphosphate + L-Aspartate = N-Carbamoyl-L-aspartate + Orthophosphate |
| PyrM_ATP_dTDP_thymidylate-kinase | true | ATP-energy + dTMP = ADP-energy + dTDP |
| PyrM_ATP_dUDP_thymidylate-kinase | true | ATP-energy + dUMP = ADP-energy + dUDP |
| PyrM_CO2_L-glutamine-amido-ligase | false | 2 ATP-energy + H2O + HCO3- + L-Glutamine = 2 ADP-energy + Carbamoylphosphate + L-Glutamate + Orthophosphate |
| PyrM_CTP-synthase | false | ATP-energy + NH3 + UTP = ADP-energy + CTP + Orthophosphate |
| PyrM_cytidilate-kinase_CTP | true | ATP-energy + CMP = ADP-energy + CDP |
| PyrM_cytidilate-kinase_dCMP | true | ATP-energy + dCMP = ADP-energy + dCDP |
| PyrM_cytidine-aminohydrolase | true | Cytidine + H2O = NH3 + Uridine |
| PyrM_cytidine-kinase | false | Cytidine + UTP = CMP + UDP |
| PyrM_cytidine-kinase_ATP | false | ATP-energy + Cytidine = ADP-energy + CMP |
| PyrM_cytidine-kinase_dATP | false | Cytidine + dATP = CMP + dADP |
| PyrM_cytidine-kinase_dCTP | false | Cytidine + dCTP-ex = CMP + dCDP-ex |
| PyrM_cytidine-kinase_dGTP | false | Cytidine + dGTP = CMP + dGDP |
| PyrM_cytidine-kinase_dTTP | false | Cytidine + dTTP-ex = CMP + dTDP-ex |
| PyrM_cytidine-kinase_dUTP | false | Cytidine + dUTP-ex = CMP + dUDP-ex |
| PyrM_cytidine-kinase_GTP | false | Cytidine + GTP = CMP + GDP |
| PyrM_cytidine-kinase_ITP | false | Cytidine + ITP = CMP + IDP |
| PyrM_cytidine-ribohydrolase | false | Cytidine + H2O = Cytosine + D-Ribose |
| PyrM_dCMP-aminohydrolase | true | H2O + dCMP = NH3 + dUMP |
| PyrM_deoxyadenosine-phosphorylase | true | Deoxyadenosine + Orthophosphate = 2-Deoxy-D-ribose1-phosphate + Adenine |
| PyrM_Deoxycytidine-aminohydrolase | true | Deoxycytidine + H2O = Deoxyuridine + NH3 |
| PyrM_Deoxycytidine-deaminase | true | Deoxycytidine + H2O = Deoxyuridine + NH3 |
| PyrM_deoxyguanosine-phosphorylase | true | Deoxyguanosine + Orthophosphate = 2-Deoxy-D-ribose1-phosphate + Guanine |
| PyrM_deoxyinosine-phosphorylase | true | Deoxyinosine + Orthophosphate = 2-Deoxy-D-ribose1-phosphate + Inosine |
| PyrM_deoxyuridine-phosphorylase | true | Deoxyuridine + Orthophosphate = 2-Deoxy-D-ribose1-phosphate + Uracil |
| PyrM_dihydroorotase | true | (S)-Dihydroorotate + H2O = N-Carbamoyl-L-aspartate |
| PyrM_dihydroorotate-oxidase | true | (S)-Dihydroorotate + Oxygen = H2O2 + Orotate |
| PyrM_dUMP-phosphotransferase | true | ATP-energy + dUMP = ADP-energy + dUDP |
| PyrM_dUTP-diphosphatase | false | H2O + dUTP = Pyrophosphate + dUMP |
| PyrM_dUTP-diphosphohydrolase | false | H2O + dUTP = Pyrophosphate + dUMP |
| PyrM_GMP-pyrophosphorylase | true | GMP + Pyrophosphate = 5-Phospho-alpha-D-ribose1-diphosphate + Guanine |
| PyrM_nucleoside-phosphate-kinase_ATP | true | ATP-energy + UMP = ADP-energy + UDP |
| PyrM_nucleoside-phosphate-kinase_ATP2 | true | ATP-energy + UMP = ADP-energy + UDP |
| PyrM_nucleoside-triphosphate-adenylate-kinase | true | ATP-energy + UTP = ADP-energy + UDP |
| PyrM_OMP-decarboxylase | false | Orotidine5-phosphate = CO2 + UMP |
| PyrM_orotate-phosphoribosyltransferase | true | Orotidine5-phosphate + Pyrophosphate = 5-Phospho-alpha-D-ribose1-diphosphate + Orotate |
| PyrM_pyrimidine-nucleoside-phosphorylase | true | Cytidine + Orthophosphate = Cytosine + alpha-D-Ribose1-phosphate |
| PyrM_thioredoxin-reductase | false | H+ + NADPH + Oxidizedthioredoxin = NADP+ + Thioredoxin |
| PyrM_thymidilate-synthase | false | 510-Methylenetetrahydrofolate + dUMP = Dihydrofolate + dTMP |
| PyrM_thymidine-kinase_dTMP | true | ATP-energy + Thymidine = ADP-energy + dTMP |
| PyrM_thymidine-kinase_dUMP | true | ATP-energy + Deoxyuridine = ADP-energy + dUMP |
| PyrM_thymidine-phosphorylase | true | Orthophosphate + Thymidine = 2-Deoxy-D-ribose1-phosphate + Thymine |
| PyrM_UMP-pyrophosphorylase | true | Pyrophosphate + UMP = 5-Phospho-alpha-D-ribose1-diphosphate + Uracil |
| PyrM_uridine-kinase_ATP | false | ATP-energy + Uridine = ADP-energy + UMP |
| PyrM_uridine-kinase_dATP | false | Uridine + dATP = UMP + dADP |
| PyrM_uridine-kinase_dCTP | false | Uridine + dCTP-ex = UMP + dCDP-ex |
| PyrM_uridine-kinase_dGTP | false | Uridine + dGTP = UMP + dGDP |
| PyrM_uridine-kinase_dTTP | false | Uridine + dTTP-ex = UMP + dTDP-ex |
| PyrM_uridine-kinase_dUTP | false | Uridine + dUTP-ex = UMP + dUDP-ex |
| PyrM_uridine-kinase_GTP | false | GTP + Uridine = GDP + UMP |
| PyrM_uridine-kinase_ITP | false | ITP + Uridine = IDP + UMP |
| PyrM_uridine-kinase_UTP | false | UTP + Uridine = UDP + UMP |
| PyrM_uridine-phosphorylase | true | Orthophosphate + Uridine = Uracil + alpha-D-Ribose1-phosphate |
| PyrM_uridine-ribohydrolase | false | H2O + Uridine = D-Ribose + Uracil |
| PyrM_UTP_L-glutamine-amido-ligase | false | ATP-energy + H2O + L-Glutamine + UTP = ADP-energy + CTP + L-Glutamate + Orthophosphate |
| SERP0290-zinc-transport_efflux | false | ATP-energy + H2O + Zn2+-intern = ADP-energy + Pyrophosphate + Zn2+-extern |
| SERP0291-zinc-transporter_import | false | ATP-energy + H2O + Zn2+-extern = ADP-energy + Pyrophosphate + Zn2+-intern |
| SERP0292-iron-dicitrate-transporter_import | false | ATP-energy + H2O + ferric-dicitrate_extern = ADP-energy + Pyrophosphate + ferric_dicitrate_intern |
| SERP0389-Glyc_Ethanol_NAD+-oxidoreductase | true | Ethanol + NAD+ = Acetaldehyde + H+ + NADH |
| SERP0653-PurM_FGAM-synthethase | false | 5-Phosphoribosyl-N-formylglycinamide + ATP-metabolism + H2O + L-Glutamine = 2-(Formamido)-N1-(5-phosphoribosyl)acetamidine + ADP-metabolism + L-Glutamate + Orthophosphate |
| SERP0655-PurM_amidophosphoribosyltransferase | false | 5-Phosphoribosylamine + L-Glutamate + Pyrophosphate = 5-Phospho-alpha-D-ribose1-diphosphate + H2O + L-Glutamine |
| SERP0656-PurM_AIR_synthetase | false | 2-(Formamido)-N1-(5-phosphoribosyl)acetamidine + ATP-metabolism = ADP-metabolism + Aminoimidazoleribotide + Orthophosphate |
| SERP0657-PurM_GAR-formyltransferase | false | 10-Formyltetrahydrofolate + 5-Phosphoribosylglycinamide = 5-Phosphoribosyl-N-formylglycinamide + Tetrahydrofolate |
| SERP0658-PurM_AICAR-formyltransferase | false | 1-(5-Phosphoribosyl)-5-amino-4-imidazolecarboxamide + 10-Formyltetrahydrofolate = 1-(5-Phosphoribosyl)-5-formamido-4-imidazolecarboxamide + Tetrahydrofolate |
| SERP0659-PurM_phosphoribosylamine-glycine-ligase | false | 5-Phosphoribosylamine + ATP-metabolism + Glycine = 5-Phosphoribosylglycinamide + ADP-metabolism + Orthophosphate |
| SERP0686-spermidine/putrescine-transport_import | false | ATP-metabolism + H2O + putrescine_extern + spermidine_extern = ADP-metabolism + 2 Pyrophosphate + putrescine_intern + spermidine_intern |
| SERP0687-spermidine/putrescine-transport_import | false | 2 ATP-metabolism + 2 H2O + putrescine_extern + spermidine_extern = 2 ADP-metabolism + 2 Pyrophosphate + putrescine_intern + spermidine_intern |
| SERP0688-spermidine/putrescine-transport_import | false | 2 ATP-metabolism + 2 H2O + putrescine_extern + spermidine_extern = 2 ADP-metabolism + 2 Pyrophosphate + putrescine_intern + spermidine_intern |
| SERP0765-Uracil-permease-transport_import | false | H+-extern + uracil_extern = H+-intern + uracil_intern |
| SERP0831-PurM_DNA-directed-DNA-polymerase_dATP | false | DNA + dATP = DNA-A + Pyrophosphate |
| SERP0831-PurM_DNA-directed-DNA-polymerase_dCTP | false | DNA + dCTP = DNA-C + Pyrophosphate |
| SERP0831-PurM_DNA-directed-DNA-polymerase_dGTP | false | DNA + dGTP = DNA-G + Pyrophosphate |
| SERP0831-PurM_DNA-directed-DNA-polymerase_dTTP | false | DNA + dTTP = DNA-T + Pyrophosphate |
| SERP0841-PurM_PNPase_ADP | false | ADP-metabolism + RNA = Orthophosphate + RNA-A |
| SERP0841-PurM_PNPase_GDP | false | GDP + RNA = Orthophosphate + RNA-G |
| SERP1403-MultiDrug-transport_efflux | false | ATP-energy + IQ-143 + H2O = ADP-energy + IQ-143_extern + Pyrophosphate |
| SERP1802-cobalt/nickel-transport_efflux | false | 2 ATP-energy + 2 H2O + cobalt-intern + nickel-intern = 2 ADP-energy + 2 Pyrophosphate + cobalt-extern + nickel-extern |
| SERP1803-cobalt/nickel-transport_efflux | false | 2 ATP-energy + 2 H2O + cobalt-intern + nickel-intern = 2 ADP-energy + 2 Pyrophosphate + cobalt-extern + nickel-extern |
| SERP1944-MultiDrug-transport_efflux | false | IQ-143 + H+-intern = IQ-143_extern + H+-extern |
| SERP1951-lipoprotein-transport_efflux/import | true | ATP-energy + H2O + lipoprotein_extern = ADP-energy + Pyrophosphate + lipoprotein_intern |
| SERP1952-macrolide-transport_efflux | false | ATP-energy + H2O + macrolide_intern = ADP-energy + Pyrophosphate + macrolide_extern |
| SERP1997-formate/nitrite-transport_efflux/import | false | H+-intern + formate_internal + nitrite_external = H+-extern + formate_extern + nitrite_intern |
| SERP2060-glyerol-transport_import | false | ATP-energy + H2O + glycerol-3-phosphate_extern = ADP-energy + Pyrophosphate + glycerol-3-phosphate_intern |
| SERP2156-Glyc_L-lactate-dehydrogenase | true | (S)-Lactate + NAD+ = H+ + NADH + Pyruvate |
| SERP2179-choline/betaine/carnitine-transp_efflux | false | H+-extern + betaine_intern + carnitine-extern + choline-extern = H+-intern + betaine-extern + carnitine-intern + choline-intern |
| SERP2186-PurM_ATP_sulfate-adenylyltransferase | false | ATP-energy + Sulfate = Adenylylsulfate + Pyrophosphate |
| SERP2283-phopsphonate-transport_import | false | ATP-energy + H2O + phosphonate_extern = ADP-energy + Pyrophosphate + phosphonate_intern |
| SERP2289-MultiDrug-transport_efflux | false | ATP-energy + IQ-143 + H2O = ADP-energy + IQ-143_extern + Pyrophosphate |
| TCA_citrate-hydro-lyase | true | Citrate = H2O + cis-Aconitate |
| TCA_citrate-hydroxymutase | true | Citrate = Isocitrate |
| TCA_citrate_synthase | true | Citrate + CoA = Acetyl-CoA + H2O + Oxaloacetate |
| TCA_fumarate-hydratase | true | (S)-Malate = Fumarate + H2O |
| TCA_isocitrate-hydro-lyase | true | Isocitrate = H2O + cis-Aconitate |
| TCA_lipoic-transsuccinylase | true | EnzymeN6-(dihydrolipoyl)lysine + Succinyl-CoA = CoA + Dihydrolipoyllysine-residuesuccinyltransferaseS-succinyldihydrolipoyllysine |
| TCA_Oxidoreductase | false | Isocitrate + NAD+ = 2-Oxoglutarate + CO2 + H+ + NADH |
| TCA_oxoglutarate-dehydrogenase-complex1 | true | 2-Oxoglutarate + Thiamindiphosphate = 3-Carboxy-1-hydroxypropyl-ThPP + CO2 |
| TCA_oxoglutarate-dehydrogenase-complex2 | true | 3-Carboxy-1-hydroxypropyl-ThPP + EnzymeN6-(lipoyl)lysine = Dihydrolipoyllysine-residuesuccinyltransferaseS-succinyldihydrolipoyllysine + Thiamindiphosphate |
| TCA_oxoglutarate-synthase | false | CO2 + Reducedferredoxin + Succinyl-CoA = 2-Oxoglutarate + CoA + Oxidizedferredoxin |
| TCA_PEP-carboxylase | true | ATP-energy + Oxaloacetate = ADP-energy + CO2 + Phosphoenolpyruvate |
| TCA_Pyruvate_CO2-ligase | true | ATP-energy + HCO3- + Pyruvate = ADP-energy + Orthophosphate + Oxaloacetate |
| TCA_pyruvate_dehydrogenase | false | 2-(alpha-Hydroxyethyl)thiaminediphosphate + EnzymeN6-(lipoyl)lysine = Dihydrolipoyllysine-residueacetyltransferaseS-acetyldihydrolipoyllysine + Thiamindiphosphate |

^1^ List of enzymes used to build the metabolic web of *S. aureus USA 300*. This list is composed of data derived from KEGG and own annotations. Abbreviations: AS: Amino acids; FA: fatty acid synthesis and degradation; Glyc: Glycolysis and Pentose Phosphate Pathways; OP: Oxidative phosphorylation; PurM: Purine Metabolism; PyrM: Pyrimidine Metabolism; TCA: Citric acid Cycle; SERP: Enzymes measured by gene expression micro array (see point IV, supplementary materials).

Table S3: List of enzmyes used to build metabolic web for *S. epidermidis* RP62A^1^.

| Enzyme name | reversible? | Reaction equation |
| --- | --- | --- |
| ADP-energy_to_ADP-metabolism | true | ADP-energy = ADP-metabolism |
| AMP-energy_to_AMP-metabolism | true | AMP-energy = AMP-metabolism |
| AS_Acetyl-CoA_to_L-Leucine | false | Acetyl-CoA + L-Glutamate + NAD+ = CO2 + L-Leucine + NADH + Oxo-Glutarate |
| AS_Acetyl-CoA_to_L-Valine | true | Acetyl-CoA + L-Glutamate = CoA-SH + Oxo-Glutarate |
| AS_Alanine_to_Pyruvate | true | Pyruvate + beta_Alanine = L-Alanine + Malonate_semi_aldehyde |
| AS_Aspartate_to_Alanine | false | L-Aspartate = CO2 + L-Alanine |
| AS_Aspartate_to_Arginine | false | ATP-energy + L-Aspartate = ADP-energy + Fumarate + L-Arginine |
| AS_Aspartate_to_Asparagine | false | ATP-energy + L-Aspartate + L-Glutamine = ADP-energy + L-Asparagine + L-Glutamate + Orthophosphate |
| AS_Aspartate_to_beta-Alanine | false | L-Aspartate = CO2 + beta_Alanine |
| AS_Aspartate_to_Homoserine | true | ATP-energy + L-Aspartate + 2 NADPH = ADP-energy + Homoserine + 2 NADP+ + Phosphate |
| AS_Glutamate_to_Glutamine | false | ATP-energy + L-Glutamate + NH3 = ADP-energy + L-Glutamine + Phosphate |
| AS_Glutamate_to_Proline | false | ATP-energy + L-Glutamate + 2 NADPH = ADP-energy + L-Proline + 2 NADP+ + 2 Phosphate |
| AS_Histidine_to_Glutamate | false | 2 H2O + L-Histidine + THF = Formamido-THF + L-Glutamate + NH3 |
| AS_Homoserine_to_Threonine | false | ATP-energy + H2O + Homoserine = ADP-energy + L-Threonine + Phosphate |
| AS_Isoleucine | false | ATP-energy + Isoleucine_ext = ADP-energy + iso-Leucine |
| AS_Leucine | false | ATP-energy + Leucine-ext = ADP-energy + L-Leucine |
| AS_Phenylalanin_to_Tyrosine | false | L-Phenylalanine + NADPH + O2 = H2O + L-Tyrosine + NADP+ |
| AS_Saccharopine_to_Lysine | true | H2O + NAD+ + Saccharopine = L-Lysine + NADH + Oxo-Glutarate |
| AS_Serine_to_Cysteine | false | Acetyl-CoA + H2S + L-Serine = Acetate + CoA-SH + L-Cysteine |
| AS_Serine_to_Glycine | true | L-Serine + THF = 5-10-Methylene-THF + H2O + L-Glycine |
| AS_Serine_to_Methionine | false | 5-10-Methylene-THF + L-Serine = L-Methionine + NH3 + Pyruvate + THF |
| AS_Serine_to_Pyruvate | false | L-Serine = NADPH + Pyruvate |
| AS_Threonine | false | ATP-energy + Threonine_ext = ADP-energy + L-Threonine |
| AS_Tryptophan_to_Tryptamine | false | L-Tryptophan = CO2 + Tryptamine |
| AS_Valine | false | ATP-energy + Valine-ext = ADP-energy + L-Valine |
| ATP-energy_to_ATP-metabolism | true | ATP-energy = ATP-metabolism |
| DNA-extern_to_DNA-intern | false | DNA-extern = DNA |
| DNA_to_DNA-blocked | false | DNA + IQ-143 = DNA_blocked + IQ-143_used |
| FA_Deg_C16_to_Acetyl-CoA | false | C16 + 6 FAD + 6 NAD+ = 6 Acetyl-CoA + 6 CoA-SH + 6 FADH2 + 6 NADH |
| FA_Syn_Acetyl-CoA_to_C16 | false | 6 Acetyl-CoA + 6 NADPH = 6 ACP + C16 + 6 CO2 + 6 H2O + 6 NADP+ |
| IQ-143-extern_to_IQ-143 | false | IQ-143-extern = IQ-143 |
| Glyc_2-Phospho-D-glycerate-2.3-phosphomutase | true | 2-Phospho-D-glycerate = 3-Phospho-D-glycerate |
| Glyc_2-phospho-D-glycerate-hydro-lyase | true | 2-Phospho-D-glycerate = H2O + Phosphoenolpyruvate |
| Glyc_6-phospho-beta-glucosidase | true | H2O + Salicin6-phosphate = Salicylalcohol + beta-D-Glucose6-phosphate |
| Glyc_6-phosphofructokinase | true | ATP-energy + beta-D-Fructose6-phosphate = ADP-energy + beta-D-Fructose16-bisphosphate |
| Glyc_acetaldehyde-dehydrogenase_NAD+ | true | Acetaldehyde + H2O + NAD+ = Acetate + H+ + NADH |
| Glyc_Actetate-CoA-ligase | true | ATP-energy + Acetate + CoA = ADP-energy + Acetyl-CoA + Pyrophosphate |
| Glyc_alpha-D-Glucose-6-phosphate-ketol-isomerase | true | alpha-D-Glucose6-phosphate = beta-D-Glucose6-phosphate |
| Glyc_alpha-D-Glucose-6-phosphate-ketol-isomerase2 | true | alpha-D-Glucose6-phosphate = beta-D-Fructose6-phosphate |
| Glyc_ATP-alpha-D-glucokinase | true | ATP-metabolism + alpha-D-Glucose = ADP-metabolism + alpha-D-Glucose6-phosphate |
| Glyc_ATP-beta-D-glucokinase | true | ATP-energy + beta-D-Glucose = ADP-metabolism + beta-D-Glucose6-phosphate |
| Glyc_beta-D-Glucose-6-phosphate-ketol-isomerase | true | beta-D-Glucose6-phosphate = beta-D-Fructose6-phosphate |
| Glyc_D-Glucose-1-epimerase | true | alpha-D-Glucose = beta-D-Glucose |
| Glyc_D-Glucose-1-epimerase-ketol-isomerase | true | (2R)-2-Hydroxy-3-(phosphonooxy)-propanal = Glyceronephosphate |
| Glyc_dihydrolipoamide-dehydrogenase | false | EnzymeN6-(dihydrolipoyl)lysine + NAD+ = EnzymeN6-(lipoyl)lysine + H+ + NADH |
| Glyc_fructose-bisphosphat-aldolase | true | beta-D-Fructose16-bisphosphate = (2R)-2-Hydroxy-3-(phosphonooxy)-propanal + Glyceronephosphate |
| Glyc_fructose-bisphosphatase | false | H2O + beta-D-Fructose16-bisphosphate = Orthophosphate + beta-D-Fructose6-phosphate |
| Glyc_glyceraldehyde-3-P-dehydrogenase_NAD+ | false | (2R)-2-Hydroxy-3-(phosphonooxy)-propanal + NAD+ + Orthophosphate = 3-Phospho-D-glyceroylphosphate + H+ + NADH |
| Glyc_glyceraldehyde-3-P-dehydrogenase_NADP+ | false | (2R)-2-Hydroxy-3-(phosphonooxy)-propanal + NADP+ + Orthophosphate = 3-Phospho-D-glyceroylphosphate + H+ + NADPH |
| Glyc_lipoic_acetyltransferase | true | Acetyl-CoA + EnzymeN6-(dihydrolipoyl)lysine = CoA + Dihydrolipoyllysine-residueacetyltransferaseS-acetyldihydrolipoyllysine |
| Glyc_phosphoglycerate-kinase | true | 3-Phospho-D-glycerate + ATP-energy = 3-Phospho-D-glyceroylphosphate + ADP-energy |
| Glyc_PTS-permease1 | true | D-Glucose + ProteinN(pi)-phospho-L-histidine = Proteinhistidine + alpha-D-Glucose6-phosphate |
| Glyc_PTS-permease2 | true | Arbutin + ProteinN(pi)-phospho-L-histidine = Arbutin6-phosphate + Proteinhistidine |
| Glyc_pyruvate_dehydrogenase | false | Pyruvate + Thiamindiphosphate = 2-(alpha-Hydroxyethyl)thiaminediphosphate + CO2 |
| Glyc_Succinate-CoA-ligase | true | ATP-energy + CoA + Succinate = ADP-energy + Orthophosphate + Succinyl-CoA |
| OP_complex1 | false | 2 H+ + NADH + Ubichinon = 4 H+_ext + NAD+ + Ubihydrochinon |
| OP_complex2 | false | 4 H+ + Succinate + Ubichinon = Fumarate + Ubihydrochinon |
| OP_complex3 | false | 2 H+ + Ubihydrochinon + cytochrome_BC = 4 H+_ext + Ubichinon + cytochrome_C |
| OP_complex4 | false | 2 H+ + O- + cytochrome_C = 2 H+_ext + H2O |
| OP_complex5 | false | ADP-energy + 3 H+_ext + Phosphate = ATP-energy + 3 H+ + H2O |
| PurM_5-nucleotidase_AMP | false | AMP-metabolism + H2O = Adenosine + Orthophosphate |
| PurM_5-nucleotidase_CMP | false | CMP + H2O = Cytidine + Orthophosphate |
| PurM_5-nucleotidase_dAMP | false | H2O + dAMP = Deoxyadenosine + Orthophosphate |
| PurM_5-nucleotidase_dCMP | false | H2O + dCMP = Deoxycytidine + Orthophosphate |
| PurM_5-nucleotidase_dGMP | false | H2O + dGMP = Deoxyguanosine + Orthophosphate |
| PurM_5-nucleotidase_dTMP | false | H2O + dTMP = Orthophosphate + Thymidine |
| PurM_5-nucleotidase_GMP | false | GMP + H2O = Guanosine + Orthophosphate |
| PurM_5-nucleotidase_IMP | false | H2O + IMP = Inosine + Orthophosphate |
| PurM_5-nucleotidase_UMP | false | H2O + UMP = Orthophosphate + Uridine |
| PurM_5-nucleotidase_XMP | false | H2O + Xanthosine5-phosphate = Orthophosphate + Xanthosine |
| PurM_adenylate-kinase_AMP | true | AMP-metabolism + ATP-energy = 2 ADP-metabolism |
| PurM_adenylate-kinase_dAMP | true | ATP-energy + dAMP = ADP-energy + dADP |
| PurM_adenylosuccinate-lyase | true | N6-(12-Dicarboxyethyl)-AMP = AMP-metabolism + Fumarate |
| PurM_adenylosuccinate-lyase2 | true | 1-(5-Phosphoribosyl)-5-amino-4-(N-succinocarboxamide)-imidazole = 1-(5-Phosphoribosyl)-5-amino-4-imidazolecarboxamide + Fumarate |
| PurM_adenylylsulfate-kinase | true | ATP-energy + Adenylylsulfate = 3-phosphoadenylylsulfate + ADP-energy |
| PurM_ADP-ribose-ribophosphohydrolase | false | ADP-ribose + H2O = AMP-metabolism + D-Ribose5-phosphate |
| PurM_AICAR-pyrophosphate-phosphoribosyltransferase | true | 1-(5-Phosphoribosyl)-5-amino-4-imidazolecarboxamide + Pyrophosphate = 5-Amino-4-imidazolecarboxyamide + 5-Phospho-alpha-D-ribose1-diphosphate |
| PurM_AIR-carboxylase | true | 1-(5-Phospho-D-ribosyl)-5-amino-4-imidazolecarboxylate = Aminoimidazoleribotide + CO2 |
| PurM_AMP-pyrophosphorylase | true | AMP-metabolism + Pyrophosphate = 5-Phospho-alpha-D-ribose1-diphosphate + Adenine |
| PurM_AMP-pyrophosphorylase2 | true | AMP-metabolism + Pyrophosphate = 5-Phospho-alpha-D-ribose1-diphosphate + Adenine |
| PurM_ATP-phosphohydrolase | false | ATP-metabolism + H2O = ADP-metabolism + Orthophosphate |
| PurM_ATP_CDP-phosphotransferase | true | ATP-energy + CDP = ADP-energy + CTP |
| PurM_ATP_dADP-phosphotransferase | true | ATP-energy + dADP = ADP-energy + dATP |
| PurM_ATP_dCDP-phosphotransferase | true | ATP-energy + dCDP = ADP-energy + dCTP |
| PurM_ATP_dGDP-phosphotransferase | true | ATP-energy + dGDP = ADP-energy + dGTP |
| PurM_ATP_dIDP-phosphotransferase | true | ATP-energy + dIDP = ADP-energy + dITP |
| PurM_ATP_dTDP-phosphotransferase | true | ATP-energy + dTDP = ADP-energy + dTTP |
| PurM_ATP_dUDP-phosphotransferase | true | ATP-energy + dUDP = ADP-energy + dUTP |
| PurM_ATP_GMP-guanylate-kinase | true | ATP-energy + GMP = ADP-energy + GDP |
| PurM_ATP_GMP_guanylate-kinase | true | ATP-energy + dGMP = ADP-energy + dGDP |
| PurM_ATP_GTP-phosphotransferase | true | ATP-energy + GDP = ADP-energy + GTP |
| PurM_ATP_IDP-phosphotransferase | true | ATP-energy + IDP = ADP-energy + ITP |
| PurM_ATP_UTP-phosphotransferase | true | ATP-energy + UDP = ADP-energy + UTP |
| PurM_carbamate-kinase_ATP | true | ATP-energy + CO2 + NH3 = ADP-energy + Carbamoylphosphate |
| PurM_D-Ribose-1,5-phosphomutase | false | alpha-D-Ribose1-phosphate = D-Ribose5-phosphate |
| PurM_deoxyadenosine-kinase_ATP | false | ATP-energy + Deoxyadenosine = ADP-energy + dAMP |
| PurM_deoxycytidine-kinase_ATP | false | ATP-energy + Adenosine = ADP-energy + AMP-metabolism |
| PurM_deoxycytidine-kinase_ATP2 | false | ATP-energy + Deoxycytidine = ADP-energy + dCMP |
| PurM_dGTP-diphosphohydrolase | false | H2O + dGTP = Pyrophosphate + dGMP |
| PurM_dITP-diphosphohydrolase | false | H2O + dITP = 2-Deoxyinosine-5-phosphate + Pyrophosphate |
| PurM_DNA-directed-RNA-polyermase_CTP | false | CTP + RNA = Pyrophosphate + RNA-C |
| PurM_DNA-directed-RNA-polyermase_GTP | false | GTP + RNA = Pyrophosphate + RNA-G |
| PurM_DNA-directed-RNA-polyermase_UTP | false | RNA + UTP = Pyrophosphate + RNA-U |
| PurM_DNA-directed-RNA-polymerase_ATP | false | ATP-metabolism + RNA = Pyrophosphate + RNA-A |
| PurM_GDP-reductase | false | GMP + H+-intern + NADPH = IMP + NADP+ + NH3 |
| PurM_GMP-pyrophosphorylase | true | GMP + Pyrophosphate = 5-Phospho-alpha-D-ribose1-diphosphate + Guanine |
| PurM_GMP-pyrophosphorylase2 | true | GMP + Pyrophosphate = 5-Phospho-alpha-D-ribose1-diphosphate + Guanine |
| PurM_GTP-diphosphohydrolase | false | GTP + H2O = GMP + Pyrophosphate |
| PurM_GTP-pyrophosphokinase | false | ATP-energy + GTP = ADP-energy + Guanosine3-diphosphate5-triphosphate |
| PurM_IMP-cyclohydrolase | true | H2O + IMP = 1-(5-Phosphoribosyl)-5-formamido-4-imidazolecarboxamide |
| PurM_IMP-dehydrogenase | false | H2O + IMP + NAD+ = H+-intern + NADH + Xanthosine5-phosphate |
| PurM_IMP-pyrophosphorylase | true | IMP + Pyrophosphate = 5-Phospho-alpha-D-ribose1-diphosphate + Hypoxanthine |
| PurM_IMP_L-aspartate-ligase | true | GTP + IMP + L-Aspartate = GDP + N6-(12-Dicarboxyethyl)-AMP + Orthophosphate |
| PurM_ITP-diphosphohydrolase | false | H2O + ITP = IMP + Pyrophosphate |
| PurM_metaphosphatase | true | Guanosine3-diphosphate5-triphosphate + H2O = Guanosine_3',5'-bis(diphosphate) + Orthophosphate |
| PurM_nucleoside-diphosphate-phosphotransferase_ATP | true | ADP-metabolism + ATP-energy = ADP-energy + ATP-metabolism |
| PurM_nucleotide-phosphatase_Adenine | true | Adenosine + Orthophosphate = Adenine + alpha-D-Ribose1-phosphate |
| PurM_nucleotide-phosphatase_Deoxyadenosine | true | Deoxyadenosine + Orthophosphate = 2-Deoxy-D-ribose1-phosphate + Adenine |
| PurM_nucleotide-phosphatase_Deoxyguanosine | true | Deoxyguanosine + Orthophosphate = 2-Deoxy-D-ribose1-phosphate + Guanine |
| PurM_nucleotide-phosphatase_Deoxyinosine | true | Deoxyinosine + Orthophosphate = 2-Deoxy-D-ribose1-phosphate + Hypoxanthine |
| PurM_nucleotide-phosphatase_Deoxyuridine | true | Deoxyuridine + Orthophosphate = 2-Deoxy-D-ribose1-phosphate + Uracil |
| PurM_nucleotide-phosphatase_Guanosine | true | Guanosine + Orthophosphate = Guanine + alpha-D-Ribose1-phosphate |
| PurM_nucleotide-phosphatase_Inosine | true | Inosine + Orthophosphate = Hypoxanthine + alpha-D-Ribose1-phosphate |
| PurM_nucleotide-phosphatase_Xanthosine | true | Orthophosphate + Xanthosine = Xanthine + alpha-D-Ribose1-phosphate |
| PurM_PRPP-synthetase | true | ATP-energy + D-Ribose5-phosphate = 5-Phospho-alpha-D-ribose1-diphosphate + ADP-energy |
| PurM_pyruvate-phosphotransferase_ATP | false | ATP-metabolism + Pyruvate = ADP-metabolism + Phosphoenolpyruvate |
| PurM_pyruvate-phosphotransferase_dATP | false | Pyruvate + dATP = Phosphoenolpyruvate + dADP |
| PurM_pyruvate-phosphotransferase_dGTP | false | Pyruvate + dGTP = Phosphoenolpyruvate + dGDP |
| PurM_pyruvate-phosphotransferase_GTP | false | GTP + Pyruvate = GDP + Phosphoenolpyruvate |
| PurM_SAICAR-synthetase | true | 1-(5-Phospho-D-ribosyl)-5-amino-4-imidazolecarboxylate + ATP-metabolism + L-Aspartate = 1-(5-Phosphoribosyl)-5-amino-4-(N-succinocarboxamide)-imidazole + ADP-metabolism + Orthophosphate |
| PurM_thioredoxin-oxidoreductase_dATP | false | ADP-metabolism + Thioredoxin = H2O + Oxidizedthioredoxin + dADP |
| PurM_thioredoxin-oxidoreductase_dCDP | false | CDP + Thioredoxin = H2O + Oxidizedthioredoxin + dCDP |
| PurM_thioredoxin-oxidoreductase_dCTP | false | CTP + Thioredoxin = Oxidizedthioredoxin + dCTP |
| PurM_thioredoxin-oxidoreductase_dGDP | false | GDP + Thioredoxin = H2O + Oxidizedferredoxin + dGDP |
| PurM_thioredoxin-oxidoreductase_dGTP | false | GTP + Thioredoxin = H2O + Oxidizedthioredoxin + dGTP |
| PurM_thioredoxin-oxidoreductase_dUDP | false | Thioredoxin + UDP = H2O + Oxidizedthioredoxin + dUDP |
| PurM_thioredoxin-oxidoreductase_dUTP | false | Thioredoxin + UTP = Oxidizedthioredoxin + dUTP |
| PurM_thioredoxin-oxidoreductased_dADP | false | ADP-metabolism + Thioredoxin = H2O + Oxidizedthioredoxin + dADP |
| PurM_urea-amidohydrolase | false | H2O + Urea = CO2 + 2 NH3 |
| PurM_UTP-diphosphohydrolase | false | H2O + UTP = Pyrophosphate + UMP |
| PurM_xanthosine-phosphoribosyltransferase | true | Pyrophosphate + Xanthosine5-phosphate = 5-Phospho-alpha-D-ribose1-diphosphate + Xanthine |
| PurM_XMP-pyrophosphorylase | true | Pyrophosphate + Xanthosine5-phosphate = 5-Phospho-alpha-D-ribose1-diphosphate + Xanthine |
| PurM_XMP_L-glutamine-amide-ligase | false | ATP-energy + H2O + L-Glutamine + Xanthosine5-phosphate = ADP-energy + GMP + L-Glutamate + Pyrophosphate |
| PurM_XTP-diphosphohydrolase | false | H2O + XTP = Pyrophosphate + Xanthosine5-phosphate |
| PyrM_2,3-cyclic-nucleotidase_CMP | false | 23-CyclicCMP + H2O = 3-CMP |
| PyrM_2,3-cyclic-nucleotidase_UMP | false | 23-CyclicUMP + H2O = 3-UMP |
| PyrM_aspartate-carbamoyltransferase | false | Carbamoylphosphate + L-Aspartate = N-Carbamoyl-L-aspartate + Orthophosphate |
| PyrM_ATP_dTDP_thymidylate-kinase | true | ATP-energy + dTMP = ADP-energy + dTDP |
| PyrM_ATP_dUDP_thymidylate-kinase | true | ATP-energy + dUMP = ADP-energy + dUDP |
| PyrM_CO2_L-glutamine-amido-ligase | false | 2 ATP-energy + H2O + HCO3- + L-Glutamine = 2 ADP-energy + Carbamoylphosphate + L-Glutamate + Orthophosphate |
| PyrM_CTP-synthase | false | ATP-energy + NH3 + UTP = ADP-energy + CTP + Orthophosphate |
| PyrM_cytidilate-kinase_CTP | true | ATP-energy + CMP = ADP-energy + CDP |
| PyrM_cytidilate-kinase_dCMP | true | ATP-energy + dCMP = ADP-energy + dCDP |
| PyrM_cytidine-aminohydrolase | true | Cytidine + H2O = NH3 + Uridine |
| PyrM_cytidine-kinase_ATP | false | ATP-energy + Cytidine = ADP-energy + CMP |
| PyrM_cytidine-kinase_dATP | false | Cytidine + dATP = CMP + dADP |
| PyrM_cytidine-kinase_dCTP | false | Cytidine + dCTP-ex = CMP + dCDP-ex |
| PyrM_cytidine-kinase_dGTP | false | Cytidine + dGTP = CMP + dGDP |
| PyrM_cytidine-kinase_dTTP | false | Cytidine + dTTP-ex = CMP + dTDP-ex |
| PyrM_cytidine-kinase_dUTP | false | Cytidine + dUTP-ex = CMP + dUDP-ex |
| PyrM_cytidine-kinase_GTP | false | Cytidine + GTP = CMP + GDP |
| PyrM_cytidine-kinase_ITP | false | Cytidine + ITP = CMP + IDP |
| PyrM_cytidine-kinase_UTP | false | Cytidine + UTP = CMP + UDP |
| PyrM_cytidine-ribohydrolase | false | Cytidine + H2O = Cytosine + D-Ribose |
| PyrM_dCMP-aminohydrolase | true | H2O + dCMP = NH3 + dUMP |
| PyrM_deoxyadenosine-phosphorylase | true | Deoxyadenosine + Orthophosphate = 2-Deoxy-D-ribose1-phosphate + Adenine |
| PyrM_Deoxycytidine-aminohydrolase | true | Deoxycytidine + H2O = Deoxyuridine + NH3 |
| PyrM_Deoxycytidine-deaminase | true | Deoxycytidine + H2O = Deoxyuridine + NH3 |
| PyrM_deoxyguanosine-phosphorylase | true | Deoxyguanosine + Orthophosphate = 2-Deoxy-D-ribose1-phosphate + Guanine |
| PyrM_deoxyinosine-phosphorylase | true | Deoxyinosine + Orthophosphate = 2-Deoxy-D-ribose1-phosphate + Inosine |
| PyrM_deoxyuridine-phosphorylase | true | Deoxyuridine + Orthophosphate = 2-Deoxy-D-ribose1-phosphate + Uracil |
| PyrM_dihydroorotase | true | (S)-Dihydroorotate + H2O = N-Carbamoyl-L-aspartate |
| PyrM_dihydroorotate-oxidase | true | (S)-Dihydroorotate + Oxygen = H2O2 + Orotate |
| PyrM_dUMP-phosphotransferase | true | ATP-energy + dUMP = ADP-energy + dUDP |
| PyrM_dUTP-diphosphatase | false | H2O + dUTP = Pyrophosphate + dUMP |
| PyrM_dUTP-diphosphohydrolase | false | H2O + dUTP = Pyrophosphate + dUMP |
| PyrM_nucleoside-phosphate-kinase_ATP | true | ATP-energy + UMP = ADP-energy + UDP |
| PyrM_nucleoside-phosphate-kinase_ATP2 | true | ATP-energy + UMP = ADP-energy + UDP |
| PyrM_nucleoside-triphosphate-adenylate-kinase | true | ATP-energy + UTP = ADP-energy + UDP |
| PyrM_OMP-decarboxylase | false | Orotidine5-phosphate = CO2 + UMP |
| PyrM_orotate-phosphoribosyltransferase | true | Orotidine5-phosphate + Pyrophosphate = 5-Phospho-alpha-D-ribose1-diphosphate + Orotate |
| PyrM_pyrimidine-nucleoside-phosphorylase | true | Cytidine + Orthophosphate = Cytosine + alpha-D-Ribose1-phosphate |
| PyrM_thioredoxin-reductase | false | H+ + NADPH + Oxidizedthioredoxin = NADP+ + Thioredoxin |
| PyrM_thymidilate-synthase | false | 510-Methylenetetrahydrofolate + dUMP = Dihydrofolate + dTMP |
| PyrM_thymidine-kinase_dTMP | true | ATP-energy + Thymidine = ADP-energy + dTMP |
| PyrM_thymidine-kinase_dUMP | true | ATP-energy + Deoxyuridine = ADP-energy + dUMP |
| PyrM_thymidine-phosphorylase | true | Orthophosphate + Thymidine = 2-Deoxy-D-ribose1-phosphate + Thymine |
| PyrM_UMP-pyrophosphorylase | true | Pyrophosphate + UMP = 5-Phospho-alpha-D-ribose1-diphosphate + Uracil |
| PyrM_uridine-kinase_ATP | false | ATP-energy + Uridine = ADP-energy + UMP |
| PyrM_uridine-kinase_dATP | false | Uridine + dATP = UMP + dADP |
| PyrM_uridine-kinase_dCTP | false | Uridine + dCTP-ex = UMP + dCDP-ex |
| PyrM_uridine-kinase_dGTP | false | Uridine + dGTP = UMP + dGDP |
| PyrM_uridine-kinase_dTTP | false | Uridine + dTTP-ex = UMP + dTDP-ex |
| PyrM_uridine-kinase_dUTP | false | Uridine + dUTP-ex = UMP + dUDP-ex |
| PyrM_uridine-kinase_GTP | false | GTP + Uridine = GDP + UMP |
| PyrM_uridine-kinase_ITP | false | ITP + Uridine = IDP + UMP |
| PyrM_uridine-kinase_UTP | false | UTP + Uridine = UDP + UMP |
| PyrM_uridine-phosphorylase | true | Orthophosphate + Uridine = Uracil + alpha-D-Ribose1-phosphate |
| PyrM_uridine-ribohydrolase | false | H2O + Uridine = D-Ribose + Uracil |
| PyrM_UTP_L-glutamine-amido-ligase | false | ATP-energy + H2O + L-Glutamine + UTP = ADP-energy + CTP + L-Glutamate + Orthophosphate |
| SERP0290-zinc-transport_efflux | false | ATP-energy + H2O + Zn2+-intern = ADP-energy + Pyrophosphate + Zn2+-extern |
| SERP0291-zinc-transporter_import | false | ATP-energy + H2O + Zn2+-extern = ADP-energy + Pyrophosphate + Zn2+-intern |
| SERP0292-iron-dicitrate-transporter_import | false | ATP-energy + H2O + ferric-dicitrate_extern = ADP-energy + Pyrophosphate + ferric_dicitrate_intern |
| SERP0389-Glyc_Ethanol_NAD+-oxidoreductase | true | Ethanol + NAD+ = Acetaldehyde + H+ + NADH |
| SERP0653-PurM_FGAM-synthethase | false | 5-Phosphoribosyl-N-formylglycinamide + ATP-metabolism + H2O + L-Glutamine = 2-(Formamido)-N1-(5-phosphoribosyl)acetamidine + ADP-metabolism + L-Glutamate + Orthophosphate |
| SERP0655-PurM_amidophosphoribosyltransferase | false | 5-Phosphoribosylamine + L-Glutamate + Pyrophosphate = 5-Phospho-alpha-D-ribose1-diphosphate + H2O + L-Glutamine |
| SERP0656-PurM_AIR_synthetase | false | 2-(Formamido)-N1-(5-phosphoribosyl)acetamidine + ATP-metabolism = ADP-metabolism + Aminoimidazoleribotide + Orthophosphate |
| SERP0657-PurM_GAR-formyltransferase | false | 10-Formyltetrahydrofolate + 5-Phosphoribosylglycinamide = 5-Phosphoribosyl-N-formylglycinamide + Tetrahydrofolate |
| SERP0658-PurM_AICAR-formyltransferase | false | 1-(5-Phosphoribosyl)-5-amino-4-imidazolecarboxamide + 10-Formyltetrahydrofolate = 1-(5-Phosphoribosyl)-5-formamido-4-imidazolecarboxamide + Tetrahydrofolate |
| SERP0659-PurM_phosphoribosylamine-glycine-ligase | false | 5-Phosphoribosylamine + ATP-metabolism + Glycine = 5-Phosphoribosylglycinamide + ADP-metabolism + Orthophosphate |
| SERP0686-spermidine/putrescine-transport_import | false | ATP-metabolism + H2O + putrescine_extern + spermidine_extern = ADP-metabolism + 2 Pyrophosphate + putrescine_intern + spermidine_intern |
| SERP0687-spermidine/putrescine-transport_import | false | 2 ATP-metabolism + 2 H2O + putrescine_extern + spermidine_extern = 2 ADP-metabolism + 2 Pyrophosphate + putrescine_intern + spermidine_intern |
| SERP0688-spermidine/putrescine-transport_import | false | 2 ATP-metabolism + 2 H2O + putrescine_extern + spermidine_extern = 2 ADP-metabolism + 2 Pyrophosphate + putrescine_intern + spermidine_intern |
| SERP0765-Uracil-permease-transport_import | false | H+-extern + uracil_extern = H+-intern + uracil_intern |
| SERP0831-PurM_DNA-directed-DNA-polymerase_dATP | false | DNA + dATP = DNA-A + Pyrophosphate |
| SERP0831-PurM_DNA-directed-DNA-polymerase_dCTP | false | DNA + dCTP = DNA-C + Pyrophosphate |
| SERP0831-PurM_DNA-directed-DNA-polymerase_dGTP | false | DNA + dGTP = DNA-G + Pyrophosphate |
| SERP0831-PurM_DNA-directed-DNA-polymerase_dTTP | false | DNA + dTTP = DNA-T + Pyrophosphate |
| SERP0841-PurM_PNPase_ADP | false | ADP-metabolism + RNA = Orthophosphate + RNA-A |
| SERP0841-PurM_PNPase_GDP | false | GDP + RNA = Orthophosphate + RNA-G |
| SERP1403-MultiDrug-transport_efflux | false | ATP-energy + IQ-143 + H2O = ADP-energy + IQ-143-extern + Pyrophosphate |
| SERP1802-cobalt/nickel-transport_efflux | false | 2 ATP-energy + 2 H2O + cobalt-intern + nickel-intern = 2 ADP-energy + 2 Pyrophosphate + cobalt-extern + nickel-extern |
| SERP1803-cobalt/nickel-transport_efflux | false | 2 ATP-energy + 2 H2O + cobalt-intern + nickel-intern = 2 ADP-energy + 2 Pyrophosphate + cobalt-extern + nickel-extern |
| SERP1944-MultiDrug-transport_efflux | false | IQ-143 + H+-intern = IQ-143-extern + H+-extern |
| SERP1951-lipoprotein-transport_efflux/import | true | ATP-energy + H2O + lipoprotein_extern = ADP-energy + Pyrophosphate + lipoprotein_intern |
| SERP1952-macrolide-transport_efflux | false | ATP-energy + H2O + macrolide_intern = ADP-energy + Pyrophosphate + macrolide_extern |
| SERP1997-formate/nitrite-transport_efflux/import | false | H+-intern + formate_internal + nitrite_external = H+-extern + formate_extern + nitrite_intern |
| SERP2060-glyerol-transport_import | false | ATP-energy + H2O + glycerol-3-phosphate_extern = ADP-energy + Pyrophosphate + glycerol-3-phosphate_intern |
| SERP2156-Glyc_L-lactate-dehydrogenase | true | (S)-Lactate + NAD+ = H+ + NADH + Pyruvate |
| SERP2179-choline/betaine/carnitine-transp_efflux | false | H+-extern + betaine_intern + carnitine-extern + choline-extern = H+-intern + betaine-extern + carnitine-intern + choline-intern |
| SERP2186-PurM_ATP_sulfate-adenylyltransferase | false | ATP-energy + Sulfate = Adenylylsulfate + Pyrophosphate |
| SERP2283-phopsphonate-transport_import | false | ATP-energy + H2O + phosphonate_extern = ADP-energy + Pyrophosphate + phosphonate_intern |
| SERP2289-MultiDrug-transport_efflux | false | ATP-energy + IQ-143 + H2O = ADP-energy + IQ-143-extern + Pyrophosphate |
| TCA_citrate-hydro-lyase | true | Citrate = H2O + cis-Aconitate |
| TCA_citrate-hydroxymutase | true | Citrate = Isocitrate |
| TCA_citrate_synthase | true | Citrate + CoA = Acetyl-CoA + H2O + Oxaloacetate |
| TCA_fumarate-hydratase | true | (S)-Malate = Fumarate + H2O |
| TCA_isocitrate-hydro-lyase | true | Isocitrate = H2O + cis-Aconitate |
| TCA_lipoic-transsuccinylase | true | EnzymeN6-(dihydrolipoyl)lysine + Succinyl-CoA = CoA + Dihydrolipoyllysine-residuesuccinyltransferaseS-succinyldihydrolipoyllysine |
| TCA_Oxidoreductase | false | Isocitrate + NAD+ = 2-Oxoglutarate + CO2 + H+ + NADH |
| TCA_oxoglutarate-dehydrogenase-complex1 | true | 2-Oxoglutarate + Thiamindiphosphate = 3-Carboxy-1-hydroxypropyl-ThPP + CO2 |
| TCA_oxoglutarate-dehydrogenase-complex2 | true | 3-Carboxy-1-hydroxypropyl-ThPP + EnzymeN6-(lipoyl)lysine = Dihydrolipoyllysine-residuesuccinyltransferaseS-succinyldihydrolipoyllysine + Thiamindiphosphate |
| TCA_oxoglutarate-synthase | false | CO2 + Reducedferredoxin + Succinyl-CoA = 2-Oxoglutarate + CoA + Oxidizedferredoxin |
| TCA_PEP-carboxylase | true | ATP-energy + Oxaloacetate = ADP-energy + CO2 + Phosphoenolpyruvate |
| TCA_Pyruvate_CO2-ligase | true | ATP-energy + HCO3- + Pyruvate = ADP-energy + Orthophosphate + Oxaloacetate |
| TCA_pyruvate_dehydrogenase | false | 2-(alpha-Hydroxyethyl)thiaminediphosphate + EnzymeN6-(lipoyl)lysine = Dihydrolipoyllysine-residueacetyltransferaseS-acetyldihydrolipoyllysine + Thiamindiphosphate |
| TCA_succinate-dehydrogenase | true | Acceptor + Succinate = Fumarate + Reducedacceptor |

^1^ List of enzymes used to build the metabolic web of *S. epidermidis* RP62A. This list is composed of data derived from KEGG and own annotations.

Abbreviations: AS: Amino acids; FA: fatty acid synthesis and degradation; Glyc: Glycolysis and Pentose Phosphate Pathways; OP: Oxidative phosphorylation; PurM: Purine Metabolism; PyrM: Pyrimidine Metabolism; TCA: Citric acid Cycle; SERP: Enzymes measured by gene expression micro array (see point IV, supplementary materials).

Table S4: List of enzmyes used to build metabolic web for man ^1^.

| Enzyme name | reversible? | Reaction equation |
| --- | --- | --- |
| ADP-energy_to_ADP-metabolism | True | ADP-metabolism = AMP-energy |
| AMP-energy_to_AMP-metabolism | True | AMP-metabolism = AMP-energy |
| ATP-energy_to_ATP-metabolism | True | ATP-metabolism = ATP-energy |
| Cytochrome_1A2 | False | ATP-energy + IQ-143 = ADP-energy + IQ-143_deactivated |
| Cytochrome_2C19 | False | ATP-energy + IQ-143 = ADP-energy + IQ-143_deactivated |
| Cytochrome_2C8 | False | ATP-energy + IQ-143 = ADP-energy + IQ-143_deactivated |
| Cytochrome_2C9 | False | ATP-energy + IQ-143 = ADP-energy + IQ-143_deactivated |
| Cytochrome_2D6 | False | ATP-energy + IQ-143 = ADP-energy + IQ-143_deactivated |
| Cytochrome_3A4 | False | ATP-energy + IQ-143 = ADP-energy + IQ-143_deactivated |
| DNA-extern_To_DNA-intern | True | DNA-extern = DNA |
| EC:1.1.1.1.-rn:R00754 | False | Ethanol + NAD+ = Acetaldehyde + H+ + NADH |
| EC:1.1.1.2-rn:R00746 | False | Ethanol + NADP+ = Acetaldehyde + H+ + NADPH |
| EC:1.1.1.205-rn:R01130 | True | H2O + IMP + NAD+ = H+ + NADH + Xanthosine5-phosphate |
| EC:1.1.1.27-rn:R00703 | True | (S)-Lactate + NAD+ = H+ + NADH + Pyruvate |
| EC:1.1.1.37-rn:R00342 | True | (S)-Malate + NAD+ = H+ + NADH + Oxaloacetate |
| EC:1.1.1.41-rn:R00709 | False | Isocitrate + NAD+ = 2-Oxoglutarate + CO2 + H+ + NADH |
| EC:1.1.1.42-rn:R00268 | True | Oxalosuccinate = 2-Oxoglutarate + CO2 |
| EC:1.1.1.42-rn:R01899 | True | Isocitrate + NADP+ = H+ + NADPH + Oxalosuccinate |
| EC:1.17.1.4-rn:R01768 | False | H2O + Hypoxanthine + NAD+ = NADH + Xanthine |
| EC:1.17.1.4-rn:R02103 | False | H2O + NAD+ + Xanthine = H+ + NADH + Urate |
| EC:1.17.3.2-rn:R01769 | False | H2O + Hypoxanthine + Oxygen = H2O2 + Xanthine |
| EC:1.17.3.2-rn:R02107 | False | H2O + Oxygen + Xanthine = H2O2 + Urate |
| EC:1.17.4.1-rn:R02017 | False | H2O + Oxidizedthioredoxin + dADP = ADP-energy + Thioredoxin |
| EC:1.17.4.1-rn:R02018 | False | H2O + Oxidizedthioredoxin + dUDP = Thioredoxin + UDP |
| EC:1.17.4.1-rn:R02019 | False | H2O + Oxidizedthioredoxin + dGDP = GDP + Thioredoxin |
| EC:1.17.4.1-rn:R02024 | False | H2O + Oxidizedthioredoxin + dCDP = CDP + Thioredoxin |
| EC:1.2.1.12-rn:R01061 | False | (2R)-2-Hydroxy-3-(phosphonooxy)-propanal + NAD+ + Orthophosphate = 3-Phospho-D-glyceroylphosphate + H+ + NADH |
| EC:1.2.1.13-rn:R01063 | False | (2R)-2-Hydroxy-3-(phosphonooxy)-propanal + NADP+ + Orthophosphate = 3-Phospho-D-glyceroylphosphate + H+ + NADPH |
| EC:1.2.1.3-rn:R00710 | True | Acetaldehyde + H2O + NAD+ = Acetate + H+ + NADH |
| EC:1.2.1.3-rn:R00711 | True | Acetaldehyde + H2O + NADP+ = Acetate + H+ + NADPH |
| EC:1.2.4.1-rn:R00014 | False | Pyruvate + Thiamindiphosphate = 2-(alpha-Hydroxyethyl)thiaminediphosphate + CO2 |
| EC:1.2.4.1-rn:R03270 | False | 2-(alpha-Hydroxyethyl)thiaminediphosphate + EnzymeN6-(lipoyl)lysine = Dihydrolipoyllysine-residueacetyltransferaseS-acetyldihydrolipoyllysine + Thiamindiphosphate |
| EC:1.2.4.2-rn:R00621 | True | 2-Oxoglutarate + Thiamindiphosphate = 3-Carboxy-1-hydroxypropyl-ThPP + CO2 |
| EC:1.2.4.2-rn:R03316 | True | 3-Carboxy-1-hydroxypropyl-ThPP + EnzymeN6-(lipoyl)lysine = Dihydrolipoyllysine-residuesuccinyltransferaseS-succinyldihydrolipoyllysine + Thiamindiphosphate |
| EC:1.3.1.2-rn:R00978 | True | 56-Dihydrouracil + NADP+ = H+ + NADPH + Uracil |
| EC:1.3.1.2-rn:R01415 | True | 56-Dihydrothymine + NADP+ = H+ + NADPH + Thymine |
| EC:1.3.3.1-rn:R01867 | True | (S)-Dihydroorotate + Oxygen = H2O2 + Orotate |
| EC:1.3.5.1-rn:R02164 | True | Succinate + Ubiquinone = Fumarate + Ubiquinol |
| EC:1.7.1.7-rn:R01134 | False | IMP + NADP+ + NH3 = GMP + H+ + NADPH |
| EC:1.8.1.4-rn:R07618 | False | EnzymeN6-(dihydrolipoyl)lysine + NAD+ = EnzymeN6-(lipoyl)lysine + H+ + NADH |
| EC:1.8.1.9-rn:R02016 | False | NADP+ + Thioredoxin = H+ + NADPH + Oxidizedthioredoxin |
| EC:2.1.1.45-rn:R02101 | False | 510-Methylenetetrahydrofolate + dUMP = Dihydrofolate + dTMP |
| EC:2.1.3.2-rn:R01397 | False | Carbamoylphosphate + L-Aspartate = N-Carbamoyl-L-aspartate + Orthophosphate |
| EC:2.3.1.12-rn:R02569 | True | Acetyl-CoA + EnzymeN6-(dihydrolipoyl)lysine = CoA + Dihydrolipoyllysine-residueacetyltransferaseS-acetyldihydrolipoyllysine |
| EC:2.3.1.61-rn:R02570 | True | EnzymeN6-(dihydrolipoyl)lysine + Succinyl-CoA = CoA + Dihydrolipoyllysine-residuesuccinyltransferaseS-succinyldihydrolipoyllysine |
| EC:2.3.3.1-rn:R00351 | True | Citrate + CoA = Acetyl-CoA + H2O + Oxaloacetate |
| EC:2.3.3.8-rn:R00352 | True | ATP-energy + Citrate + CoA = ADP-energy + Acetyl-CoA + Orthophosphate + Oxaloacetate |
| EC:2.4.2.1-rn:R01561 | True | Adenosine + Orthophosphate = Adenine + alpha-D-Ribose1-phosphate |
| EC:2.4.2.1-rn:R01863 | True | Inosine + Orthophosphate = Hypoxanthine + alpha-D-Ribose1-phosphate |
| EC:2.4.2.1-rn:R01969 | True | Deoxyguanosine + Orthophosphate = 2-Deoxy-D-ribose1-phosphate + Guanine |
| EC:2.4.2.1-rn:R02147 | True | Guanosine + Orthophosphate = Guanine + alpha-D-Ribose1-phosphate |
| EC:2.4.2.1-rn:R02297 | True | Orthophosphate + Xanthosine = Xanthine + alpha-D-Ribose1-phosphate |
| EC:2.4.2.1-rn:R02484 | True | Deoxyuridine + Orthophosphate = 2-Deoxy-D-ribose1-phosphate + Uracil |
| EC:2.4.2.1-rn:R02557 | True | Deoxyadenosine + Orthophosphate = 2-Deoxy-D-ribose1-phosphate + Adenine |
| EC:2.4.2.1-rn:R02748 | True | Deoxyinosine + Orthophosphate = 2-Deoxy-D-ribose1-phosphate + Hypoxanthine |
| EC:2.4.2.10-rn:R01870 | True | Orotidine5-phosphate + Pyrophosphate = 5-Phospho-alpha-D-ribose1-diphosphate + Orotate |
| EC:2.4.2.3-rn:R01876 | True | Orthophosphate + Uridine = Uracil + alpha-D-Ribose1-phosphate |
| EC:2.4.2.4-rn:R01570 | True | Orthophosphate + Thymidine = 2-Deoxy-D-ribose1-phosphate + Thymine |
| EC:2.4.2.4-rn:R01969 | True | 2-Deoxy-D-ribose1-phosphate + Guanine = Deoxyguanosine + Orthophosphate |
| EC:2.4.2.4-rn:R02484 | True | Deoxyuridine + Orthophosphate = 2-Deoxy-D-ribose1-phosphate + Uracil |
| EC:2.4.2.4-rn:R02557 | True | Deoxyadenosine + Orthophosphate = 2-Deoxy-D-ribose1-phosphate + Adenine |
| EC:2.4.2.4-rn:R02748 | True | Deoxyinosine + Orthophosphate = 2-Deoxy-D-ribose1-phosphate + Hypoxanthine |
| EC:2.4.2.7-rn:R00190 | True | AMP-energy + Pyrophosphate = 5-Phospho-alpha-D-ribose1-diphosphate + Adenine |
| EC:2.4.2.7-rn:R01229 | True | GMP + Pyrophosphate = 5-Phospho-alpha-D-ribose1-diphosphate + Guanine |
| EC:2.4.2.7-rn:R04378 | True | AICAR + Pyrophosphate = 5-Amino-4-imidazolecarboxyamide + 5-Phospho-alpha-D-ribose1-diphosphate |
| EC:2.4.2.8-rn:R01132 | True | IMP + Pyrophosphate = 5-Phospho-alpha-D-ribose1-diphosphate + Hypoxanthine |
| EC:2.4.2.8-rn:R02142 | True | Pyrophosphate + Xanthosine5-phosphate = 5-Phospho-alpha-D-ribose1-diphosphate + Xanthine |
| EC:2.4.2.9-rn:R00966 | True | Pyrophosphate + UMP = 5-Phospho-alpha-D-ribose1-diphosphate + Uracil |
| EC:2.7.1.11-rn:R04779 | True | ATP-energy + beta-D-Fructose6-phosphate = ADP-energy + beta-D-Fructose16-bisphosphate |
| EC:2.7.1.113-rn:R01967 | False | ATP-energy + Deoxyguanosine = ADP-energy + dGMP |
| EC:2.7.1.2-rn:R01600 | True | ATP-energy + beta-D-Glucose = ADP-energy + beta-D-Glucose6-phosphate |
| EC:2.7.1.2-rn:R01786 | True | ATP-energy + alpha-D-Glucose = ADP-energy + alpha-D-Glucose6-phosphate |
| EC:2.7.1.21-rn:R01567 | True | ATP-energy + Thymidine = ADP-energy + dTMP |
| EC:2.7.1.21-rn:R02099 | True | ATP-energy + Deoxyuridine = ADP-energy + dUMP |
| EC:2.7.1.25-rn:R00509 | True | ATP-energy + Adenylylsulfate = 3-Phosphoadenylylsulfate + ADP-energy |
| EC:2.7.1.40-rn:R00200 | False | ATP-energy + Pyruvate = ADP-energy + Phosphoenolpyruvate |
| EC:2.7.1.40-rn:R00430 | False | GTP + Pyruvate = GDP + Phosphoenolpyruvate |
| EC:2.7.1.40-rn:R01138 | False | Pyruvate + dATP = Phosphoenolpyruvate + dADP |
| EC:2.7.1.40-rn:R01858 | False | Pyruvate + dGTP = Phosphoenolpyruvate + dGDP |
| EC:2.7.1.48-rn:R00513 | False | ATP-energy + Cytidine = ADP-energy + CMP |
| EC:2.7.1.48-rn:R00516 | False | Cytidine + UTP = CMP + UDP |
| EC:2.7.1.48-rn:R00517 | False | Cytidine + GTP = CMP + GDP |
| EC:2.7.1.48-rn:R00962 | False | Cytidine + ITP = CMP + IDP |
| EC:2.7.1.48-rn:R00964 | False | ATP-energy + Uridine = ADP-energy + UMP |
| EC:2.7.1.48-rn:R00967 | False | UTP + Uridine = UDP + UMP |
| EC:2.7.1.48-rn:R00968 | False | GTP + Uridine = GDP + UMP |
| EC:2.7.1.48-rn:R00970 | False | ITP + Uridine = IDP + UMP |
| EC:2.7.1.48-rn:R01548 | False | Cytidine + dATP = CMP + dADP |
| EC:2.7.1.48-rn:R01549 | False | Uridine + dATP = UMP + dADP |
| EC:2.7.1.48-rn:R01880 | False | Uridine + dGTP = UMP + dGDP |
| EC:2.7.1.48-rn:R02091 | False | Cytidine + dGTP = CMP + dGDP |
| EC:2.7.1.48-rn:R02096 | False | Cytidine + dTTP = CMP + dTDP |
| EC:2.7.1.48-rn:R02097 | False | Uridine + dTTP = UMP + dTDP |
| EC:2.7.1.48-rn:R02327 | False | Uridine + dCTP = UMP + dCDP |
| EC:2.7.1.48-rn:R02332 | False | Uridine + dUTP = UMP + dUDP |
| EC:2.7.1.48-rn:R02371 | False | Cytidine + dCTP = CMP + dCDP |
| EC:2.7.1.48-rn:R02372 | False | Cytidine + dUTP = CMP + dUDP |
| EC:2.7.1.74-rn:R00185 | False | ATP-energy + Adenosine = ADP-energy + AMP-metabolism |
| EC:2.7.1.74-rn:R01666 | False | ATP-energy + Deoxycytidine = ADP-energy + dCMP |
| EC:2.7.2.3-rn:R01512 | True | 3-Phospho-D-glycerate + ATP-energy = 3-Phospho-D-glyceroylphosphate + ADP-energy |
| EC:2.7.4.10-rn:R00157 | True | AMP-energy + UTP = ADP-energy + UDP |
| EC:2.7.4.14-rn:R00158 | True | ATP-energy + UMP = ADP-energy + UDP |
| EC:2.7.4.14-rn:R00512 | True | ATP-energy + CMP = ADP-energy + CDP |
| EC:2.7.4.14-rn:R01665 | True | ATP-energy + dCMP = ADP-energy + dCDP |
| EC:2.7.4.3-rn:R00127 | True | AMP-metabolism + ATP-energy = 2 ADP-metabolism |
| EC:2.7.4.3-rn:R01547 | True | ATP-energy + dAMP = ADP-energy + dADP |
| EC:2.7.4.4-rn:R00334 | True | ATP-energy + UMP = ADP-energy + UDP |
| EC:2.7.4.4-rn:R02098 | True | ATP-energy + dUMP = ADP-energy + dUDP |
| EC:2.7.4.6-rn:R00124 | True | ADP-metabolism + ATP-energy = ADP-energy + ATP-metabolism |
| EC:2.7.4.6-rn:R00156 | True | ATP-energy + UDP = ADP-energy + UTP |
| EC:2.7.4.6-rn:R00330 | True | ATP-energy + GDP = ADP-energy + GTP |
| EC:2.7.4.6-rn:R00570 | True | ATP-energy + CDP = ADP-energy + CTP |
| EC:2.7.4.6-rn:R00722 | True | ATP-energy + IDP = ADP-energy + ITP |
| EC:2.7.4.6-rn:R01137 | True | ATP-energy + dADP = ADP-energy + dATP |
| EC:2.7.4.6-rn:R01857 | True | ATP-energy + dGDP = ADP-energy + dGTP |
| EC:2.7.4.6-rn:R02093 | True | ATP-energy + dTDP = ADP-energy + dTTP |
| EC:2.7.4.6-rn:R02326 | True | ATP-energy + dCDP = ADP-energy + dCTP |
| EC:2.7.4.6-rn:R02331 | True | ATP-energy + dUDP = ADP-energy + dUTP |
| EC:2.7.4.6-rn:R03530 | True | ATP-energy + dIDP = ADP-energy + dITP |
| EC:2.7.4.8-rn:R00332 | True | ATP-energy + GMP = ADP-energy + GDP |
| EC:2.7.4.8-rn:R02090 | True | ATP-energy + dGMP = ADP-energy + dGDP |
| EC:2.7.4.9-rn:R02094 | True | ATP-energy + dTMP = ADP-energy + dTDP |
| EC:2.7.4.9-rn:R02098 | True | ATP-energy + dUMP = ADP-energy + dUDP |
| EC:2.7.6.1-rn:R01049 | True | ATP-energy + D-Ribose5-phosphate = 5-Phospho-alpha-D-ribose1-diphosphate + AMP-energy |
| EC:2.7.7.6-rn:R00435 | True | ATP-metabolism + RNA = Pyrophosphate + RNA-A |
| EC:2.7.7.6-rn:R00441 | True | GTP + RNA = Pyrophosphate + RNA-G |
| EC:2.7.7.6-rn:R00442 | True | CTP + RNA = Pyrophosphate + RNA-C |
| EC:2.7.7.6-rn:R00443 | True | RNA + UTP = Pyrophosphate + RNA-U |
| EC:3.1.3.11-rn:R04780 | False | H2O + beta-D-Fructose16-bisphosphate = Orthophosphate + beta-D-Fructose6-phosphate |
| EC:3.1.3.5-rn:R00183 | False | AMP-energy + H2O = Adenosine + Orthophosphate |
| EC:3.1.3.5-rn:R00511 | False | CMP + H2O = Cytidine + Orthophosphate |
| EC:3.1.3.5-rn:R00963 | False | H2O + UMP = Orthophosphate + Uridine |
| EC:3.1.3.5-rn:R01126 | False | H2O + IMP = Inosine + Orthophosphate |
| EC:3.1.3.5-rn:R01227 | False | GMP + H2O = Guanosine + Orthophosphate |
| EC:3.1.3.5-rn:R01569 | False | H2O + dTMP = Orthophosphate + Thymidine |
| EC:3.1.3.5-rn:R01664 | False | H2O + dCMP = Deoxycytidine + Orthophosphate |
| EC:3.1.3.5-rn:R01968 | False | H2O + dGMP = Deoxyguanosine + Orthophosphate |
| EC:3.1.3.5-rn:R02088 | False | H2O + dAMP = Deoxyadenosine + Orthophosphate |
| EC:3.1.3.5-rn:R02719 | False | H2O + Xanthosine5-phosphate = Orthophosphate + Xanthosine |
| EC:3.1.3.9-rn:R01788 | False | H2O + alpha-D-Glucose6-phosphate = Orthophosphate + alpha-D-Glucose |
| EC:3.1.4.17-rn:R00191 | False | 35-CyclicAMP + H2O = AMP-energy |
| EC:3.1.4.17-rn:R01234 | False | 35-CyclicGMP + H2O = GMP |
| EC:3.5.1.6-rn:R00905 | False | 3-Ureidopropionate + H2O = CO2 + NH3 + beta-Alanine |
| EC:3.5.1.6-rn:R04666 | False | 3-Ureidoisobutyrate + H2O = 3-Aminoisobutanoate + CO2 + NH3 |
| EC:3.5.2.2-rn:R02269 | True | 56-Dihydrouracil + H2O = 3-Ureidopropionate |
| EC:3.5.2.2-rn:R03055 | True | 56-Dihydrothymine + H2O = 3-Ureidoisobutyrate |
| EC:3.5.2.3-rn:R01993 | True | (S)-Dihydroorotate + H2O = N-Carbamoyl-L-aspartate |
| EC:3.5.2.5-rn:R02425 | False | Allantoate + H2O = Allantoine |
| EC:3.5.3.4-rn:R02422 | False | Allantoate + H2O = (-)-Ureidoglycolate + Urea |
| EC:3.5.4.10-rn:R01127 | True | H2O + IMP = FAICAR |
| EC:3.5.4.12-rn:R01663 | True | H2O + dCMP = NH3 + dUMP |
| EC:3.5.4.3-rn:R01676 | False | Guanine + H2O = NH3 + Xanthine |
| EC:3.5.4.4-rn:R01560 | False | Adenosine + H2O = Inosine + NH3 |
| EC:3.5.4.4-rn:R02556 | False | Deoxyadenosine + H2O = Deoxyinosine + NH3 |
| EC:3.5.4.5-rn:R01878 | True | Cytidine + H2O = NH3 + Uridine |
| EC:3.5.4.5-rn:R02485 | True | Deoxycytidine + H2O = Deoxyuridine + NH3 |
| EC:3.5.4.6-rn:R00181 | False | AMP-energy + H2O = IMP + NH3 |
| EC:3.6.1.11-rn:R03409 | True | Guanosine3-diphosphate5-triphosphate + H2O = Guanosine35-bis(diphosphate) + Orthophosphate |
| EC:3.6.1.13-rn:R01054 | False | ADP-ribose + H2O = AMP-energy + D-Ribose5-phosphate |
| EC:3.6.1.17-rn:R00184 | True | H2O + P1P4-Bis(5-adenosyl)tetraphosphate = AMP-energy + ATP-energy |
| EC:3.6.1.17-rn:R00969 | True | H2O + P1P4-Bis(5-uridyl)tetraphosphate = UMP + UTP |
| EC:3.6.1.17-rn:R01232 | True | H2O + P1P4-Bis(5-guanosyl)tetraphosphate = GMP + GTP |
| EC:3.6.1.17-rn:R02805 | True | H2O + P1P4-Bis(5-xanthosyl)tetraphosphate = XTP + Xanthosine5-phosphate |
| EC:3.6.1.19-rn:R00426 | False | GTP + H2O = GMP + Pyrophosphate |
| EC:3.6.1.19-rn:R00662 | False | H2O + UTP = Pyrophosphate + UMP |
| EC:3.6.1.19-rn:R00720 | False | H2O + ITP = IMP + Pyrophosphate |
| EC:3.6.1.19-rn:R01855 | False | H2O + dGTP = Pyrophosphate + dGMP |
| EC:3.6.1.19-rn:R02100 | False | H2O + dUTP = Pyrophosphate + dUMP |
| EC:3.6.1.19-rn:R02720 | False | H2O + XTP = Pyrophosphate + Xanthosine5-phosphate |
| EC:3.6.1.19-rn:R03531 | False | H2O + dITP = 2-Deoxyinosine5-phosphate + Pyrophosphate |
| EC:3.6.1.23-RN:R02100 | False | H2O + dUTP = Pyrophosphate + dUMP |
| EC:3.6.1.29-rn:R00187 | True | H2O + P1P3-Bis(5-adenosyl)triphosphate = ADP-energy + AMP-energy |
| EC:3.6.1.3-rn:R00086 | False | ATP-metabolism + H2O = ADP-metabolism + Orthophosphate |
| EC:3.6.1.41-rn:R00125 | False | H2O + P1P4-Bis(5-adenosyl)tetraphosphate = 2 ADP-energy |
| EC:3.6.1.5-rn:R00086 | False | ATP-energy + Ca2+IN + H2O = ADP-energy + Orthophosphate |
| EC:3.6.1.5-rn:R00122 | False | ADP-energy + Ca2+IN + H2O = AMP-energy + Orthophosphate |
| EC:3.6.1.5-rn:R00155 | False | Ca2+IN + H2O + UDP = Orthophosphate + UMP |
| EC:3.6.1.5-rn:R00159 | False | Ca2+IN + H2O + UTP = Orthophosphate + UDP |
| EC:3.6.1.5-rn:R00328 | False | Ca2+IN + GDP + H2O = GMP + Orthophosphate |
| EC:3.6.1.5-rn:R00335 | False | Ca2+IN + GTP + H2O = GDP + Orthophosphate |
| EC:3.6.1.5-rn:R00514 | False | CDP + Ca2+IN + H2O = CMP + Orthophosphate |
| EC:3.6.1.5-rn:R00569 | False | CTP + Ca2+IN + H2O = CDP + Orthophosphate |
| EC:3.6.1.5-rn:R00719 | False | Ca2+IN + H2O + ITP = IDP + Orthophosphate |
| EC:3.6.1.5-rn:R00961 | False | Ca2+IN + H2O + IDP = IMP + Orthophosphate |
| EC:3.6.1.5-rn:R02092 | False | Ca2+IN + H2O + dTDP = Orthophosphate + dTMP |
| EC:3.6.1.5-rn:R02095 | False | Ca2+IN + H2O + dTTP = Orthophosphate + dTDP |
| EC:3.6.1.6-rn:R00155 | False | Ca2+IN + H2O + UDP = Orthophosphate + UMP |
| EC:3.6.1.6-rn:R00328 | False | Ca2+IN + GDP + H2O = GMP + Orthophosphate |
| EC:3.6.1.6-rn:R00961 | False | Ca2+IN + H2O + IDP = IMP + Orthophosphate |
| EC:3.6.1.7-rn:R01515 | False | 3-Phospho-D-glyceroylphosphate + H2O = 3-Phospho-D-glycerate + Orthophosphate |
| EC:3.6.1.8-rn:R00086 | False | ATP-metabolism + Ca2+IN + H2O = ADP-energy + Orthophosphate |
| EC:3.6.1.8-rn:R00087 | False | ATP-metabolism + Ca2+IN + H2O = AMP-energy + Orthophosphate |
| EC:3.6.1.8-rn:R00426 | False | Ca2+IN + GTP-extern + H2O = GMP + Pyrophosphate |
| EC:3.6.1.8-rn:R00720 | False | Ca2+IN + H2O + ITP-extern = IMP + Pyrophosphate |
| EC:3.6.1.9-rn:R00507 | False | 3-Phosphoadenylylsulfate + H2O = Adenosine35-bisphosphate + Sulfate |
| EC:4.1.1.21-rn:R04209 | True | 1-(5-Phospho-D-ribosyl)-5-amino-4-imidazolecarboxylate = Aminoimidazoleribotide + CO2 |
| EC:4.1.1.23-rn:R00965 | True | Orotidine5-phosphate = CO2 + UMP |
| EC:4.1.1.32-rn:R00431 | True | GTP + Oxaloacetate = CO2 + GDP + Phosphoenolpyruvate |
| EC:4.1.1.32-rn:R00726 | True | ITP + Oxaloacetate = CO2 + IDP + Phosphoenolpyruvate |
| EC:4.1.2.13-rn:R01070 | True | beta-D-Fructose16-bisphosphate = (2R)-2-Hydroxy-3-(phosphonooxy)-propanal + Glyceronephosphate |
| EC:4.1.3.6-rn:R00362 | False | Citrate = Acetate + Oxaloacetate |
| EC:4.2.1.11-rn:R00658 | True | 2-Phospho-D-glycerate = H2O + Phosphoenolpyruvate |
| EC:4.2.1.2-rn:R01082 | True | (S)-Malate = Fumarate + H2O |
| EC:4.2.1.3-rn:R01324 | True | Citrate = Isocitrate |
| EC:4.2.1.3-rn:R01325 | True | Citrate = H2O + cis-Aconitate |
| EC:4.2.1.3-rn:R01900 | True | Isocitrate = H2O + cis-Aconitate |
| EC:4.2.1.70-rn:R01055 | True | D-Ribose5-phosphate + Uracil = H2O + Pseudouridine5-phosphate |
| EC:4.3.2.2-rn:R01083 | True | N6-(12-Dicarboxyethyl)-AMP = AMP-energy + Fumarate |
| EC:4.3.2.2-rn:R04559 | True | 1-(5-Phosphoribosyl)-5-amino-4-(N-succinocarboxamide)-imidazole = AICAR + Fumarate |
| EC:4.6.1.1-rn:R00089 | False | ATP-energy = 35-CyclicAMP + Pyrophosphate |
| EC:4.6.1.1-rn:R00434 | False | GTP = 35-CyclicGMP + Pyrophosphate |
| EC:4.6.1.2-rn:R00434 | False | GTP = 35-CyclicGMP + Pyrophosphate |
| EC:5.1.3.3-rn:R01602 | True | alpha-D-Glucose = beta-D-Glucose |
| EC:5.3.1.1-rn:R01015 | True | (2R)-2-Hydroxy-3-(phosphonooxy)-propanal = Glyceronephosphate |
| EC:5.3.1.9-rn:R02739 | True | alpha-D-Glucose6-phosphate = beta-D-Glucose6-phosphate |
| EC:5.3.1.9-rn:R02740 | True | alpha-D-Glucose6-phosphate = beta-D-Fructose6-phosphate |
| EC:5.3.1.9-rn:R03321 | True | beta-D-Glucose6-phosphate = beta-D-Fructose6-phosphate |
| EC:5.4.2.1-rn:R01518 | True | 2-Phospho-D-glycerate = 3-Phospho-D-glycerate |
| EC:5.4.2.1-rn:R01662 | False | 3-Phospho-D-glyceroylphosphate = 23-Bisphospho-D-glycerate |
| EC:5.4.2.2-rn:R00959 | True | D-Glucose1-phosphate = alpha-D-Glucose6-phosphate |
| EC:5.4.2.4-rn:R01516 | False | 23-Bisphospho-D-glycerate + H2O = 3-Phospho-D-glycerate + Orthophosphate |
| EC:6.2.1.1-rn:R00235 | True | ATP-energy + Acetate + CoA = AMP-energy + Acetyl-CoA + Pyrophosphate |
| EC:6.2.1.4-rn:R00432 | True | CoA + GTP + Succinate = GDP + Orthophosphate + Succinyl-CoA |
| EC:6.2.1.4-rn:R00727 | True | CoA + ITP + Succinate = IDP + Orthophosphate + Succinyl-CoA |
| EC:6.2.1.5-rn:R00405 | True | ATP-energy + CoA + Succinate = ADP-energy + Orthophosphate + Succinyl-CoA |
| EC:6.3.2.6-rn:R04591 | true | 1-(5-Phospho-D-ribosyl)-5-amino-4-imidazolecarboxylate + ATP-metabolism + L-Aspartate = 1-(5-Phosphoribosyl)-5-amino-4-(N-succinocarboxamide)-imidazole + ADP-metabolism + Orthophosphate |
| EC:6.3.4.1-rn:R01230 | False | ATP-energy + NH3 + Xanthosine5-phosphate = AMP-energy + GMP + Pyrophosphate |
| EC:6.3.4.2-rn:R00571 | False | ATP-energy + NH3 + UTP = ADP-energy + CTP + Orthophosphate |
| EC:6.3.4.2-rn:R00573 | False | ATP-energy + H2O + L-Glutamine + UTP = ADP-energy + CTP + L-Glutamate + Orthophosphate |
| EC:6.3.4.4-rn:R01135 | True | GTP + IMP + L-Aspartate = GDP + N6-(12-Dicarboxyethyl)-AMP + Orthophosphate |
| EC:6.3.5.2-rn:R01231 | False | ATP-energy + H2O + L-Glutamine + Xanthosine5-phosphate = AMP-energy + GMP + L-Glutamate + Pyrophosphate |
| EC:6.3.5.5-rn:R00575 | False | 2 ATP-energy + H2O + HCO3- + L-Glutamine = 2 ADP-energy + Carbamoylphosphate + L-Glutamate + Orthophosphate |
| EC:6.4.1.1-rn:R00344 | True | ATP-energy + HCO3- + Pyruvate = ADP-energy + Orthophosphate + Oxaloacetate |
| SERP0653-EC:6.3.5.3-rn:R04463 | False | 5-Phosphoribosyl-N-formylglycinamide + ATP-energy + H2O + L-Glutamine = 2-(Formamido)-N1-(5-phosphoribosyl)acetamidine + ADP-energy + L-Glutamate + Orthophosphate |
| SERP0653-EC:6.3.5.3-rn:R04463_blocked | False | 5-Phosphoribosyl-N-formylglycinamide + ATP-energy + IQ-143 + H2O + L-Glutamine = 2-(Formamido)-N1-(5-phosphoribosyl)acetamidine_blo + ADP-energy + IQ-143_used + L-Glutamate + Orthophosphate |
| SERP0655-EC:2.4.2.14-rn:R01072 | False | 5-Phosphoribosylamine + L-Glutamate + Pyrophosphate = 5-Phospho-alpha-D-ribose1-diphosphate + H2O + L-Glutamine |
| SERP0655-EC:2.4.2.14-rn:R01072_blocked | False | 5-Phosphoribosylamine + IQ-143 + L-Glutamate + Pyrophosphate = 5-Phospho-alpha-D-ribose1-diphosphate_blocked + IQ-143_used + H2O + L-Glutamine |
| SERP0656-EC:6.3.3.1-rn:R04208 | False | 2-(Formamido)-N1-(5-phosphoribosyl)acetamidine + ATP-energy = ADP-energy + Aminoimidazoleribotide + Orthophosphate |
| SERP0656-EC:6.3.3.1-rn:R04208_blocked | False | 2-(Formamido)-N1-(5-phosphoribosyl)acetamidine + ATP-energy + IQ-143 = 5-Phosphoribosyl-N-formylglycinamide_blocked + IQ-143_used + Tetrahydrofolate |
| SERP0657-EC:2.1.2.2-rn:R04325 | False | 10-Formyltetrahydrofolate + 5-Phosphoribosylglycinamide = 5-Phosphoribosyl-N-formylglycinamide + Tetrahydrofolate |
| SERP0657-EC:2.1.2.2-rn:R04325_blocked | False | 10-Formyltetrahydrofolate + 5-Phosphoribosylglycinamide + IQ-143 = 5-Phosphoribosyl-N-formylglycinamide_blocked + IQ-143_used + Tetrahydrofolate |
| SERP0658-EC:2.1.2.3-rn:R04560 | False | 10-Formyltetrahydrofolate + AICAR = FAICAR + Tetrahydrofolate |
| SERP0658-EC:2.1.2.3-rn:R04560_blocked | false | 10-Formyltetrahydrofolate + AICAR + IQ-143 = FAICAR-blocked + IQ-143_used + Tetrahydrofolate |
| SERP0659-EC:6.3.4.13-rn:R04144 | False | 5-Phosphoribosylamine + ATP-energy + Glycine = 5-Phosphoribosylglycinamide + ADP-energy + Orthophosphate |
| SERP0659-EC:6.3.4.13-rn:R04144_blocked | False | 5-Phosphoribosylamine + ATP-energy + IQ-143 + Glycine = 5-Phosphoribosylglycinamide + ADP-energy + IQ-143_used + Orthophosphate |
| SERP0831-EC:2.7.7.7-rn:R00375 | True | DNA + dATP = DNA-A + Pyrophosphate |
| SERP0831-EC:2.7.7.7-rn:R00375_blocked | False | DNA + IQ-143+ dATP = DNA-A_blocked + IQ-_used + Pyrophosphate |
| SERP0831-EC:2.7.7.7-rn:R00376 | True | DNA + dGTP = DNA-G + Pyrophosphate |
| SERP0831-EC:2.7.7.7-rn:R00376_blocked | False | DNA + IQ-143+ dGTP = DNA-G_blocked + IQ-_used + Pyrophosphate |
| SERP0831-EC:2.7.7.7-rn:R00377 | True | DNA + dCTP = DNA-C + Pyrophosphate |
| SERP0831-EC:2.7.7.7-rn:R00377_blocked | False | DNA + IQ-143+ dCTP = DNA-C_blocked + IQ-_used + Pyrophosphate |
| SERP0831-EC:2.7.7.7-rn:R00378 | True | DNA + dTTP = DNA-T + Pyrophosphate |
| SERP0831-EC:2.7.7.7-rn:R00378_blocked | False | DNA + IQ-143+ dTTP = DNA-T_blocked + IQ-_used + Pyrophosphate |
| SERP0841-EC:2.7.7.8-rn:R00437 | False | ADP-metabolism + RNA = Orthophosphate + RNA-A |
| SERP0841-EC:2.7.7.8-rn:R00437_blocked | False | ADP-metabolism + IQ-143+ RNA = IQ-_used + Orthophosphate + RNA-A_blocked |
| SERP0841-EC:2.7.7.8-rn:R00438 | False | RNA + UDP = Orthophosphate + RNA-U |
| SERP0841-EC:2.7.7.8-rn:R00438_blocked | False | IQ-143+ RNA + UDP = IQ-_used + Orthophosphate + RNA-U_blocked |
| SERP0841-EC:2.7.7.8-rn:R00439 | False | GDP + RNA = Orthophosphate + RNA-G |
| SERP0841-EC:2.7.7.8-rn:R00439_blocked | False | IQ-143+ GDP + RNA = IQ-_used + Orthophosphate + RNA-G_blocked |
| SERP0841-EC:2.7.7.8-rn:R00440 | False | CDP + RNA = Orthophosphate + RNA-C |
| SERP0841-EC:2.7.7.8-rn:R00440_blocked | False | CDP + IQ-143+ RNA = IQ-_used + Orthophosphate + RNA-C_blocked |
| SERP2186-EC:2.7.7.4-rn:R00529 | False | ATP-energy + Sulfate = Adenylylsulfate + Pyrophosphate |
| SERP2186-EC:2.7.7.4-rn:R00529_blocked | False | ATP-energy + IQ-143+ Sulfate = Adenylylsulfate_blocked + IQ-_used + Pyrophosphate |

^1^ List of enzymes used to build the metabolic web of *H. sapiens*. This list is composed of data derived from KEGG and own annotations.

Abbreviations: SERP: Enzymes measured by gene expression micro array (see point IV, supplementary materials).

**III: Synthesis and effects of the isoquinoline IQ-143.**

Figure S1: Synthesis of IQ-143 according to Bringmann et al 2007 [49]:


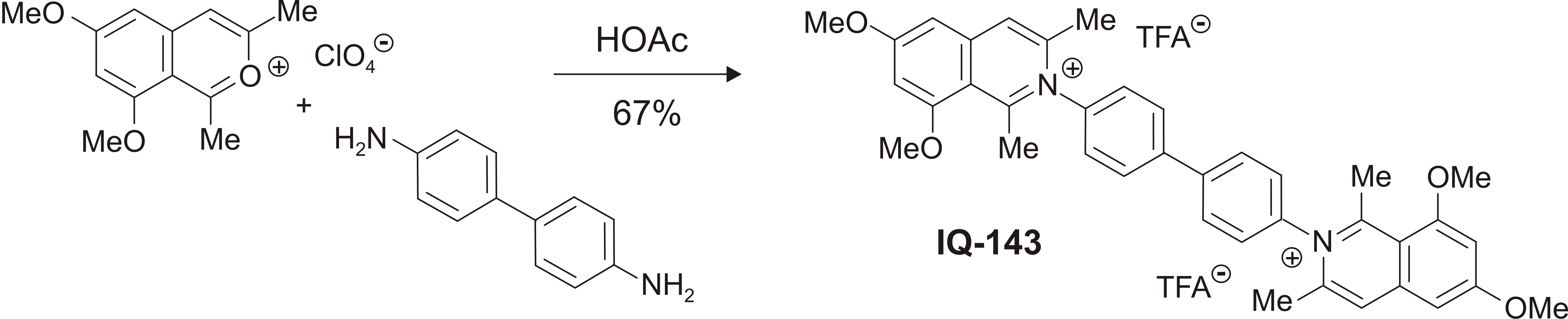


The compound IQ-143 was synthesized according to the method above by T. Gulder [49].

Figure S2: Effects of IQ-143 on *S. epidermidis* RP62A (Bringmann et al. 2007 [49])


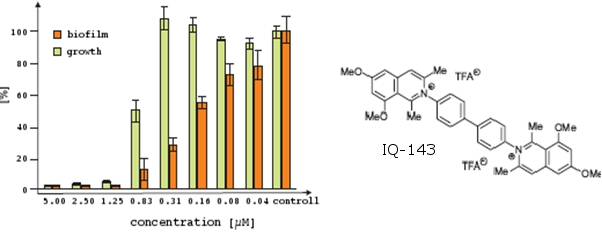


^1^ Growth inhibition of different concentrations of IQ-143[49].

Figure S3: Effects of IQ143 on human Cytochrome- P- enzymes^1^.


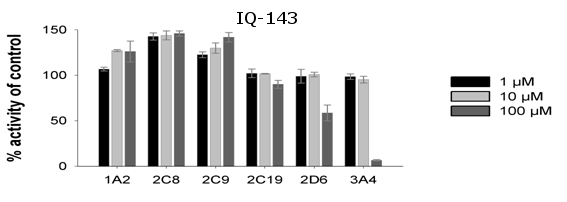


^1^ Percentage of cytochrome activation is shown on the y-axis, the six key cytochromes in man were incubated in vitro with different concentrations of IQ-143 (see materials and methods). Only one human cytochrome (3A4) is inhibited at high concentrations, two other (2C19 and 2D6) are reduced.

**IV) Gene expression data gathered by the Ohlsen group.**

Table S5: This data shows the effects of 1.25µM IQ-143 on *S. epidermidis* RP62A as measured by the Ohlsen group.

|  | **Concentration: 1.25µM** |  |  |  |  |  |  |  |  |  |
| --- | --- | --- | --- | --- | --- | --- | --- | --- | --- | --- |
| **id** | **Name** | **Mean (Stats)** | **Median (Stats)** | **SD (Stats)** | **CV (Stats)** | **Count (Stats)** | **Min (Stats)** | **Max (Stats)** | **Max-Min (Stats)** | **One Sample t-Test (Adv)** |
| SERP0630 | 2-succinyl-6-hydroxy-2,4-cyclohexadiene-1-carboxylic acid synthase-2-oxoglutarate decarboxylase (menD) [4.1.1.71] {Staphylococcus epidermidis RP62A} | **2.224** | 2.210 | **0.026** | 1.155 | **6** | 2.092 | 2.475 | 0.383 | **0.000000** |
| SERP0372 | 6-pyruvoyl tetrahydrobiopterin synthase, putative {Staphylococcus epidermidis RP62A} | **4.007** | 3.599 | **0.126** | 3.157 | **6** | 3.104 | 6.170 | 3.067 | **0.000081** |
| SERP1803 | ABC transporter, ATP-binding protein {Staphylococcus epidermidis RP62A} | **2.276** | 2.301 | **0.023** | 1.004 | **6** | 2.132 | 2.407 | 0.275 | **0.000000** |
| SERP1802 | ABC transporter, ATP-binding protein {Staphylococcus epidermidis RP62A} | **2.449** | 2.401 | **0.143** | 5.827 | **6** | 1.655 | 4.008 | 2.352 | **0.001138** |
| SERP1403 | ABC transporter, permease-ATP-binding protein {Staphylococcus epidermidis RP62A} | **2.050** | 2.063 | **0.044** | 2.145 | **6** | 1.758 | 2.307 | 0.549 | **0.000012** |
| SERP2311 | acetyltransferase, GNAT family {Staphylococcus epidermidis RP62A} | **2.739** | 2.676 | **0.072** | 2.638 | **6** | 2.089 | 3.366 | 1.276 | **0.000025** |
| SERP1996 | acetyltransferase, GNAT family {Staphylococcus epidermidis RP62A} | **3.323** | 3.258 | **0.075** | 2.261 | **6** | 2.669 | 4.270 | 1.601 | **0.000013** |
| SERP0389 | alcohol dehydrogenase, iron-containing [1.1.1.1] {Staphylococcus epidermidis RP62A} | **3.358** | 3.070 | **0.081** | 2.418 | **5** | 2.803 | 4.153 | 1.349 | **0.000132** |
| SERP2112 | alcohol dehydrogenase, zinc-containing [1.1.1.1] {Staphylococcus epidermidis RP62A} | **4.080** | 4.015 | **0.038** | 0.934 | **3** | 3.771 | 4.485 | 0.714 | **0.001296** |
| SERP0963 | aspartokinase, alpha and beta subunits (lysC) [2.7.2.4] {Staphylococcus epidermidis RP62A} | **3.391** | 4.010 | **0.187** | 5.527 | **5** | 1.726 | 5.326 | 3.599 | **0.003192** |
| SERP2244 | capA-related protein {Staphylococcus epidermidis RP62A} | **2.716** | 2.786 | **0.056** | 2.052 | **6** | 2.175 | 3.060 | 0.885 | **0.000007** |
| SERP2179 | choline-carnitine-betaine transporter {Staphylococcus epidermidis RP62A} | **2.225** | 2.389 | **0.133** | 5.995 | **6** | 1.364 | 3.077 | 1.714 | **0.001402** |
| SERP1144 | conserved hypothetical protein {Staphylococcus epidermidis RP62A} | **2.019** | 1.955 | **0.046** | 2.256 | **6** | 1.796 | 2.372 | 0.576 | **0.000015** |
| SERP1215 | conserved hypothetical protein {Staphylococcus epidermidis RP62A} | **2.131** | 2.031 | **0.067** | 3.147 | **6** | 1.851 | 2.872 | 1.021 | **0.000071** |
| SERP1405 | conserved hypothetical protein {Staphylococcus epidermidis RP62A} | **2.168** | 1.966 | **0.126** | 5.791 | **6** | 1.676 | 3.721 | 2.045 | **0.001237** |
| SERP1998 | conserved hypothetical protein {Staphylococcus epidermidis RP62A} | **2.001** | 1.980 | **0.048** | 2.385 | **5** | 1.765 | 2.341 | 0.576 | **0.000146** |
| SERP2079 | conserved hypothetical protein {Staphylococcus epidermidis RP62A} | **2.146** | 2.163 | **0.016** | 0.765 | **4** | 2.040 | 2.222 | 0.182 | **0.000033** |
| SERP0916 | conserved hypothetical protein {Staphylococcus epidermidis RP62A} | **2.047** | 2.046 | **0.032** | 1.540 | **6** | 1.874 | 2.211 | 0.337 | **0.000002** |
| SERP0241 | conserved hypothetical protein {Staphylococcus epidermidis RP62A} | **2.198** | 2.161 | **0.057** | 2.580 | **6** | 1.927 | 2.658 | 0.731 | **0.000026** |
| SERP2527 | conserved hypothetical protein {Staphylococcus epidermidis RP62A} | **2.248** | 2.206 | **0.075** | 3.358 | **5** | 1.919 | 2.951 | 1.032 | **0.000479** |
| SERP2068 | conserved hypothetical protein {Staphylococcus epidermidis RP62A} | **2.305** | 2.365 | **0.109** | 4.735 | **5** | 1.662 | 3.329 | 1.667 | **0.001751** |
| SERP2178 | conserved hypothetical protein {Staphylococcus epidermidis RP62A} | **2.837** | 3.109 | **0.134** | 4.721 | **6** | 1.848 | 3.853 | 2.005 | **0.000419** |
| SERP0591 | conserved hypothetical protein {Staphylococcus epidermidis RP62A} | **2.731** | 2.772 | **0.165** | 6.029 | **5** | 1.513 | 4.288 | 2.774 | **0.004065** |
| SERP1053 | conserved hypothetical protein {Staphylococcus epidermidis RP62A} | **2.491** | 2.209 | **0.123** | 4.947 | **6** | 1.857 | 3.731 | 1.874 | **0.000530** |
| SERP2110 | conserved hypothetical protein {Staphylococcus epidermidis RP62A} | **2.800** | 2.845 | **0.047** | 1.670 | **6** | 2.468 | 3.195 | 0.727 | **0.000003** |
| SERP0182 | conserved hypothetical protein {Staphylococcus epidermidis RP62A} | **2.920** | 3.009 | **0.051** | 1.744 | **6** | 2.468 | 3.282 | 0.814 | **0.000003** |
| SERP0467 | conserved hypothetical protein {Staphylococcus epidermidis RP62A} | **3.526** | 3.286 | **0.061** | 1.723 | **5** | 3.059 | 4.180 | 1.122 | **0.000036** |
| SERP1015 | conserved hypothetical protein {Staphylococcus epidermidis RP62A} | **4.525** | 4.557 | **0.022** | 0.489 | **6** | 4.208 | 4.821 | 0.613 | **0.000000** |
| SERP1145 | conserved hypothetical protein TIGR00046 {Staphylococcus epidermidis RP62A} | **2.131** | 2.097 | **0.041** | 1.903 | **6** | 1.897 | 2.485 | 0.588 | **0.000006** |
| SERP0672 | cytochrome bd ubiquinol oxidase, subunit I (cydA) [1.10.3.-] {Staphylococcus epidermidis RP62A} | **12.078** | 12.680 | **0.126** | 1.043 | **6** | 8.463 | 16.268 | 7.804 | **0.000004** |
| SERP0673 | cytochrome bd ubiquinol oxidase, subunit II (cydB) [1.10.3.-] {Staphylococcus epidermidis RP62A} | **8.390** | 8.002 | **0.104** | 1.241 | **6** | 6.402 | 12.612 | 6.210 | **0.000004** |
| SERP0831 | DNA polymerase III, alpha subunit, Gram-positive type [2.7.7.7] {Staphylococcus epidermidis RP62A} | **2.156** | 2.202 | **0.051** | 2.343 | **6** | 1.787 | 2.392 | 0.605 | **0.000016** |
| SERP1433 | DNA-damage-inducible protein P (dinP) {Staphylococcus epidermidis RP62A} | **2.806** | 2.470 | **0.134** | 4.760 | **6** | 2.073 | 4.190 | 2.117 | **0.000434** |
| SERP1944 | drug resistance transporter, EmrB-QacA family {Staphylococcus epidermidis RP62A} | **2.212** | 2.075 | **0.086** | 3.888 | **6** | 1.857 | 3.106 | 1.250 | **0.000187** |
| SERP0263 | endonuclease III, putative {Staphylococcus epidermidis RP62A} | **3.752** | 3.678 | **0.090** | 2.406 | **4** | 3.089 | 4.743 | 1.655 | **0.001048** |
| SERP0729 | excinuclease ABC, C subunit (uvrC) {Staphylococcus epidermidis RP62A} | **2.994** | 2.973 | **0.053** | 1.781 | **6** | 2.639 | 3.644 | 1.005 | **0.000004** |
| SERP0373 | exsB protein {Staphylococcus epidermidis RP62A} | **4.199** | 3.926 | **0.098** | 2.327 | **6** | 3.286 | 5.554 | 2.268 | **0.000020** |
| SERP0371 | exsD protein {Staphylococcus epidermidis RP62A} | **3.878** | 3.623 | **0.092** | 2.360 | **6** | 3.118 | 5.312 | 2.194 | **0.000019** |
| SERP2366 | formate acetyltransferase (pflB) [2.3.1.54] {Staphylococcus epidermidis RP62A} | **4.184** | 4.481 | **0.167** | 3.994 | **4** | 2.586 | 5.904 | 3.318 | **0.005026** |
| SERP1997 | formate-nitrite transporter family protein {Staphylococcus epidermidis RP62A} | **2.553** | 2.619 | **0.109** | 4.263 | **6** | 1.648 | 3.556 | 1.909 | **0.000260** |
| SERP0413 | GGDEF domain protein {Staphylococcus epidermidis RP62A} | **2.015** | 2.008 | **0.075** | 3.743 | **6** | 1.531 | 2.563 | 1.032 | **0.000181** |
| SERP1760 | glucosamine--fructose-6-phosphate aminotransferase, isomerizing (glmS) [2.6.1.16] {Staphylococcus epidermidis RP62A} | **2.710** | 2.666 | **0.030** | 1.091 | **6** | 2.555 | 3.084 | 0.529 | **0.000000** |
| SERP2060 | glycerol-3-phosphate transporter (glpT) {Staphylococcus epidermidis RP62A} | **2.700** | 2.823 | **0.055** | 2.049 | **5** | 2.340 | 3.148 | 0.809 | **0.000064** |
| SERP0363 | glycosyl transferase, group 2 family protein {Staphylococcus epidermidis RP62A} | **2.484** | 2.734 | **0.110** | 4.444 | **6** | 1.732 | 3.224 | 1.491 | **0.000320** |
| SERP1208 | GTP-binding protein, GTP1-OBG family {Staphylococcus epidermidis RP62A} | **2.165** | 2.053 | **0.069** | 3.177 | **6** | 1.857 | 2.694 | 0.837 | **0.000072** |
| SERP1696 | HD domain protein {Staphylococcus epidermidis RP62A} | **3.237** | 3.657 | **0.196** | 6.044 | **5** | 1.505 | 4.604 | 3.099 | **0.004312** |
| SERP1206 | Holliday junction DNA helicase RuvA (ruvA) {Staphylococcus epidermidis RP62A} | **2.071** | 1.999 | **0.067** | 3.219 | **6** | 1.716 | 2.726 | 1.009 | **0.000083** |
| SERP0350 | hypothetical protein {Staphylococcus epidermidis RP62A} | **2.444** | 2.615 | **0.103** | 4.226 | **4** | 1.754 | 2.976 | 1.222 | **0.004882** |
| SERP2321 | immunodominant antigen B, putative {Staphylococcus epidermidis RP62A} | **2.030** | 1.957 | **0.064** | 3.159 | **6** | 1.750 | 2.525 | 0.775 | **0.000079** |
| SERP0629 | isochorismate synthase family protein {Staphylococcus epidermidis RP62A} | **2.266** | 2.222 | **0.043** | 1.882 | **6** | 2.017 | 2.662 | 0.646 | **0.000005** |
| SERP1796 | lactose phosphotransferase system repressor (lacR) {Staphylococcus epidermidis RP62A} | **6.112** | 5.855 | **0.186** | 3.045 | **6** | 4.076 | 13.083 | 9.007 | **0.000145** |
| SERP0043 | lysozyme domain protein {Staphylococcus epidermidis RP62A} | **2.715** | 2.909 | **0.095** | 3.502 | **5** | 2.030 | 3.437 | 1.408 | **0.000520** |
| SERP1230 | membrane protein, putative {Staphylococcus epidermidis RP62A} | **2.628** | 2.768 | **0.122** | 4.653 | **6** | 1.735 | 3.656 | 1.921 | **0.000391** |
| SERP2520 | methicillin-resistance regulatory protein MecR1 (mecR1) {Staphylococcus epidermidis RP62A} | **2.547** | 2.449 | **0.144** | 5.669 | **6** | 1.792 | 3.745 | 1.953 | **0.000988** |
| SERP0084 | NADH dehydrogenase I, F subunit (nuoF) [1.6.5.3] {Staphylococcus epidermidis RP62A} | **2.264** | 2.307 | **0.082** | 3.643 | **6** | 1.659 | 2.939 | 1.280 | **0.000133** |
| SERP1980 | nitrite extrusion protein {Staphylococcus epidermidis RP62A} | **2.001** | 1.867 | **0.101** | 5.069 | **6** | 1.583 | 2.802 | 1.218 | **0.000768** |
| SERP0119 | Orn-Lys-Arg decarboxylase {Staphylococcus epidermidis RP62A} | **2.127** | 2.002 | **0.129** | 6.051 | **6** | 1.596 | 3.221 | 1.625 | **0.001550** |
| SERP1151 | oxygen-independent coproporphyrinogen III oxidase, putative {Staphylococcus epidermidis RP62A} | **2.177** | 2.215 | **0.055** | 2.526 | **6** | 1.774 | 2.521 | 0.747 | **0.000023** |
| SERP2064 | PAP2 family protein {Staphylococcus epidermidis RP62A} | **3.337** | 3.422 | **0.106** | 3.174 | **4** | 2.448 | 4.328 | 1.880 | **0.002203** |
| SERP0828 | phosphatidate cytidylyltransferase (cdsA) [2.7.7.41] {Staphylococcus epidermidis RP62A} | **2.400** | 2.377 | **0.096** | 4.020 | **6** | 1.697 | 3.170 | 1.472 | **0.000202** |
| SERP2283 | phosphonate ABC transporter, permease protein {Staphylococcus epidermidis RP62A} | **2.654** | 2.680 | **0.048** | 1.802 | **3** | 2.367 | 2.948 | 0.581 | **0.004214** |
| SERP1721 | phosphotyrosine protein phosphatase [3.1.3.48] {Staphylococcus epidermidis RP62A} | **2.917** | 2.959 | **0.021** | 0.720 | **6** | 2.704 | 3.090 | 0.387 | **0.000000** |
| SERP0841 | polyribonucleotide nucleotidyltransferase (pnp) [2.7.7.8] {Staphylococcus epidermidis RP62A} | **2.698** | 2.867 | **0.052** | 1.936 | **6** | 2.214 | 3.007 | 0.792 | **0.000005** |
| SERP0448 | preprotein translocase, SecG subunit (secG) {Staphylococcus epidermidis RP62A} | **2.288** | 2.146 | **0.122** | 5.353 | **6** | 1.851 | 3.921 | 2.070 | **0.000811** |
| SERP1324 | proline dehydrogenase (putA) [1.5.99.8] {Staphylococcus epidermidis RP62A} | **3.816** | 3.585 | **0.063** | 1.642 | **3** | 3.445 | 4.501 | 1.056 | **0.003845** |
| SERP1909 | PTS system, IIBC components {Staphylococcus epidermidis RP62A} | **4.222** | 3.952 | **0.073** | 1.726 | **3** | 3.727 | 5.109 | 1.382 | **0.004493** |
| SERP0002 | ribonuclease P protein component (rnpA) [3.1.26.5] {Staphylococcus epidermidis RP62A} | **3.710** | 3.617 | **0.088** | 2.385 | **6** | 2.929 | 5.049 | 2.120 | **0.000019** |
| SERP0179 | ribosomal protein L1 (rplA) {Staphylococcus epidermidis RP62A} | **2.036** | 2.177 | **0.066** | 3.263 | **6** | 1.590 | 2.374 | 0.784 | **0.000091** |
| SERP0840 | ribosomal protein S15 (rpsO) {Staphylococcus epidermidis RP62A} | **2.022** | 2.054 | **0.038** | 1.901 | **6** | 1.713 | 2.231 | 0.518 | **0.000007** |
| SERP0826 | ribosome recycling factor (frr) {Staphylococcus epidermidis RP62A} | **2.333** | 2.373 | **0.026** | 1.128 | **6** | 2.084 | 2.479 | 0.395 | **0.000000** |
| SERP1435 | RNA methyltransferase, TrmA family {Staphylococcus epidermidis RP62A} | **2.011** | 1.964 | **0.034** | 1.670 | **6** | 1.840 | 2.243 | 0.403 | **0.000003** |
| SERP2401 | serine protease {Staphylococcus epidermidis RP62A} | **4.396** | 4.388 | **0.055** | 1.256 | **6** | 3.783 | 5.175 | 1.392 | **0.000001** |
| SERP2201 | sodium:solute symporter family protein {Staphylococcus epidermidis RP62A} | **2.415** | 2.369 | **0.069** | 2.859 | **6** | 1.970 | 3.109 | 1.139 | **0.000039** |
| SERP0118 | spermidine N1-acetyltransferase, putative {Staphylococcus epidermidis RP62A} | **2.133** | 2.376 | **0.165** | 7.712 | **6** | 1.342 | 3.297 | 1.955 | **0.004476** |
| SERP0686 | spermidine-putrescine ABC transporter, ATP-binding protein (potA) {Staphylococcus epidermidis RP62A} | **2.401** | 2.361 | **0.098** | 4.070 | **6** | 1.883 | 3.363 | 1.481 | **0.000215** |
| SERP0687 | spermidine-putrescine ABC transporter, permease protein PotB (potB) {Staphylococcus epidermidis RP62A} | **2.181** | 2.208 | **0.040** | 1.844 | **6** | 1.905 | 2.401 | 0.497 | **0.000005** |
| SERP0688 | spermidine-putrescine ABC transporter, permease protein PotC (potC) {Staphylococcus epidermidis RP62A} | **2.103** | 2.075 | **0.053** | 2.497 | **6** | 1.853 | 2.441 | 0.589 | **0.000023** |
| SERP1722 | Sua5-YciO-YrdC-YwlC family protein {Staphylococcus epidermidis RP62A} | **2.841** | 2.762 | **0.071** | 2.494 | **6** | 2.382 | 3.539 | 1.157 | **0.000019** |
| SERP1673 | threonine dehydratase (ilvA) [4.2.1.16] {Staphylococcus epidermidis RP62A} | **4.317** | 4.223 | **0.121** | 2.799 | **6** | 3.211 | 6.798 | 3.587 | **0.000050** |
| SERP0110 | transcriptional regulator, LysR family {Staphylococcus epidermidis RP62A} | **2.646** | 2.526 | **0.158** | 5.980 | **6** | 1.744 | 4.892 | 3.148 | **0.001250** |
| SERP0264 | transcriptional regulator, MerR family {Staphylococcus epidermidis RP62A} | **3.488** | 3.412 | **0.092** | 2.624 | **6** | 2.836 | 5.172 | 2.336 | **0.000028** |
| SERPA0009 | transcriptional regulator, putative {Staphylococcus epidermidis RP62A} | **2.177** | 2.237 | **0.084** | 3.839 | **5** | 1.664 | 2.616 | 0.952 | **0.000830** |
| SERP1797 | transcriptional regulator, Sir2 family {Staphylococcus epidermidis RP62A} | **15.612** | 16.124 | **0.047** | 0.300 | **6** | 13.009 | 17.749 | 4.740 | **0.000000** |
| SERP2289 | transporter, putative {Staphylococcus epidermidis RP62A} | **2.131** | 1.971 | **0.102** | 4.782 | **6** | 1.682 | 2.985 | 1.303 | **0.000523** |
| SERP2240 | transposase, IS200 family {Staphylococcus epidermidis RP62A} | **3.218** | 3.381 | **0.114** | 3.541 | **6** | 2.241 | 4.693 | 2.452 | **0.000112** |
| SERP2245 | tributyrin esterase EstA, putative {Staphylococcus epidermidis RP62A} | **2.307** | 2.531 | **0.096** | 4.156 | **6** | 1.550 | 2.774 | 1.224 | **0.000245** |
| SERP0806 | tRNA (guanine-N1)-methyltransferase (trmD) [2.1.1.31] {Staphylococcus epidermidis RP62A} | **2.973** | 2.973 | **0.019** | 0.627 | **6** | 2.809 | 3.174 | 0.365 | **0.000000** |
| SERP0765 | uracil permease (uraA) {Staphylococcus epidermidis RP62A} | **3.120** | 2.763 | **0.224** | 7.164 | **6** | 1.901 | 6.819 | 4.918 | **0.002906** |
| SERP0825 | uridylate kinase (pyrH) [2.7.4.-] {Staphylococcus epidermidis RP62A} | **2.225** | 2.255 | **0.039** | 1.753 | **6** | 1.926 | 2.429 | 0.504 | **0.000004** |
| SERP2547 | YjeF-related protein {Staphylococcus epidermidis RP62A} | **2.074** | 2.081 | **0.057** | 2.732 | **6** | 1.744 | 2.522 | 0.778 | **0.000037** |
| SERP0292 | ABC transporter, ATP-binding protein (sitA) {Staphylococcus epidermidis RP62A} | **0.452** | 0.430 | **0.057** | 12.654 | **6** | 0.404 | 0.531 | 0.127 | **0.000026** |
| SERP1951 | ABC transporter, ATP-binding protein {Staphylococcus epidermidis RP62A} | **0.469** | 0.457 | **0.032** | 6.918 | **6** | 0.437 | 0.536 | 0.099 | **0.000002** |
| SERP0291 | ABC transporter, permease protein (sitB) {Staphylococcus epidermidis RP62A} | **0.436** | 0.450 | **0.052** | 12.029 | **6** | 0.355 | 0.504 | 0.150 | **0.000013** |
| SERP1952 | ABC transporter, permease protein {Staphylococcus epidermidis RP62A} | **0.395** | 0.386 | **0.068** | 17.286 | **6** | 0.319 | 0.502 | 0.183 | **0.000029** |
| SERP0290 | ABC transporter, substrate-binding protein (sitC) {Staphylococcus epidermidis RP62A} | **0.454** | 0.449 | **0.034** | 7.582 | **6** | 0.407 | 0.519 | 0.112 | **0.000002** |
| SERP2192 | phosophoadenylyl-sulfate reductase (cysH) [1.8.4.8] {Staphylococcus epidermidis RP62A} | **0.415** | 0.428 | **0.046** | 11.132 | **6** | 0.351 | 0.460 | 0.109 | **0.000005** |
| SERP0659 | phosphoribosylamine--glycine ligase (purD) [6.3.4.13] {Staphylococcus epidermidis RP62A} | **0.389** | 0.392 | **0.086** | 22.178 | **6** | 0.296 | 0.532 | 0.236 | **0.000082** |
| SERP0658 | phosphoribosylaminoimidazolecarboxamide formyltransferase-IMP cyclohydrolase (purH) {Staphylococcus epidermidis RP62A} | **0.429** | 0.439 | **0.086** | 20.004 | **6** | 0.317 | 0.545 | 0.229 | **0.000136** |
| SERP0656 | phosphoribosylformylglycinamidine cyclo-ligase (purM) [6.3.3.1] {Staphylococcus epidermidis RP62A} | **0.427** | 0.424 | **0.077** | 18.090 | **6** | 0.342 | 0.519 | 0.176 | **0.000079** |
| SERP0653 | phosphoribosylformylglycinamidine synthase I (purQ) [6.3.5.3] {Staphylococcus epidermidis RP62A} | **0.483** | 0.491 | **0.101** | 20.965 | **6** | 0.370 | 0.646 | 0.275 | **0.000612** |
| SERP0654 | phosphoribosylformylglycinamidine synthase II (purL) [6.3.5.3] {Staphylococcus epidermidis RP62A} | **0.456** | 0.441 | **0.080** | 17.525 | **6** | 0.381 | 0.593 | 0.213 | **0.000138** |
| SERP0652 | phosphoribosylformylglycinamidine synthase, PurS protein (purS) {Staphylococcus epidermidis RP62A} | **0.481** | 0.486 | **0.116** | 24.197 | **6** | 0.331 | 0.727 | 0.396 | **0.001134** |
| SERP0657 | phosphoribosylglycinamide formyltransferase (purN) [2.1.2.2] {Staphylococcus epidermidis RP62A} | **0.427** | 0.426 | **0.090** | 21.199 | **6** | 0.338 | 0.534 | 0.196 | **0.000170** |
| SERP0655 | amidophosphoribosyltransferase (purF) [2.4.2.14] {Staphylococcus epidermidis RP62A} | **0.432** | 0.436 | **0.089** | 20.572 | **6** | 0.340 | 0.544 | 0.204 | **0.000168** |
| SERP1285 | glycerophosphoryl diester phosphodiesterase, putative {Staphylococcus epidermidis RP62A} | **0.493** | 0.501 | **0.044** | 8.898 | **6** | 0.435 | 0.555 | 0.120 | **0.000012** |
| SERP0256 | conserved domain protein {Staphylococcus epidermidis RP62A} | **0.473** | 0.452 | **0.111** | 23.487 | **6** | 0.370 | 0.730 | 0.361 | **0.000818** |
| SERP2187 | conserved hypothetical protein {Staphylococcus epidermidis RP62A} | **0.366** | 0.378 | **0.060** | 16.537 | **6** | 0.286 | 0.416 | 0.130 | **0.000011** |
| SERP0173 | conserved hypothetical protein {Staphylococcus epidermidis RP62A} | **0.422** | 0.415 | **0.069** | 16.328 | **6** | 0.341 | 0.509 | 0.168 | **0.000043** |
| SERP0171 | conserved hypothetical protein {Staphylococcus epidermidis RP62A} | **0.435** | 0.422 | **0.089** | 20.486 | **6** | 0.338 | 0.546 | 0.208 | **0.000176** |
| SERP0330 | conserved hypothetical protein {Staphylococcus epidermidis RP62A} | **0.470** | 0.499 | **0.098** | 20.781 | **6** | 0.341 | 0.609 | 0.268 | **0.000429** |
| SERP2186 | sulfate adenylyltransferase (sat) [2.7.7.4] {Staphylococcus epidermidis RP62A} | **0.343** | 0.349 | **0.043** | 12.442 | **6** | 0.284 | 0.377 | 0.093 | **0.000001** |
| SERP2191 | sulfite reductase (NADPH) flavoprotein alpha-component (cysJ) [1.8.1.2] {Staphylococcus epidermidis RP62A} | **0.440** | 0.443 | **0.026** | 5.950 | **6** | 0.398 | 0.467 | 0.069 | **0.000000** |
| SERP2190 | sulfite reductase (NADPH) hemoprotein beta-component (cysI) [1.8.1.2] {Staphylococcus epidermidis RP62A} | **0.375** | 0.378 | **0.024** | 6.363 | **6** | 0.339 | 0.394 | 0.055 | **0.000000** |
| SERP0172 | RNA methyltransferase, TrmH family, group 3 {Staphylococcus epidermidis RP62A} | **0.451** | 0.430 | **0.091** | 20.152 | **6** | 0.374 | 0.661 | 0.287 | **0.000242** |
| SERP0174 | RNA polymerase sigma factor sigW, putative {Staphylococcus epidermidis RP62A} | **0.423** | 0.403 | **0.083** | 19.562 | **6** | 0.353 | 0.600 | 0.247 | **0.000105** |
| SERP0357 | transcriptional regulator, DeoR family {Staphylococcus epidermidis RP62A} | **0.360** | 0.374 | **0.081** | 22.581 | **6** | 0.284 | 0.455 | 0.172 | **0.000042** |
| SERP0170 | cysteinyl-tRNA synthetase (cysS) [6.1.1.16] {Staphylococcus epidermidis RP62A} | **0.454** | 0.436 | **0.084** | 18.468 | **6** | 0.362 | 0.616 | 0.254 | **0.000169** |
| SERP1195 | D-tyrosyl-tRNA(Tyr) deacylase (dtd) [3.1.-.-] {Staphylococcus epidermidis RP62A} | **0.342** | 0.335 | **0.043** | 12.551 | **6** | 0.301 | 0.391 | 0.089 | **0.000001** |
| SERP0169 | serine acetyltransferase (cysE) [2.3.1.30] {Staphylococcus epidermidis RP62A} | **0.443** | 0.420 | **0.067** | 15.201 | **6** | 0.390 | 0.588 | 0.199 | **0.000051** |
| SERP2188 | siroheme synthase, putative {Staphylococcus epidermidis RP62A} | **0.347** | 0.359 | **0.046** | 13.172 | **6** | 0.287 | 0.388 | 0.101 | **0.000002** |
| SERP2189 | uroporphyrin-III C-methyltransferase, putative {Staphylococcus epidermidis RP62A} | **0.378** | 0.374 | **0.053** | 14.127 | **6** | 0.311 | 0.447 | 0.136 | **0.000007** |
| SERP1258 | citrate synthase (gltA) [2.3.3.1] {Staphylococcus epidermidis RP62A} | **0.472** | 0.475 | **0.048** | 10.257 | **6** | 0.397 | 0.537 | 0.140 | **0.000015** |
| SERP2156 | L-lactate dehydrogenase (ldh) [1.1.1.27] {Staphylococcus epidermidis RP62A} | **0.486** | 0.478 | **0.049** | 10.144 | **6** | 0.429 | 0.570 | 0.141 | **0.000020** |
| SERP0358 | 1-phosphofructokinase (fruK) [2.7.1.56] {Staphylococcus epidermidis RP62A} | **0.351** | 0.360 | **0.085** | 24.130 | **6** | 0.253 | 0.443 | 0.190 | **0.000046** |

Table S6: This data shows the effects of 0.16µM IQ-143 on *S. epidermidis* RP62A as measured by the Ohlsen group.

|  | Concentration: 0.16µM |  |  |  |  |  |  |  |  |  |
| --- | --- | --- | --- | --- | --- | --- | --- | --- | --- | --- |
|  | Name | Mean (Stats) | Median (Stats) | SD (Stats) | CV (Stats) | Count (Stats) | Min (Stats) | Max (Stats) | Max-Min (Stats) | One Sample t-Test (Adv) |
| SERP2178 | conserved hypothetical protein {Staphylococcus epidermidis RP62A} | 4.464 | 4.406 | 0.071 | 1.599 | 12 | 3.383 | 5.570 | 2.187 | 0.000000 |
| SERP2179 | choline-carnitine-betaine transporter {Staphylococcus epidermidis RP62A} | 8.477 | 7.071 | 0.149 | 1.762 | 12 | 5.884 | 17.771 | 11.888 | 0.000000 |
| SERP2176 | choline dehydrogenase (betA) [1.1.99.1] {Staphylococcus epidermidis RP62A} | 22.286 | 29.051 | 0.322 | 1.444 | 12 | 3.685 | 53.000 | 49.315 | 0.000000 |
| SERP2177 | betaine aldehyde dehydrogenase (betB) [1.2.1.8] {Staphylococcus epidermidis RP62A} | 38.110 | 36.852 | 0.088 | 0.231 | 12 | 27.884 | 55.324 | 27.441 | 0.000000 |
| SERP2294 | intercellular adhesion protein D (icaD) {Staphylococcus epidermidis RP62A} | 0.340 | 0.316 | 0.177 | 52.277 | 7 | 0.221 | 0.667 | 0.445 | 0.000426 |
| SERP2293 | intercellular adhesion protein A (icaA) {Staphylococcus epidermidis RP62A} | 0.239 | 0.237 | 0.288 | 120.615 | 6 | 0.086 | 0.555 | 0.469 | 0.003229 |
| SERP1792 | tagatose 1 | 0.462 | 0.511 | 0.116 | 25.142 | 11 | 0.293 | 0.632 | 0.339 | 0.000002 |
| SERP1794 | galactose-6-phosphate isomerase | 0.426 | 0.399 | 0.158 | 37.077 | 10 | 0.260 | 0.711 | 0.451 | 0.000040 |
| SERP1793 | tagatose-6-phosphate kinase (lacC) [2.7.1.144] {Staphylococcus epidermidis RP62A} | 0.494 | 0.479 | 0.114 | 23.172 | 6 | 0.367 | 0.686 | 0.319 | 0.001230 |
| SERP1795 | galactose-6-phosphate isomerase | 0.464 | 0.478 | 0.170 | 36.552 | 5 | 0.277 | 0.758 | 0.481 | 0.011668 |
| SERP1791 | PTS system | 0.485 | 0.500 | 0.111 | 22.828 | 9 | 0.320 | 0.644 | 0.324 | 0.000027 |
| SERP0572 | oligopeptide ABC transporter | 0.493 | 0.609 | 0.219 | 44.522 | 11 | 0.148 | 0.788 | 0.640 | 0.000920 |
| SERP0571 | oligopeptide ABC transporter | 0.490 | 0.579 | 0.209 | 42.644 | 10 | 0.270 | 1.064 | 0.794 | 0.001134 |
| SERP0570 | oligopeptide ABC transporter | 0.449 | 0.418 | 0.282 | 62.788 | 9 | 0.197 | 1.126 | 0.929 | 0.005989 |
| SERP0290 | ABC transporter | 0.407 | 0.399 | 0.292 | 71.665 | 8 | 0.181 | 1.147 | 0.967 | 0.006904 |
| SERP0292 | ABC transporter | 0.488 | 0.543 | 0.288 | 58.952 | 10 | 0.221 | 1.389 | 1.168 | 0.007665 |
| SERP0291 | ABC transporter | 0.437 | 0.544 | 0.253 | 57.919 | 9 | 0.202 | 1.080 | 0.878 | 0.002769 |
| SERP1790 | PTS system | 0.669 | 0.655 | 0.054 | 8.091 | 9 | 0.510 | 0.789 | 0.278 | 0.000011 |

**V) Extreme modes calculated by our YANAsquare models:**

Table S7: Extreme Modes of *S. aureus* USA300 without IQ-143^1^:

| # | Activity | Flux sum | Reversible? | Pathlength | Reactions |
| --- | --- | --- | --- | --- | --- |
| 1 | 1,00 | 1 | true | 1 | (1 AS_Alanine_to_Pyruvate) |
| 2 | 1,00 | 1 | true | 1 | (1 PyrM_dCMP-aminohydrolase) |
| 3 | -0,65 | 1 | true | 1 | (1 PyrM_GMP-pyrophosphorylase) |
| 4 | 1,00 | 1 | true | 1 | (1 PurM_ATP_IDP-phosphotransferase) |
| 5 | 1,00 | 1 | true | 1 | (1 PyrM_cytidilate-kinase_dCMP) |
| 6 | 1,00 | 1 | true | 1 | (1 PyrM_dUMP-phosphotransferase) |
| 7 | 1,00 | 1 | true | 1 | (1 PurM_ATP_GMP-guanylate-kinase) |
| 8 | 1,00 | 1 | true | 1 | (1 PyrM_cytidine-aminohydrolase) |
| 9 | 1,00 | 1 | true | 1 | (1 PurM_ATP_UTP-phosphotransferase) |
| 10 | 1,00 | 1 | true | 1 | (1 PurM_ATP_GTP-phosphotransferase) |
| 11 | 1,00 | 2 | true | 2 | (-1 PurM_nucleotide-phosphatase_Deoxyguanosine) (1 PyrM_deoxyguanosine-phosphorylase) |
| 12 | 1,00 | 2 | true | 2 | (-1 PyrM_dihydroorotase) (1 PyrM_dihydroorotate-oxidase) |
| 13 | 1,00 | 1 | true | 1 | (1 PurM_ATP_dIDP-phosphotransferase) |
| 14 | 1,00 | 1 | true | 1 | (1 PyrM_thymidine-kinase_dTMP) |
| 15 | 1,00 | 1 | true | 1 | (1 PurM_xanthosine-phosphoribosyltransferase) |
| 16 | 1,00 | 1 | true | 1 | (1 PurM_ATP_dUDP-phosphotransferase) |
| 17 | 0,96 | 1 | true | 1 | (1 TCA_fumarate-hydratase) |
| 18 | 1,00 | 3 | true | 3 | (1 PurM_IMP-pyrophosphorylase) (-1 PurM_nucleotide-phosphatase_Inosine) (1 PyrM_uridine-phosphorylase) |
| 19 | 1,00 | 3 | true | 3 | (1 PurM_IMP-pyrophosphorylase) (1 PurM_nucleotide-phosphatase_Guanosine) (-1 PurM_nucleotide-phosphatase_Inosine) |
| 20 | 1,00 | 3 | true | 3 | (1 PurM_IMP-pyrophosphorylase) (-1 PurM_nucleotide-phosphatase_Inosine) (1 PurM_nucleotide-phosphatase_Xanthosine) |
| 21 | 1,00 | 3 | true | 3 | (1 PurM_IMP-pyrophosphorylase) (-1 PurM_nucleotide-phosphatase_Deoxyinosine) (1 PyrM_deoxyinosine-phosphorylase) |
| 22 | 1,00 | 1 | true | 1 | (1 AS_Serine_to_Glycine) |
| 23 | 1,00 | 1 | true | 1 | (1 SERP1951-lipoprotein-transport_efflux/import) |
| 24 | 1,00 | 1 | true | 1 | (1 PurM_GMP-pyrophosphorylase2) |
| 25 | 1,00 | 1 | true | 1 | (1 PyrM_nucleoside-phosphate-kinase_ATP) |
| 26 | 1,00 | 1 | true | 1 | (1 PyrM_ATP_dTDP_thymidylate-kinase) |
| 27 | 1,00 | 1 | true | 1 | (1 PurM_ATP_dTDP-phosphotransferase) |
| 28 | 1,00 | 1 | true | 1 | (1 PurM_ATP_GMP_guanylate-kinase) |
| 29 | 1,00 | 1 | true | 1 | (1 PyrM_nucleoside-phosphate-kinase_ATP2) |
| 30 | -1,33 | 2 | true | 2 | (-1 PurM_nucleotide-phosphatase_Deoxyuridine) (1 PyrM_deoxyuridine-phosphorylase) |
| 31 | 1,00 | 2 | true | 2 | (1 PyrM_deoxyuridine-phosphorylase) (-1 PyrM_thymidine-kinase_dUMP) |
| 32 | 1,00 | 1 | true | 1 | (1 PurM_ATP_dGDP-phosphotransferase) |
| 33 | 1,00 | 1 | true | 1 | (1 AS_Aspartate_to_Homoserine) |
| 34 | 1,00 | 1 | true | 1 | (1 PurM_ATP_dADP-phosphotransferase) |
| 35 | 0,96 | 2 | true | 2 | (1 TCA_citrate-hydro-lyase) (-1 TCA_citrate-hydroxymutase) |
| 36 | 1,00 | 3 | true | 3 | (1 Glyc_lipoic_acetyltransferase) (-1 TCA_citrate-hydro-lyase) (1 TCA_citrate_synthase) |
| 37 | 1,00 | 7 | true | 6 | (-1 Glyc_acetaldehyde-dehydrogenase_NAD+) (-1 Glyc_Actetate-CoA-ligase) (-1 SERP0389-Glyc_Ethanol_NAD+-oxidoreductase) (2 SERP2156-Glyc_L-lactate-dehydrogenase) (-1 TCA_citrate-hydro-lyase) (1 TCA_citrate_synthase) |
| 38 | 1,00 | 1 | true | 1 | (1 PurM_ATP_CDP-phosphotransferase) |
| 39 | 1,00 | 1 | true | 1 | (1 AS_Saccharopine_to_Lysine) |
| 40 | 1,00 | 1 | true | 1 | (1 PurM_IMP_L-aspartate-ligase) |
| 41 | 1,00 | 1 | true | 1 | (1 Glyc_Succinate-CoA-ligase) |
| 42 | 1,00 | 3 | true | 3 | (1 AS_Acetyl-CoA_to_L-Valine) (-1 TCA_citrate-hydro-lyase) (1 TCA_citrate_synthase) |
| 43 | 1,00 | 1 | true | 1 | (1 PyrM_orotate-phosphoribosyltransferase) |
| 44 | 1,00 | 1 | true | 1 | (1 PurM_metaphosphatase) |
| 45 | 1,00 | 1 | true | 1 | (1 PyrM_cytidilate-kinase_CTP) |
| 46 | 1,00 | 1 | true | 1 | (1 Glyc_PTS-permease2) |
| 47 | 1,00 | 1 | true | 1 | (1 PyrM_thymidine-phosphorylase) |
| 48 | 1,00 | 1 | true | 1 | (1 PurM_ATP_dCDP-phosphotransferase) |
| 49 | -0,87 | 1 | true | 1 | (1 PyrM_UMP-pyrophosphorylase) |
| 50 | -0,33 | 1 | true | 1 | (1 PyrM_nucleoside-triphosphate-adenylate-kinase) |
| 51 | -0,33 | 1 | true | 1 | (1 PurM_XMP-pyrophosphorylase) |
| 52 | -0,33 | 2 | true | 2 | (1 PyrM_Deoxycytidine-aminohydrolase) (-1 PyrM_Deoxycytidine-deaminase) |
| 53 | -0,33 | 2 | true | 2 | (-1 PurM_nucleotide-phosphatase_Deoxyadenosine) (1 PyrM_deoxyadenosine-phosphorylase) |
| 54 | -0,33 | 1 | true | 1 | (1 PyrM_ATP_dUDP_thymidylate-kinase) |
| 55 | 0,73 | 1 | true | 1 | (1 PurM_adenylate-kinase_dAMP) |
| 56 | 0,72 | 2 | true | 2 | (1 AMP-energy_to_AMP-metabolism) (1 PurM_AMP-pyrophosphorylase2) |
| 57 | 1,28 | 2 | true | 2 | (1 AMP-energy_to_AMP-metabolism) (1 PyrM_AMP-pyrophosphorylase) |
| 58 | -0,50 | 10 | true | 6 | (1 AMP-energy_to_AMP-metabolism) (2 Glyc_alpha-D-Glucose-6-phosphate-ketol-isomerase) (2 Glyc_ATP-alpha-D-glucokinase) (-2 Glyc_ATP-beta-D-glucokinase) (-2 Glyc_D-Glucose-1-epimerase) (1 PurM_adenylate-kinase_AMP) |
| 59 | 0,37 | 6 | true | 4 | (-1 AMP-energy_to_AMP-metabolism) (2 ATP-energy_to_ATP-metabolism) (-1 PurM_adenylate-kinase_AMP) (-2 PurM_nucleoside-diphosphate-phosphotransferase_ATP) |
| 60 | 0,38 | 2 | true | 2 | (-1 AMP-energy_to_AMP-metabolism) (1 PurM_adenylosuccinate-lyase) |
| 61 | 0,72 | 12 | true | 7 | (-1 AMP-energy_to_AMP-metabolism) (-2 Glyc_alpha-D-Glucose-6-phosphate-ketol-isomerase2) (-2 Glyc_ATP-alpha-D-glucokinase) (2 Glyc_ATP-beta-D-glucokinase) (2 Glyc_beta-D-Glucose-6-phosphate-ketol-isomerase) (2 Glyc_D-Glucose-1-epimerase) (-1 PurM_adenylate-kinase_AMP) |
| 62 | 0,51 | 12 | true | 7 | (-1 AMP-energy_to_AMP-metabolism) (-2 Glyc_6-phospho-beta-glucosidase) (-2 Glyc_ATP-alpha-D-glucokinase) (2 Glyc_ATP-beta-D-glucokinase) (2 Glyc_D-Glucose-1-epimerase) (2 Glyc_PTS-permease1) (-1 PurM_adenylate-kinase_AMP) |
| 63 | 1,00 | 1 | true | 1 | (1 PurM_adenylylsulfate-kinase) |
| 64 | 1,00 | 4 | true | 3 | (2 ADP-energy_to_ADP-metabolism) (-1 AMP-energy_to_AMP-metabolism) (-1 PurM_adenylate-kinase_AMP) |
| 65 | 1,00 | 3 | true | 3 | (1 PurM_IMP-pyrophosphorylase) (1 PurM_nucleotide-phosphatase_Adenine) (-1 PurM_nucleotide-phosphatase_Inosine) |
| 66 | 1,00 | 3 | true | 3 | (1 PurM_IMP-pyrophosphorylase) (-1 PurM_nucleotide-phosphatase_Inosine) (1 PyrM_pyrimidine-nucleoside-phosphorylase) |
| 67 | 1,00 | 1 | true | 1 | (1 TCA_isocitrate-hydro-lyase) |
| 68 | 1,00 | 2 | false | 2 | (1 PurM_ATP-phosphohydrolase) (1 PurM_nucleoside-diphosphate-phosphotransferase_ATP) |
| 69 | 1,00 | 2 | false | 2 | (1 AMP-energy_to_AMP-metabolism) (1 PurM_5-nucleotidase_AMP) |
| 70 | 1,00 | 1 | false | 1 | (1 PyrM_uridine-kinase_dGTP) |
| 71 | 1,00 | 4 | false | 3 | (1 AMP-energy_to_AMP-metabolism) (1 PurM_adenylate-kinase_AMP) (2 SERP0841-PurM_PNPase_ADP) |
| 72 | 1,00 | 1 | false | 1 | (1 PurM_5-nucleotidase_XMP) |
| 73 | 1,00 | 1 | false | 1 | (1 SERP0831-PurM_DNA-directed-DNA-polymerase_dATP) |
| 74 | 1,00 | 1 | false | 1 | (1 PyrM_uridine-kinase_dTTP) |
| 75 | 1,00 | 1 | false | 1 | (1 PyrM_cytidine-kinase_dCTP) |
| 76 | 1,00 | 1 | false | 1 | (1 SERP1952-macrolide-transport_efflux) |
| 77 | 1,00 | 2 | false | 2 | (1 PurM_deoxyadenosine-kinase_ATP) (-1 PyrM_deoxyadenosine-phosphorylase) |
| 78 | 0,53 | 1 | false | 1 | (1 PyrM_dUTP-diphosphatase) |
| 79 | 1,00 | 1 | false | 1 | (1 AS_Aspartate_to_beta-Alanine) |
| 80 | 1,00 | 1 | false | 1 | (1 SERP1803-cobalt/nickel-transport_efflux) |
| 81 | 1,28 | 1 | false | 1 | (1 PyrM_cytidine-kinase_dGTP) |
| 82 | 1,00 | 1 | false | 1 | (1 PurM_thioredoxin-oxidoreductase_dUTP) |
| 83 | 1,00 | 1 | false | 1 | (1 SERP0292-iron-dicitrate-transporter_import) |
| 84 | 0,25 | 2 | false | 2 | (1 Glyc_6-phosphofructokinase) (1 Glyc_fructose-bisphosphatase) |
| 85 | 1,00 | 1 | false | 1 | (1 PurM_XMP_L-glutamine-amide-ligase) |
| 86 | 1,00 | 1 | false | 1 | (1 AS_Aspartate_to_Arginine) |
| 87 | 1,00 | 48 | false | 20 | (1 AMP-energy_to_AMP-metabolism) (-4 Glyc_2-Phospho-D-glycerate-2.3-phosphomutase) (4 Glyc_2-phospho-D-glycerate-hydro-lyase) (2 Glyc_6-phospho-beta-glucosidase) (2 Glyc_6-phosphofructokinase) (-2 Glyc_acetaldehyde-dehydrogenase_NAD+) (-2 Glyc_Actetate-CoA-ligase) (2 Glyc_alpha-D-Glucose-6-phosphate-ketol-isomerase2) (2 Glyc_ATP-alpha-D-glucokinase) (-2 Glyc_ATP-beta-D-glucokinase) (-2 Glyc_D-Glucose-1-epimerase) (-2 Glyc_D-Glucose-1-epimerase-ketol-isomerase) (2 Glyc_fructose-bisphosphat-aldolase) (4 Glyc_glyceraldehyde-3-P-dehydrogenase_NAD+) (-4 Glyc_phosphoglycerate-kinase) (1 PurM_adenylate-kinase_AMP) (-2 SERP0389-Glyc_Ethanol_NAD+-oxidoreductase) (-2 TCA_citrate-hydro-lyase) (2 TCA_citrate_synthase) (-4 TCA_PEP-carboxylase) |
| 88 | 1,00 | 7 | false | 6 | (1 Glyc_acetaldehyde-dehydrogenase_NAD+) (1 Glyc_Actetate-CoA-ligase) (2 PyrM_thioredoxin-reductase) (1 SERP0389-Glyc_Ethanol_NAD+-oxidoreductase) (1 TCA_citrate-hydro-lyase) (-1 TCA_citrate_synthase) |
| 89 | 1,00 | 1 | false | 1 | (1 PyrM_2,3-cyclic-nucleotidase_UMP) |
| 90 | 0,13 | 1 | false | 1 | (1 DNA-extern_to_DNA-intern) |
| 91 | 1,00 | 1 | false | 1 | (1 SERP0831-PurM_DNA-directed-DNA-polymerase_dCTP) |
| 92 | 0,67 | 1 | false | 1 | (1 SERP0841-PurM_PNPase_GDP) |
| 93 | 0,38 | 2 | false | 2 | (1 PurM_carbamate-kinase_ATP) (1 PyrM_aspartate-carbamoyltransferase) |
| 94 | 0,16 | 1 | false | 1 | (1 PurM_thioredoxin-oxidoreductase_dGDP) |
| 95 | 1,00 | 1 | false | 1 | (1 PurM_ITP-diphosphohydrolase) |
| 96 | 0,31 | 3 | false | 2 | (2 PurM_nucleoside-diphosphate-phosphotransferase_ATP) (1 SERP0688-spermidine/putrescine-transport_import) |
| 97 | 0,58 | 3 | false | 3 | (1 PurM_5-nucleotidase_dCMP) (1 PyrM_Deoxycytidine-aminohydrolase) (1 PyrM_deoxyuridine-phosphorylase) |
| 98 | 1,00 | 2 | false | 2 | (1 PurM_nucleoside-diphosphate-phosphotransferase_ATP) (1 SERP0686-spermidine/putrescine-transport_import) |
| 99 | 1,00 | 1 | false | 1 | (1 PyrM_dUTP-diphosphohydrolase) |
| 100 | 0,38 | 1 | false | 1 | (1 PurM_dITP-diphosphohydrolase) |
| 101 | 1,00 | 2 | false | 2 | (1 PurM_pyruvate-phosphotransferase_GTP) (-1 TCA_PEP-carboxylase) |
| 102 | 0,58 | 2 | false | 2 | (1 PurM_pyruvate-phosphotransferase_dATP) (-1 TCA_PEP-carboxylase) |
| 103 | 1,00 | 1 | false | 1 | (1 PurM_5-nucleotidase_UMP) |
| 104 | 1,00 | 1 | false | 1 | (1 SERP2283-phopsphonate-transport_import) |
| 105 | 1,00 | 2 | false | 2 | (1 PurM_GDP-reductase) (1 SERP2179-choline/betaine/carnitine-transp_efflux) |
| 106 | 1,00 | 1 | false | 1 | (1 AS_Valine) |
| 107 | 0,49 | 2 | false | 2 | (1 PurM_5-nucleotidase_dAMP) (1 PyrM_deoxyadenosine-phosphorylase) |
| 108 | 1,00 | 1 | false | 1 | (1 SERP1802-cobalt/nickel-transport_efflux) |
| 109 | 0,72 | 1 | false | 1 | (1 PurM_DNA-directed-RNA-polyermase_UTP) |
| 110 | 1,00 | 4 | false | 4 | (-1 TCA_lipoic-transsuccinylase) (1 TCA_oxoglutarate-dehydrogenase-complex1) (1 TCA_oxoglutarate-dehydrogenase-complex2) (1 TCA_oxoglutarate-synthase) |
| 111 | 1,00 | 1 | false | 1 | (1 PyrM_cytidine-kinase_dTTP) |
| 112 | 1,00 | 1 | false | 1 | (1 PurM_UTP-diphosphohydrolase) |
| 113 | 1,00 | 1 | false | 1 | (1 AS_Serine_to_Methionine) |
| 114 | 1,00 | 2 | false | 2 | (-1 AMP-energy_to_AMP-metabolism) (1 PurM_deoxycytidine-kinase_ATP) |
| 115 | 1,00 | 2 | false | 2 | (1 PurM_5-nucleotidase_dGMP) (1 PyrM_deoxyguanosine-phosphorylase) |
| 116 | 0,12 | 1 | false | 1 | (1 PurM_thioredoxin-oxidoreductase_dGTP) |
| 117 | 0,12 | 3 | false | 3 | (1 IQ-143-extern_to_IQ-) (1 SERP1944-MultiDrug-transport_efflux) (1 SERP2179-choline/betaine/carnitine-transp_efflux) |
| 118 | 1,00 | 1 | false | 1 | (1 PyrM_cytidine-kinase) |
| 119 | 0,38 | 1 | false | 1 | (1 AS_Leucine) |
| 120 | 0,38 | 48 | false | 20 | (1 AMP-energy_to_AMP-metabolism) (-4 Glyc_2-Phospho-D-glycerate-2.3-phosphomutase) (4 Glyc_2-phospho-D-glycerate-hydro-lyase) (2 Glyc_6-phospho-beta-glucosidase) (2 Glyc_6-phosphofructokinase) (-2 Glyc_acetaldehyde-dehydrogenase_NAD+) (-2 Glyc_Actetate-CoA-ligase) (2 Glyc_alpha-D-Glucose-6-phosphate-ketol-isomerase2) (2 Glyc_ATP-alpha-D-glucokinase) (-2 Glyc_ATP-beta-D-glucokinase) (-2 Glyc_D-Glucose-1-epimerase) (-2 Glyc_D-Glucose-1-epimerase-ketol-isomerase) (2 Glyc_fructose-bisphosphat-aldolase) (4 Glyc_glyceraldehyde-3-P-dehydrogenase_NADP+) (-4 Glyc_phosphoglycerate-kinase) (1 PurM_adenylate-kinase_AMP) (-2 SERP0389-Glyc_Ethanol_NAD+-oxidoreductase) (-2 TCA_citrate-hydro-lyase) (2 TCA_citrate_synthase) (-4 TCA_PEP-carboxylase) |
| 121 | 0,25 | 1 | false | 1 | (1 PyrM_2,3-cyclic-nucleotidase_CMP) |
| 122 | 0,22 | 2 | false | 2 | (1 SERP1997-formate/nitrite-transport_efflux/import) (1 SERP2179-choline/betaine/carnitine-transp_efflux) |
| 123 | 1,00 | 1 | false | 1 | (1 PyrM_CTP-synthase) |
| 124 | 1,00 | 2 | false | 2 | (1 IQ-143-extern_to_IQ-) (1 SERP2289-MultiDrug-transport_efflux) |
| 125 | 1,00 | 1 | false | 1 | (1 AS_Glutamate_to_Proline) |
| 126 | 0,75 | 1 | false | 1 | (1 PyrM_uridine-kinase_dCTP) |
| 127 | 1,50 | 1 | false | 1 | (1 PyrM_uridine-kinase_UTP) |
| 128 | 1,00 | 3 | false | 3 | (1 PurM_deoxycytidine-kinase_ATP2) (-1 PyrM_Deoxycytidine-aminohydrolase) (-1 PyrM_deoxyuridine-phosphorylase) |
| 129 | 1,00 | 1 | false | 1 | (1 AS_Threonine) |
| 130 | 0,60 | 1 | false | 1 | (1 PurM_allantoinase) |
| 131 | 1,00 | 1 | false | 1 | (1 SERP0831-PurM_DNA-directed-DNA-polymerase_dTTP) |
| 132 | 1,00 | 1 | false | 1 | (1 PurM_GTP-diphosphohydrolase) |
| 133 | 1,00 | 1 | false | 1 | (1 PyrM_uridine-kinase_dATP) |
| 134 | 1,00 | 1 | false | 1 | (1 AS_Serine_to_Pyruvate) |
| 135 | 1,00 | 2 | false | 2 | (1 SERP0290-zinc-transport_efflux) (1 SERP0291-zinc-transporter_import) |
| 136 | 1,00 | 1 | false | 1 | (1 AS_Isoleucine) |
| 137 | 0,55 | 1 | false | 1 | (1 PurM_thioredoxin-oxidoreductase_dCDP) |
| 138 | 1,00 | 2 | false | 2 | (1 PurM_pyruvate-phosphotransferase_dGTP) (-1 TCA_PEP-carboxylase) |
| 139 | 0,38 | 1 | false | 1 | (1 PurM_5-nucleotidase_dTMP) |
| 140 | 0,12 | 4 | false | 4 | (1 PurM_D-Ribose-1,5-phosphomutase) (-1 PurM_IMP-pyrophosphorylase) (1 PurM_nucleotide-phosphatase_Inosine) (1 PurM_PRPP-synthetase) |
| 141 | 0,38 | 1 | false | 1 | (1 PyrM_uridine-kinase_ATP) |
| 142 | 1,00 | 1 | false | 1 | (1 PurM_DNA-directed-RNA-polyermase_GTP) |
| 143 | 1,00 | 7 | false | 6 | (-1 Glyc_acetaldehyde-dehydrogenase_NAD+) (-1 Glyc_Actetate-CoA-ligase) (2 Glyc_dihydrolipoamide-dehydrogenase) (-1 SERP0389-Glyc_Ethanol_NAD+-oxidoreductase) (-1 TCA_citrate-hydro-lyase) (1 TCA_citrate_synthase) |
| 144 | 1,00 | 1 | false | 1 | (1 PyrM_OMP-decarboxylase) |
| 145 | 1,00 | 1 | false | 1 | (1 AS_Glutamate_to_Glutamine) |
| 146 | 1,00 | 1 | false | 1 | (1 PurM_urea-amidohydrolase) |
| 147 | 1,00 | 4 | false | 3 | (1 AMP-energy_to_AMP-metabolism) (1 PurM_adenylate-kinase_AMP) (2 PurM_thioredoxin-oxidoreductase_dADP) |
| 148 | 0,60 | 1 | false | 1 | (1 AS_Aspartate_to_Asparagine) |
| 149 | 0,41 | 1 | false | 1 | (1 PyrM_cytidine-kinase_ATP) |
| 150 | 0,81 | 2 | false | 2 | (1 PurM_GDP-reductase) (1 SERP0765-Uracil-permease-transport_import) |
| 151 | 0,64 | 3 | false | 3 | (1 IQ-143-extern_to_IQ-) (1 SERP0765-Uracil-permease-transport_import) (1 SERP1944-MultiDrug-transport_efflux) |
| 152 | 1,00 | 2 | false | 2 | (1 SERP0765-Uracil-permease-transport_import) (1 SERP1997-formate/nitrite-transport_efflux/import) |
| 153 | 1,00 | 13 | false | 9 | (-1 Glyc_acetaldehyde-dehydrogenase_NAD+) (-1 Glyc_Actetate-CoA-ligase) (-1 SERP0389-Glyc_Ethanol_NAD+-oxidoreductase) (-1 TCA_citrate-hydro-lyase) (1 TCA_citrate_synthase) (-2 TCA_lipoic-transsuccinylase) (2 TCA_Oxidoreductase) (2 TCA_oxoglutarate-dehydrogenase-complex1) (2 TCA_oxoglutarate-dehydrogenase-complex2) |
| 154 | 0,57 | 1 | false | 1 | (1 PurM_XTP-diphosphohydrolase) |
| 155 | 0,45 | 3 | false | 3 | (-1 PurM_carbamate-kinase_ATP) (1 PyrM_CO2_L-glutamine-amido-ligase) (-1 TCA_Pyruvate_CO2-ligase) |
| 156 | 1,00 | 12 | false | 7 | (2 Glyc_acetaldehyde-dehydrogenase_NAD+) (2 Glyc_Actetate-CoA-ligase) (1 OP_complex1) (1 OP_complex3) (2 SERP0389-Glyc_Ethanol_NAD+-oxidoreductase) (2 TCA_citrate-hydro-lyase) (-2 TCA_citrate_synthase) |
| 157 | 1,00 | 17 | false | 7 | (3 Glyc_acetaldehyde-dehydrogenase_NAD+) (3 Glyc_Actetate-CoA-ligase) (1 OP_complex2) (1 OP_complex3) (3 SERP0389-Glyc_Ethanol_NAD+-oxidoreductase) (3 TCA_citrate-hydro-lyase) (-3 TCA_citrate_synthase) |
| 158 | 0,22 | 1 | false | 1 | (1 PurM_GTP-pyrophosphokinase) |
| 159 | 1,00 | 6 | false | 6 | (1 Glyc_acetaldehyde-dehydrogenase_NAD+) (1 Glyc_Actetate-CoA-ligase) (1 OP_complex4) (1 SERP0389-Glyc_Ethanol_NAD+-oxidoreductase) (1 TCA_citrate-hydro-lyase) (-1 TCA_citrate_synthase) |
| 160 | 1,00 | 2 | false | 2 | (1 Glyc_pyruvate_dehydrogenase) (1 TCA_pyruvate_dehydrogenase) |
| 161 | 0,27 | 17 | false | 6 | (-3 Glyc_acetaldehyde-dehydrogenase_NAD+) (-3 Glyc_Actetate-CoA-ligase) (2 OP_complex5) (-3 SERP0389-Glyc_Ethanol_NAD+-oxidoreductase) (-3 TCA_citrate-hydro-lyase) (3 TCA_citrate_synthase) |
| 162 | 1,00 | 1 | false | 1 | (1 AS_Aspartate_to_Alanine) |
| 163 | 1,00 | 2 | false | 2 | (1 AS_Serine_to_Cysteine) (1 Glyc_Actetate-CoA-ligase) |
| 164 | 1,00 | 6 | false | 4 | (1 AMP-energy_to_AMP-metabolism) (1 PurM_adenylate-kinase_AMP) (2 PurM_nucleoside-diphosphate-phosphotransferase_ATP) (2 PurM_thioredoxin-oxidoreductase_dATP) |
| 165 | 1,00 | 1 | false | 1 | (1 SERP2186-PurM_ATP_sulfate-adenylyltransferase) |
| 166 | 0,25 | 3 | false | 3 | (-1 AMP-energy_to_AMP-metabolism) (1 PurM_ADP-ribose-ribophosphohydrolase) (1 PurM_PRPP-synthetase) |
| 167 | 1,25 | 2 | false | 2 | (1 IQ-143-extern_to_IQ-) (1 SERP1403-MultiDrug-transport_efflux) |
| 168 | 1,00 | 13 | false | 3 | (1 FA_Syn_Acetyl-CoA_to_C16) (-6 TCA_citrate-hydro-lyase) (6 TCA_citrate_synthase) |
| 169 | 0,25 | 1 | false | 1 | (1 PyrM_UTP_L-glutamine-amido-ligase) |
| 170 | 1,00 | 1 | false | 1 | (1 AS_Phenylalanin_to_Tyrosine) |
| 171 | 1,00 | 1 | false | 1 | (1 PurM_5-nucleotidase_IMP) |
| 172 | 1,00 | 6 | false | 4 | (1 AMP-energy_to_AMP-metabolism) (1 PurM_adenylate-kinase_AMP) (2 PurM_DNA-directed-RNA-polyermase_ATP) (2 PurM_nucleoside-diphosphate-phosphotransferase_ATP) |
| 173 | 0,50 | 2 | false | 2 | (1 PurM_GDP-reductase) (1 PurM_IMP-dehydrogenase) |
| 174 | 0,49 | 3 | false | 3 | (1 IQ-143-extern_to_IQ-) (1 PurM_IMP-dehydrogenase) (1 SERP1944-MultiDrug-transport_efflux) |
| 175 | 0,25 | 2 | false | 2 | (1 PurM_IMP-dehydrogenase) (1 SERP1997-formate/nitrite-transport_efflux/import) |
| 176 | 1,00 | 1 | false | 1 | (1 PurM_5-nucleotidase_GMP) |
| 177 | 1,00 | 1 | false | 1 | (1 PyrM_cytidine-kinase_ITP) |
| 178 | 1,00 | 1 | false | 1 | (1 AS_Histidine_to_Glutamate) |
| 179 | 1,00 | 1 | false | 1 | (1 PyrM_cytidine-kinase_GTP) |
| 180 | 0,75 | 13 | false | 3 | (1 FA_Deg_C16_to_Acetyl-CoA) (6 TCA_citrate-hydro-lyase) (-6 TCA_citrate_synthase) |
| 181 | 1,00 | 3 | false | 3 | (1 PurM_nucleoside-diphosphate-phosphotransferase_ATP) (1 PurM_pyruvate-phosphotransferase_ATP) (-1 TCA_PEP-carboxylase) |
| 182 | 1,00 | 1 | false | 1 | (1 PyrM_cytidine-kinase_dUTP) |
| 183 | 0,16 | 3 | false | 2 | (2 PurM_nucleoside-diphosphate-phosphotransferase_ATP) (1 SERP0687-spermidine/putrescine-transport_import) |
| 184 | 0,27 | 1 | false | 1 | (1 PurM_thioredoxin-oxidoreductase_dUDP) |
| 185 | 1,00 | 1 | false | 1 | (1 PurM_dGTP-diphosphohydrolase) |
| 186 | 1,00 | 1 | false | 1 | (1 PurM_DNA-directed-RNA-polyermase_CTP) |
| 187 | 1,00 | 1 | false | 1 | (1 SERP0831-PurM_DNA-directed-DNA-polymerase_dGTP) |
| 188 | 1,00 | 1 | false | 1 | (1 PurM_thioredoxin-oxidoreductase_dCTP) |
| 189 | 1,00 | 1 | false | 1 | (1 AS_Tryptophan_to_Tryptamine) |
| 190 | 1,04 | 3 | false | 3 | (1 AS_Acetyl-CoA_to_L-Leucine) (-1 TCA_citrate-hydro-lyase) (1 TCA_citrate_synthase) |
| 191 | 1,00 | 1 | false | 1 | (1 AS_Homoserine_to_Threonine) |
| 192 | 1,00 | 1 | false | 1 | (1 PurM_5-nucleotidase_CMP) |
| 193 | 1,00 | 1 | false | 1 | (1 PyrM_uridine-kinase_dUTP) |
| 194 | 1,00 | 1 | false | 1 | (1 PyrM_uridine-kinase_ITP) |
| 195 | 1,00 | 1 | false | 1 | (1 PurM_XMP-ligase) |
| 196 | 1,08 | 1 | false | 1 | (1 PyrM_cytidine-kinase_dATP) |
| 197 | 1,00 | 1 | false | 1 | (1 SERP2060-glyerol-transport_import) |
| 198 | 1,00 | 1 | false | 1 | (1 PyrM_uridine-kinase_GTP) |

^1^ This data shows the elementary mode Analysis for *S. aureus* USA300 without IQ-143.

Table S8: Extreme Modes of *S. aureus* USA300 with 0.16µM IQ-143^1^.

| # | Activity | Flux sum | Reversible? | Pathlength | Reactions |
| --- | --- | --- | --- | --- | --- |
| 1 | 1,00 | 1 | true | 1 | (1 AS_Alanine_to_Pyruvate) |
| 2 | 1,00 | 1 | true | 1 | (1 PyrM_dCMP-aminohydrolase) |
| 3 | -0,66 | 1 | true | 1 | (1 PyrM_GMP-pyrophosphorylase) |
| 4 | 1,00 | 1 | true | 1 | (1 PurM_ATP_IDP-phosphotransferase) |
| 5 | 1,00 | 1 | true | 1 | (1 PyrM_cytidilate-kinase_dCMP) |
| 6 | 1,00 | 1 | true | 1 | (1 PyrM_dUMP-phosphotransferase) |
| 7 | 1,00 | 1 | true | 1 | (1 PurM_ATP_GMP-guanylate-kinase) |
| 8 | 1,00 | 1 | true | 1 | (1 PyrM_cytidine-aminohydrolase) |
| 9 | 1,00 | 1 | true | 1 | (1 PurM_ATP_UTP-phosphotransferase) |
| 10 | 1,00 | 1 | true | 1 | (1 PurM_ATP_GTP-phosphotransferase) |
| 11 | 1,00 | 2 | true | 2 | (-1 PurM_nucleotide-phosphatase_Deoxyguanosine) (1 PyrM_deoxyguanosine-phosphorylase) |
| 12 | 1,00 | 2 | true | 2 | (-1 PyrM_dihydroorotase) (1 PyrM_dihydroorotate-oxidase) |
| 13 | 1,00 | 1 | true | 1 | (1 PurM_ATP_dIDP-phosphotransferase) |
| 14 | 1,00 | 1 | true | 1 | (1 PyrM_thymidine-kinase_dTMP) |
| 15 | 1,00 | 1 | true | 1 | (1 PurM_xanthosine-phosphoribosyltransferase) |
| 16 | 1,00 | 1 | true | 1 | (1 PurM_ATP_dUDP-phosphotransferase) |
| 17 | 0,98 | 1 | true | 1 | (1 TCA_fumarate-hydratase) |
| 18 | 1,00 | 3 | true | 3 | (1 PurM_IMP-pyrophosphorylase) (-1 PurM_nucleotide-phosphatase_Inosine) (1 PyrM_uridine-phosphorylase) |
| 19 | 1,00 | 3 | true | 3 | (1 PurM_IMP-pyrophosphorylase) (1 PurM_nucleotide-phosphatase_Guanosine) (-1 PurM_nucleotide-phosphatase_Inosine) |
| 20 | 1,00 | 3 | true | 3 | (1 PurM_IMP-pyrophosphorylase) (-1 PurM_nucleotide-phosphatase_Inosine) (1 PurM_nucleotide-phosphatase_Xanthosine) |
| 21 | 1,00 | 3 | true | 3 | (1 PurM_IMP-pyrophosphorylase) (-1 PurM_nucleotide-phosphatase_Deoxyinosine) (1 PyrM_deoxyinosine-phosphorylase) |
| 22 | 1,00 | 1 | true | 1 | (1 AS_Serine_to_Glycine) |
| 23 | 1,00 | 1 | true | 1 | (1 SERP1951-lipoprotein-transport_efflux/import) |
| 24 | 1,00 | 1 | true | 1 | (1 PurM_GMP-pyrophosphorylase2) |
| 25 | -0,57 | 1 | true | 1 | (1 PyrM_nucleoside-phosphate-kinase_ATP) |
| 26 | 1,00 | 1 | true | 1 | (1 PyrM_ATP_dTDP_thymidylate-kinase) |
| 27 | 1,00 | 1 | true | 1 | (1 PurM_ATP_dTDP-phosphotransferase) |
| 28 | 1,00 | 1 | true | 1 | (1 PurM_ATP_GMP_guanylate-kinase) |
| 29 | 1,00 | 1 | true | 1 | (1 PyrM_nucleoside-phosphate-kinase_ATP2) |
| 30 | -1,33 | 2 | true | 2 | (-1 PurM_nucleotide-phosphatase_Deoxyuridine) (1 PyrM_deoxyuridine-phosphorylase) |
| 31 | 1,00 | 2 | true | 2 | (1 PyrM_deoxyuridine-phosphorylase) (-1 PyrM_thymidine-kinase_dUMP) |
| 32 | 1,00 | 1 | true | 1 | (1 PurM_ATP_dGDP-phosphotransferase) |
| 33 | 1,00 | 1 | true | 1 | (1 AS_Aspartate_to_Homoserine) |
| 34 | 1,00 | 1 | true | 1 | (1 PurM_ATP_dADP-phosphotransferase) |
| 35 | 0,98 | 2 | true | 2 | (1 TCA_citrate-hydro-lyase) (-1 TCA_citrate-hydroxymutase) |
| 36 | 1,00 | 3 | true | 3 | (1 Glyc_lipoic_acetyltransferase) (-1 TCA_citrate-hydro-lyase) (1 TCA_citrate_synthase) |
| 37 | 1,00 | 7 | true | 6 | (-1 Glyc_acetaldehyde-dehydrogenase_NAD+) (-1 Glyc_Actetate-CoA-ligase) (-1 SERP0389-Glyc_Ethanol_NAD+-oxidoreductase) (2 SERP2156-Glyc_L-lactate-dehydrogenase) (-1 TCA_citrate-hydro-lyase) (1 TCA_citrate_synthase) |
| 38 | 1,00 | 1 | true | 1 | (1 PurM_ATP_CDP-phosphotransferase) |
| 39 | 1,00 | 1 | true | 1 | (1 AS_Saccharopine_to_Lysine) |
| 40 | 1,00 | 1 | true | 1 | (1 PurM_IMP_L-aspartate-ligase) |
| 41 | 1,00 | 1 | true | 1 | (1 Glyc_Succinate-CoA-ligase) |
| 42 | 1,00 | 3 | true | 3 | (1 AS_Acetyl-CoA_to_L-Valine) (-1 TCA_citrate-hydro-lyase) (1 TCA_citrate_synthase) |
| 43 | 1,00 | 1 | true | 1 | (1 PyrM_orotate-phosphoribosyltransferase) |
| 44 | 1,00 | 1 | true | 1 | (1 PurM_metaphosphatase) |
| 45 | 0,50 | 1 | true | 1 | (1 PyrM_cytidilate-kinase_CTP) |
| 46 | 1,00 | 1 | true | 1 | (1 Glyc_PTS-permease2) |
| 47 | 1,00 | 1 | true | 1 | (1 PyrM_thymidine-phosphorylase) |
| 48 | 1,00 | 1 | true | 1 | (1 PurM_ATP_dCDP-phosphotransferase) |
| 49 | -0,39 | 1 | true | 1 | (1 PyrM_UMP-pyrophosphorylase) |
| 50 | 0,39 | 1 | true | 1 | (1 PyrM_nucleoside-triphosphate-adenylate-kinase) |
| 51 | 0,39 | 1 | true | 1 | (1 PurM_XMP-pyrophosphorylase) |
| 52 | 0,39 | 2 | true | 2 | (1 PyrM_Deoxycytidine-aminohydrolase) (-1 PyrM_Deoxycytidine-deaminase) |
| 53 | 0,39 | 2 | true | 2 | (-1 PurM_nucleotide-phosphatase_Deoxyadenosine) (1 PyrM_deoxyadenosine-phosphorylase) |
| 54 | 0,39 | 1 | true | 1 | (1 PyrM_ATP_dUDP_thymidylate-kinase) |
| 55 | 0,79 | 1 | true | 1 | (1 PurM_adenylate-kinase_dAMP) |
| 56 | 0,88 | 2 | true | 2 | (1 AMP-energy_to_AMP-metabolism) (1 PurM_AMP-pyrophosphorylase2) |
| 57 | 1,12 | 2 | true | 2 | (1 AMP-energy_to_AMP-metabolism) (1 PyrM_AMP-pyrophosphorylase) |
| 58 | 0,51 | 10 | true | 6 | (1 AMP-energy_to_AMP-metabolism) (2 Glyc_alpha-D-Glucose-6-phosphate-ketol-isomerase) (2 Glyc_ATP-alpha-D-glucokinase) (-2 Glyc_ATP-beta-D-glucokinase) (-2 Glyc_D-Glucose-1-epimerase) (1 PurM_adenylate-kinase_AMP) |
| 59 | 0,37 | 6 | true | 4 | (-1 AMP-energy_to_AMP-metabolism) (2 ATP-energy_to_ATP-metabolism) (-1 PurM_adenylate-kinase_AMP) (-2 PurM_nucleoside-diphosphate-phosphotransferase_ATP) |
| 60 | 0,38 | 2 | true | 2 | (-1 AMP-energy_to_AMP-metabolism) (1 PurM_adenylosuccinate-lyase) |
| 61 | 0,88 | 12 | true | 7 | (-1 AMP-energy_to_AMP-metabolism) (-2 Glyc_alpha-D-Glucose-6-phosphate-ketol-isomerase2) (-2 Glyc_ATP-alpha-D-glucokinase) (2 Glyc_ATP-beta-D-glucokinase) (2 Glyc_beta-D-Glucose-6-phosphate-ketol-isomerase) (2 Glyc_D-Glucose-1-epimerase) (-1 PurM_adenylate-kinase_AMP) |
| 62 | 0,60 | 12 | true | 7 | (-1 AMP-energy_to_AMP-metabolism) (-2 Glyc_6-phospho-beta-glucosidase) (-2 Glyc_ATP-alpha-D-glucokinase) (2 Glyc_ATP-beta-D-glucokinase) (2 Glyc_D-Glucose-1-epimerase) (2 Glyc_PTS-permease1) (-1 PurM_adenylate-kinase_AMP) |
| 63 | 1,00 | 1 | true | 1 | (1 PurM_adenylylsulfate-kinase) |
| 64 | 1,00 | 4 | true | 3 | (2 ADP-energy_to_ADP-metabolism) (-1 AMP-energy_to_AMP-metabolism) (-1 PurM_adenylate-kinase_AMP) |
| 65 | 1,00 | 3 | true | 3 | (1 PurM_IMP-pyrophosphorylase) (1 PurM_nucleotide-phosphatase_Adenine) (-1 PurM_nucleotide-phosphatase_Inosine) |
| 66 | 1,00 | 3 | true | 3 | (1 PurM_IMP-pyrophosphorylase) (-1 PurM_nucleotide-phosphatase_Inosine) (1 PyrM_pyrimidine-nucleoside-phosphorylase) |
| 67 | 1,00 | 1 | true | 1 | (1 TCA_isocitrate-hydro-lyase) |
| 68 | 1,00 | 2 | false | 2 | (1 PurM_ATP-phosphohydrolase) (1 PurM_nucleoside-diphosphate-phosphotransferase_ATP) |
| 69 | 1,00 | 2 | false | 2 | (1 AMP-energy_to_AMP-metabolism) (1 PurM_5-nucleotidase_AMP) |
| 70 | 1,00 | 1 | false | 1 | (1 PyrM_uridine-kinase_dGTP) |
| 71 | 1,00 | 4 | false | 3 | (1 AMP-energy_to_AMP-metabolism) (1 PurM_adenylate-kinase_AMP) (2 SERP0841-PurM_PNPase_ADP) |
| 72 | 1,00 | 1 | false | 1 | (1 PurM_5-nucleotidase_XMP) |
| 73 | 1,00 | 1 | false | 1 | (1 SERP0831-PurM_DNA-directed-DNA-polymerase_dATP) |
| 74 | 1,00 | 1 | false | 1 | (1 PyrM_uridine-kinase_dTTP) |
| 75 | 1,00 | 1 | false | 1 | (1 PyrM_cytidine-kinase_dCTP) |
| 76 | 1,00 | 1 | false | 1 | (1 SERP1952-macrolide-transport_efflux) |
| 77 | 1,00 | 2 | false | 2 | (1 PurM_deoxyadenosine-kinase_ATP) (-1 PyrM_deoxyadenosine-phosphorylase) |
| 78 | 0,51 | 1 | false | 1 | (1 PyrM_dUTP-diphosphatase) |
| 79 | 1,00 | 1 | false | 1 | (1 AS_Aspartate_to_beta-Alanine) |
| 80 | 1,00 | 1 | false | 1 | (1 SERP1803-cobalt/nickel-transport_efflux) |
| 81 | 1,12 | 1 | false | 1 | (1 PyrM_cytidine-kinase_dGTP) |
| 82 | 1,00 | 1 | false | 1 | (1 PurM_thioredoxin-oxidoreductase_dUTP) |
| 83 | 0,54 | 1 | false | 1 | (1 SERP0292-iron-dicitrate-transporter_import) |
| 84 | 0,25 | 2 | false | 2 | (1 Glyc_6-phosphofructokinase) (1 Glyc_fructose-bisphosphatase) |
| 85 | 1,00 | 1 | false | 1 | (1 PurM_XMP_L-glutamine-amide-ligase) |
| 86 | 1,00 | 1 | false | 1 | (1 AS_Aspartate_to_Arginine) |
| 87 | 1,00 | 48 | false | 20 | (1 AMP-energy_to_AMP-metabolism) (-4 Glyc_2-Phospho-D-glycerate-2.3-phosphomutase) (4 Glyc_2-phospho-D-glycerate-hydro-lyase) (2 Glyc_6-phospho-beta-glucosidase) (2 Glyc_6-phosphofructokinase) (-2 Glyc_acetaldehyde-dehydrogenase_NAD+) (-2 Glyc_Actetate-CoA-ligase) (2 Glyc_alpha-D-Glucose-6-phosphate-ketol-isomerase2) (2 Glyc_ATP-alpha-D-glucokinase) (-2 Glyc_ATP-beta-D-glucokinase) (-2 Glyc_D-Glucose-1-epimerase) (-2 Glyc_D-Glucose-1-epimerase-ketol-isomerase) (2 Glyc_fructose-bisphosphat-aldolase) (4 Glyc_glyceraldehyde-3-P-dehydrogenase_NAD+) (-4 Glyc_phosphoglycerate-kinase) (1 PurM_adenylate-kinase_AMP) (-2 SERP0389-Glyc_Ethanol_NAD+-oxidoreductase) (-2 TCA_citrate-hydro-lyase) (2 TCA_citrate_synthase) (-4 TCA_PEP-carboxylase) |
| 88 | 1,00 | 7 | false | 6 | (1 Glyc_acetaldehyde-dehydrogenase_NAD+) (1 Glyc_Actetate-CoA-ligase) (2 PyrM_thioredoxin-reductase) (1 SERP0389-Glyc_Ethanol_NAD+-oxidoreductase) (1 TCA_citrate-hydro-lyase) (-1 TCA_citrate_synthase) |
| 89 | 1,00 | 1 | false | 1 | (1 PyrM_2,3-cyclic-nucleotidase_UMP) |
| 90 | 1,00 | 1 | false | 1 | (1 DNA-extern_to_DNA-intern) |
| 91 | 1,00 | 1 | false | 1 | (1 SERP0831-PurM_DNA-directed-DNA-polymerase_dCTP) |
| 92 | 0,67 | 1 | false | 1 | (1 SERP0841-PurM_PNPase_GDP) |
| 93 | 0,00 | 2 | false | 2 | (1 PurM_carbamate-kinase_ATP) (1 PyrM_aspartate-carbamoyltransferase) |
| 94 | 0,23 | 1 | false | 1 | (1 PurM_thioredoxin-oxidoreductase_dGDP) |
| 95 | 1,00 | 1 | false | 1 | (1 PurM_ITP-diphosphohydrolase) |
| 96 | 0,33 | 3 | false | 2 | (2 PurM_nucleoside-diphosphate-phosphotransferase_ATP) (1 SERP0688-spermidine/putrescine-transport_import) |
| 97 | 0,61 | 3 | false | 3 | (1 PurM_5-nucleotidase_dCMP) (1 PyrM_Deoxycytidine-aminohydrolase) (1 PyrM_deoxyuridine-phosphorylase) |
| 98 | 1,00 | 2 | false | 2 | (1 PurM_nucleoside-diphosphate-phosphotransferase_ATP) (1 SERP0686-spermidine/putrescine-transport_import) |
| 99 | 1,00 | 1 | false | 1 | (1 PyrM_dUTP-diphosphohydrolase) |
| 100 | 2,02 | 1 | false | 1 | (1 PurM_dITP-diphosphohydrolase) |
| 101 | 1,00 | 2 | false | 2 | (1 PurM_pyruvate-phosphotransferase_GTP) (-1 TCA_PEP-carboxylase) |
| 102 | 0,62 | 2 | false | 2 | (1 PurM_pyruvate-phosphotransferase_dATP) (-1 TCA_PEP-carboxylase) |
| 103 | 1,00 | 1 | false | 1 | (1 PurM_5-nucleotidase_UMP) |
| 104 | 1,00 | 1 | false | 1 | (1 SERP2283-phopsphonate-transport_import) |
| 105 | 1,00 | 2 | false | 2 | (1 PurM_GDP-reductase) (1 SERP2179-choline/betaine/carnitine-transp_efflux) |
| 106 | 1,00 | 1 | false | 1 | (1 AS_Valine) |
| 107 | 0,40 | 2 | false | 2 | (1 PurM_5-nucleotidase_dAMP) (1 PyrM_deoxyadenosine-phosphorylase) |
| 108 | 1,00 | 1 | false | 1 | (1 SERP1802-cobalt/nickel-transport_efflux) |
| 109 | 0,88 | 1 | false | 1 | (1 PurM_DNA-directed-RNA-polyermase_UTP) |
| 110 | 1,00 | 4 | false | 4 | (-1 TCA_lipoic-transsuccinylase) (1 TCA_oxoglutarate-dehydrogenase-complex1) (1 TCA_oxoglutarate-dehydrogenase-complex2) (1 TCA_oxoglutarate-synthase) |
| 111 | 1,00 | 1 | false | 1 | (1 PyrM_cytidine-kinase_dTTP) |
| 112 | 1,00 | 1 | false | 1 | (1 PurM_UTP-diphosphohydrolase) |
| 113 | 1,00 | 1 | false | 1 | (1 AS_Serine_to_Methionine) |
| 114 | 1,00 | 2 | false | 2 | (-1 AMP-energy_to_AMP-metabolism) (1 PurM_deoxycytidine-kinase_ATP) |
| 115 | 1,00 | 2 | false | 2 | (1 PurM_5-nucleotidase_dGMP) (1 PyrM_deoxyguanosine-phosphorylase) |
| 116 | 0,00 | 1 | false | 1 | (1 PurM_thioredoxin-oxidoreductase_dGTP) |
| 117 | 2,02 | 3 | false | 3 | (1 IQ-143-extern_to_IQ-) (1 SERP1944-MultiDrug-transport_efflux) (1 SERP2179-choline/betaine/carnitine-transp_efflux) |
| 118 | 1,00 | 1 | false | 1 | (1 PyrM_cytidine-kinase) |
| 119 | 0,00 | 1 | false | 1 | (1 AS_Leucine) |
| 120 | 2,02 | 48 | false | 20 | (1 AMP-energy_to_AMP-metabolism) (-4 Glyc_2-Phospho-D-glycerate-2.3-phosphomutase) (4 Glyc_2-phospho-D-glycerate-hydro-lyase) (2 Glyc_6-phospho-beta-glucosidase) (2 Glyc_6-phosphofructokinase) (-2 Glyc_acetaldehyde-dehydrogenase_NAD+) (-2 Glyc_Actetate-CoA-ligase) (2 Glyc_alpha-D-Glucose-6-phosphate-ketol-isomerase2) (2 Glyc_ATP-alpha-D-glucokinase) (-2 Glyc_ATP-beta-D-glucokinase) (-2 Glyc_D-Glucose-1-epimerase) (-2 Glyc_D-Glucose-1-epimerase-ketol-isomerase) (2 Glyc_fructose-bisphosphat-aldolase) (4 Glyc_glyceraldehyde-3-P-dehydrogenase_NADP+) (-4 Glyc_phosphoglycerate-kinase) (1 PurM_adenylate-kinase_AMP) (-2 SERP0389-Glyc_Ethanol_NAD+-oxidoreductase) (-2 TCA_citrate-hydro-lyase) (2 TCA_citrate_synthase) (-4 TCA_PEP-carboxylase) |
| 121 | 0,25 | 1 | false | 1 | (1 PyrM_2,3-cyclic-nucleotidase_CMP) |
| 122 | 1,00 | 2 | false | 2 | (1 SERP1997-formate/nitrite-transport_efflux/import) (1 SERP2179-choline/betaine/carnitine-transp_efflux) |
| 123 | 1,00 | 1 | false | 1 | (1 PyrM_CTP-synthase) |
| 124 | 0,80 | 2 | false | 2 | (1 IQ-143-extern_to_IQ-) (1 SERP2289-MultiDrug-transport_efflux) |
| 125 | 1,00 | 1 | false | 1 | (1 AS_Glutamate_to_Proline) |
| 126 | 0,75 | 1 | false | 1 | (1 PyrM_uridine-kinase_dCTP) |
| 127 | 1,50 | 1 | false | 1 | (1 PyrM_uridine-kinase_UTP) |
| 128 | 1,00 | 3 | false | 3 | (1 PurM_deoxycytidine-kinase_ATP2) (-1 PyrM_Deoxycytidine-aminohydrolase) (-1 PyrM_deoxyuridine-phosphorylase) |
| 129 | 1,00 | 1 | false | 1 | (1 AS_Threonine) |
| 130 | 1,40 | 1 | false | 1 | (1 PurM_allantoinase) |
| 131 | 1,00 | 1 | false | 1 | (1 SERP0831-PurM_DNA-directed-DNA-polymerase_dTTP) |
| 132 | 1,00 | 1 | false | 1 | (1 PurM_GTP-diphosphohydrolase) |
| 133 | 0,47 | 1 | false | 1 | (1 PyrM_uridine-kinase_dATP) |
| 134 | 1,00 | 1 | false | 1 | (1 AS_Serine_to_Pyruvate) |
| 135 | 1,00 | 2 | false | 2 | (1 SERP0290-zinc-transport_efflux) (1 SERP0291-zinc-transporter_import) |
| 136 | 1,00 | 1 | false | 1 | (1 AS_Isoleucine) |
| 137 | 0,57 | 1 | false | 1 | (1 PurM_thioredoxin-oxidoreductase_dCDP) |
| 138 | 1,00 | 2 | false | 2 | (1 PurM_pyruvate-phosphotransferase_dGTP) (-1 TCA_PEP-carboxylase) |
| 139 | 0,00 | 1 | false | 1 | (1 PurM_5-nucleotidase_dTMP) |
| 140 | 0,00 | 4 | false | 4 | (1 PurM_D-Ribose-1,5-phosphomutase) (-1 PurM_IMP-pyrophosphorylase) (1 PurM_nucleotide-phosphatase_Inosine) (1 PurM_PRPP-synthetase) |
| 141 | 0,00 | 1 | false | 1 | (1 PyrM_uridine-kinase_ATP) |
| 142 | 1,00 | 1 | false | 1 | (1 PurM_DNA-directed-RNA-polyermase_GTP) |
| 143 | 1,00 | 7 | false | 6 | (-1 Glyc_acetaldehyde-dehydrogenase_NAD+) (-1 Glyc_Actetate-CoA-ligase) (2 Glyc_dihydrolipoamide-dehydrogenase) (-1 SERP0389-Glyc_Ethanol_NAD+-oxidoreductase) (-1 TCA_citrate-hydro-lyase) (1 TCA_citrate_synthase) |
| 144 | 1,00 | 1 | false | 1 | (1 PyrM_OMP-decarboxylase) |
| 145 | 1,00 | 1 | false | 1 | (1 AS_Glutamate_to_Glutamine) |
| 146 | 1,00 | 1 | false | 1 | (1 PurM_urea-amidohydrolase) |
| 147 | 1,00 | 4 | false | 3 | (1 AMP-energy_to_AMP-metabolism) (1 PurM_adenylate-kinase_AMP) (2 PurM_thioredoxin-oxidoreductase_dADP) |
| 148 | 0,57 | 1 | false | 1 | (1 AS_Aspartate_to_Asparagine) |
| 149 | 0,27 | 1 | false | 1 | (1 PyrM_cytidine-kinase_ATP) |
| 150 | 0,70 | 2 | false | 2 | (1 PurM_GDP-reductase) (1 SERP0765-Uracil-permease-transport_import) |
| 151 | 0,72 | 3 | false | 3 | (1 IQ-143-extern_to_IQ-) (1 SERP0765-Uracil-permease-transport_import) (1 SERP1944-MultiDrug-transport_efflux) |
| 152 | 1,00 | 2 | false | 2 | (1 SERP0765-Uracil-permease-transport_import) (1 SERP1997-formate/nitrite-transport_efflux/import) |
| 153 | 1,00 | 13 | false | 9 | (-1 Glyc_acetaldehyde-dehydrogenase_NAD+) (-1 Glyc_Actetate-CoA-ligase) (-1 SERP0389-Glyc_Ethanol_NAD+-oxidoreductase) (-1 TCA_citrate-hydro-lyase) (1 TCA_citrate_synthase) (-2 TCA_lipoic-transsuccinylase) (2 TCA_Oxidoreductase) (2 TCA_oxoglutarate-dehydrogenase-complex1) (2 TCA_oxoglutarate-dehydrogenase-complex2) |
| 154 | 0,54 | 1 | false | 1 | (1 PurM_XTP-diphosphohydrolase) |
| 155 | 0,43 | 3 | false | 3 | (-1 PurM_carbamate-kinase_ATP) (1 PyrM_CO2_L-glutamine-amido-ligase) (-1 TCA_Pyruvate_CO2-ligase) |
| 156 | 1,00 | 12 | false | 7 | (2 Glyc_acetaldehyde-dehydrogenase_NAD+) (2 Glyc_Actetate-CoA-ligase) (1 OP_complex1) (1 OP_complex3) (2 SERP0389-Glyc_Ethanol_NAD+-oxidoreductase) (2 TCA_citrate-hydro-lyase) (-2 TCA_citrate_synthase) |
| 157 | 1,00 | 17 | false | 7 | (3 Glyc_acetaldehyde-dehydrogenase_NAD+) (3 Glyc_Actetate-CoA-ligase) (1 OP_complex2) (1 OP_complex3) (3 SERP0389-Glyc_Ethanol_NAD+-oxidoreductase) (3 TCA_citrate-hydro-lyase) (-3 TCA_citrate_synthase) |
| 158 | 1,00 | 1 | false | 1 | (1 PurM_GTP-pyrophosphokinase) |
| 159 | 1,00 | 6 | false | 6 | (1 Glyc_acetaldehyde-dehydrogenase_NAD+) (1 Glyc_Actetate-CoA-ligase) (1 OP_complex4) (1 SERP0389-Glyc_Ethanol_NAD+-oxidoreductase) (1 TCA_citrate-hydro-lyase) (-1 TCA_citrate_synthase) |
| 160 | 1,00 | 2 | false | 2 | (1 Glyc_pyruvate_dehydrogenase) (1 TCA_pyruvate_dehydrogenase) |
| 161 | 0,21 | 17 | false | 6 | (-3 Glyc_acetaldehyde-dehydrogenase_NAD+) (-3 Glyc_Actetate-CoA-ligase) (2 OP_complex5) (-3 SERP0389-Glyc_Ethanol_NAD+-oxidoreductase) (-3 TCA_citrate-hydro-lyase) (3 TCA_citrate_synthase) |
| 162 | 1,00 | 1 | false | 1 | (1 AS_Aspartate_to_Alanine) |
| 163 | 1,00 | 2 | false | 2 | (1 AS_Serine_to_Cysteine) (1 Glyc_Actetate-CoA-ligase) |
| 164 | 1,00 | 6 | false | 4 | (1 AMP-energy_to_AMP-metabolism) (1 PurM_adenylate-kinase_AMP) (2 PurM_nucleoside-diphosphate-phosphotransferase_ATP) (2 PurM_thioredoxin-oxidoreductase_dATP) |
| 165 | 1,00 | 1 | false | 1 | (1 SERP2186-PurM_ATP_sulfate-adenylyltransferase) |
| 166 | 0,25 | 3 | false | 3 | (-1 AMP-energy_to_AMP-metabolism) (1 PurM_ADP-ribose-ribophosphohydrolase) (1 PurM_PRPP-synthetase) |
| 167 | 1,10 | 2 | false | 2 | (1 IQ-143-extern_to_IQ-) (1 SERP1403-MultiDrug-transport_efflux) |
| 168 | 1,00 | 13 | false | 3 | (1 FA_Syn_Acetyl-CoA_to_C16) (-6 TCA_citrate-hydro-lyase) (6 TCA_citrate_synthase) |
| 169 | 0,25 | 1 | false | 1 | (1 PyrM_UTP_L-glutamine-amido-ligase) |
| 170 | 1,00 | 1 | false | 1 | (1 AS_Phenylalanin_to_Tyrosine) |
| 171 | 1,00 | 1 | false | 1 | (1 PurM_5-nucleotidase_IMP) |
| 172 | 0,00 | 6 | false | 4 | (1 AMP-energy_to_AMP-metabolism) (1 PurM_adenylate-kinase_AMP) (2 PurM_DNA-directed-RNA-polyermase_ATP) (2 PurM_nucleoside-diphosphate-phosphotransferase_ATP) |
| 173 | 0,50 | 2 | false | 2 | (1 PurM_GDP-reductase) (1 PurM_IMP-dehydrogenase) |
| 174 | 0,40 | 3 | false | 3 | (1 IQ-143-extern_to_IQ-) (1 PurM_IMP-dehydrogenase) (1 SERP1944-MultiDrug-transport_efflux) |
| 175 | 0,25 | 2 | false | 2 | (1 PurM_IMP-dehydrogenase) (1 SERP1997-formate/nitrite-transport_efflux/import) |
| 176 | 1,00 | 1 | false | 1 | (1 PurM_5-nucleotidase_GMP) |
| 177 | 1,00 | 1 | false | 1 | (1 PyrM_cytidine-kinase_ITP) |
| 178 | 1,00 | 1 | false | 1 | (1 AS_Histidine_to_Glutamate) |
| 179 | 1,00 | 1 | false | 1 | (1 PyrM_cytidine-kinase_GTP) |
| 180 | 0,90 | 13 | false | 3 | (1 FA_Deg_C16_to_Acetyl-CoA) (6 TCA_citrate-hydro-lyase) (-6 TCA_citrate_synthase) |
| 181 | 1,00 | 3 | false | 3 | (1 PurM_nucleoside-diphosphate-phosphotransferase_ATP) (1 PurM_pyruvate-phosphotransferase_ATP) (-1 TCA_PEP-carboxylase) |
| 182 | 1,00 | 1 | false | 1 | (1 PyrM_cytidine-kinase_dUTP) |
| 183 | 0,23 | 3 | false | 2 | (2 PurM_nucleoside-diphosphate-phosphotransferase_ATP) (1 SERP0687-spermidine/putrescine-transport_import) |
| 184 | 0,21 | 1 | false | 1 | (1 PurM_thioredoxin-oxidoreductase_dUDP) |
| 185 | 1,00 | 1 | false | 1 | (1 PurM_dGTP-diphosphohydrolase) |
| 186 | 1,00 | 1 | false | 1 | (1 PurM_DNA-directed-RNA-polyermase_CTP) |
| 187 | 1,00 | 1 | false | 1 | (1 SERP0831-PurM_DNA-directed-DNA-polymerase_dGTP) |
| 188 | 1,00 | 1 | false | 1 | (1 PurM_thioredoxin-oxidoreductase_dCTP) |
| 189 | 1,00 | 1 | false | 1 | (1 AS_Tryptophan_to_Tryptamine) |
| 190 | 1,02 | 3 | false | 3 | (1 AS_Acetyl-CoA_to_L-Leucine) (-1 TCA_citrate-hydro-lyase) (1 TCA_citrate_synthase) |
| 191 | 1,00 | 1 | false | 1 | (1 AS_Homoserine_to_Threonine) |
| 192 | 1,00 | 1 | false | 1 | (1 PurM_5-nucleotidase_CMP) |
| 193 | 1,00 | 1 | false | 1 | (1 PyrM_uridine-kinase_dUTP) |
| 194 | 1,00 | 1 | false | 1 | (1 PyrM_uridine-kinase_ITP) |
| 195 | 1,00 | 1 | false | 1 | (1 PurM_XMP-ligase) |
| 196 | 0,55 | 1 | false | 1 | (1 PyrM_cytidine-kinase_dATP) |
| 197 | 1,00 | 1 | false | 1 | (1 SERP2060-glyerol-transport_import) |
| 198 | 1,00 | 1 | false | 1 | (1 PyrM_uridine-kinase_GTP) |

^1^ This data shows the elementary mode Analysis for *S. aureus* USA300 with 0.16µM IQ-143.

Table S9: Extreme Modes of *S. aureus* USA300 with 1.25µM IQ-143^1^:

| # | Activity | Flux sum | Reversible? | Pathlength | Reactions |
| --- | --- | --- | --- | --- | --- |
| 1 | 1,00 | 1 | true | 1 | (1 AS_Alanine_to_Pyruvate) |
| 2 | 1,00 | 1 | true | 1 | (1 PyrM_dCMP-aminohydrolase) |
| 3 | -0,66 | 1 | true | 1 | (1 PyrM_GMP-pyrophosphorylase) |
| 4 | 1,00 | 1 | true | 1 | (1 PurM_ATP_IDP-phosphotransferase) |
| 5 | 1,00 | 1 | true | 1 | (1 PyrM_cytidilate-kinase_dCMP) |
| 6 | 1,00 | 1 | true | 1 | (1 PyrM_dUMP-phosphotransferase) |
| 7 | 1,00 | 1 | true | 1 | (1 PurM_ATP_GMP-guanylate-kinase) |
| 8 | 1,00 | 1 | true | 1 | (1 PyrM_cytidine-aminohydrolase) |
| 9 | 1,00 | 1 | true | 1 | (1 PurM_ATP_UTP-phosphotransferase) |
| 10 | 1,00 | 1 | true | 1 | (1 PurM_ATP_GTP-phosphotransferase) |
| 11 | 1,00 | 2 | true | 2 | (-1 PurM_nucleotide-phosphatase_Deoxyguanosine) (1 PyrM_deoxyguanosine-phosphorylase) |
| 12 | 1,00 | 2 | true | 2 | (-1 PyrM_dihydroorotase) (1 PyrM_dihydroorotate-oxidase) |
| 13 | 1,00 | 1 | true | 1 | (1 PurM_ATP_dIDP-phosphotransferase) |
| 14 | 1,00 | 1 | true | 1 | (1 PyrM_thymidine-kinase_dTMP) |
| 15 | 1,00 | 1 | true | 1 | (1 PurM_xanthosine-phosphoribosyltransferase) |
| 16 | 1,00 | 1 | true | 1 | (1 PurM_ATP_dUDP-phosphotransferase) |
| 17 | 0,97 | 1 | true | 1 | (1 TCA_fumarate-hydratase) |
| 18 | 1,00 | 3 | true | 3 | (1 PurM_IMP-pyrophosphorylase) (-1 PurM_nucleotide-phosphatase_Inosine) (1 PyrM_uridine-phosphorylase) |
| 19 | 0,46 | 3 | true | 3 | (1 PurM_IMP-pyrophosphorylase) (1 PurM_nucleotide-phosphatase_Guanosine) (-1 PurM_nucleotide-phosphatase_Inosine) |
| 20 | 1,00 | 3 | true | 3 | (1 PurM_IMP-pyrophosphorylase) (-1 PurM_nucleotide-phosphatase_Inosine) (1 PurM_nucleotide-phosphatase_Xanthosine) |
| 21 | 1,00 | 3 | true | 3 | (1 PurM_IMP-pyrophosphorylase) (-1 PurM_nucleotide-phosphatase_Deoxyinosine) (1 PyrM_deoxyinosine-phosphorylase) |
| 22 | 1,00 | 1 | true | 1 | (1 AS_Serine_to_Glycine) |
| 23 | 1,00 | 1 | true | 1 | (1 SERP1951-lipoprotein-transport_efflux/import) |
| 24 | 1,00 | 1 | true | 1 | (1 PurM_GMP-pyrophosphorylase2) |
| 25 | -0,59 | 1 | true | 1 | (1 PyrM_nucleoside-phosphate-kinase_ATP) |
| 26 | 1,00 | 1 | true | 1 | (1 PyrM_ATP_dTDP_thymidylate-kinase) |
| 27 | 1,00 | 1 | true | 1 | (1 PurM_ATP_dTDP-phosphotransferase) |
| 28 | 1,00 | 1 | true | 1 | (1 PurM_ATP_GMP_guanylate-kinase) |
| 29 | 1,00 | 1 | true | 1 | (1 PyrM_nucleoside-phosphate-kinase_ATP2) |
| 30 | -1,33 | 2 | true | 2 | (-1 PurM_nucleotide-phosphatase_Deoxyuridine) (1 PyrM_deoxyuridine-phosphorylase) |
| 31 | 1,00 | 2 | true | 2 | (1 PyrM_deoxyuridine-phosphorylase) (-1 PyrM_thymidine-kinase_dUMP) |
| 32 | 1,00 | 1 | true | 1 | (1 PurM_ATP_dGDP-phosphotransferase) |
| 33 | 1,00 | 1 | true | 1 | (1 AS_Aspartate_to_Homoserine) |
| 34 | 1,00 | 1 | true | 1 | (1 PurM_ATP_dADP-phosphotransferase) |
| 35 | 0,97 | 2 | true | 2 | (1 TCA_citrate-hydro-lyase) (-1 TCA_citrate-hydroxymutase) |
| 36 | 1,00 | 3 | true | 3 | (1 Glyc_lipoic_acetyltransferase) (-1 TCA_citrate-hydro-lyase) (1 TCA_citrate_synthase) |
| 37 | 1,00 | 7 | true | 6 | (-1 Glyc_acetaldehyde-dehydrogenase_NAD+) (-1 Glyc_Actetate-CoA-ligase) (-1 SERP0389-Glyc_Ethanol_NAD+-oxidoreductase) (2 SERP2156-Glyc_L-lactate-dehydrogenase) (-1 TCA_citrate-hydro-lyase) (1 TCA_citrate_synthase) |
| 38 | 1,00 | 1 | true | 1 | (1 PurM_ATP_CDP-phosphotransferase) |
| 39 | 1,00 | 1 | true | 1 | (1 AS_Saccharopine_to_Lysine) |
| 40 | 1,00 | 1 | true | 1 | (1 PurM_IMP_L-aspartate-ligase) |
| 41 | 1,00 | 1 | true | 1 | (1 Glyc_Succinate-CoA-ligase) |
| 42 | 1,00 | 3 | true | 3 | (1 AS_Acetyl-CoA_to_L-Valine) (-1 TCA_citrate-hydro-lyase) (1 TCA_citrate_synthase) |
| 43 | 1,00 | 1 | true | 1 | (1 PyrM_orotate-phosphoribosyltransferase) |
| 44 | 1,00 | 1 | true | 1 | (1 PurM_metaphosphatase) |
| 45 | 1,00 | 1 | true | 1 | (1 PyrM_cytidilate-kinase_CTP) |
| 46 | 1,00 | 1 | true | 1 | (1 Glyc_PTS-permease2) |
| 47 | 1,00 | 1 | true | 1 | (1 PyrM_thymidine-phosphorylase) |
| 48 | 1,00 | 1 | true | 1 | (1 PurM_ATP_dCDP-phosphotransferase) |
| 49 | -0,36 | 1 | true | 1 | (1 PyrM_UMP-pyrophosphorylase) |
| 50 | 0,40 | 1 | true | 1 | (1 PyrM_nucleoside-triphosphate-adenylate-kinase) |
| 51 | 0,40 | 1 | true | 1 | (1 PurM_XMP-pyrophosphorylase) |
| 52 | 0,40 | 2 | true | 2 | (1 PyrM_Deoxycytidine-aminohydrolase) (-1 PyrM_Deoxycytidine-deaminase) |
| 53 | 0,40 | 2 | true | 2 | (-1 PurM_nucleotide-phosphatase_Deoxyadenosine) (1 PyrM_deoxyadenosine-phosphorylase) |
| 54 | 0,40 | 1 | true | 1 | (1 PyrM_ATP_dUDP_thymidylate-kinase) |
| 55 | 0,95 | 1 | true | 1 | (1 PurM_adenylate-kinase_dAMP) |
| 56 | 0,92 | 2 | true | 2 | (1 AMP-energy_to_AMP-metabolism) (1 PurM_AMP-pyrophosphorylase2) |
| 57 | 1,08 | 2 | true | 2 | (1 AMP-energy_to_AMP-metabolism) (1 PyrM_AMP-pyrophosphorylase) |
| 58 | -0,50 | 10 | true | 6 | (1 AMP-energy_to_AMP-metabolism) (2 Glyc_alpha-D-Glucose-6-phosphate-ketol-isomerase) (2 Glyc_ATP-alpha-D-glucokinase) (-2 Glyc_ATP-beta-D-glucokinase) (-2 Glyc_D-Glucose-1-epimerase) (1 PurM_adenylate-kinase_AMP) |
| 59 | -0,64 | 6 | true | 4 | (-1 AMP-energy_to_AMP-metabolism) (2 ATP-energy_to_ATP-metabolism) (-1 PurM_adenylate-kinase_AMP) (-2 PurM_nucleoside-diphosphate-phosphotransferase_ATP) |
| 60 | 0,36 | 2 | true | 2 | (-1 AMP-energy_to_AMP-metabolism) (1 PurM_adenylosuccinate-lyase) |
| 61 | 0,92 | 12 | true | 7 | (-1 AMP-energy_to_AMP-metabolism) (-2 Glyc_alpha-D-Glucose-6-phosphate-ketol-isomerase2) (-2 Glyc_ATP-alpha-D-glucokinase) (2 Glyc_ATP-beta-D-glucokinase) (2 Glyc_beta-D-Glucose-6-phosphate-ketol-isomerase) (2 Glyc_D-Glucose-1-epimerase) (-1 PurM_adenylate-kinase_AMP) |
| 62 | -0,48 | 12 | true | 7 | (-1 AMP-energy_to_AMP-metabolism) (-2 Glyc_6-phospho-beta-glucosidase) (-2 Glyc_ATP-alpha-D-glucokinase) (2 Glyc_ATP-beta-D-glucokinase) (2 Glyc_D-Glucose-1-epimerase) (2 Glyc_PTS-permease1) (-1 PurM_adenylate-kinase_AMP) |
| 63 | 1,00 | 1 | true | 1 | (1 PurM_adenylylsulfate-kinase) |
| 64 | 1,00 | 4 | true | 3 | (2 ADP-energy_to_ADP-metabolism) (-1 AMP-energy_to_AMP-metabolism) (-1 PurM_adenylate-kinase_AMP) |
| 65 | 1,00 | 3 | true | 3 | (1 PurM_IMP-pyrophosphorylase) (1 PurM_nucleotide-phosphatase_Adenine) (-1 PurM_nucleotide-phosphatase_Inosine) |
| 66 | 1,00 | 3 | true | 3 | (1 PurM_IMP-pyrophosphorylase) (-1 PurM_nucleotide-phosphatase_Inosine) (1 PyrM_pyrimidine-nucleoside-phosphorylase) |
| 67 | 1,00 | 1 | true | 1 | (1 TCA_isocitrate-hydro-lyase) |
| 68 | 1,00 | 2 | false | 2 | (1 PurM_ATP-phosphohydrolase) (1 PurM_nucleoside-diphosphate-phosphotransferase_ATP) |
| 69 | 1,00 | 2 | false | 2 | (1 AMP-energy_to_AMP-metabolism) (1 PurM_5-nucleotidase_AMP) |
| 70 | 1,00 | 1 | false | 1 | (1 PyrM_uridine-kinase_dGTP) |
| 71 | 1,00 | 4 | false | 3 | (1 AMP-energy_to_AMP-metabolism) (1 PurM_adenylate-kinase_AMP) (2 SERP0841-PurM_PNPase_ADP) |
| 72 | 1,00 | 1 | false | 1 | (1 PurM_5-nucleotidase_XMP) |
| 73 | 0,39 | 1 | false | 1 | (1 SERP0831-PurM_DNA-directed-DNA-polymerase_dATP) |
| 74 | 1,00 | 1 | false | 1 | (1 PyrM_uridine-kinase_dTTP) |
| 75 | 1,00 | 1 | false | 1 | (1 PyrM_cytidine-kinase_dCTP) |
| 76 | 2,30 | 1 | false | 1 | (1 SERP1952-macrolide-transport_efflux) |
| 77 | 1,00 | 2 | false | 2 | (1 PurM_deoxyadenosine-kinase_ATP) (-1 PyrM_deoxyadenosine-phosphorylase) |
| 78 | 0,27 | 1 | false | 1 | (1 PyrM_dUTP-diphosphatase) |
| 79 | 1,00 | 1 | false | 1 | (1 AS_Aspartate_to_beta-Alanine) |
| 80 | 1,00 | 1 | false | 1 | (1 SERP1803-cobalt/nickel-transport_efflux) |
| 81 | 1,08 | 1 | false | 1 | (1 PyrM_cytidine-kinase_dGTP) |
| 82 | 1,00 | 1 | false | 1 | (1 PurM_thioredoxin-oxidoreductase_dUTP) |
| 83 | 0,43 | 1 | false | 1 | (1 SERP0292-iron-dicitrate-transporter_import) |
| 84 | 0,22 | 2 | false | 2 | (1 Glyc_6-phosphofructokinase) (1 Glyc_fructose-bisphosphatase) |
| 85 | 1,00 | 1 | false | 1 | (1 PurM_XMP_L-glutamine-amide-ligase) |
| 86 | 1,00 | 1 | false | 1 | (1 AS_Aspartate_to_Arginine) |
| 87 | 1,00 | 48 | false | 20 | (1 AMP-energy_to_AMP-metabolism) (-4 Glyc_2-Phospho-D-glycerate-2.3-phosphomutase) (4 Glyc_2-phospho-D-glycerate-hydro-lyase) (2 Glyc_6-phospho-beta-glucosidase) (2 Glyc_6-phosphofructokinase) (-2 Glyc_acetaldehyde-dehydrogenase_NAD+) (-2 Glyc_Actetate-CoA-ligase) (2 Glyc_alpha-D-Glucose-6-phosphate-ketol-isomerase2) (2 Glyc_ATP-alpha-D-glucokinase) (-2 Glyc_ATP-beta-D-glucokinase) (-2 Glyc_D-Glucose-1-epimerase) (-2 Glyc_D-Glucose-1-epimerase-ketol-isomerase) (2 Glyc_fructose-bisphosphat-aldolase) (4 Glyc_glyceraldehyde-3-P-dehydrogenase_NAD+) (-4 Glyc_phosphoglycerate-kinase) (1 PurM_adenylate-kinase_AMP) (-2 SERP0389-Glyc_Ethanol_NAD+-oxidoreductase) (-2 TCA_citrate-hydro-lyase) (2 TCA_citrate_synthase) (-4 TCA_PEP-carboxylase) |
| 88 | 1,00 | 7 | false | 6 | (1 Glyc_acetaldehyde-dehydrogenase_NAD+) (1 Glyc_Actetate-CoA-ligase) (2 PyrM_thioredoxin-reductase) (1 SERP0389-Glyc_Ethanol_NAD+-oxidoreductase) (1 TCA_citrate-hydro-lyase) (-1 TCA_citrate_synthase) |
| 89 | 1,00 | 1 | false | 1 | (1 PyrM_2,3-cyclic-nucleotidase_UMP) |
| 90 | 1,04 | 1 | false | 1 | (1 DNA-extern_to_DNA-intern) |
| 91 | 1,00 | 1 | false | 1 | (1 SERP0831-PurM_DNA-directed-DNA-polymerase_dCTP) |
| 92 | 0,67 | 1 | false | 1 | (1 SERP0841-PurM_PNPase_GDP) |
| 93 | 0,02 | 2 | false | 2 | (1 PurM_carbamate-kinase_ATP) (1 PyrM_aspartate-carbamoyltransferase) |
| 94 | 0,39 | 1 | false | 1 | (1 PurM_thioredoxin-oxidoreductase_dGDP) |
| 95 | 1,00 | 1 | false | 1 | (1 PurM_ITP-diphosphohydrolase) |
| 96 | 0,49 | 3 | false | 2 | (2 PurM_nucleoside-diphosphate-phosphotransferase_ATP) (1 SERP0688-spermidine/putrescine-transport_import) |
| 97 | 1,52 | 3 | false | 3 | (1 PurM_5-nucleotidase_dCMP) (1 PyrM_Deoxycytidine-aminohydrolase) (1 PyrM_deoxyuridine-phosphorylase) |
| 98 | 1,00 | 2 | false | 2 | (1 PurM_nucleoside-diphosphate-phosphotransferase_ATP) (1 SERP0686-spermidine/putrescine-transport_import) |
| 99 | 2,68 | 1 | false | 1 | (1 PyrM_dUTP-diphosphohydrolase) |
| 100 | 0,47 | 1 | false | 1 | (1 PurM_dITP-diphosphohydrolase) |
| 101 | 1,00 | 2 | false | 2 | (1 PurM_pyruvate-phosphotransferase_GTP) (-1 TCA_PEP-carboxylase) |
| 102 | 0,16 | 2 | false | 2 | (1 PurM_pyruvate-phosphotransferase_dATP) (-1 TCA_PEP-carboxylase) |
| 103 | 2,40 | 1 | false | 1 | (1 PurM_5-nucleotidase_UMP) |
| 104 | 1,00 | 1 | false | 1 | (1 SERP2283-phopsphonate-transport_import) |
| 105 | 1,00 | 2 | false | 2 | (1 PurM_GDP-reductase) (1 SERP2179-choline/betaine/carnitine-transp_efflux) |
| 106 | 1,00 | 1 | false | 1 | (1 AS_Valine) |
| 107 | 1,41 | 2 | false | 2 | (1 PurM_5-nucleotidase_dAMP) (1 PyrM_deoxyadenosine-phosphorylase) |
| 108 | 1,00 | 1 | false | 1 | (1 SERP1802-cobalt/nickel-transport_efflux) |
| 109 | 0,92 | 1 | false | 1 | (1 PurM_DNA-directed-RNA-polyermase_UTP) |
| 110 | 1,00 | 4 | false | 4 | (-1 TCA_lipoic-transsuccinylase) (1 TCA_oxoglutarate-dehydrogenase-complex1) (1 TCA_oxoglutarate-dehydrogenase-complex2) (1 TCA_oxoglutarate-synthase) |
| 111 | 1,00 | 1 | false | 1 | (1 PyrM_cytidine-kinase_dTTP) |
| 112 | 2,87 | 1 | false | 1 | (1 PurM_UTP-diphosphohydrolase) |
| 113 | 1,00 | 1 | false | 1 | (1 AS_Serine_to_Methionine) |
| 114 | 1,00 | 2 | false | 2 | (-1 AMP-energy_to_AMP-metabolism) (1 PurM_deoxycytidine-kinase_ATP) |
| 115 | 1,00 | 2 | false | 2 | (1 PurM_5-nucleotidase_dGMP) (1 PyrM_deoxyguanosine-phosphorylase) |
| 116 | 0,36 | 1 | false | 1 | (1 PurM_thioredoxin-oxidoreductase_dGTP) |
| 117 | 0,83 | 3 | false | 3 | (1 IQ-143-extern_to_IQ-) (1 SERP1944-MultiDrug-transport_efflux) (1 SERP2179-choline/betaine/carnitine-transp_efflux) |
| 118 | 1,00 | 1 | false | 1 | (1 PyrM_cytidine-kinase) |
| 119 | 0,54 | 1 | false | 1 | (1 AS_Leucine) |
| 120 | 1,01 | 48 | false | 20 | (1 AMP-energy_to_AMP-metabolism) (-4 Glyc_2-Phospho-D-glycerate-2.3-phosphomutase) (4 Glyc_2-phospho-D-glycerate-hydro-lyase) (2 Glyc_6-phospho-beta-glucosidase) (2 Glyc_6-phosphofructokinase) (-2 Glyc_acetaldehyde-dehydrogenase_NAD+) (-2 Glyc_Actetate-CoA-ligase) (2 Glyc_alpha-D-Glucose-6-phosphate-ketol-isomerase2) (2 Glyc_ATP-alpha-D-glucokinase) (-2 Glyc_ATP-beta-D-glucokinase) (-2 Glyc_D-Glucose-1-epimerase) (-2 Glyc_D-Glucose-1-epimerase-ketol-isomerase) (2 Glyc_fructose-bisphosphat-aldolase) (4 Glyc_glyceraldehyde-3-P-dehydrogenase_NADP+) (-4 Glyc_phosphoglycerate-kinase) (1 PurM_adenylate-kinase_AMP) (-2 SERP0389-Glyc_Ethanol_NAD+-oxidoreductase) (-2 TCA_citrate-hydro-lyase) (2 TCA_citrate_synthase) (-4 TCA_PEP-carboxylase) |
| 121 | 0,28 | 1 | false | 1 | (1 PyrM_2,3-cyclic-nucleotidase_CMP) |
| 122 | 1,97 | 2 | false | 2 | (1 SERP1997-formate/nitrite-transport_efflux/import) (1 SERP2179-choline/betaine/carnitine-transp_efflux) |
| 123 | 1,00 | 1 | false | 1 | (1 PyrM_CTP-synthase) |
| 124 | 0,80 | 2 | false | 2 | (1 IQ-143-extern_to_IQ-) (1 SERP2289-MultiDrug-transport_efflux) |
| 125 | 1,00 | 1 | false | 1 | (1 AS_Glutamate_to_Proline) |
| 126 | 0,72 | 1 | false | 1 | (1 PyrM_uridine-kinase_dCTP) |
| 127 | 1,50 | 1 | false | 1 | (1 PyrM_uridine-kinase_UTP) |
| 128 | 1,00 | 3 | false | 3 | (1 PurM_deoxycytidine-kinase_ATP2) (-1 PyrM_Deoxycytidine-aminohydrolase) (-1 PyrM_deoxyuridine-phosphorylase) |
| 129 | 1,00 | 1 | false | 1 | (1 AS_Threonine) |
| 130 | 1,40 | 1 | false | 1 | (1 PurM_allantoinase) |
| 131 | 1,00 | 1 | false | 1 | (1 SERP0831-PurM_DNA-directed-DNA-polymerase_dTTP) |
| 132 | 1,00 | 1 | false | 1 | (1 PurM_GTP-diphosphohydrolase) |
| 133 | 0,45 | 1 | false | 1 | (1 PyrM_uridine-kinase_dATP) |
| 134 | 1,00 | 1 | false | 1 | (1 AS_Serine_to_Pyruvate) |
| 135 | 1,00 | 2 | false | 2 | (1 SERP0290-zinc-transport_efflux) (1 SERP0291-zinc-transporter_import) |
| 136 | 1,00 | 1 | false | 1 | (1 AS_Isoleucine) |
| 137 | 0,85 | 1 | false | 1 | (1 PurM_thioredoxin-oxidoreductase_dCDP) |
| 138 | 1,00 | 2 | false | 2 | (1 PurM_pyruvate-phosphotransferase_dGTP) (-1 TCA_PEP-carboxylase) |
| 139 | 0,59 | 1 | false | 1 | (1 PurM_5-nucleotidase_dTMP) |
| 140 | 0,96 | 4 | false | 4 | (1 PurM_D-Ribose-1,5-phosphomutase) (-1 PurM_IMP-pyrophosphorylase) (1 PurM_nucleotide-phosphatase_Inosine) (1 PurM_PRPP-synthetase) |
| 141 | 1,14 | 1 | false | 1 | (1 PyrM_uridine-kinase_ATP) |
| 142 | 1,00 | 1 | false | 1 | (1 PurM_DNA-directed-RNA-polyermase_GTP) |
| 143 | 2,20 | 7 | false | 6 | (-1 Glyc_acetaldehyde-dehydrogenase_NAD+) (-1 Glyc_Actetate-CoA-ligase) (2 Glyc_dihydrolipoamide-dehydrogenase) (-1 SERP0389-Glyc_Ethanol_NAD+-oxidoreductase) (-1 TCA_citrate-hydro-lyase) (1 TCA_citrate_synthase) |
| 144 | 1,00 | 1 | false | 1 | (1 PyrM_OMP-decarboxylase) |
| 145 | 2,20 | 1 | false | 1 | (1 AS_Glutamate_to_Glutamine) |
| 146 | 2,20 | 1 | false | 1 | (1 PurM_urea-amidohydrolase) |
| 147 | 2,20 | 4 | false | 3 | (1 AMP-energy_to_AMP-metabolism) (1 PurM_adenylate-kinase_AMP) (2 PurM_thioredoxin-oxidoreductase_dADP) |
| 148 | 2,66 | 1 | false | 1 | (1 AS_Aspartate_to_Asparagine) |
| 149 | 1,27 | 1 | false | 1 | (1 PyrM_cytidine-kinase_ATP) |
| 150 | 0,00 | 2 | false | 2 | (1 PurM_GDP-reductase) (1 SERP0765-Uracil-permease-transport_import) |
| 151 | 1,55 | 3 | false | 3 | (1 IQ-143-extern_to_IQ-) (1 SERP0765-Uracil-permease-transport_import) (1 SERP1944-MultiDrug-transport_efflux) |
| 152 | 1,00 | 2 | false | 2 | (1 SERP0765-Uracil-permease-transport_import) (1 SERP1997-formate/nitrite-transport_efflux/import) |
| 153 | 1,00 | 13 | false | 9 | (-1 Glyc_acetaldehyde-dehydrogenase_NAD+) (-1 Glyc_Actetate-CoA-ligase) (-1 SERP0389-Glyc_Ethanol_NAD+-oxidoreductase) (-1 TCA_citrate-hydro-lyase) (1 TCA_citrate_synthase) (-2 TCA_lipoic-transsuccinylase) (2 TCA_Oxidoreductase) (2 TCA_oxoglutarate-dehydrogenase-complex1) (2 TCA_oxoglutarate-dehydrogenase-complex2) |
| 154 | 0,00 | 1 | false | 1 | (1 PurM_XTP-diphosphohydrolase) |
| 155 | 0,15 | 3 | false | 3 | (-1 PurM_carbamate-kinase_ATP) (1 PyrM_CO2_L-glutamine-amido-ligase) (-1 TCA_Pyruvate_CO2-ligase) |
| 156 | 1,00 | 12 | false | 7 | (2 Glyc_acetaldehyde-dehydrogenase_NAD+) (2 Glyc_Actetate-CoA-ligase) (1 OP_complex1) (1 OP_complex3) (2 SERP0389-Glyc_Ethanol_NAD+-oxidoreductase) (2 TCA_citrate-hydro-lyase) (-2 TCA_citrate_synthase) |
| 157 | 1,00 | 17 | false | 7 | (3 Glyc_acetaldehyde-dehydrogenase_NAD+) (3 Glyc_Actetate-CoA-ligase) (1 OP_complex2) (1 OP_complex3) (3 SERP0389-Glyc_Ethanol_NAD+-oxidoreductase) (3 TCA_citrate-hydro-lyase) (-3 TCA_citrate_synthase) |
| 158 | 2,06 | 1 | false | 1 | (1 PurM_GTP-pyrophosphokinase) |
| 159 | 1,00 | 6 | false | 6 | (1 Glyc_acetaldehyde-dehydrogenase_NAD+) (1 Glyc_Actetate-CoA-ligase) (1 OP_complex4) (1 SERP0389-Glyc_Ethanol_NAD+-oxidoreductase) (1 TCA_citrate-hydro-lyase) (-1 TCA_citrate_synthase) |
| 160 | 1,00 | 2 | false | 2 | (1 Glyc_pyruvate_dehydrogenase) (1 TCA_pyruvate_dehydrogenase) |
| 161 | 0,05 | 17 | false | 6 | (-3 Glyc_acetaldehyde-dehydrogenase_NAD+) (-3 Glyc_Actetate-CoA-ligase) (2 OP_complex5) (-3 SERP0389-Glyc_Ethanol_NAD+-oxidoreductase) (-3 TCA_citrate-hydro-lyase) (3 TCA_citrate_synthase) |
| 162 | 1,00 | 1 | false | 1 | (1 AS_Aspartate_to_Alanine) |
| 163 | 1,00 | 2 | false | 2 | (1 AS_Serine_to_Cysteine) (1 Glyc_Actetate-CoA-ligase) |
| 164 | 1,00 | 6 | false | 4 | (1 AMP-energy_to_AMP-metabolism) (1 PurM_adenylate-kinase_AMP) (2 PurM_nucleoside-diphosphate-phosphotransferase_ATP) (2 PurM_thioredoxin-oxidoreductase_dATP) |
| 165 | 1,00 | 1 | false | 1 | (1 SERP2186-PurM_ATP_sulfate-adenylyltransferase) |
| 166 | 0,22 | 3 | false | 3 | (-1 AMP-energy_to_AMP-metabolism) (1 PurM_ADP-ribose-ribophosphohydrolase) (1 PurM_PRPP-synthetase) |
| 167 | 1,20 | 2 | false | 2 | (1 IQ-143-extern_to_IQ-) (1 SERP1403-MultiDrug-transport_efflux) |
| 168 | 1,00 | 13 | false | 3 | (1 FA_Syn_Acetyl-CoA_to_C16) (-6 TCA_citrate-hydro-lyase) (6 TCA_citrate_synthase) |
| 169 | 0,22 | 1 | false | 1 | (1 PyrM_UTP_L-glutamine-amido-ligase) |
| 170 | 1,00 | 1 | false | 1 | (1 AS_Phenylalanin_to_Tyrosine) |
| 171 | 1,00 | 1 | false | 1 | (1 PurM_5-nucleotidase_IMP) |
| 172 | 0,00 | 6 | false | 4 | (1 AMP-energy_to_AMP-metabolism) (1 PurM_adenylate-kinase_AMP) (2 PurM_DNA-directed-RNA-polyermase_ATP) (2 PurM_nucleoside-diphosphate-phosphotransferase_ATP) |
| 173 | 0,50 | 2 | false | 2 | (1 PurM_GDP-reductase) (1 PurM_IMP-dehydrogenase) |
| 174 | 0,48 | 3 | false | 3 | (1 IQ-143-extern_to_IQ-) (1 PurM_IMP-dehydrogenase) (1 SERP1944-MultiDrug-transport_efflux) |
| 175 | 0,28 | 2 | false | 2 | (1 PurM_IMP-dehydrogenase) (1 SERP1997-formate/nitrite-transport_efflux/import) |
| 176 | 1,00 | 1 | false | 1 | (1 PurM_5-nucleotidase_GMP) |
| 177 | 1,00 | 1 | false | 1 | (1 PyrM_cytidine-kinase_ITP) |
| 178 | 1,00 | 1 | false | 1 | (1 AS_Histidine_to_Glutamate) |
| 179 | 1,00 | 1 | false | 1 | (1 PyrM_cytidine-kinase_GTP) |
| 180 | 0,80 | 13 | false | 3 | (1 FA_Deg_C16_to_Acetyl-CoA) (6 TCA_citrate-hydro-lyase) (-6 TCA_citrate_synthase) |
| 181 | 1,00 | 3 | false | 3 | (1 PurM_nucleoside-diphosphate-phosphotransferase_ATP) (1 PurM_pyruvate-phosphotransferase_ATP) (-1 TCA_PEP-carboxylase) |
| 182 | 1,00 | 1 | false | 1 | (1 PyrM_cytidine-kinase_dUTP) |
| 183 | 0,53 | 3 | false | 2 | (2 PurM_nucleoside-diphosphate-phosphotransferase_ATP) (1 SERP0687-spermidine/putrescine-transport_import) |
| 184 | 0,05 | 1 | false | 1 | (1 PurM_thioredoxin-oxidoreductase_dUDP) |
| 185 | 1,00 | 1 | false | 1 | (1 PurM_dGTP-diphosphohydrolase) |
| 186 | 1,00 | 1 | false | 1 | (1 PurM_DNA-directed-RNA-polyermase_CTP) |
| 187 | 1,00 | 1 | false | 1 | (1 SERP0831-PurM_DNA-directed-DNA-polymerase_dGTP) |
| 188 | 1,00 | 1 | false | 1 | (1 PurM_thioredoxin-oxidoreductase_dCTP) |
| 189 | 1,00 | 1 | false | 1 | (1 AS_Tryptophan_to_Tryptamine) |
| 190 | 1,03 | 3 | false | 3 | (1 AS_Acetyl-CoA_to_L-Leucine) (-1 TCA_citrate-hydro-lyase) (1 TCA_citrate_synthase) |
| 191 | 1,00 | 1 | false | 1 | (1 AS_Homoserine_to_Threonine) |
| 192 | 1,00 | 1 | false | 1 | (1 PurM_5-nucleotidase_CMP) |
| 193 | 0,35 | 1 | false | 1 | (1 PyrM_uridine-kinase_dUTP) |
| 194 | 1,00 | 1 | false | 1 | (1 PyrM_uridine-kinase_ITP) |
| 195 | 1,00 | 1 | false | 1 | (1 PurM_XMP-ligase) |
| 196 | 0,52 | 1 | false | 1 | (1 PyrM_cytidine-kinase_dATP) |
| 197 | 1,00 | 1 | false | 1 | (1 SERP2060-glyerol-transport_import) |
| 198 | 2,82 | 1 | false | 1 | (1 PyrM_uridine-kinase_GTP) |

^1^ This data shows the elementary mode Analysis for *S. aureus* USA300 with 1.25µM IQ-143.

Table S10: Extreme Modes of *S. epidermidis* RP62A without IQ-143^1^:

| # | Activity | Flux sum | Reversible? | Pathlength | Reactions |
| --- | --- | --- | --- | --- | --- |
| 1 | 1,00 | 1 | true | 1 | (1 AS_Alanine_to_Pyruvate) |
| 2 | 1,00 | 1 | true | 1 | (1 PyrM_dCMP-aminohydrolase) |
| 3 | 0,70 | 1 | true | 1 | (1 PurM_ATP_IDP-phosphotransferase) |
| 4 | 1,00 | 1 | true | 1 | (1 PyrM_cytidilate-kinase_dCMP) |
| 5 | 1,00 | 1 | true | 1 | (1 PyrM_dUMP-phosphotransferase) |
| 6 | 1,00 | 1 | true | 1 | (1 PurM_ATP_GMP-guanylate-kinase) |
| 7 | 1,00 | 1 | true | 1 | (1 PyrM_cytidine-aminohydrolase) |
| 8 | 1,00 | 1 | true | 1 | (1 PurM_ATP_UTP-phosphotransferase) |
| 9 | 1,00 | 1 | true | 1 | (1 PurM_ATP_GTP-phosphotransferase) |
| 10 | 1,00 | 2 | true | 2 | (-1 PurM_nucleotide-phosphatase_Deoxyguanosine) (1 PyrM_deoxyguanosine-phosphorylase) |
| 11 | 1,00 | 2 | true | 2 | (-1 PyrM_dihydroorotase) (1 PyrM_dihydroorotate-oxidase) |
| 12 | 1,00 | 1 | true | 1 | (1 PurM_ATP_dIDP-phosphotransferase) |
| 13 | 1,00 | 1 | true | 1 | (1 PyrM_thymidine-kinase_dTMP) |
| 14 | 1,00 | 1 | true | 1 | (1 PurM_xanthosine-phosphoribosyltransferase) |
| 15 | 1,00 | 1 | true | 1 | (1 PurM_ATP_dUDP-phosphotransferase) |
| 16 | 1,00 | 1 | true | 1 | (1 TCA_fumarate-hydratase) |
| 17 | 0,91 | 3 | true | 3 | (1 PurM_IMP-pyrophosphorylase) (-1 PurM_nucleotide-phosphatase_Inosine) (1 PyrM_uridine-phosphorylase) |
| 18 | 1,00 | 3 | true | 3 | (1 PurM_IMP-pyrophosphorylase) (1 PurM_nucleotide-phosphatase_Guanosine) (-1 PurM_nucleotide-phosphatase_Inosine) |
| 19 | 1,00 | 3 | true | 3 | (1 PurM_IMP-pyrophosphorylase) (-1 PurM_nucleotide-phosphatase_Inosine) (1 PurM_nucleotide-phosphatase_Xanthosine) |
| 20 | 1,00 | 3 | true | 3 | (1 PurM_IMP-pyrophosphorylase) (-1 PurM_nucleotide-phosphatase_Deoxyinosine) (1 PyrM_deoxyinosine-phosphorylase) |
| 21 | 1,00 | 1 | true | 1 | (1 AS_Serine_to_Glycine) |
| 22 | 1,00 | 1 | true | 1 | (1 SERP1951-lipoprotein-transport_efflux/import) |
| 23 | 1,00 | 1 | true | 1 | (1 PurM_GMP-pyrophosphorylase2) |
| 24 | 1,00 | 1 | true | 1 | (1 PyrM_nucleoside-phosphate-kinase_ATP) |
| 25 | -0,52 | 1 | true | 1 | (1 PyrM_ATP_dTDP_thymidylate-kinase) |
| 26 | 1,00 | 1 | true | 1 | (1 PurM_ATP_dTDP-phosphotransferase) |
| 27 | 1,00 | 1 | true | 1 | (1 PurM_ATP_GMP_guanylate-kinase) |
| 28 | 1,00 | 1 | true | 1 | (1 PyrM_nucleoside-phosphate-kinase_ATP2) |
| 29 | 1,00 | 2 | true | 2 | (-1 PurM_nucleotide-phosphatase_Deoxyuridine) (1 PyrM_deoxyuridine-phosphorylase) |
| 30 | -1,33 | 2 | true | 2 | (1 PyrM_deoxyuridine-phosphorylase) (-1 PyrM_thymidine-kinase_dUMP) |
| 31 | 1,00 | 1 | true | 1 | (1 PurM_ATP_dGDP-phosphotransferase) |
| 32 | 1,00 | 1 | true | 1 | (1 AS_Aspartate_to_Homoserine) |
| 33 | 1,00 | 1 | true | 1 | (1 PurM_ATP_dADP-phosphotransferase) |
| 34 | 1,00 | 2 | true | 2 | (1 TCA_citrate-hydro-lyase) (-1 TCA_citrate-hydroxymutase) |
| 35 | 0,91 | 3 | true | 3 | (1 Glyc_lipoic_acetyltransferase) (-1 TCA_citrate-hydro-lyase) (1 TCA_citrate_synthase) |
| 36 | -1,00 | 7 | true | 6 | (-1 Glyc_acetaldehyde-dehydrogenase_NAD+) (-1 Glyc_Actetate-CoA-ligase) (-1 SERP0389-Glyc_Ethanol_NAD+-oxidoreductase) (2 SERP2156-Glyc_L-lactate-dehydrogenase) (-1 TCA_citrate-hydro-lyase) (1 TCA_citrate_synthase) |
| 37 | 1,00 | 1 | true | 1 | (1 PurM_ATP_CDP-phosphotransferase) |
| 38 | 1,00 | 1 | true | 1 | (1 AS_Saccharopine_to_Lysine) |
| 39 | 1,00 | 1 | true | 1 | (1 PurM_IMP_L-aspartate-ligase) |
| 40 | 0,50 | 1 | true | 1 | (1 Glyc_Succinate-CoA-ligase) |
| 41 | 0,50 | 3 | true | 3 | (1 AS_Acetyl-CoA_to_L-Valine) (-1 TCA_citrate-hydro-lyase) (1 TCA_citrate_synthase) |
| 42 | 1,00 | 1 | true | 1 | (1 PyrM_orotate-phosphoribosyltransferase) |
| 43 | 1,00 | 1 | true | 1 | (1 PurM_metaphosphatase) |
| 44 | 1,05 | 1 | true | 1 | (1 PyrM_cytidilate-kinase_CTP) |
| 45 | 0,73 | 1 | true | 1 | (1 PurM_GMP-pyrophosphorylase) |
| 46 | 1,00 | 3 | true | 3 | (1 Glyc_alpha-D-Glucose-6-phosphate-ketol-isomerase) (-1 Glyc_alpha-D-Glucose-6-phosphate-ketol-isomerase2) (1 Glyc_beta-D-Glucose-6-phosphate-ketol-isomerase) |
| 47 | 1,00 | 6 | true | 6 | (1 ATP-energy_to_ATP-metabolism) (1 Glyc_alpha-D-Glucose-6-phosphate-ketol-isomerase2) (1 Glyc_ATP-alpha-D-glucokinase) (-1 Glyc_ATP-beta-D-glucokinase) (-1 Glyc_beta-D-Glucose-6-phosphate-ketol-isomerase) (-1 Glyc_D-Glucose-1-epimerase) |
| 48 | 0,75 | 1 | true | 1 | (1 Glyc_PTS-permease2) |
| 49 | 1,00 | 4 | true | 4 | (-1 Glyc_6-phospho-beta-glucosidase) (1 Glyc_alpha-D-Glucose-6-phosphate-ketol-isomerase2) (-1 Glyc_beta-D-Glucose-6-phosphate-ketol-isomerase) (1 Glyc_PTS-permease1) |
| 50 | 1,00 | 1 | true | 1 | (1 PyrM_thymidine-phosphorylase) |
| 51 | 1,00 | 1 | true | 1 | (1 PurM_ATP_dCDP-phosphotransferase) |
| 52 | -1,34 | 1 | true | 1 | (1 PyrM_UMP-pyrophosphorylase) |
| 53 | 0,79 | 1 | true | 1 | (1 PyrM_nucleoside-triphosphate-adenylate-kinase) |
| 54 | 0,53 | 1 | true | 1 | (1 PurM_XMP-pyrophosphorylase) |
| 55 | 0,53 | 1 | true | 1 | (1 TCA_succinate-dehydrogenase) |
| 56 | 0,53 | 2 | true | 2 | (1 PyrM_Deoxycytidine-aminohydrolase) (-1 PyrM_Deoxycytidine-deaminase) |
| 57 | 0,53 | 2 | true | 2 | (-1 PurM_nucleotide-phosphatase_Deoxyadenosine) (1 PyrM_deoxyadenosine-phosphorylase) |
| 58 | 0,53 | 1 | true | 1 | (1 PyrM_ATP_dUDP_thymidylate-kinase) |
| 59 | 0,92 | 1 | true | 1 | (1 PurM_adenylate-kinase_dAMP) |
| 60 | 1,08 | 2 | true | 2 | (1 AMP-energy_to_AMP-metabolism) (1 PurM_AMP-pyrophosphorylase2) |
| 61 | -0,65 | 2 | true | 2 | (-1 AMP-energy_to_AMP-metabolism) (1 PurM_adenylosuccinate-lyase) |
| 62 | 0,92 | 2 | true | 2 | (1 AMP-energy_to_AMP-metabolism) (1 PurM_AMP-pyrophosphorylase) |
| 63 | -0,48 | 14 | true | 8 | (1 AMP-energy_to_AMP-metabolism) (2 Glyc_alpha-D-Glucose-6-phosphate-ketol-isomerase2) (2 Glyc_ATP-alpha-D-glucokinase) (-2 Glyc_ATP-beta-D-glucokinase) (-2 Glyc_beta-D-Glucose-6-phosphate-ketol-isomerase) (-2 Glyc_D-Glucose-1-epimerase) (1 PurM_adenylate-kinase_AMP) (2 PurM_nucleoside-diphosphate-phosphotransferase_ATP) |
| 64 | 1,00 | 1 | true | 1 | (1 PurM_adenylylsulfate-kinase) |
| 65 | 1,00 | 4 | true | 3 | (2 ADP-energy_to_ADP-metabolism) (-1 AMP-energy_to_AMP-metabolism) (-1 PurM_adenylate-kinase_AMP) |
| 66 | 1,00 | 3 | true | 3 | (1 PurM_IMP-pyrophosphorylase) (1 PurM_nucleotide-phosphatase_Adenine) (-1 PurM_nucleotide-phosphatase_Inosine) |
| 67 | 1,00 | 3 | true | 3 | (1 PurM_IMP-pyrophosphorylase) (-1 PurM_nucleotide-phosphatase_Inosine) (1 PyrM_pyrimidine-nucleoside-phosphorylase) |
| 68 | 1,00 | 1 | true | 1 | (1 TCA_isocitrate-hydro-lyase) |
| 69 | 1,00 | 14 | false | 8 | (-1 AMP-energy_to_AMP-metabolism) (-2 Glyc_alpha-D-Glucose-6-phosphate-ketol-isomerase2) (-2 Glyc_ATP-alpha-D-glucokinase) (2 Glyc_ATP-beta-D-glucokinase) (2 Glyc_beta-D-Glucose-6-phosphate-ketol-isomerase) (2 Glyc_D-Glucose-1-epimerase) (-1 PurM_adenylate-kinase_AMP) (2 PurM_ATP-phosphohydrolase) |
| 70 | 1,00 | 2 | false | 2 | (1 AMP-energy_to_AMP-metabolism) (1 PurM_5-nucleotidase_AMP) |
| 71 | 1,00 | 1 | false | 1 | (1 PyrM_uridine-kinase_dGTP) |
| 72 | 1,00 | 4 | false | 3 | (1 AMP-energy_to_AMP-metabolism) (1 PurM_adenylate-kinase_AMP) (2 SERP0841-PurM_PNPase_ADP) |
| 73 | 1,00 | 1 | false | 1 | (1 PurM_5-nucleotidase_XMP) |
| 74 | 1,00 | 1 | false | 1 | (1 SERP0831-PurM_DNA-directed-DNA-polymerase_dATP) |
| 75 | 1,00 | 1 | false | 1 | (1 PyrM_uridine-kinase_dTTP) |
| 76 | 1,00 | 1 | false | 1 | (1 PyrM_cytidine-kinase_dCTP) |
| 77 | 1,00 | 1 | false | 1 | (1 SERP1952-macrolide-transport_efflux) |
| 78 | 1,00 | 2 | false | 2 | (1 PurM_deoxyadenosine-kinase_ATP) (-1 PyrM_deoxyadenosine-phosphorylase) |
| 79 | 0,55 | 1 | false | 1 | (1 PyrM_dUTP-diphosphatase) |
| 80 | 1,00 | 1 | false | 1 | (1 AS_Aspartate_to_beta-Alanine) |
| 81 | 1,00 | 1 | false | 1 | (1 SERP1803-cobalt/nickel-transport_efflux) |
| 82 | 1,00 | 1 | false | 1 | (1 PyrM_cytidine-kinase_dGTP) |
| 83 | 1,00 | 1 | false | 1 | (1 PurM_thioredoxin-oxidoreductase_dUTP) |
| 84 | 1,00 | 1 | false | 1 | (1 SERP0292-iron-dicitrate-transporter_import) |
| 85 | 0,25 | 2 | false | 2 | (1 Glyc_6-phosphofructokinase) (1 Glyc_fructose-bisphosphatase) |
| 86 | 1,00 | 1 | false | 1 | (1 PurM_XMP_L-glutamine-amide-ligase) |
| 87 | 1,00 | 1 | false | 1 | (1 AS_Aspartate_to_Arginine) |
| 88 | 1,00 | 7 | false | 6 | (1 Glyc_acetaldehyde-dehydrogenase_NAD+) (1 Glyc_Actetate-CoA-ligase) (2 PyrM_thioredoxin-reductase) (1 SERP0389-Glyc_Ethanol_NAD+-oxidoreductase) (1 TCA_citrate-hydro-lyase) (-1 TCA_citrate_synthase) |
| 89 | 1,00 | 20 | false | 15 | (-2 Glyc_2-Phospho-D-glycerate-2.3-phosphomutase) (2 Glyc_2-phospho-D-glycerate-hydro-lyase) (1 Glyc_6-phospho-beta-glucosidase) (1 Glyc_6-phosphofructokinase) (-1 Glyc_acetaldehyde-dehydrogenase_NAD+) (-1 Glyc_Actetate-CoA-ligase) (1 Glyc_beta-D-Glucose-6-phosphate-ketol-isomerase) (-1 Glyc_D-Glucose-1-epimerase-ketol-isomerase) (1 Glyc_fructose-bisphosphat-aldolase) (2 Glyc_glyceraldehyde-3-P-dehydrogenase_NAD+) (-2 Glyc_phosphoglycerate-kinase) (-1 SERP0389-Glyc_Ethanol_NAD+-oxidoreductase) (-1 TCA_citrate-hydro-lyase) (1 TCA_citrate_synthase) (-2 TCA_PEP-carboxylase) |
| 90 | 0,96 | 1 | false | 1 | (1 PyrM_2,3-cyclic-nucleotidase_UMP) |
| 91 | 1,00 | 1 | false | 1 | (1 DNA-extern_to_DNA-intern) |
| 92 | 0,67 | 1 | false | 1 | (1 SERP0831-PurM_DNA-directed-DNA-polymerase_dCTP) |
| 93 | 0,36 | 1 | false | 1 | (1 SERP0841-PurM_PNPase_GDP) |
| 94 | 0,41 | 2 | false | 2 | (1 PurM_carbamate-kinase_ATP) (1 PyrM_aspartate-carbamoyltransferase) |
| 95 | 1,00 | 1 | false | 1 | (1 PurM_thioredoxin-oxidoreductase_dGDP) |
| 96 | 0,30 | 1 | false | 1 | (1 PurM_ITP-diphosphohydrolase) |
| 97 | 0,35 | 13 | false | 8 | (-1 AMP-energy_to_AMP-metabolism) (-2 Glyc_alpha-D-Glucose-6-phosphate-ketol-isomerase2) (-2 Glyc_ATP-alpha-D-glucokinase) (2 Glyc_ATP-beta-D-glucokinase) (2 Glyc_beta-D-Glucose-6-phosphate-ketol-isomerase) (2 Glyc_D-Glucose-1-epimerase) (-1 PurM_adenylate-kinase_AMP) (1 SERP0688-spermidine/putrescine-transport_import) |
| 98 | 1,00 | 3 | false | 3 | (1 PurM_5-nucleotidase_dCMP) (1 PyrM_Deoxycytidine-aminohydrolase) (1 PyrM_deoxyuridine-phosphorylase) |
| 99 | 1,00 | 14 | false | 8 | (-1 AMP-energy_to_AMP-metabolism) (-2 Glyc_alpha-D-Glucose-6-phosphate-ketol-isomerase2) (-2 Glyc_ATP-alpha-D-glucokinase) (2 Glyc_ATP-beta-D-glucokinase) (2 Glyc_beta-D-Glucose-6-phosphate-ketol-isomerase) (2 Glyc_D-Glucose-1-epimerase) (-1 PurM_adenylate-kinase_AMP) (2 SERP0686-spermidine/putrescine-transport_import) |
| 100 | 0,36 | 1 | false | 1 | (1 PyrM_dUTP-diphosphohydrolase) |
| 101 | 1,00 | 1 | false | 1 | (1 PurM_dITP-diphosphohydrolase) |
| 102 | 0,35 | 2 | false | 2 | (1 PurM_pyruvate-phosphotransferase_GTP) (-1 TCA_PEP-carboxylase) |
| 103 | 1,00 | 2 | false | 2 | (1 PurM_pyruvate-phosphotransferase_dATP) (-1 TCA_PEP-carboxylase) |
| 104 | 1,00 | 1 | false | 1 | (1 PurM_5-nucleotidase_UMP) |
| 105 | 1,00 | 1 | false | 1 | (1 SERP2283-phopsphonate-transport_import) |
| 106 | 1,00 | 2 | false | 2 | (1 PurM_GDP-reductase) (1 SERP2179-choline/betaine/carnitine-transp_efflux) |
| 107 | 0,48 | 1 | false | 1 | (1 AS_Valine) |
| 108 | 1,00 | 2 | false | 2 | (1 PurM_5-nucleotidase_dAMP) (1 PyrM_deoxyadenosine-phosphorylase) |
| 109 | 0,92 | 1 | false | 1 | (1 SERP1802-cobalt/nickel-transport_efflux) |
| 110 | 1,00 | 1 | false | 1 | (1 PurM_DNA-directed-RNA-polyermase_UTP) |
| 111 | 1,00 | 4 | false | 4 | (-1 TCA_lipoic-transsuccinylase) (1 TCA_oxoglutarate-dehydrogenase-complex1) (1 TCA_oxoglutarate-dehydrogenase-complex2) (1 TCA_oxoglutarate-synthase) |
| 112 | 1,00 | 1 | false | 1 | (1 PyrM_cytidine-kinase_dTTP) |
| 113 | 1,00 | 1 | false | 1 | (1 PurM_UTP-diphosphohydrolase) |
| 114 | 1,00 | 1 | false | 1 | (1 AS_Serine_to_Methionine) |
| 115 | 1,00 | 2 | false | 2 | (1 PurM_5-nucleotidase_dGMP) (1 PyrM_deoxyguanosine-phosphorylase) |
| 116 | 0,19 | 2 | false | 2 | (-1 AMP-energy_to_AMP-metabolism) (1 PurM_deoxycytidine-kinase_ATP) |
| 117 | 0,19 | 1 | false | 1 | (1 PurM_thioredoxin-oxidoreductase_dGTP) |
| 118 | 1,00 | 3 | false | 3 | (1 IQ-143-extern_to_IQ-) (1 SERP1944-MultiDrug-transport_efflux) (1 SERP2179-choline/betaine/carnitine-transp_efflux) |
| 119 | 0,36 | 1 | false | 1 | (1 AS_Leucine) |
| 120 | 0,36 | 20 | false | 15 | (-2 Glyc_2-Phospho-D-glycerate-2.3-phosphomutase) (2 Glyc_2-phospho-D-glycerate-hydro-lyase) (1 Glyc_6-phospho-beta-glucosidase) (1 Glyc_6-phosphofructokinase) (-1 Glyc_acetaldehyde-dehydrogenase_NAD+) (-1 Glyc_Actetate-CoA-ligase) (1 Glyc_beta-D-Glucose-6-phosphate-ketol-isomerase) (-1 Glyc_D-Glucose-1-epimerase-ketol-isomerase) (1 Glyc_fructose-bisphosphat-aldolase) (2 Glyc_glyceraldehyde-3-P-dehydrogenase_NADP+) (-2 Glyc_phosphoglycerate-kinase) (-1 SERP0389-Glyc_Ethanol_NAD+-oxidoreductase) (-1 TCA_citrate-hydro-lyase) (1 TCA_citrate_synthase) (-2 TCA_PEP-carboxylase) |
| 121 | 0,25 | 1 | false | 1 | (1 PyrM_2,3-cyclic-nucleotidase_CMP) |
| 122 | 0,48 | 2 | false | 2 | (1 SERP1997-formate/nitrite-transport_efflux/import) (1 SERP2179-choline/betaine/carnitine-transp_efflux) |
| 123 | 1,00 | 1 | false | 1 | (1 PyrM_CTP-synthase) |
| 124 | 0,80 | 2 | false | 2 | (1 IQ-143-extern_to_IQ-) (1 SERP2289-MultiDrug-transport_efflux) |
| 125 | 1,00 | 1 | false | 1 | (1 AS_Glutamate_to_Proline) |
| 126 | 0,75 | 1 | false | 1 | (1 PyrM_uridine-kinase_dCTP) |
| 127 | 1,75 | 1 | false | 1 | (1 PyrM_uridine-kinase_UTP) |
| 128 | 1,00 | 3 | false | 3 | (1 PurM_deoxycytidine-kinase_ATP2) (-1 PyrM_Deoxycytidine-aminohydrolase) (-1 PyrM_deoxyuridine-phosphorylase) |
| 129 | 1,00 | 1 | false | 1 | (1 AS_Threonine) |
| 130 | 1,40 | 1 | false | 1 | (1 SERP0831-PurM_DNA-directed-DNA-polymerase_dTTP) |
| 131 | 1,00 | 1 | false | 1 | (1 PurM_GTP-diphosphohydrolase) |
| 132 | 1,00 | 1 | false | 1 | (1 PyrM_uridine-kinase_dATP) |
| 133 | 1,00 | 1 | false | 1 | (1 AS_Serine_to_Pyruvate) |
| 134 | 1,00 | 2 | false | 2 | (1 SERP0290-zinc-transport_efflux) (1 SERP0291-zinc-transporter_import) |
| 135 | 1,00 | 1 | false | 1 | (1 AS_Isoleucine) |
| 136 | 1,00 | 1 | false | 1 | (1 PurM_thioredoxin-oxidoreductase_dCDP) |
| 137 | 0,52 | 2 | false | 2 | (1 PurM_pyruvate-phosphotransferase_dGTP) (-1 TCA_PEP-carboxylase) |
| 138 | 1,00 | 1 | false | 1 | (1 PurM_5-nucleotidase_dTMP) |
| 139 | 0,36 | 1 | false | 1 | (1 PyrM_uridine-kinase_ATP) |
| 140 | 0,19 | 4 | false | 4 | (1 PurM_D-Ribose-1,5-phosphomutase) (-1 PurM_IMP-pyrophosphorylase) (1 PurM_nucleotide-phosphatase_Inosine) (1 PurM_PRPP-synthetase) |
| 141 | 0,36 | 1 | false | 1 | (1 PurM_DNA-directed-RNA-polyermase_GTP) |
| 142 | 1,00 | 7 | false | 6 | (-1 Glyc_acetaldehyde-dehydrogenase_NAD+) (-1 Glyc_Actetate-CoA-ligase) (2 Glyc_dihydrolipoamide-dehydrogenase) (-1 SERP0389-Glyc_Ethanol_NAD+-oxidoreductase) (-1 TCA_citrate-hydro-lyase) (1 TCA_citrate_synthase) |
| 143 | 1,00 | 1 | false | 1 | (1 PyrM_OMP-decarboxylase) |
| 144 | 1,00 | 1 | false | 1 | (1 AS_Glutamate_to_Glutamine) |
| 145 | 1,00 | 1 | false | 1 | (1 PurM_urea-amidohydrolase) |
| 146 | 1,00 | 1 | false | 1 | (1 AS_Aspartate_to_Asparagine) |
| 147 | 1,00 | 1 | false | 1 | (1 PyrM_cytidine-kinase_ATP) |
| 148 | 0,64 | 2 | false | 2 | (1 PurM_GDP-reductase) (1 SERP0765-Uracil-permease-transport_import) |
| 149 | 0,48 | 3 | false | 3 | (1 IQ-143-extern_to_IQ-) (1 SERP0765-Uracil-permease-transport_import) (1 SERP1944-MultiDrug-transport_efflux) |
| 150 | 0,91 | 2 | false | 2 | (1 SERP0765-Uracil-permease-transport_import) (1 SERP1997-formate/nitrite-transport_efflux/import) |
| 151 | 0,56 | 13 | false | 9 | (-1 Glyc_acetaldehyde-dehydrogenase_NAD+) (-1 Glyc_Actetate-CoA-ligase) (-1 SERP0389-Glyc_Ethanol_NAD+-oxidoreductase) (-1 TCA_citrate-hydro-lyase) (1 TCA_citrate_synthase) (-2 TCA_lipoic-transsuccinylase) (2 TCA_Oxidoreductase) (2 TCA_oxoglutarate-dehydrogenase-complex1) (2 TCA_oxoglutarate-dehydrogenase-complex2) |
| 152 | 1,00 | 1 | false | 1 | (1 PurM_XTP-diphosphohydrolase) |
| 153 | 1,00 | 3 | false | 3 | (-1 PurM_carbamate-kinase_ATP) (1 PyrM_CO2_L-glutamine-amido-ligase) (-1 TCA_Pyruvate_CO2-ligase) |
| 154 | 0,60 | 12 | false | 7 | (2 Glyc_acetaldehyde-dehydrogenase_NAD+) (2 Glyc_Actetate-CoA-ligase) (1 OP_complex1) (1 OP_complex3) (2 SERP0389-Glyc_Ethanol_NAD+-oxidoreductase) (2 TCA_citrate-hydro-lyase) (-2 TCA_citrate_synthase) |
| 155 | 0,48 | 17 | false | 7 | (3 Glyc_acetaldehyde-dehydrogenase_NAD+) (3 Glyc_Actetate-CoA-ligase) (1 OP_complex2) (1 OP_complex3) (3 SERP0389-Glyc_Ethanol_NAD+-oxidoreductase) (3 TCA_citrate-hydro-lyase) (-3 TCA_citrate_synthase) |
| 156 | 1,00 | 1 | false | 1 | (1 PurM_GTP-pyrophosphokinase) |
| 157 | 1,00 | 6 | false | 6 | (1 Glyc_acetaldehyde-dehydrogenase_NAD+) (1 Glyc_Actetate-CoA-ligase) (1 OP_complex4) (1 SERP0389-Glyc_Ethanol_NAD+-oxidoreductase) (1 TCA_citrate-hydro-lyase) (-1 TCA_citrate_synthase) |
| 158 | 0,48 | 2 | false | 2 | (1 Glyc_pyruvate_dehydrogenase) (1 TCA_pyruvate_dehydrogenase) |
| 159 | 1,00 | 17 | false | 6 | (-3 Glyc_acetaldehyde-dehydrogenase_NAD+) (-3 Glyc_Actetate-CoA-ligase) (2 OP_complex5) (-3 SERP0389-Glyc_Ethanol_NAD+-oxidoreductase) (-3 TCA_citrate-hydro-lyase) (3 TCA_citrate_synthase) |
| 160 | 1,00 | 1 | false | 1 | (1 AS_Aspartate_to_Alanine) |
| 161 | 0,66 | 2 | false | 2 | (1 AS_Serine_to_Cysteine) (1 Glyc_Actetate-CoA-ligase) |
| 162 | 1,00 | 4 | false | 3 | (1 AMP-energy_to_AMP-metabolism) (1 PurM_adenylate-kinase_AMP) (2 PurM_thioredoxin-oxidoreductase_dATP) |
| 163 | 1,00 | 1 | false | 1 | (1 SERP2186-PurM_ATP_sulfate-adenylyltransferase) |
| 164 | 1,00 | 3 | false | 3 | (-1 AMP-energy_to_AMP-metabolism) (1 PurM_ADP-ribose-ribophosphohydrolase) (1 PurM_PRPP-synthetase) |
| 165 | 1,00 | 2 | false | 2 | (1 IQ-143-extern_to_IQ-) (1 SERP1403-MultiDrug-transport_efflux) |
| 166 | 0,25 | 13 | false | 3 | (1 FA_Syn_Acetyl-CoA_to_C16) (-6 TCA_citrate-hydro-lyase) (6 TCA_citrate_synthase) |
| 167 | 0,51 | 1 | false | 1 | (1 PyrM_UTP_L-glutamine-amido-ligase) |
| 168 | 0,25 | 1 | false | 1 | (1 AS_Phenylalanin_to_Tyrosine) |
| 169 | 1,00 | 1 | false | 1 | (1 PurM_5-nucleotidase_IMP) |
| 170 | 1,00 | 2 | false | 2 | (1 PurM_GDP-reductase) (1 PurM_IMP-dehydrogenase) |
| 171 | 0,00 | 3 | false | 3 | (1 IQ-143-extern_to_IQ-) (1 PurM_IMP-dehydrogenase) (1 SERP1944-MultiDrug-transport_efflux) |
| 172 | 0,25 | 2 | false | 2 | (1 PurM_IMP-dehydrogenase) (1 SERP1997-formate/nitrite-transport_efflux/import) |
| 173 | 0,48 | 1 | false | 1 | (1 PurM_5-nucleotidase_GMP) |
| 174 | 0,25 | 1 | false | 1 | (1 PyrM_cytidine-kinase_ITP) |
| 175 | 1,00 | 1 | false | 1 | (1 AS_Histidine_to_Glutamate) |
| 176 | 1,00 | 1 | false | 1 | (1 PyrM_cytidine-kinase_GTP) |
| 177 | 1,00 | 13 | false | 3 | (1 FA_Deg_C16_to_Acetyl-CoA) (6 TCA_citrate-hydro-lyase) (-6 TCA_citrate_synthase) |
| 178 | 1,00 | 16 | false | 9 | (-1 AMP-energy_to_AMP-metabolism) (-2 Glyc_alpha-D-Glucose-6-phosphate-ketol-isomerase2) (-2 Glyc_ATP-alpha-D-glucokinase) (2 Glyc_ATP-beta-D-glucokinase) (2 Glyc_beta-D-Glucose-6-phosphate-ketol-isomerase) (2 Glyc_D-Glucose-1-epimerase) (-1 PurM_adenylate-kinase_AMP) (2 PurM_pyruvate-phosphotransferase_ATP) (-2 TCA_PEP-carboxylase) |
| 179 | 0,48 | 1 | false | 1 | (1 PyrM_cytidine-kinase_dUTP) |
| 180 | 1,00 | 6 | false | 6 | (-1 Glyc_alpha-D-Glucose-6-phosphate-ketol-isomerase2) (-1 Glyc_ATP-alpha-D-glucokinase) (1 Glyc_ATP-beta-D-glucokinase) (1 Glyc_beta-D-Glucose-6-phosphate-ketol-isomerase) (1 Glyc_D-Glucose-1-epimerase) (1 PurM_DNA-directed-RNA-polymerase_ATP) |
| 181 | 1,00 | 13 | false | 8 | (-1 AMP-energy_to_AMP-metabolism) (-2 Glyc_alpha-D-Glucose-6-phosphate-ketol-isomerase2) (-2 Glyc_ATP-alpha-D-glucokinase) (2 Glyc_ATP-beta-D-glucokinase) (2 Glyc_beta-D-Glucose-6-phosphate-ketol-isomerase) (2 Glyc_D-Glucose-1-epimerase) (-1 PurM_adenylate-kinase_AMP) (1 SERP0687-spermidine/putrescine-transport_import) |
| 182 | 0,41 | 1 | false | 1 | (1 PurM_thioredoxin-oxidoreductase_dUDP) |
| 183 | 0,48 | 1 | false | 1 | (1 PurM_dGTP-diphosphohydrolase) |
| 184 | 1,00 | 1 | false | 1 | (1 PurM_DNA-directed-RNA-polyermase_CTP) |
| 185 | 1,00 | 1 | false | 1 | (1 SERP0831-PurM_DNA-directed-DNA-polymerase_dGTP) |
| 186 | 1,00 | 4 | false | 3 | (1 AMP-energy_to_AMP-metabolism) (1 PurM_adenylate-kinase_AMP) (2 PurM_thioredoxin-oxidoreductased_dADP) |
| 187 | 1,00 | 1 | false | 1 | (1 PurM_thioredoxin-oxidoreductase_dCTP) |
| 188 | 1,00 | 1 | false | 1 | (1 AS_Tryptophan_to_Tryptamine) |
| 189 | 1,00 | 3 | false | 3 | (1 AS_Acetyl-CoA_to_L-Leucine) (-1 TCA_citrate-hydro-lyase) (1 TCA_citrate_synthase) |
| 190 | 1,00 | 1 | false | 1 | (1 AS_Homoserine_to_Threonine) |
| 191 | 1,00 | 1 | false | 1 | (1 PurM_5-nucleotidase_CMP) |
| 192 | 1,00 | 1 | false | 1 | (1 PyrM_cytidine-kinase_UTP) |
| 193 | 1,00 | 1 | false | 1 | (1 PyrM_uridine-kinase_dUTP) |
| 194 | 1,00 | 1 | false | 1 | (1 PyrM_uridine-kinase_ITP) |
| 195 | 0,56 | 1 | false | 1 | (1 PyrM_cytidine-kinase_dATP) |
| 196 | 1,00 | 1 | false | 1 | (1 SERP2060-glyerol-transport_import) |
| 197 | 1,00 | 1 | false | 1 | (1 PyrM_uridine-kinase_GTP) |

^1^ This data shows the elementary mode Analysis for *S. epidermidis* RP62A without IQ-143.

Table S11: Extreme Modes of *S. epidermidis* RP62A with 0.16µM IQ-143^1^:

| # | Activity | Flux sum | Reversible? | Pathlength | Reactions |
| --- | --- | --- | --- | --- | --- |
| 1 | 1,00 | 1 | true | 1 | (1 AS_Alanine_to_Pyruvate) |
| 2 | 1,00 | 1 | true | 1 | (1 PyrM_dCMP-aminohydrolase) |
| 3 | 0,70 | 1 | true | 1 | (1 PurM_ATP_IDP-phosphotransferase) |
| 4 | 1,00 | 1 | true | 1 | (1 PyrM_cytidilate-kinase_dCMP) |
| 5 | 1,00 | 1 | true | 1 | (1 PyrM_dUMP-phosphotransferase) |
| 6 | 1,00 | 1 | true | 1 | (1 PurM_ATP_GMP-guanylate-kinase) |
| 7 | 1,00 | 1 | true | 1 | (1 PyrM_cytidine-aminohydrolase) |
| 8 | 1,00 | 1 | true | 1 | (1 PurM_ATP_UTP-phosphotransferase) |
| 9 | 1,00 | 1 | true | 1 | (1 PurM_ATP_GTP-phosphotransferase) |
| 10 | 1,00 | 2 | true | 2 | (-1 PurM_nucleotide-phosphatase_Deoxyguanosine) (1 PyrM_deoxyguanosine-phosphorylase) |
| 11 | 1,00 | 2 | true | 2 | (-1 PyrM_dihydroorotase) (1 PyrM_dihydroorotate-oxidase) |
| 12 | 1,00 | 1 | true | 1 | (1 PurM_ATP_dIDP-phosphotransferase) |
| 13 | 1,00 | 1 | true | 1 | (1 PyrM_thymidine-kinase_dTMP) |
| 14 | 1,00 | 1 | true | 1 | (1 PurM_xanthosine-phosphoribosyltransferase) |
| 15 | 1,00 | 1 | true | 1 | (1 PurM_ATP_dUDP-phosphotransferase) |
| 16 | 1,00 | 1 | true | 1 | (1 TCA_fumarate-hydratase) |
| 17 | 0,91 | 3 | true | 3 | (1 PurM_IMP-pyrophosphorylase) (-1 PurM_nucleotide-phosphatase_Inosine) (1 PyrM_uridine-phosphorylase) |
| 18 | 1,00 | 3 | true | 3 | (1 PurM_IMP-pyrophosphorylase) (1 PurM_nucleotide-phosphatase_Guanosine) (-1 PurM_nucleotide-phosphatase_Inosine) |
| 19 | 1,00 | 3 | true | 3 | (1 PurM_IMP-pyrophosphorylase) (-1 PurM_nucleotide-phosphatase_Inosine) (1 PurM_nucleotide-phosphatase_Xanthosine) |
| 20 | 1,00 | 3 | true | 3 | (1 PurM_IMP-pyrophosphorylase) (-1 PurM_nucleotide-phosphatase_Deoxyinosine) (1 PyrM_deoxyinosine-phosphorylase) |
| 21 | 1,00 | 1 | true | 1 | (1 AS_Serine_to_Glycine) |
| 22 | 1,00 | 1 | true | 1 | (1 SERP1951-lipoprotein-transport_efflux/import) |
| 23 | 1,00 | 1 | true | 1 | (1 PurM_GMP-pyrophosphorylase2) |
| 24 | 1,00 | 1 | true | 1 | (1 PyrM_nucleoside-phosphate-kinase_ATP) |
| 25 | -0,52 | 1 | true | 1 | (1 PyrM_ATP_dTDP_thymidylate-kinase) |
| 26 | 1,00 | 1 | true | 1 | (1 PurM_ATP_dTDP-phosphotransferase) |
| 27 | 1,00 | 1 | true | 1 | (1 PurM_ATP_GMP_guanylate-kinase) |
| 28 | 1,00 | 1 | true | 1 | (1 PyrM_nucleoside-phosphate-kinase_ATP2) |
| 29 | 1,00 | 2 | true | 2 | (-1 PurM_nucleotide-phosphatase_Deoxyuridine) (1 PyrM_deoxyuridine-phosphorylase) |
| 30 | -1,33 | 2 | true | 2 | (1 PyrM_deoxyuridine-phosphorylase) (-1 PyrM_thymidine-kinase_dUMP) |
| 31 | 1,00 | 1 | true | 1 | (1 PurM_ATP_dGDP-phosphotransferase) |
| 32 | 1,00 | 1 | true | 1 | (1 AS_Aspartate_to_Homoserine) |
| 33 | 1,00 | 1 | true | 1 | (1 PurM_ATP_dADP-phosphotransferase) |
| 34 | 1,00 | 2 | true | 2 | (1 TCA_citrate-hydro-lyase) (-1 TCA_citrate-hydroxymutase) |
| 35 | 0,91 | 3 | true | 3 | (1 Glyc_lipoic_acetyltransferase) (-1 TCA_citrate-hydro-lyase) (1 TCA_citrate_synthase) |
| 36 | -1,00 | 7 | true | 6 | (-1 Glyc_acetaldehyde-dehydrogenase_NAD+) (-1 Glyc_Actetate-CoA-ligase) (-1 SERP0389-Glyc_Ethanol_NAD+-oxidoreductase) (2 SERP2156-Glyc_L-lactate-dehydrogenase) (-1 TCA_citrate-hydro-lyase) (1 TCA_citrate_synthase) |
| 37 | 1,00 | 1 | true | 1 | (1 PurM_ATP_CDP-phosphotransferase) |
| 38 | 1,00 | 1 | true | 1 | (1 AS_Saccharopine_to_Lysine) |
| 39 | 1,00 | 1 | true | 1 | (1 PurM_IMP_L-aspartate-ligase) |
| 40 | 0,50 | 1 | true | 1 | (1 Glyc_Succinate-CoA-ligase) |
| 41 | 0,50 | 3 | true | 3 | (1 AS_Acetyl-CoA_to_L-Valine) (-1 TCA_citrate-hydro-lyase) (1 TCA_citrate_synthase) |
| 42 | 1,00 | 1 | true | 1 | (1 PyrM_orotate-phosphoribosyltransferase) |
| 43 | 1,00 | 1 | true | 1 | (1 PurM_metaphosphatase) |
| 44 | 1,05 | 1 | true | 1 | (1 PyrM_cytidilate-kinase_CTP) |
| 45 | 0,73 | 1 | true | 1 | (1 PurM_GMP-pyrophosphorylase) |
| 46 | 1,00 | 3 | true | 3 | (1 Glyc_alpha-D-Glucose-6-phosphate-ketol-isomerase) (-1 Glyc_alpha-D-Glucose-6-phosphate-ketol-isomerase2) (1 Glyc_beta-D-Glucose-6-phosphate-ketol-isomerase) |
| 47 | 1,00 | 6 | true | 6 | (1 ATP-energy_to_ATP-metabolism) (1 Glyc_alpha-D-Glucose-6-phosphate-ketol-isomerase2) (1 Glyc_ATP-alpha-D-glucokinase) (-1 Glyc_ATP-beta-D-glucokinase) (-1 Glyc_beta-D-Glucose-6-phosphate-ketol-isomerase) (-1 Glyc_D-Glucose-1-epimerase) |
| 48 | 0,75 | 1 | true | 1 | (1 Glyc_PTS-permease2) |
| 49 | 1,00 | 4 | true | 4 | (-1 Glyc_6-phospho-beta-glucosidase) (1 Glyc_alpha-D-Glucose-6-phosphate-ketol-isomerase2) (-1 Glyc_beta-D-Glucose-6-phosphate-ketol-isomerase) (1 Glyc_PTS-permease1) |
| 50 | 1,00 | 1 | true | 1 | (1 PyrM_thymidine-phosphorylase) |
| 51 | 1,00 | 1 | true | 1 | (1 PurM_ATP_dCDP-phosphotransferase) |
| 52 | -1,34 | 1 | true | 1 | (1 PyrM_UMP-pyrophosphorylase) |
| 53 | 0,79 | 1 | true | 1 | (1 PyrM_nucleoside-triphosphate-adenylate-kinase) |
| 54 | 0,53 | 1 | true | 1 | (1 PurM_XMP-pyrophosphorylase) |
| 55 | 0,53 | 1 | true | 1 | (1 TCA_succinate-dehydrogenase) |
| 56 | 0,53 | 2 | true | 2 | (1 PyrM_Deoxycytidine-aminohydrolase) (-1 PyrM_Deoxycytidine-deaminase) |
| 57 | 0,53 | 2 | true | 2 | (-1 PurM_nucleotide-phosphatase_Deoxyadenosine) (1 PyrM_deoxyadenosine-phosphorylase) |
| 58 | 0,53 | 1 | true | 1 | (1 PyrM_ATP_dUDP_thymidylate-kinase) |
| 59 | 0,92 | 1 | true | 1 | (1 PurM_adenylate-kinase_dAMP) |
| 60 | 1,08 | 2 | true | 2 | (1 AMP-energy_to_AMP-metabolism) (1 PurM_AMP-pyrophosphorylase2) |
| 61 | -0,65 | 2 | true | 2 | (-1 AMP-energy_to_AMP-metabolism) (1 PurM_adenylosuccinate-lyase) |
| 62 | 0,92 | 2 | true | 2 | (1 AMP-energy_to_AMP-metabolism) (1 PurM_AMP-pyrophosphorylase) |
| 63 | -0,48 | 14 | true | 8 | (1 AMP-energy_to_AMP-metabolism) (2 Glyc_alpha-D-Glucose-6-phosphate-ketol-isomerase2) (2 Glyc_ATP-alpha-D-glucokinase) (-2 Glyc_ATP-beta-D-glucokinase) (-2 Glyc_beta-D-Glucose-6-phosphate-ketol-isomerase) (-2 Glyc_D-Glucose-1-epimerase) (1 PurM_adenylate-kinase_AMP) (2 PurM_nucleoside-diphosphate-phosphotransferase_ATP) |
| 64 | 1,00 | 1 | true | 1 | (1 PurM_adenylylsulfate-kinase) |
| 65 | 1,00 | 4 | true | 3 | (2 ADP-energy_to_ADP-metabolism) (-1 AMP-energy_to_AMP-metabolism) (-1 PurM_adenylate-kinase_AMP) |
| 66 | 1,00 | 3 | true | 3 | (1 PurM_IMP-pyrophosphorylase) (1 PurM_nucleotide-phosphatase_Adenine) (-1 PurM_nucleotide-phosphatase_Inosine) |
| 67 | 1,00 | 3 | true | 3 | (1 PurM_IMP-pyrophosphorylase) (-1 PurM_nucleotide-phosphatase_Inosine) (1 PyrM_pyrimidine-nucleoside-phosphorylase) |
| 68 | 1,00 | 1 | true | 1 | (1 TCA_isocitrate-hydro-lyase) |
| 69 | 1,00 | 14 | false | 8 | (-1 AMP-energy_to_AMP-metabolism) (-2 Glyc_alpha-D-Glucose-6-phosphate-ketol-isomerase2) (-2 Glyc_ATP-alpha-D-glucokinase) (2 Glyc_ATP-beta-D-glucokinase) (2 Glyc_beta-D-Glucose-6-phosphate-ketol-isomerase) (2 Glyc_D-Glucose-1-epimerase) (-1 PurM_adenylate-kinase_AMP) (2 PurM_ATP-phosphohydrolase) |
| 70 | 1,00 | 2 | false | 2 | (1 AMP-energy_to_AMP-metabolism) (1 PurM_5-nucleotidase_AMP) |
| 71 | 1,00 | 1 | false | 1 | (1 PyrM_uridine-kinase_dGTP) |
| 72 | 1,00 | 4 | false | 3 | (1 AMP-energy_to_AMP-metabolism) (1 PurM_adenylate-kinase_AMP) (2 SERP0841-PurM_PNPase_ADP) |
| 73 | 1,00 | 1 | false | 1 | (1 PurM_5-nucleotidase_XMP) |
| 74 | 1,00 | 1 | false | 1 | (1 SERP0831-PurM_DNA-directed-DNA-polymerase_dATP) |
| 75 | 1,00 | 1 | false | 1 | (1 PyrM_uridine-kinase_dTTP) |
| 76 | 1,00 | 1 | false | 1 | (1 PyrM_cytidine-kinase_dCTP) |
| 77 | 1,00 | 1 | false | 1 | (1 SERP1952-macrolide-transport_efflux) |
| 78 | 1,00 | 2 | false | 2 | (1 PurM_deoxyadenosine-kinase_ATP) (-1 PyrM_deoxyadenosine-phosphorylase) |
| 79 | 0,55 | 1 | false | 1 | (1 PyrM_dUTP-diphosphatase) |
| 80 | 1,00 | 1 | false | 1 | (1 AS_Aspartate_to_beta-Alanine) |
| 81 | 1,00 | 1 | false | 1 | (1 SERP1803-cobalt/nickel-transport_efflux) |
| 82 | 1,00 | 1 | false | 1 | (1 PyrM_cytidine-kinase_dGTP) |
| 83 | 1,00 | 1 | false | 1 | (1 PurM_thioredoxin-oxidoreductase_dUTP) |
| 84 | 1,00 | 1 | false | 1 | (1 SERP0292-iron-dicitrate-transporter_import) |
| 85 | 0,25 | 2 | false | 2 | (1 Glyc_6-phosphofructokinase) (1 Glyc_fructose-bisphosphatase) |
| 86 | 1,00 | 1 | false | 1 | (1 PurM_XMP_L-glutamine-amide-ligase) |
| 87 | 1,00 | 1 | false | 1 | (1 AS_Aspartate_to_Arginine) |
| 88 | 1,00 | 7 | false | 6 | (1 Glyc_acetaldehyde-dehydrogenase_NAD+) (1 Glyc_Actetate-CoA-ligase) (2 PyrM_thioredoxin-reductase) (1 SERP0389-Glyc_Ethanol_NAD+-oxidoreductase) (1 TCA_citrate-hydro-lyase) (-1 TCA_citrate_synthase) |
| 89 | 1,00 | 20 | false | 15 | (-2 Glyc_2-Phospho-D-glycerate-2.3-phosphomutase) (2 Glyc_2-phospho-D-glycerate-hydro-lyase) (1 Glyc_6-phospho-beta-glucosidase) (1 Glyc_6-phosphofructokinase) (-1 Glyc_acetaldehyde-dehydrogenase_NAD+) (-1 Glyc_Actetate-CoA-ligase) (1 Glyc_beta-D-Glucose-6-phosphate-ketol-isomerase) (-1 Glyc_D-Glucose-1-epimerase-ketol-isomerase) (1 Glyc_fructose-bisphosphat-aldolase) (2 Glyc_glyceraldehyde-3-P-dehydrogenase_NAD+) (-2 Glyc_phosphoglycerate-kinase) (-1 SERP0389-Glyc_Ethanol_NAD+-oxidoreductase) (-1 TCA_citrate-hydro-lyase) (1 TCA_citrate_synthase) (-2 TCA_PEP-carboxylase) |
| 90 | 0,96 | 1 | false | 1 | (1 PyrM_2,3-cyclic-nucleotidase_UMP) |
| 91 | 1,00 | 1 | false | 1 | (1 DNA-extern_to_DNA-intern) |
| 92 | 0,67 | 1 | false | 1 | (1 SERP0831-PurM_DNA-directed-DNA-polymerase_dCTP) |
| 93 | 0,36 | 1 | false | 1 | (1 SERP0841-PurM_PNPase_GDP) |
| 94 | 0,41 | 2 | false | 2 | (1 PurM_carbamate-kinase_ATP) (1 PyrM_aspartate-carbamoyltransferase) |
| 95 | 1,00 | 1 | false | 1 | (1 PurM_thioredoxin-oxidoreductase_dGDP) |
| 96 | 0,30 | 1 | false | 1 | (1 PurM_ITP-diphosphohydrolase) |
| 97 | 0,35 | 13 | false | 8 | (-1 AMP-energy_to_AMP-metabolism) (-2 Glyc_alpha-D-Glucose-6-phosphate-ketol-isomerase2) (-2 Glyc_ATP-alpha-D-glucokinase) (2 Glyc_ATP-beta-D-glucokinase) (2 Glyc_beta-D-Glucose-6-phosphate-ketol-isomerase) (2 Glyc_D-Glucose-1-epimerase) (-1 PurM_adenylate-kinase_AMP) (1 SERP0688-spermidine/putrescine-transport_import) |
| 98 | 1,00 | 3 | false | 3 | (1 PurM_5-nucleotidase_dCMP) (1 PyrM_Deoxycytidine-aminohydrolase) (1 PyrM_deoxyuridine-phosphorylase) |
| 99 | 1,00 | 14 | false | 8 | (-1 AMP-energy_to_AMP-metabolism) (-2 Glyc_alpha-D-Glucose-6-phosphate-ketol-isomerase2) (-2 Glyc_ATP-alpha-D-glucokinase) (2 Glyc_ATP-beta-D-glucokinase) (2 Glyc_beta-D-Glucose-6-phosphate-ketol-isomerase) (2 Glyc_D-Glucose-1-epimerase) (-1 PurM_adenylate-kinase_AMP) (2 SERP0686-spermidine/putrescine-transport_import) |
| 100 | 0,36 | 1 | false | 1 | (1 PyrM_dUTP-diphosphohydrolase) |
| 101 | 1,00 | 1 | false | 1 | (1 PurM_dITP-diphosphohydrolase) |
| 102 | 0,35 | 2 | false | 2 | (1 PurM_pyruvate-phosphotransferase_GTP) (-1 TCA_PEP-carboxylase) |
| 103 | 1,00 | 2 | false | 2 | (1 PurM_pyruvate-phosphotransferase_dATP) (-1 TCA_PEP-carboxylase) |
| 104 | 1,00 | 1 | false | 1 | (1 PurM_5-nucleotidase_UMP) |
| 105 | 1,00 | 1 | false | 1 | (1 SERP2283-phopsphonate-transport_import) |
| 106 | 1,00 | 2 | false | 2 | (1 PurM_GDP-reductase) (1 SERP2179-choline/betaine/carnitine-transp_efflux) |
| 107 | 0,48 | 1 | false | 1 | (1 AS_Valine) |
| 108 | 1,00 | 2 | false | 2 | (1 PurM_5-nucleotidase_dAMP) (1 PyrM_deoxyadenosine-phosphorylase) |
| 109 | 0,92 | 1 | false | 1 | (1 SERP1802-cobalt/nickel-transport_efflux) |
| 110 | 1,00 | 1 | false | 1 | (1 PurM_DNA-directed-RNA-polyermase_UTP) |
| 111 | 1,00 | 4 | false | 4 | (-1 TCA_lipoic-transsuccinylase) (1 TCA_oxoglutarate-dehydrogenase-complex1) (1 TCA_oxoglutarate-dehydrogenase-complex2) (1 TCA_oxoglutarate-synthase) |
| 112 | 1,00 | 1 | false | 1 | (1 PyrM_cytidine-kinase_dTTP) |
| 113 | 1,00 | 1 | false | 1 | (1 PurM_UTP-diphosphohydrolase) |
| 114 | 1,00 | 1 | false | 1 | (1 AS_Serine_to_Methionine) |
| 115 | 1,00 | 2 | false | 2 | (1 PurM_5-nucleotidase_dGMP) (1 PyrM_deoxyguanosine-phosphorylase) |
| 116 | 0,19 | 2 | false | 2 | (-1 AMP-energy_to_AMP-metabolism) (1 PurM_deoxycytidine-kinase_ATP) |
| 117 | 0,19 | 1 | false | 1 | (1 PurM_thioredoxin-oxidoreductase_dGTP) |
| 118 | 1,00 | 3 | false | 3 | (1 IQ-143-extern_to_IQ-) (1 SERP1944-MultiDrug-transport_efflux) (1 SERP2179-choline/betaine/carnitine-transp_efflux) |
| 119 | 0,36 | 1 | false | 1 | (1 AS_Leucine) |
| 120 | 0,36 | 20 | false | 15 | (-2 Glyc_2-Phospho-D-glycerate-2.3-phosphomutase) (2 Glyc_2-phospho-D-glycerate-hydro-lyase) (1 Glyc_6-phospho-beta-glucosidase) (1 Glyc_6-phosphofructokinase) (-1 Glyc_acetaldehyde-dehydrogenase_NAD+) (-1 Glyc_Actetate-CoA-ligase) (1 Glyc_beta-D-Glucose-6-phosphate-ketol-isomerase) (-1 Glyc_D-Glucose-1-epimerase-ketol-isomerase) (1 Glyc_fructose-bisphosphat-aldolase) (2 Glyc_glyceraldehyde-3-P-dehydrogenase_NADP+) (-2 Glyc_phosphoglycerate-kinase) (-1 SERP0389-Glyc_Ethanol_NAD+-oxidoreductase) (-1 TCA_citrate-hydro-lyase) (1 TCA_citrate_synthase) (-2 TCA_PEP-carboxylase) |
| 121 | 0,25 | 1 | false | 1 | (1 PyrM_2,3-cyclic-nucleotidase_CMP) |
| 122 | 0,48 | 2 | false | 2 | (1 SERP1997-formate/nitrite-transport_efflux/import) (1 SERP2179-choline/betaine/carnitine-transp_efflux) |
| 123 | 1,00 | 1 | false | 1 | (1 PyrM_CTP-synthase) |
| 124 | 0,80 | 2 | false | 2 | (1 IQ-143-extern_to_IQ-) (1 SERP2289-MultiDrug-transport_efflux) |
| 125 | 1,00 | 1 | false | 1 | (1 AS_Glutamate_to_Proline) |
| 126 | 0,75 | 1 | false | 1 | (1 PyrM_uridine-kinase_dCTP) |
| 127 | 1,75 | 1 | false | 1 | (1 PyrM_uridine-kinase_UTP) |
| 128 | 1,00 | 3 | false | 3 | (1 PurM_deoxycytidine-kinase_ATP2) (-1 PyrM_Deoxycytidine-aminohydrolase) (-1 PyrM_deoxyuridine-phosphorylase) |
| 129 | 1,00 | 1 | false | 1 | (1 AS_Threonine) |
| 130 | 1,40 | 1 | false | 1 | (1 SERP0831-PurM_DNA-directed-DNA-polymerase_dTTP) |
| 131 | 1,00 | 1 | false | 1 | (1 PurM_GTP-diphosphohydrolase) |
| 132 | 1,00 | 1 | false | 1 | (1 PyrM_uridine-kinase_dATP) |
| 133 | 1,00 | 1 | false | 1 | (1 AS_Serine_to_Pyruvate) |
| 134 | 1,00 | 2 | false | 2 | (1 SERP0290-zinc-transport_efflux) (1 SERP0291-zinc-transporter_import) |
| 135 | 1,00 | 1 | false | 1 | (1 AS_Isoleucine) |
| 136 | 1,00 | 1 | false | 1 | (1 PurM_thioredoxin-oxidoreductase_dCDP) |
| 137 | 0,52 | 2 | false | 2 | (1 PurM_pyruvate-phosphotransferase_dGTP) (-1 TCA_PEP-carboxylase) |
| 138 | 1,00 | 1 | false | 1 | (1 PurM_5-nucleotidase_dTMP) |
| 139 | 0,36 | 1 | false | 1 | (1 PyrM_uridine-kinase_ATP) |
| 140 | 0,19 | 4 | false | 4 | (1 PurM_D-Ribose-1,5-phosphomutase) (-1 PurM_IMP-pyrophosphorylase) (1 PurM_nucleotide-phosphatase_Inosine) (1 PurM_PRPP-synthetase) |
| 141 | 0,36 | 1 | false | 1 | (1 PurM_DNA-directed-RNA-polyermase_GTP) |
| 142 | 1,00 | 7 | false | 6 | (-1 Glyc_acetaldehyde-dehydrogenase_NAD+) (-1 Glyc_Actetate-CoA-ligase) (2 Glyc_dihydrolipoamide-dehydrogenase) (-1 SERP0389-Glyc_Ethanol_NAD+-oxidoreductase) (-1 TCA_citrate-hydro-lyase) (1 TCA_citrate_synthase) |
| 143 | 1,00 | 1 | false | 1 | (1 PyrM_OMP-decarboxylase) |
| 144 | 1,00 | 1 | false | 1 | (1 AS_Glutamate_to_Glutamine) |
| 145 | 1,00 | 1 | false | 1 | (1 PurM_urea-amidohydrolase) |
| 146 | 1,00 | 1 | false | 1 | (1 AS_Aspartate_to_Asparagine) |
| 147 | 1,00 | 1 | false | 1 | (1 PyrM_cytidine-kinase_ATP) |
| 148 | 0,64 | 2 | false | 2 | (1 PurM_GDP-reductase) (1 SERP0765-Uracil-permease-transport_import) |
| 149 | 0,48 | 3 | false | 3 | (1 IQ-143-extern_to_IQ-) (1 SERP0765-Uracil-permease-transport_import) (1 SERP1944-MultiDrug-transport_efflux) |
| 150 | 0,91 | 2 | false | 2 | (1 SERP0765-Uracil-permease-transport_import) (1 SERP1997-formate/nitrite-transport_efflux/import) |
| 151 | 0,56 | 13 | false | 9 | (-1 Glyc_acetaldehyde-dehydrogenase_NAD+) (-1 Glyc_Actetate-CoA-ligase) (-1 SERP0389-Glyc_Ethanol_NAD+-oxidoreductase) (-1 TCA_citrate-hydro-lyase) (1 TCA_citrate_synthase) (-2 TCA_lipoic-transsuccinylase) (2 TCA_Oxidoreductase) (2 TCA_oxoglutarate-dehydrogenase-complex1) (2 TCA_oxoglutarate-dehydrogenase-complex2) |
| 152 | 1,00 | 1 | false | 1 | (1 PurM_XTP-diphosphohydrolase) |
| 153 | 1,00 | 3 | false | 3 | (-1 PurM_carbamate-kinase_ATP) (1 PyrM_CO2_L-glutamine-amido-ligase) (-1 TCA_Pyruvate_CO2-ligase) |
| 154 | 0,60 | 12 | false | 7 | (2 Glyc_acetaldehyde-dehydrogenase_NAD+) (2 Glyc_Actetate-CoA-ligase) (1 OP_complex1) (1 OP_complex3) (2 SERP0389-Glyc_Ethanol_NAD+-oxidoreductase) (2 TCA_citrate-hydro-lyase) (-2 TCA_citrate_synthase) |
| 155 | 0,48 | 17 | false | 7 | (3 Glyc_acetaldehyde-dehydrogenase_NAD+) (3 Glyc_Actetate-CoA-ligase) (1 OP_complex2) (1 OP_complex3) (3 SERP0389-Glyc_Ethanol_NAD+-oxidoreductase) (3 TCA_citrate-hydro-lyase) (-3 TCA_citrate_synthase) |
| 156 | 1,00 | 1 | false | 1 | (1 PurM_GTP-pyrophosphokinase) |
| 157 | 1,00 | 6 | false | 6 | (1 Glyc_acetaldehyde-dehydrogenase_NAD+) (1 Glyc_Actetate-CoA-ligase) (1 OP_complex4) (1 SERP0389-Glyc_Ethanol_NAD+-oxidoreductase) (1 TCA_citrate-hydro-lyase) (-1 TCA_citrate_synthase) |
| 158 | 0,48 | 2 | false | 2 | (1 Glyc_pyruvate_dehydrogenase) (1 TCA_pyruvate_dehydrogenase) |
| 159 | 1,00 | 17 | false | 6 | (-3 Glyc_acetaldehyde-dehydrogenase_NAD+) (-3 Glyc_Actetate-CoA-ligase) (2 OP_complex5) (-3 SERP0389-Glyc_Ethanol_NAD+-oxidoreductase) (-3 TCA_citrate-hydro-lyase) (3 TCA_citrate_synthase) |
| 160 | 1,00 | 1 | false | 1 | (1 AS_Aspartate_to_Alanine) |
| 161 | 0,66 | 2 | false | 2 | (1 AS_Serine_to_Cysteine) (1 Glyc_Actetate-CoA-ligase) |
| 162 | 1,00 | 4 | false | 3 | (1 AMP-energy_to_AMP-metabolism) (1 PurM_adenylate-kinase_AMP) (2 PurM_thioredoxin-oxidoreductase_dATP) |
| 163 | 1,00 | 1 | false | 1 | (1 SERP2186-PurM_ATP_sulfate-adenylyltransferase) |
| 164 | 1,00 | 3 | false | 3 | (-1 AMP-energy_to_AMP-metabolism) (1 PurM_ADP-ribose-ribophosphohydrolase) (1 PurM_PRPP-synthetase) |
| 165 | 1,00 | 2 | false | 2 | (1 IQ-143-extern_to_IQ-) (1 SERP1403-MultiDrug-transport_efflux) |
| 166 | 0,25 | 13 | false | 3 | (1 FA_Syn_Acetyl-CoA_to_C16) (-6 TCA_citrate-hydro-lyase) (6 TCA_citrate_synthase) |
| 167 | 0,51 | 1 | false | 1 | (1 PyrM_UTP_L-glutamine-amido-ligase) |
| 168 | 0,25 | 1 | false | 1 | (1 AS_Phenylalanin_to_Tyrosine) |
| 169 | 1,00 | 1 | false | 1 | (1 PurM_5-nucleotidase_IMP) |
| 170 | 1,00 | 2 | false | 2 | (1 PurM_GDP-reductase) (1 PurM_IMP-dehydrogenase) |
| 171 | 0,00 | 3 | false | 3 | (1 IQ-143-extern_to_IQ-) (1 PurM_IMP-dehydrogenase) (1 SERP1944-MultiDrug-transport_efflux) |
| 172 | 0,25 | 2 | false | 2 | (1 PurM_IMP-dehydrogenase) (1 SERP1997-formate/nitrite-transport_efflux/import) |
| 173 | 0,48 | 1 | false | 1 | (1 PurM_5-nucleotidase_GMP) |
| 174 | 0,25 | 1 | false | 1 | (1 PyrM_cytidine-kinase_ITP) |
| 175 | 1,00 | 1 | false | 1 | (1 AS_Histidine_to_Glutamate) |
| 176 | 1,00 | 1 | false | 1 | (1 PyrM_cytidine-kinase_GTP) |
| 177 | 1,00 | 13 | false | 3 | (1 FA_Deg_C16_to_Acetyl-CoA) (6 TCA_citrate-hydro-lyase) (-6 TCA_citrate_synthase) |
| 178 | 1,00 | 16 | false | 9 | (-1 AMP-energy_to_AMP-metabolism) (-2 Glyc_alpha-D-Glucose-6-phosphate-ketol-isomerase2) (-2 Glyc_ATP-alpha-D-glucokinase) (2 Glyc_ATP-beta-D-glucokinase) (2 Glyc_beta-D-Glucose-6-phosphate-ketol-isomerase) (2 Glyc_D-Glucose-1-epimerase) (-1 PurM_adenylate-kinase_AMP) (2 PurM_pyruvate-phosphotransferase_ATP) (-2 TCA_PEP-carboxylase) |
| 179 | 0,48 | 1 | false | 1 | (1 PyrM_cytidine-kinase_dUTP) |
| 180 | 1,00 | 6 | false | 6 | (-1 Glyc_alpha-D-Glucose-6-phosphate-ketol-isomerase2) (-1 Glyc_ATP-alpha-D-glucokinase) (1 Glyc_ATP-beta-D-glucokinase) (1 Glyc_beta-D-Glucose-6-phosphate-ketol-isomerase) (1 Glyc_D-Glucose-1-epimerase) (1 PurM_DNA-directed-RNA-polymerase_ATP) |
| 181 | 1,00 | 13 | false | 8 | (-1 AMP-energy_to_AMP-metabolism) (-2 Glyc_alpha-D-Glucose-6-phosphate-ketol-isomerase2) (-2 Glyc_ATP-alpha-D-glucokinase) (2 Glyc_ATP-beta-D-glucokinase) (2 Glyc_beta-D-Glucose-6-phosphate-ketol-isomerase) (2 Glyc_D-Glucose-1-epimerase) (-1 PurM_adenylate-kinase_AMP) (1 SERP0687-spermidine/putrescine-transport_import) |
| 182 | 0,41 | 1 | false | 1 | (1 PurM_thioredoxin-oxidoreductase_dUDP) |
| 183 | 0,48 | 1 | false | 1 | (1 PurM_dGTP-diphosphohydrolase) |
| 184 | 1,00 | 1 | false | 1 | (1 PurM_DNA-directed-RNA-polyermase_CTP) |
| 185 | 1,00 | 1 | false | 1 | (1 SERP0831-PurM_DNA-directed-DNA-polymerase_dGTP) |
| 186 | 1,00 | 4 | false | 3 | (1 AMP-energy_to_AMP-metabolism) (1 PurM_adenylate-kinase_AMP) (2 PurM_thioredoxin-oxidoreductased_dADP) |
| 187 | 1,00 | 1 | false | 1 | (1 PurM_thioredoxin-oxidoreductase_dCTP) |
| 188 | 1,00 | 1 | false | 1 | (1 AS_Tryptophan_to_Tryptamine) |
| 189 | 1,00 | 3 | false | 3 | (1 AS_Acetyl-CoA_to_L-Leucine) (-1 TCA_citrate-hydro-lyase) (1 TCA_citrate_synthase) |
| 190 | 1,00 | 1 | false | 1 | (1 AS_Homoserine_to_Threonine) |
| 191 | 1,00 | 1 | false | 1 | (1 PurM_5-nucleotidase_CMP) |
| 192 | 1,00 | 1 | false | 1 | (1 PyrM_cytidine-kinase_UTP) |
| 193 | 1,00 | 1 | false | 1 | (1 PyrM_uridine-kinase_dUTP) |
| 194 | 1,00 | 1 | false | 1 | (1 PyrM_uridine-kinase_ITP) |
| 195 | 0,56 | 1 | false | 1 | (1 PyrM_cytidine-kinase_dATP) |
| 196 | 1,00 | 1 | false | 1 | (1 SERP2060-glyerol-transport_import) |
| 197 | 1,00 | 1 | false | 1 | (1 PyrM_uridine-kinase_GTP) |

^1^ This data shows the elementary mode Analysis for *S. epidermidis* RP62A with 0.16µMIQ-143.

Table S12: Extreme Modes of *S. epidermidis* RP62A with 1.25µM IQ-143^1^:

| # | Activity | Flux sum | Reversible? | Pathlength | Reactions |
| --- | --- | --- | --- | --- | --- |
| 1 | 1,00 | 1 | true | 1 | (1 AS_Alanine_to_Pyruvate) |
| 2 | 1,00 | 1 | true | 1 | (1 PyrM_dCMP-aminohydrolase) |
| 3 | -0,67 | 1 | true | 1 | (1 PurM_ATP_IDP-phosphotransferase) |
| 4 | 1,00 | 1 | true | 1 | (1 PyrM_cytidilate-kinase_dCMP) |
| 5 | 1,00 | 1 | true | 1 | (1 PyrM_dUMP-phosphotransferase) |
| 6 | 1,00 | 1 | true | 1 | (1 PurM_ATP_GMP-guanylate-kinase) |
| 7 | 1,00 | 1 | true | 1 | (1 PyrM_cytidine-aminohydrolase) |
| 8 | 1,00 | 1 | true | 1 | (1 PurM_ATP_UTP-phosphotransferase) |
| 9 | 1,00 | 1 | true | 1 | (1 PurM_ATP_GTP-phosphotransferase) |
| 10 | 1,00 | 2 | true | 2 | (-1 PurM_nucleotide-phosphatase_Deoxyguanosine) (1 PyrM_deoxyguanosine-phosphorylase) |
| 11 | 1,00 | 2 | true | 2 | (-1 PyrM_dihydroorotase) (1 PyrM_dihydroorotate-oxidase) |
| 12 | 1,00 | 1 | true | 1 | (1 PurM_ATP_dIDP-phosphotransferase) |
| 13 | 1,00 | 1 | true | 1 | (1 PyrM_thymidine-kinase_dTMP) |
| 14 | 1,00 | 1 | true | 1 | (1 PurM_xanthosine-phosphoribosyltransferase) |
| 15 | 1,00 | 1 | true | 1 | (1 PurM_ATP_dUDP-phosphotransferase) |
| 16 | 1,00 | 1 | true | 1 | (1 TCA_fumarate-hydratase) |
| 17 | 1,01 | 3 | true | 3 | (1 PurM_IMP-pyrophosphorylase) (-1 PurM_nucleotide-phosphatase_Inosine) (1 PyrM_uridine-phosphorylase) |
| 18 | 1,00 | 3 | true | 3 | (1 PurM_IMP-pyrophosphorylase) (1 PurM_nucleotide-phosphatase_Guanosine) (-1 PurM_nucleotide-phosphatase_Inosine) |
| 19 | 0,39 | 3 | true | 3 | (1 PurM_IMP-pyrophosphorylase) (-1 PurM_nucleotide-phosphatase_Inosine) (1 PurM_nucleotide-phosphatase_Xanthosine) |
| 20 | 1,00 | 3 | true | 3 | (1 PurM_IMP-pyrophosphorylase) (-1 PurM_nucleotide-phosphatase_Deoxyinosine) (1 PyrM_deoxyinosine-phosphorylase) |
| 21 | 1,00 | 1 | true | 1 | (1 AS_Serine_to_Glycine) |
| 22 | 1,00 | 1 | true | 1 | (1 SERP1951-lipoprotein-transport_efflux/import) |
| 23 | 1,00 | 1 | true | 1 | (1 PurM_GMP-pyrophosphorylase2) |
| 24 | 1,00 | 1 | true | 1 | (1 PyrM_nucleoside-phosphate-kinase_ATP) |
| 25 | -0,52 | 1 | true | 1 | (1 PyrM_ATP_dTDP_thymidylate-kinase) |
| 26 | 1,00 | 1 | true | 1 | (1 PurM_ATP_dTDP-phosphotransferase) |
| 27 | 1,00 | 1 | true | 1 | (1 PurM_ATP_GMP_guanylate-kinase) |
| 28 | 1,00 | 1 | true | 1 | (1 PyrM_nucleoside-phosphate-kinase_ATP2) |
| 29 | 1,00 | 2 | true | 2 | (-1 PurM_nucleotide-phosphatase_Deoxyuridine) (1 PyrM_deoxyuridine-phosphorylase) |
| 30 | -1,33 | 2 | true | 2 | (1 PyrM_deoxyuridine-phosphorylase) (-1 PyrM_thymidine-kinase_dUMP) |
| 31 | 1,00 | 1 | true | 1 | (1 PurM_ATP_dGDP-phosphotransferase) |
| 32 | 1,00 | 1 | true | 1 | (1 AS_Aspartate_to_Homoserine) |
| 33 | 1,00 | 1 | true | 1 | (1 PurM_ATP_dADP-phosphotransferase) |
| 34 | 1,00 | 2 | true | 2 | (1 TCA_citrate-hydro-lyase) (-1 TCA_citrate-hydroxymutase) |
| 35 | 1,00 | 3 | true | 3 | (1 Glyc_lipoic_acetyltransferase) (-1 TCA_citrate-hydro-lyase) (1 TCA_citrate_synthase) |
| 36 | 1,00 | 7 | true | 6 | (-1 Glyc_acetaldehyde-dehydrogenase_NAD+) (-1 Glyc_Actetate-CoA-ligase) (-1 SERP0389-Glyc_Ethanol_NAD+-oxidoreductase) (2 SERP2156-Glyc_L-lactate-dehydrogenase) (-1 TCA_citrate-hydro-lyase) (1 TCA_citrate_synthase) |
| 37 | 1,00 | 1 | true | 1 | (1 PurM_ATP_CDP-phosphotransferase) |
| 38 | 1,00 | 1 | true | 1 | (1 AS_Saccharopine_to_Lysine) |
| 39 | 1,00 | 1 | true | 1 | (1 PurM_IMP_L-aspartate-ligase) |
| 40 | 0,75 | 1 | true | 1 | (1 Glyc_Succinate-CoA-ligase) |
| 41 | 0,75 | 3 | true | 3 | (1 AS_Acetyl-CoA_to_L-Valine) (-1 TCA_citrate-hydro-lyase) (1 TCA_citrate_synthase) |
| 42 | 1,00 | 1 | true | 1 | (1 PyrM_orotate-phosphoribosyltransferase) |
| 43 | 1,00 | 1 | true | 1 | (1 PurM_metaphosphatase) |
| 44 | 1,11 | 1 | true | 1 | (1 PyrM_cytidilate-kinase_CTP) |
| 45 | 0,67 | 1 | true | 1 | (1 PurM_GMP-pyrophosphorylase) |
| 46 | 1,00 | 3 | true | 3 | (1 Glyc_alpha-D-Glucose-6-phosphate-ketol-isomerase) (-1 Glyc_alpha-D-Glucose-6-phosphate-ketol-isomerase2) (1 Glyc_beta-D-Glucose-6-phosphate-ketol-isomerase) |
| 47 | 1,00 | 6 | true | 6 | (1 ATP-energy_to_ATP-metabolism) (1 Glyc_alpha-D-Glucose-6-phosphate-ketol-isomerase2) (1 Glyc_ATP-alpha-D-glucokinase) (-1 Glyc_ATP-beta-D-glucokinase) (-1 Glyc_beta-D-Glucose-6-phosphate-ketol-isomerase) (-1 Glyc_D-Glucose-1-epimerase) |
| 48 | 1,12 | 1 | true | 1 | (1 Glyc_PTS-permease2) |
| 49 | 1,00 | 4 | true | 4 | (-1 Glyc_6-phospho-beta-glucosidase) (1 Glyc_alpha-D-Glucose-6-phosphate-ketol-isomerase2) (-1 Glyc_beta-D-Glucose-6-phosphate-ketol-isomerase) (1 Glyc_PTS-permease1) |
| 50 | 1,00 | 1 | true | 1 | (1 PyrM_thymidine-phosphorylase) |
| 51 | 1,00 | 1 | true | 1 | (1 PurM_ATP_dCDP-phosphotransferase) |
| 52 | -1,74 | 1 | true | 1 | (1 PyrM_UMP-pyrophosphorylase) |
| 53 | 0,79 | 1 | true | 1 | (1 PyrM_nucleoside-triphosphate-adenylate-kinase) |
| 54 | 0,53 | 1 | true | 1 | (1 PurM_XMP-pyrophosphorylase) |
| 55 | 0,53 | 1 | true | 1 | (1 TCA_succinate-dehydrogenase) |
| 56 | 0,53 | 2 | true | 2 | (1 PyrM_Deoxycytidine-aminohydrolase) (-1 PyrM_Deoxycytidine-deaminase) |
| 57 | 0,53 | 2 | true | 2 | (-1 PurM_nucleotide-phosphatase_Deoxyadenosine) (1 PyrM_deoxyadenosine-phosphorylase) |
| 58 | 0,53 | 1 | true | 1 | (1 PyrM_ATP_dUDP_thymidylate-kinase) |
| 59 | 0,92 | 1 | true | 1 | (1 PurM_adenylate-kinase_dAMP) |
| 60 | 1,08 | 2 | true | 2 | (1 AMP-energy_to_AMP-metabolism) (1 PurM_AMP-pyrophosphorylase2) |
| 61 | -0,92 | 2 | true | 2 | (-1 AMP-energy_to_AMP-metabolism) (1 PurM_adenylosuccinate-lyase) |
| 62 | 0,92 | 2 | true | 2 | (1 AMP-energy_to_AMP-metabolism) (1 PurM_AMP-pyrophosphorylase) |
| 63 | 0,45 | 14 | true | 8 | (1 AMP-energy_to_AMP-metabolism) (2 Glyc_alpha-D-Glucose-6-phosphate-ketol-isomerase2) (2 Glyc_ATP-alpha-D-glucokinase) (-2 Glyc_ATP-beta-D-glucokinase) (-2 Glyc_beta-D-Glucose-6-phosphate-ketol-isomerase) (-2 Glyc_D-Glucose-1-epimerase) (1 PurM_adenylate-kinase_AMP) (2 PurM_nucleoside-diphosphate-phosphotransferase_ATP) |
| 64 | 1,00 | 1 | true | 1 | (1 PurM_adenylylsulfate-kinase) |
| 65 | 1,00 | 4 | true | 3 | (2 ADP-energy_to_ADP-metabolism) (-1 AMP-energy_to_AMP-metabolism) (-1 PurM_adenylate-kinase_AMP) |
| 66 | 1,00 | 3 | true | 3 | (1 PurM_IMP-pyrophosphorylase) (1 PurM_nucleotide-phosphatase_Adenine) (-1 PurM_nucleotide-phosphatase_Inosine) |
| 67 | 1,00 | 3 | true | 3 | (1 PurM_IMP-pyrophosphorylase) (-1 PurM_nucleotide-phosphatase_Inosine) (1 PyrM_pyrimidine-nucleoside-phosphorylase) |
| 68 | 1,00 | 1 | true | 1 | (1 TCA_isocitrate-hydro-lyase) |
| 69 | 1,00 | 14 | false | 8 | (-1 AMP-energy_to_AMP-metabolism) (-2 Glyc_alpha-D-Glucose-6-phosphate-ketol-isomerase2) (-2 Glyc_ATP-alpha-D-glucokinase) (2 Glyc_ATP-beta-D-glucokinase) (2 Glyc_beta-D-Glucose-6-phosphate-ketol-isomerase) (2 Glyc_D-Glucose-1-epimerase) (-1 PurM_adenylate-kinase_AMP) (2 PurM_ATP-phosphohydrolase) |
| 70 | 1,00 | 2 | false | 2 | (1 AMP-energy_to_AMP-metabolism) (1 PurM_5-nucleotidase_AMP) |
| 71 | 1,00 | 1 | false | 1 | (1 PyrM_uridine-kinase_dGTP) |
| 72 | 1,00 | 4 | false | 3 | (1 AMP-energy_to_AMP-metabolism) (1 PurM_adenylate-kinase_AMP) (2 SERP0841-PurM_PNPase_ADP) |
| 73 | 1,00 | 1 | false | 1 | (1 PurM_5-nucleotidase_XMP) |
| 74 | 2,62 | 1 | false | 1 | (1 SERP0831-PurM_DNA-directed-DNA-polymerase_dATP) |
| 75 | 1,00 | 1 | false | 1 | (1 PyrM_uridine-kinase_dTTP) |
| 76 | 1,00 | 1 | false | 1 | (1 PyrM_cytidine-kinase_dCTP) |
| 77 | 2,07 | 1 | false | 1 | (1 SERP1952-macrolide-transport_efflux) |
| 78 | 1,00 | 2 | false | 2 | (1 PurM_deoxyadenosine-kinase_ATP) (-1 PyrM_deoxyadenosine-phosphorylase) |
| 79 | 0,28 | 1 | false | 1 | (1 PyrM_dUTP-diphosphatase) |
| 80 | 1,00 | 1 | false | 1 | (1 AS_Aspartate_to_beta-Alanine) |
| 81 | 1,00 | 1 | false | 1 | (1 SERP1803-cobalt/nickel-transport_efflux) |
| 82 | 1,00 | 1 | false | 1 | (1 PyrM_cytidine-kinase_dGTP) |
| 83 | 1,00 | 1 | false | 1 | (1 PurM_thioredoxin-oxidoreductase_dUTP) |
| 84 | 3,07 | 1 | false | 1 | (1 SERP0292-iron-dicitrate-transporter_import) |
| 85 | 0,25 | 2 | false | 2 | (1 Glyc_6-phosphofructokinase) (1 Glyc_fructose-bisphosphatase) |
| 86 | 1,00 | 1 | false | 1 | (1 PurM_XMP_L-glutamine-amide-ligase) |
| 87 | 1,00 | 1 | false | 1 | (1 AS_Aspartate_to_Arginine) |
| 88 | 1,00 | 7 | false | 6 | (1 Glyc_acetaldehyde-dehydrogenase_NAD+) (1 Glyc_Actetate-CoA-ligase) (2 PyrM_thioredoxin-reductase) (1 SERP0389-Glyc_Ethanol_NAD+-oxidoreductase) (1 TCA_citrate-hydro-lyase) (-1 TCA_citrate_synthase) |
| 89 | 1,00 | 20 | false | 15 | (-2 Glyc_2-Phospho-D-glycerate-2.3-phosphomutase) (2 Glyc_2-phospho-D-glycerate-hydro-lyase) (1 Glyc_6-phospho-beta-glucosidase) (1 Glyc_6-phosphofructokinase) (-1 Glyc_acetaldehyde-dehydrogenase_NAD+) (-1 Glyc_Actetate-CoA-ligase) (1 Glyc_beta-D-Glucose-6-phosphate-ketol-isomerase) (-1 Glyc_D-Glucose-1-epimerase-ketol-isomerase) (1 Glyc_fructose-bisphosphat-aldolase) (2 Glyc_glyceraldehyde-3-P-dehydrogenase_NAD+) (-2 Glyc_phosphoglycerate-kinase) (-1 SERP0389-Glyc_Ethanol_NAD+-oxidoreductase) (-1 TCA_citrate-hydro-lyase) (1 TCA_citrate_synthase) (-2 TCA_PEP-carboxylase) |
| 90 | 0,96 | 1 | false | 1 | (1 PyrM_2,3-cyclic-nucleotidase_UMP) |
| 91 | 1,00 | 1 | false | 1 | (1 DNA-extern_to_DNA-intern) |
| 92 | 0,67 | 1 | false | 1 | (1 SERP0831-PurM_DNA-directed-DNA-polymerase_dCTP) |
| 93 | 0,17 | 1 | false | 1 | (1 SERP0841-PurM_PNPase_GDP) |
| 94 | 1,09 | 2 | false | 2 | (1 PurM_carbamate-kinase_ATP) (1 PyrM_aspartate-carbamoyltransferase) |
| 95 | 1,00 | 1 | false | 1 | (1 PurM_thioredoxin-oxidoreductase_dGDP) |
| 96 | 0,48 | 1 | false | 1 | (1 PurM_ITP-diphosphohydrolase) |
| 97 | 0,69 | 13 | false | 8 | (-1 AMP-energy_to_AMP-metabolism) (-2 Glyc_alpha-D-Glucose-6-phosphate-ketol-isomerase2) (-2 Glyc_ATP-alpha-D-glucokinase) (2 Glyc_ATP-beta-D-glucokinase) (2 Glyc_beta-D-Glucose-6-phosphate-ketol-isomerase) (2 Glyc_D-Glucose-1-epimerase) (-1 PurM_adenylate-kinase_AMP) (1 SERP0688-spermidine/putrescine-transport_import) |
| 98 | 1,00 | 3 | false | 3 | (1 PurM_5-nucleotidase_dCMP) (1 PyrM_Deoxycytidine-aminohydrolase) (1 PyrM_deoxyuridine-phosphorylase) |
| 99 | 1,97 | 14 | false | 8 | (-1 AMP-energy_to_AMP-metabolism) (-2 Glyc_alpha-D-Glucose-6-phosphate-ketol-isomerase2) (-2 Glyc_ATP-alpha-D-glucokinase) (2 Glyc_ATP-beta-D-glucokinase) (2 Glyc_beta-D-Glucose-6-phosphate-ketol-isomerase) (2 Glyc_D-Glucose-1-epimerase) (-1 PurM_adenylate-kinase_AMP) (2 SERP0686-spermidine/putrescine-transport_import) |
| 100 | 0,01 | 1 | false | 1 | (1 PyrM_dUTP-diphosphohydrolase) |
| 101 | 1,00 | 1 | false | 1 | (1 PurM_dITP-diphosphohydrolase) |
| 102 | 0,08 | 2 | false | 2 | (1 PurM_pyruvate-phosphotransferase_GTP) (-1 TCA_PEP-carboxylase) |
| 103 | 2,30 | 2 | false | 2 | (1 PurM_pyruvate-phosphotransferase_dATP) (-1 TCA_PEP-carboxylase) |
| 104 | 1,00 | 1 | false | 1 | (1 PurM_5-nucleotidase_UMP) |
| 105 | 1,00 | 1 | false | 1 | (1 SERP2283-phopsphonate-transport_import) |
| 106 | 1,00 | 2 | false | 2 | (1 PurM_GDP-reductase) (1 SERP2179-choline/betaine/carnitine-transp_efflux) |
| 107 | 1,48 | 1 | false | 1 | (1 AS_Valine) |
| 108 | 1,00 | 2 | false | 2 | (1 PurM_5-nucleotidase_dAMP) (1 PyrM_deoxyadenosine-phosphorylase) |
| 109 | 0,96 | 1 | false | 1 | (1 SERP1802-cobalt/nickel-transport_efflux) |
| 110 | 1,00 | 1 | false | 1 | (1 PurM_DNA-directed-RNA-polyermase_UTP) |
| 111 | 1,00 | 4 | false | 4 | (-1 TCA_lipoic-transsuccinylase) (1 TCA_oxoglutarate-dehydrogenase-complex1) (1 TCA_oxoglutarate-dehydrogenase-complex2) (1 TCA_oxoglutarate-synthase) |
| 112 | 2,06 | 1 | false | 1 | (1 PyrM_cytidine-kinase_dTTP) |
| 113 | 1,00 | 1 | false | 1 | (1 PurM_UTP-diphosphohydrolase) |
| 114 | 1,00 | 1 | false | 1 | (1 AS_Serine_to_Methionine) |
| 115 | 1,00 | 2 | false | 2 | (1 PurM_5-nucleotidase_dGMP) (1 PyrM_deoxyguanosine-phosphorylase) |
| 116 | 0,00 | 2 | false | 2 | (-1 AMP-energy_to_AMP-metabolism) (1 PurM_deoxycytidine-kinase_ATP) |
| 117 | 0,00 | 1 | false | 1 | (1 PurM_thioredoxin-oxidoreductase_dGTP) |
| 118 | 1,00 | 3 | false | 3 | (1 IQ-143-extern_to_IQ-) (1 SERP1944-MultiDrug-transport_efflux) (1 SERP2179-choline/betaine/carnitine-transp_efflux) |
| 119 | 0,89 | 1 | false | 1 | (1 AS_Leucine) |
| 120 | 0,39 | 20 | false | 15 | (-2 Glyc_2-Phospho-D-glycerate-2.3-phosphomutase) (2 Glyc_2-phospho-D-glycerate-hydro-lyase) (1 Glyc_6-phospho-beta-glucosidase) (1 Glyc_6-phosphofructokinase) (-1 Glyc_acetaldehyde-dehydrogenase_NAD+) (-1 Glyc_Actetate-CoA-ligase) (1 Glyc_beta-D-Glucose-6-phosphate-ketol-isomerase) (-1 Glyc_D-Glucose-1-epimerase-ketol-isomerase) (1 Glyc_fructose-bisphosphat-aldolase) (2 Glyc_glyceraldehyde-3-P-dehydrogenase_NADP+) (-2 Glyc_phosphoglycerate-kinase) (-1 SERP0389-Glyc_Ethanol_NAD+-oxidoreductase) (-1 TCA_citrate-hydro-lyase) (1 TCA_citrate_synthase) (-2 TCA_PEP-carboxylase) |
| 121 | 0,28 | 1 | false | 1 | (1 PyrM_2,3-cyclic-nucleotidase_CMP) |
| 122 | 0,20 | 2 | false | 2 | (1 SERP1997-formate/nitrite-transport_efflux/import) (1 SERP2179-choline/betaine/carnitine-transp_efflux) |
| 123 | 1,00 | 1 | false | 1 | (1 PyrM_CTP-synthase) |
| 124 | 0,80 | 2 | false | 2 | (1 IQ-143-extern_to_IQ-) (1 SERP2289-MultiDrug-transport_efflux) |
| 125 | 1,00 | 1 | false | 1 | (1 AS_Glutamate_to_Proline) |
| 126 | 0,72 | 1 | false | 1 | (1 PyrM_uridine-kinase_dCTP) |
| 127 | 1,12 | 1 | false | 1 | (1 PyrM_uridine-kinase_UTP) |
| 128 | 1,00 | 3 | false | 3 | (1 PurM_deoxycytidine-kinase_ATP2) (-1 PyrM_Deoxycytidine-aminohydrolase) (-1 PyrM_deoxyuridine-phosphorylase) |
| 129 | 1,00 | 1 | false | 1 | (1 AS_Threonine) |
| 130 | 1,40 | 1 | false | 1 | (1 SERP0831-PurM_DNA-directed-DNA-polymerase_dTTP) |
| 131 | 1,00 | 1 | false | 1 | (1 PurM_GTP-diphosphohydrolase) |
| 132 | 1,00 | 1 | false | 1 | (1 PyrM_uridine-kinase_dATP) |
| 133 | 0,44 | 1 | false | 1 | (1 AS_Serine_to_Pyruvate) |
| 134 | 1,00 | 2 | false | 2 | (1 SERP0290-zinc-transport_efflux) (1 SERP0291-zinc-transporter_import) |
| 135 | 1,00 | 1 | false | 1 | (1 AS_Isoleucine) |
| 136 | 1,00 | 1 | false | 1 | (1 PurM_thioredoxin-oxidoreductase_dCDP) |
| 137 | 0,83 | 2 | false | 2 | (1 PurM_pyruvate-phosphotransferase_dGTP) (-1 TCA_PEP-carboxylase) |
| 138 | 1,00 | 1 | false | 1 | (1 PurM_5-nucleotidase_dTMP) |
| 139 | 0,77 | 1 | false | 1 | (1 PyrM_uridine-kinase_ATP) |
| 140 | 0,00 | 4 | false | 4 | (1 PurM_D-Ribose-1,5-phosphomutase) (-1 PurM_IMP-pyrophosphorylase) (1 PurM_nucleotide-phosphatase_Inosine) (1 PurM_PRPP-synthetase) |
| 141 | 1,49 | 1 | false | 1 | (1 PurM_DNA-directed-RNA-polyermase_GTP) |
| 142 | 1,00 | 7 | false | 6 | (-1 Glyc_acetaldehyde-dehydrogenase_NAD+) (-1 Glyc_Actetate-CoA-ligase) (2 Glyc_dihydrolipoamide-dehydrogenase) (-1 SERP0389-Glyc_Ethanol_NAD+-oxidoreductase) (-1 TCA_citrate-hydro-lyase) (1 TCA_citrate_synthase) |
| 143 | 2,87 | 1 | false | 1 | (1 PyrM_OMP-decarboxylase) |
| 144 | 1,00 | 1 | false | 1 | (1 AS_Glutamate_to_Glutamine) |
| 145 | 2,20 | 1 | false | 1 | (1 PurM_urea-amidohydrolase) |
| 146 | 2,20 | 1 | false | 1 | (1 AS_Aspartate_to_Asparagine) |
| 147 | 2,20 | 1 | false | 1 | (1 PyrM_cytidine-kinase_ATP) |
| 148 | 0,00 | 2 | false | 2 | (1 PurM_GDP-reductase) (1 SERP0765-Uracil-permease-transport_import) |
| 149 | 2,69 | 3 | false | 3 | (1 IQ-143-extern_to_IQ-) (1 SERP0765-Uracil-permease-transport_import) (1 SERP1944-MultiDrug-transport_efflux) |
| 150 | 0,00 | 2 | false | 2 | (1 SERP0765-Uracil-permease-transport_import) (1 SERP1997-formate/nitrite-transport_efflux/import) |
| 151 | 1,23 | 13 | false | 9 | (-1 Glyc_acetaldehyde-dehydrogenase_NAD+) (-1 Glyc_Actetate-CoA-ligase) (-1 SERP0389-Glyc_Ethanol_NAD+-oxidoreductase) (-1 TCA_citrate-hydro-lyase) (1 TCA_citrate_synthase) (-2 TCA_lipoic-transsuccinylase) (2 TCA_Oxidoreductase) (2 TCA_oxoglutarate-dehydrogenase-complex1) (2 TCA_oxoglutarate-dehydrogenase-complex2) |
| 152 | 1,00 | 1 | false | 1 | (1 PurM_XTP-diphosphohydrolase) |
| 153 | 1,00 | 3 | false | 3 | (-1 PurM_carbamate-kinase_ATP) (1 PyrM_CO2_L-glutamine-amido-ligase) (-1 TCA_Pyruvate_CO2-ligase) |
| 154 | 0,43 | 12 | false | 7 | (2 Glyc_acetaldehyde-dehydrogenase_NAD+) (2 Glyc_Actetate-CoA-ligase) (1 OP_complex1) (1 OP_complex3) (2 SERP0389-Glyc_Ethanol_NAD+-oxidoreductase) (2 TCA_citrate-hydro-lyase) (-2 TCA_citrate_synthase) |
| 155 | 0,17 | 17 | false | 7 | (3 Glyc_acetaldehyde-dehydrogenase_NAD+) (3 Glyc_Actetate-CoA-ligase) (1 OP_complex2) (1 OP_complex3) (3 SERP0389-Glyc_Ethanol_NAD+-oxidoreductase) (3 TCA_citrate-hydro-lyase) (-3 TCA_citrate_synthase) |
| 156 | 1,00 | 1 | false | 1 | (1 PurM_GTP-pyrophosphokinase) |
| 157 | 1,00 | 6 | false | 6 | (1 Glyc_acetaldehyde-dehydrogenase_NAD+) (1 Glyc_Actetate-CoA-ligase) (1 OP_complex4) (1 SERP0389-Glyc_Ethanol_NAD+-oxidoreductase) (1 TCA_citrate-hydro-lyase) (-1 TCA_citrate_synthase) |
| 158 | 1,60 | 2 | false | 2 | (1 Glyc_pyruvate_dehydrogenase) (1 TCA_pyruvate_dehydrogenase) |
| 159 | 1,00 | 17 | false | 6 | (-3 Glyc_acetaldehyde-dehydrogenase_NAD+) (-3 Glyc_Actetate-CoA-ligase) (2 OP_complex5) (-3 SERP0389-Glyc_Ethanol_NAD+-oxidoreductase) (-3 TCA_citrate-hydro-lyase) (3 TCA_citrate_synthase) |
| 160 | 1,00 | 1 | false | 1 | (1 AS_Aspartate_to_Alanine) |
| 161 | 0,26 | 2 | false | 2 | (1 AS_Serine_to_Cysteine) (1 Glyc_Actetate-CoA-ligase) |
| 162 | 1,00 | 4 | false | 3 | (1 AMP-energy_to_AMP-metabolism) (1 PurM_adenylate-kinase_AMP) (2 PurM_thioredoxin-oxidoreductase_dATP) |
| 163 | 1,00 | 1 | false | 1 | (1 SERP2186-PurM_ATP_sulfate-adenylyltransferase) |
| 164 | 1,00 | 3 | false | 3 | (-1 AMP-energy_to_AMP-metabolism) (1 PurM_ADP-ribose-ribophosphohydrolase) (1 PurM_PRPP-synthetase) |
| 165 | 1,00 | 2 | false | 2 | (1 IQ-143-extern_to_IQ-) (1 SERP1403-MultiDrug-transport_efflux) |
| 166 | 0,25 | 13 | false | 3 | (1 FA_Syn_Acetyl-CoA_to_C16) (-6 TCA_citrate-hydro-lyase) (6 TCA_citrate_synthase) |
| 167 | 1,58 | 1 | false | 1 | (1 PyrM_UTP_L-glutamine-amido-ligase) |
| 168 | 0,25 | 1 | false | 1 | (1 AS_Phenylalanin_to_Tyrosine) |
| 169 | 1,00 | 1 | false | 1 | (1 PurM_5-nucleotidase_IMP) |
| 170 | 1,00 | 2 | false | 2 | (1 PurM_GDP-reductase) (1 PurM_IMP-dehydrogenase) |
| 171 | 0,00 | 3 | false | 3 | (1 IQ-143-extern_to_IQ-) (1 PurM_IMP-dehydrogenase) (1 SERP1944-MultiDrug-transport_efflux) |
| 172 | 0,88 | 2 | false | 2 | (1 PurM_IMP-dehydrogenase) (1 SERP1997-formate/nitrite-transport_efflux/import) |
| 173 | 0,55 | 1 | false | 1 | (1 PurM_5-nucleotidase_GMP) |
| 174 | 0,28 | 1 | false | 1 | (1 PyrM_cytidine-kinase_ITP) |
| 175 | 1,00 | 1 | false | 1 | (1 AS_Histidine_to_Glutamate) |
| 176 | 1,00 | 1 | false | 1 | (1 PyrM_cytidine-kinase_GTP) |
| 177 | 1,00 | 13 | false | 3 | (1 FA_Deg_C16_to_Acetyl-CoA) (6 TCA_citrate-hydro-lyase) (-6 TCA_citrate_synthase) |
| 178 | 1,00 | 16 | false | 9 | (-1 AMP-energy_to_AMP-metabolism) (-2 Glyc_alpha-D-Glucose-6-phosphate-ketol-isomerase2) (-2 Glyc_ATP-alpha-D-glucokinase) (2 Glyc_ATP-beta-D-glucokinase) (2 Glyc_beta-D-Glucose-6-phosphate-ketol-isomerase) (2 Glyc_D-Glucose-1-epimerase) (-1 PurM_adenylate-kinase_AMP) (2 PurM_pyruvate-phosphotransferase_ATP) (-2 TCA_PEP-carboxylase) |
| 179 | 1,00 | 1 | false | 1 | (1 PyrM_cytidine-kinase_dUTP) |
| 180 | 1,00 | 6 | false | 6 | (-1 Glyc_alpha-D-Glucose-6-phosphate-ketol-isomerase2) (-1 Glyc_ATP-alpha-D-glucokinase) (1 Glyc_ATP-beta-D-glucokinase) (1 Glyc_beta-D-Glucose-6-phosphate-ketol-isomerase) (1 Glyc_D-Glucose-1-epimerase) (1 PurM_DNA-directed-RNA-polymerase_ATP) |
| 181 | 1,00 | 13 | false | 8 | (-1 AMP-energy_to_AMP-metabolism) (-2 Glyc_alpha-D-Glucose-6-phosphate-ketol-isomerase2) (-2 Glyc_ATP-alpha-D-glucokinase) (2 Glyc_ATP-beta-D-glucokinase) (2 Glyc_beta-D-Glucose-6-phosphate-ketol-isomerase) (2 Glyc_D-Glucose-1-epimerase) (-1 PurM_adenylate-kinase_AMP) (1 SERP0687-spermidine/putrescine-transport_import) |
| 182 | 0,41 | 1 | false | 1 | (1 PurM_thioredoxin-oxidoreductase_dUDP) |
| 183 | 0,48 | 1 | false | 1 | (1 PurM_dGTP-diphosphohydrolase) |
| 184 | 1,00 | 1 | false | 1 | (1 PurM_DNA-directed-RNA-polyermase_CTP) |
| 185 | 1,00 | 1 | false | 1 | (1 SERP0831-PurM_DNA-directed-DNA-polymerase_dGTP) |
| 186 | 1,00 | 4 | false | 3 | (1 AMP-energy_to_AMP-metabolism) (1 PurM_adenylate-kinase_AMP) (2 PurM_thioredoxin-oxidoreductased_dADP) |
| 187 | 1,00 | 1 | false | 1 | (1 PurM_thioredoxin-oxidoreductase_dCTP) |
| 188 | 1,00 | 1 | false | 1 | (1 AS_Tryptophan_to_Tryptamine) |
| 189 | 1,00 | 3 | false | 3 | (1 AS_Acetyl-CoA_to_L-Leucine) (-1 TCA_citrate-hydro-lyase) (1 TCA_citrate_synthase) |
| 190 | 1,00 | 1 | false | 1 | (1 AS_Homoserine_to_Threonine) |
| 191 | 1,00 | 1 | false | 1 | (1 PurM_5-nucleotidase_CMP) |
| 192 | 2,68 | 1 | false | 1 | (1 PyrM_cytidine-kinase_UTP) |
| 193 | 1,00 | 1 | false | 1 | (1 PyrM_uridine-kinase_dUTP) |
| 194 | 1,00 | 1 | false | 1 | (1 PyrM_uridine-kinase_ITP) |
| 195 | 0,56 | 1 | false | 1 | (1 PyrM_cytidine-kinase_dATP) |
| 196 | 1,00 | 1 | false | 1 | (1 SERP2060-glyerol-transport_import) |
| 197 | 0,49 | 1 | false | 1 | (1 PyrM_uridine-kinase_GTP) |

^1^ This data shows the elementary mode Analysis for *S. epidermidis* RP62A with 1.25µM IQ-143.

Table S13: Extreme Modes of *H. sapiens* without IQ-143^1^:

| # | Activity | Flux sum | Reversible? | Pathlength | Net reaction |
| --- | --- | --- | --- | --- | --- |
| 1 | 0.01655153401230347 | 1 | true | 1 | H2O + P1P4-Bis(5-adenosyl)tetraphosphate = AMP-energy + ATP-energy |
| 2 | 0.9718829672703104 | 2 | true | 2 | ADP-energy + GTP = ATP-energy + GDP |
| 3 | 0.03494402270226615 | 1 | true | 1 | Orthophosphate + Uridine = Uracil + alpha-D-Ribose1-phosphate |
| 4 | 0.015529980226903106 | 1 | true | 1 | ATP-energy + UMP = ADP-energy + UDP |
| 5 | 0.01125584770243826 | 1 | true | 1 | ATP-energy + GMP = ADP-energy + GDP |
| 6 | 6.774006132614918E-4 | 3 | true | 3 | ATP-energy + CDP + RNA-U = ADP-energy + RNA-C + UTP |
| 7 | 0.025167558640849963 | 3 | true | 3 | ATP-energy + CDP + RNA-G = ADP-energy + GTP + RNA-C |
| 8 | 0.028076999689041093 | 1 | true | 1 | ATP-energy + UMP = ADP-energy + UDP |
| 9 | 5.899637709720906E-4 | 1 | true | 1 | ATP-metabolism = ATP-energy |
| 10 | 0.018001729088897522 | 1 | true | 1 | Orotidine5-phosphate = CO2 + UMP |
| 11 | 0.01554561791018938 | 2 | true | 2 | ATP-energy + H2O = ADP-energy + Orthophosphate |
| 12 | 0.029944097937886216 | 1 | true | 1 | AMP-metabolism = AMP-energy |
| 13 | 0.9527511006480274 | 4 | true | 4 | ADP-energy + GTP = ATP-energy + GDP |
| 14 | 0.9876120638225723 | 4 | true | 4 | 2 ATP-energy + HCO3- + Pyruvate = 2 ADP-energy + CO2 + Orthophosphate + Phosphoenolpyruvate |
| 15 | 0.014188190744323315 | 2 | true | 2 | no net reaction |
| 16 | 0.02118596232367509 | 1 | true | 1 | Pyrophosphate + UMP = 5-Phospho-alpha-D-ribose1-diphosphate + Uracil |
| 17 | 0.010106457228134413 | 1 | true | 1 | ATP-energy + CMP = ADP-energy + CDP |
| 18 | 0.009358474654924787 | 1 | true | 1 | ATP-energy + UDP = ADP-energy + UTP |
| 19 | 0.07943073523980781 | 1 | true | 1 | H2O + P1P3-Bis(5-adenosyl)triphosphate = ADP-energy + AMP-energy |
| 20 | 0.010044051015754452 | 2 | true | 2 | no net reaction |
| 21 | 0.01824437762284048 | 2 | true | 2 | no net reaction |
| 22 | 0.0012509594977667682 | 3 | true | 3 | no net reaction |
| 23 | 0.9602115557760446 | 3 | true | 3 | no net reaction |
| 24 | 0.01645565948549066 | 1 | true | 1 | GMP + Pyrophosphate = 5-Phospho-alpha-D-ribose1-diphosphate + Guanine |
| 25 | 0.0021665356272504432 | 3 | true | 3 | ATP-energy + CDP + RNA-A = ADP-energy + ATP-metabolism + RNA-C |
| 26 | 0.0943099346725943 | 1 | true | 1 | (S)-Malate = Fumarate + H2O |
| 27 | 0.012948684236533947 | 1 | true | 1 | H2O + P1P4-Bis(5-guanosyl)tetraphosphate = GMP + GTP |
| 28 | 0.045555583674236844 | 1 | true | 1 | ATP-energy + GDP = ADP-energy + GTP |
| 29 | 0.013937927079011314 | 1 | true | 1 | AMP-metabolism + ATP-energy = 2 ADP-metabolism |
| 30 | 0.04317061235472264 | 1 | true | 1 | Orotidine5-phosphate + Pyrophosphate = 5-Phospho-alpha-D-ribose1-diphosphate + Orotate |
| 31 | 0.00793872622557823 | 2 | true | 2 | GTP + IMP + L-Aspartate = AMP-energy + Fumarate + GDP + Orthophosphate |
| 32 | 0.02717156409365451 | 2 | true | 2 | no net reaction |
| 33 | 0.005678293834417758 | 2 | true | 2 | no net reaction |
| 34 | 0.009930339633110297 | 1 | true | 1 | ADP-metabolism + ATP-energy = ADP-energy + ATP-metabolism |
| 35 | 0.03209480095504813 | 5 | true | 5 | (S)-Malate + ATP-energy + Pyruvate = (S)-Lactate + ADP-energy + CO2 + Phosphoenolpyruvate |
| 36 | 0.04450111475800855 | 1 | true | 1 | ADP-metabolism = AMP-energy |
| 37 | 0.01989702660635051 | 1 | true | 1 | AMP-energy + UTP = ADP-energy + UDP |
| 38 | 0.01692849626786197 | 1 | true | 1 | H2O + P1P4-Bis(5-uridyl)tetraphosphate = UMP + UTP |
| 39 | 0.06952355118557085 | 5 | true | 5 | (S)-Malate + ATP-energy + Xanthosine5-phosphate = ADP-energy + CO2 + H2O + IMP + Phosphoenolpyruvate |
| 40 | 0.008330465255968367 | 4 | true | 4 | no net reaction |
| 41 | 0.005058992507136817 | 5 | true | 5 | no net reaction |
| 42 | 5.505708648326779E-4 | 4 | true | 4 | 5-Amino-4-imidazolecarboxyamide + 5-Phospho-alpha-D-ribose1-diphosphate + ADP-metabolism + Fumarate + Orthophosphate = ATP-metabolism + Aminoimidazoleribotide + CO2 + L-Aspartate + Pyrophosphate |
| 43 | 0.08181961410412486 | 1 | true | 1 | IMP + Pyrophosphate = 5-Phospho-alpha-D-ribose1-diphosphate + Hypoxanthine |
| 44 | 0.005050457086610116 | 3 | false | 3 | ADP-energy + Pyrophosphate = ATP-energy + Orthophosphate |
| 45 | 0.018167680737062875 | 1 | false | 1 | ADP-energy + Ca2+IN + H2O = AMP-energy + Orthophosphate |
| 46 | 0.015324950057069353 | 4 | false | 4 | AMP-energy + H2O + Pyrophosphate = 5-Phospho-alpha-D-ribose1-diphosphate + Hypoxanthine + NH3 |
| 47 | 4.7720160015451984E-4 | 2 | false | 2 | ATP-energy + H2O = ADP-energy + Orthophosphate |
| 48 | 0.003578665589720975 | 3 | false | 3 | ADP-energy + IQ-143+ GDP + Pyrophosphate + RNA-C = ATP-energy + CDP + IQ-_used + Orthophosphate + RNA-G_blocked |
| 49 | 0.001221154736165464 | 4 | false | 4 | ATP-energy + H2O = ADP-energy + Orthophosphate |
| 50 | 0.009096800791878135 | 2 | false | 2 | ATP-energy + Uridine = ADP-energy + UMP |
| 51 | 0.03951104134964545 | 1 | false | 1 | ATP-energy + IQ-143= ADP-energy + IQ-_deactivated |
| 52 | 0.00523060743484105 | 1 | false | 1 | Ca2+IN + GTP-extern + H2O = GMP + Pyrophosphate |
| 53 | 0.9968666667562996 | 3 | false | 3 | ATP-energy + Uridine = ADP-energy + UMP |
| 54 | 0.0032694373666711796 | 2 | false | 2 | ATP-energy + H2O = ADP-energy + Orthophosphate |
| 55 | 0.0039695467237707005 | 1 | false | 1 | H2O + P1P4-Bis(5-adenosyl)tetraphosphate = 2 ADP-energy |
| 56 | 0.0121455865991974 | 3 | false | 3 | ATP-energy + NH3 + Uridine = ADP-energy + CMP + H2O |
| 57 | 0.01140728480657438 | 2 | false | 2 | ATP-energy + Uridine = ADP-energy + UMP |
| 58 | 0.06216909045003616 | 4 | false | 4 | ATP-energy + NH3 + Uridine = ADP-energy + CMP + H2O |
| 59 | 0.007239691436841333 | 4 | false | 4 | ADP-energy + DNA-C + H2O + Oxidizedthioredoxin + Pyrophosphate = ATP-energy + CDP + DNA-extern + Thioredoxin |
| 60 | 0.016436038228987426 | 2 | false | 2 | GTP + H2O = GMP + Pyrophosphate |
| 61 | 0.0017676334216116807 | 2 | false | 2 | ATP-energy + Uridine = ADP-energy + UMP |
| 62 | 0.010915335683589156 | 2 | false | 2 | ATP-energy + H2O = ADP-energy + Orthophosphate |
| 63 | 0.00806401832991821 | 1 | false | 1 | ATP-metabolism + Ca2+IN + H2O = AMP-energy + Orthophosphate |
| 64 | 0.008111073626983156 | 1 | false | 1 | CDP + Ca2+IN + H2O = CMP + Orthophosphate |
| 65 | 0.00551366953277066 | 2 | false | 2 | ATP-energy + Pyruvate = ADP-energy + Phosphoenolpyruvate |
| 66 | 0.004837438314087672 | 2 | false | 2 | ADP-ribose + ATP-energy + H2O = 5-Phospho-alpha-D-ribose1-diphosphate + 2 AMP-energy |
| 67 | 0.002719567373834275 | 2 | false | 2 | 5-Phospho-alpha-D-ribose1-diphosphate + Guanine + H2O = NH3 + Pyrophosphate + Xanthosine5-phosphate |
| 68 | 0.004433263231509499 | 3 | false | 3 | ADP-energy + Pyrophosphate + RNA-C + UDP = ATP-energy + CDP + Orthophosphate + RNA-U |
| 69 | 0.0017683832496308005 | 11 | false | 11 | ATP-energy + DNA-G + NH3 + Uracil = ADP-energy + DNA-C + Guanine + Orthophosphate |
| 70 | 0.006389732960451755 | 6 | false | 6 | ADP-energy + CO2 + H2O + Phosphoenolpyruvate + Pyrophosphate + Xanthosine5-phosphate = (S)-Malate + 5-Phospho-alpha-D-ribose1-diphosphate + ATP-energy + Urate |
| 71 | 0.03245474140844962 | 1 | false | 1 | Ca2+IN + GDP + H2O = GMP + Orthophosphate |
| 72 | 0.02876252165783144 | 1 | false | 1 | ATP-metabolism + Ca2+IN + H2O = ADP-energy + Orthophosphate |
| 73 | 0.02655006243116209 | 3 | false | 3 | 5-Phospho-alpha-D-ribose1-diphosphate + H2O = Pyrophosphate + alpha-D-Ribose1-phosphate |
| 74 | 0.005829600642677901 | 2 | false | 2 | ATP-energy + Ca2+IN + H2O = ADP-energy + Orthophosphate |
| 75 | 0.015879066073576364 | 6 | false | 6 | 5-Phospho-alpha-D-ribose1-diphosphate + ADP-energy + CO2 + H+ + H2O + Hypoxanthine + Phosphoenolpyruvate = (S)-Malate + ATP-energy + Pyrophosphate + Xanthosine5-phosphate |
| 76 | 0.01305179100730347 | 2 | false | 2 | ATP-energy + Ca2+IN + H2O = ADP-energy + Orthophosphate |
| 77 | 0.0012688045742796916 | 4 | false | 4 | ADP-energy + DNA-G + H2O + Oxidizedthioredoxin + Pyrophosphate = ATP-energy + DNA-extern + GDP + Thioredoxin |
| 78 | 9.123969621090966E-4 | 2 | false | 2 | 2 ATP-energy + Sulfate = 3-Phosphoadenylylsulfate + ADP-energy + Pyrophosphate |
| 79 | 0.009300834767392185 | 1 | false | 1 | ATP-energy + Ca2+IN + H2O = ADP-energy + Orthophosphate |
| 80 | 0.0022206474374074636 | 1 | false | 1 | Ca2+IN + H2O + ITP-extern = IMP + Pyrophosphate |
| 81 | 0.008825953610068216 | 1 | false | 1 | H2O + UTP = Pyrophosphate + UMP |
| 82 | 0.008979317355852112 | 7 | false | 7 | ADP-energy + CO2 + IMP + NH3 + Phosphoenolpyruvate = (S)-Malate + ATP-energy + GMP |
| 83 | 0.001492299580795775 | 5 | false | 5 | no net reaction |
| 84 | 0.00848849181548772 | 2 | false | 2 | ATP-energy + NH3 + Uridine = ADP-energy + CMP + H2O |
| 85 | 0.021681261743009217 | 2 | false | 2 | GMP + H2O = Guanine + alpha-D-Ribose1-phosphate |
| 86 | 0.057702289633061454 | 2 | false | 2 | DNA-G + IQ-143= DNA-G_blocked + IQ-_used |
| 87 | 0.0012961275747516154 | 4 | false | 4 | AMP-energy + H2O + Pyrophosphate = 5-Phospho-alpha-D-ribose1-diphosphate + Hypoxanthine + NH3 |
| 88 | 0.010894109338160374 | 3 | false | 3 | ATP-energy + NH3 + Uridine = ADP-energy + CMP + H2O |
| 89 | 0.030690514747585662 | 2 | false | 2 | DNA-A + IQ-143= DNA-A_blocked + IQ-_used |
| 90 | 0.0074227893781351195 | 3 | false | 3 | ADP-energy + IQ-143+ Pyrophosphate + RNA-C + UDP = ATP-energy + CDP + IQ-_used + Orthophosphate + RNA-U_blocked |
| 91 | 0.010613528041014009 | 3 | false | 3 | 5-Phospho-alpha-D-ribose1-diphosphate + H2O = Pyrophosphate + alpha-D-Ribose1-phosphate |
| 92 | 0.8837780364757674 | 1 | false | 1 | ATP-energy + IQ-143+ Sulfate = Adenylylsulfate_blocked + IQ-_used + Pyrophosphate |
| 93 | 0.024348581021184867 | 1 | false | 1 | GTP + Pyruvate = GDP + Phosphoenolpyruvate |
| 94 | 0.007376710993599267 | 1 | false | 1 | Ca2+IN + H2O + UDP = Orthophosphate + UMP |
| 95 | 0.004201194469749314 | 3 | false | 3 | ATP-energy + NH3 + Uridine = ADP-energy + CMP + H2O |
| 96 | 0.0341601707739696 | 1 | false | 1 | Ca2+IN + GDP + H2O = GMP + Orthophosphate |
| 97 | 0.0019757778213018717 | 2 | false | 2 | ATP-energy + H2O = ADP-energy + Orthophosphate |
| 98 | 4.581880484155754E-5 | 1 | false | 1 | H2O + UMP = Orthophosphate + Uridine |
| 99 | 0.004142418877938869 | 2 | false | 2 | ATP-energy + Pyruvate = ADP-energy + Phosphoenolpyruvate |
| 100 | 6.64663831680401E-4 | 8 | false | 8 | 510-Methylenetetrahydrofolate + DNA-C + H2O = DNA-T + Dihydrofolate + NH3 |
| 101 | 3.174750994855424E-4 | 3 | false | 3 | ATP-energy + NH3 + Uridine = ADP-energy + CMP + H2O |
| 102 | 0.001804175549338094 | 3 | false | 3 | ATP-energy + H2O = AMP-energy + Pyrophosphate |
| 103 | 0.02591033431757106 | 1 | false | 1 | ATP-energy + NH3 + Xanthosine5-phosphate = AMP-energy + GMP + Pyrophosphate |
| 104 | 2.335524305914216E-4 | 2 | false | 2 | GTP + NH3 + Uridine = CMP + GDP + H2O |
| 105 | 0.006859798908416703 | 4 | false | 4 | 2 ATP-energy + HCO3- + L-Aspartate + L-Glutamine + Oxygen = 2 ADP-energy + H2O2 + L-Glutamate + Orotate + 2 Orthophosphate |
| 106 | 0.09430912891069909 | 1 | false | 1 | Ca2+IN + GTP + H2O = GDP + Orthophosphate |
| 107 | 0.01513046193649914 | 2 | false | 2 | NH3 + UTP + Uridine = CMP + H2O + UDP |
| 108 | 0.0031978007666132457 | 3 | false | 3 | 2 ATP-energy + H2O = 2 ADP-energy + Pyrophosphate |
| 109 | 0.9866322844197013 | 3 | false | 3 | ATP-energy + Ca2+IN + H2O = ADP-energy + Orthophosphate |
| 110 | 0.004823885645214965 | 2 | false | 2 | ATP-energy + Uridine = ADP-energy + UMP |
| 111 | 0.02008514117824356 | 2 | false | 2 | ATP-energy + Ca2+IN + H2O = ADP-energy + Orthophosphate |
| 112 | 0.0037166464650704167 | 4 | false | 4 | no net reaction |
| 113 | 0.027461291380987185 | 2 | false | 2 | NH3 + UTP = CDP + Orthophosphate |
| 114 | 1.238309503881485E-4 | 1 | false | 1 | ATP-energy + H2O + L-Glutamine + Xanthosine5-phosphate = AMP-energy + GMP + L-Glutamate + Pyrophosphate |
| 115 | 0.0069347528912339085 | 3 | false | 3 | ADP-energy + ADP-metabolism + IQ-143+ Pyrophosphate + RNA-C = ATP-energy + CDP + IQ-_used + Orthophosphate + RNA-A_blocked |
| 116 | 0.0014966550786249533 | 12 | false | 12 | 5-Phospho-alpha-D-ribose1-diphosphate + ATP-energy + DNA-A + NH3 + Uracil = ADP-energy + AMP-energy + DNA-C + Orthophosphate + Pyrophosphate |
| 117 | 0.00961678170064062 | 2 | false | 2 | 5-Phospho-alpha-D-ribose1-diphosphate + H2O + Hypoxanthine + Oxygen = H2O2 + Pyrophosphate + Xanthosine5-phosphate |
| 118 | 0.003486425047949271 | 1 | false | 1 | ATP-energy + IQ-143= ADP-energy + IQ-_deactivated |
| 119 | 0.004048704451746876 | 1 | false | 1 | GTP + Uridine = GDP + UMP |
| 120 | 7.601547877701398E-4 | 2 | false | 2 | H2O + IMP = Hypoxanthine + alpha-D-Ribose1-phosphate |
| 121 | 0.0023616325357191936 | 2 | false | 2 | CMP + 2 H2O = NH3 + Orthophosphate + Uridine |
| 122 | 0.010139460239020348 | 1 | false | 1 | ATP-energy + IQ-143= ADP-energy + IQ-_deactivated |
| 123 | 0.013870108901137601 | 2 | false | 2 | ATP-energy + Uridine = ADP-energy + UMP |
| 124 | 0.008015444330371158 | 1 | false | 1 | ATP-energy + Pyruvate = ADP-energy + Phosphoenolpyruvate |
| 125 | 0.0030630428480734606 | 3 | false | 3 | 2 ATP-energy + H2O = 2 ADP-energy + Pyrophosphate |
| 126 | 0.007861645349033441 | 2 | false | 2 | ATP-energy + H2O = AMP-energy + Pyrophosphate |
| 127 | 0.026861811545211167 | 3 | false | 3 | ATP-energy + H2O = ADP-energy + Orthophosphate |
| 128 | 0.015648974186699194 | 3 | false | 3 | ADP-energy + ADP-metabolism + Pyrophosphate + RNA-C = ATP-energy + CDP + Orthophosphate + RNA-A |
| 129 | 0.02750206970242519 | 1 | false | 1 | ATP-metabolism + H2O = ADP-metabolism + Orthophosphate |
| 130 | 0.006421668756456889 | 1 | false | 1 | ATP-energy + IQ-143= ADP-energy + IQ-_deactivated |
| 131 | 0.017854367612828104 | 3 | false | 3 | ADP-energy + IQ-143+ Pyrophosphate + RNA-C = ATP-energy + IQ-_used + Orthophosphate + RNA-C_blocked |
| 132 | 0.007359227270793189 | 2 | false | 2 | 2 H2O + P1P4-Bis(5-xanthosyl)tetraphosphate = Pyrophosphate + 2 Xanthosine5-phosphate |
| 133 | 0.0025019821493916794 | 2 | false | 2 | GTP + H2O = GMP + Pyrophosphate |
| 134 | 0.02467670775406272 | 1 | false | 1 | ATP-energy + IQ-143= ADP-energy + IQ-_deactivated |
| 135 | 0.005500357152584456 | 1 | false | 1 | ATP-energy + Uridine = ADP-energy + UMP |
| 136 | 0.006538273927002236 | 4 | false | 4 | DNA-A + H2O + Oxidizedthioredoxin + Pyrophosphate = ATP-energy + DNA-extern + Thioredoxin |
| 137 | 4.8528168517425474E-4 | 3 | false | 3 | ATP-energy + NH3 + Uridine = ADP-energy + CMP + H2O |
| 138 | 0.0058922524232456475 | 2 | false | 2 | H2O + Oxygen + Pyrophosphate + Xanthosine5-phosphate = 5-Phospho-alpha-D-ribose1-diphosphate + H2O2 + Urate |
| 139 | 0.00868422262778401 | 2 | false | 2 | H2O + L-Glutamine + UTP = CDP + L-Glutamate + Orthophosphate |
| 140 | 0.015653147128281475 | 2 | false | 2 | DNA-T + IQ-143= DNA-T_blocked + IQ-_used |
| 141 | 0.0289175789015248 | 1 | false | 1 | ATP-energy + IQ-143= ADP-energy + IQ-_deactivated |
| 142 | 0.017844171075269033 | 1 | false | 1 | Ca2+IN + H2O + UTP = Orthophosphate + UDP |
| 143 | 9.747119662268933E-4 | 7 | false | 7 | ADP-energy + DNA-C + 2 H2O + Oxidizedthioredoxin + Pyrophosphate = ATP-energy + DNA-extern + NH3 + Thioredoxin + UDP |
| 144 | 0.023538204503250504 | 7 | false | 7 | ADP-energy + CO2 + Phosphoenolpyruvate + Thioredoxin = (S)-Malate + ATP-energy + Oxidizedthioredoxin |
| 145 | 0.03866035115109223 | 1 | false | 1 | GTP + H2O = GMP + Pyrophosphate |
| 146 | 0.010487485921525264 | 1 | false | 1 | AMP-energy + H2O = IMP + NH3 |
| 147 | 0.0022429419981450094 | 3 | false | 3 | AMP-energy + ATP-energy + Pyrophosphate + alpha-D-Ribose1-phosphate = 5-Phospho-alpha-D-ribose1-diphosphate + ADP-energy + AMP-metabolism + Orthophosphate |
| 148 | 0.015311481162256335 | 3 | false | 3 | ADP-energy + GDP + Pyrophosphate + RNA-C = ATP-energy + CDP + Orthophosphate + RNA-G |
| 149 | 0.012361030082629898 | 1 | false | 1 | UTP + Uridine = UDP + UMP |
| 150 | 0.0038361570176190796 | 3 | false | 3 | 2 ATP-energy + H2O = 2 ADP-energy + Pyrophosphate |
| 151 | 0.024077724104495934 | 2 | false | 2 | DNA-C + IQ-143= DNA-C_blocked + IQ-_used |
| 152 | 0.004517643364860158 | 11 | false | 11 | DNA-C + Guanine + H2O = DNA-G + NH3 + Uracil |
| 153 | 0.004049895406326054 | 1 | false | 1 | Allantoate + H2O = Allantoine |
| 154 | 0.0011179498930405929 | 32 | false | 19 | 5 ADP-energy + CO2 + H2O + Orthophosphate + 4 Phosphoenolpyruvate + Pyruvate + Ubiquinone = 3 (S)-Malate + 5 ATP-energy + Fumarate + Ubiquinol |
| 155 | 0.004243888613050029 | 1 | false | 1 | Ca2+IN + H2O + UDP = Orthophosphate + UMP |

^1^ This data shows the elementary mode Analysis for *H. sapiens* without IQ-143:

Table S14: Extreme Modes of *H. sapiens* with 0.16µM IQ-143^1^:

| # | Activity | Flux sum | Reversible? | Pathlength | Net reaction |
| --- | --- | --- | --- | --- | --- |
| 1 | 0.005130030045226164 | 1 | true | 1 | H2O + P1P4-Bis(5-adenosyl)tetraphosphate = AMP-energy + ATP-energy |
| 2 | 0.4397620271448569 | 2 | true | 2 | ADP-energy + GTP = ATP-energy + GDP |
| 3 | 0.09480536073977541 | 1 | true | 1 | Orthophosphate + Uridine = Uracil + alpha-D-Ribose1-phosphate |
| 4 | 0.03823934247721361 | 1 | true | 1 | ATP-energy + UMP = ADP-energy + UDP |
| 5 | 0.009104482032904815 | 1 | true | 1 | ATP-energy + GMP = ADP-energy + GDP |
| 6 | 0.007704237115931267 | 3 | true | 3 | ATP-energy + CDP + RNA-U = ADP-energy + RNA-C + UTP |
| 7 | 0.0017270300754455015 | 3 | true | 3 | ATP-energy + CDP + RNA-G = ADP-energy + GTP + RNA-C |
| 8 | 0.05725996742131523 | 1 | true | 1 | ATP-energy + UMP = ADP-energy + UDP |
| 9 | 0.024214500034897335 | 1 | true | 1 | ATP-metabolism = ATP-energy |
| 10 | 0.009442734667085562 | 1 | true | 1 | Orotidine5-phosphate = CO2 + UMP |
| 11 | 0.01202298234818866 | 2 | true | 2 | ATP-energy + H2O = ADP-energy + Orthophosphate |
| 12 | 0.011202803201679434 | 1 | true | 1 | AMP-metabolism = AMP-energy |
| 13 | 0.9979458833338856 | 4 | true | 4 | ADP-energy + GTP = ATP-energy + GDP |
| 14 | 0.9941042589766935 | 4 | true | 4 | 2 ATP-energy + HCO3- + Pyruvate = 2 ADP-energy + CO2 + Orthophosphate + Phosphoenolpyruvate |
| 15 | 0.011028459588632078 | 2 | true | 2 | no net reaction |
| 16 | 0.020505076858065685 | 1 | true | 1 | Pyrophosphate + UMP = 5-Phospho-alpha-D-ribose1-diphosphate + Uracil |
| 17 | 0.020231747454989013 | 1 | true | 1 | ATP-energy + CMP = ADP-energy + CDP |
| 18 | 0.02088527667504403 | 1 | true | 1 | ATP-energy + UDP = ADP-energy + UTP |
| 19 | 0.004049843361527694 | 1 | true | 1 | H2O + P1P3-Bis(5-adenosyl)triphosphate = ADP-energy + AMP-energy |
| 20 | 0.006013692692126038 | 2 | true | 2 | no net reaction |
| 21 | 0.007596162022488362 | 2 | true | 2 | no net reaction |
| 22 | 0.004653191538830148 | 3 | true | 3 | no net reaction |
| 23 | 0.973957437873535 | 3 | true | 3 | no net reaction |
| 24 | 0.015164298309223834 | 1 | true | 1 | GMP + Pyrophosphate = 5-Phospho-alpha-D-ribose1-diphosphate + Guanine |
| 25 | 7.342594063648455E-4 | 3 | true | 3 | ATP-energy + CDP + RNA-A = ADP-energy + ATP-metabolism + RNA-C |
| 26 | 0.007375314266941269 | 1 | true | 1 | (S)-Malate = Fumarate + H2O |
| 27 | 0.008856758026085099 | 1 | true | 1 | H2O + P1P4-Bis(5-guanosyl)tetraphosphate = GMP + GTP |
| 28 | 0.044765835448331015 | 1 | true | 1 | ATP-energy + GDP = ADP-energy + GTP |
| 29 | 0.007429803106260624 | 1 | true | 1 | AMP-metabolism + ATP-energy = 2 ADP-metabolism |
| 30 | 0.04581608968990736 | 1 | true | 1 | Orotidine5-phosphate + Pyrophosphate = 5-Phospho-alpha-D-ribose1-diphosphate + Orotate |
| 31 | 0.009072609077389049 | 2 | true | 2 | GTP + IMP + L-Aspartate = AMP-energy + Fumarate + GDP + Orthophosphate |
| 32 | 0.0018817352810703847 | 2 | true | 2 | no net reaction |
| 33 | 0.001122515017348391 | 2 | true | 2 | no net reaction |
| 34 | 0.06639499216836431 | 1 | true | 1 | ADP-metabolism + ATP-energy = ADP-energy + ATP-metabolism |
| 35 | 0.03186315028386555 | 5 | true | 5 | (S)-Malate + ATP-energy + Pyruvate = (S)-Lactate + ADP-energy + CO2 + Phosphoenolpyruvate |
| 36 | 0.002501413320115886 | 1 | true | 1 | ADP-metabolism = AMP-energy |
| 37 | 0.004531527096812593 | 1 | true | 1 | AMP-energy + UTP = ADP-energy + UDP |
| 38 | 0.024179372922236708 | 1 | true | 1 | H2O + P1P4-Bis(5-uridyl)tetraphosphate = UMP + UTP |
| 39 | 0.0026795687764290532 | 5 | true | 5 | (S)-Malate + ATP-energy + Xanthosine5-phosphate = ADP-energy + CO2 + H2O + IMP + Phosphoenolpyruvate |
| 40 | 0.005717595903887207 | 4 | true | 4 | no net reaction |
| 41 | 0.028842907966356357 | 5 | true | 5 | no net reaction |
| 42 | 0.0011252802077290625 | 4 | true | 4 | 5-Amino-4-imidazolecarboxyamide + 5-Phospho-alpha-D-ribose1-diphosphate + ADP-metabolism + Fumarate + Orthophosphate = ATP-metabolism + Aminoimidazoleribotide + CO2 + L-Aspartate + Pyrophosphate |
| 43 | 0.005496024523628518 | 1 | true | 1 | IMP + Pyrophosphate = 5-Phospho-alpha-D-ribose1-diphosphate + Hypoxanthine |
| 44 | 3.5454087822606173E-4 | 3 | false | 3 | ADP-energy + Pyrophosphate = ATP-energy + Orthophosphate |
| 45 | 0.038917876242217875 | 1 | false | 1 | ADP-energy + Ca2+IN + H2O = AMP-energy + Orthophosphate |
| 46 | 0.0013525861946372952 | 4 | false | 4 | AMP-energy + H2O + Pyrophosphate = 5-Phospho-alpha-D-ribose1-diphosphate + Hypoxanthine + NH3 |
| 47 | 0.002492485469045347 | 2 | false | 2 | ATP-energy + H2O = ADP-energy + Orthophosphate |
| 48 | 0.002626741631829521 | 3 | false | 3 | ADP-energy + IQ-143+ GDP + Pyrophosphate + RNA-C = ATP-energy + CDP + IQ-_used + Orthophosphate + RNA-G_blocked |
| 49 | 0.004093079518189935 | 4 | false | 4 | ATP-energy + H2O = ADP-energy + Orthophosphate |
| 50 | 0.0013834190967744187 | 2 | false | 2 | ATP-energy + Uridine = ADP-energy + UMP |
| 51 | 0.06249878824378918 | 1 | false | 1 | ATP-energy + IQ-143= ADP-energy + IQ-_deactivated |
| 52 | 0.02767266096177401 | 1 | false | 1 | Ca2+IN + GTP-extern + H2O = GMP + Pyrophosphate |
| 53 | 0.9217398821749008 | 3 | false | 3 | ATP-energy + Uridine = ADP-energy + UMP |
| 54 | 0.030031470687912343 | 2 | false | 2 | ATP-energy + H2O = ADP-energy + Orthophosphate |
| 55 | 0.06378578683142644 | 1 | false | 1 | H2O + P1P4-Bis(5-adenosyl)tetraphosphate = 2 ADP-energy |
| 56 | 0.03379212500206907 | 3 | false | 3 | ATP-energy + NH3 + Uridine = ADP-energy + CMP + H2O |
| 57 | 0.003730768499201065 | 2 | false | 2 | ATP-energy + Uridine = ADP-energy + UMP |
| 58 | 0.0017552338135035095 | 4 | false | 4 | ATP-energy + NH3 + Uridine = ADP-energy + CMP + H2O |
| 59 | 0.014714368899165442 | 4 | false | 4 | ADP-energy + DNA-C + H2O + Oxidizedthioredoxin + Pyrophosphate = ATP-energy + CDP + DNA-extern + Thioredoxin |
| 60 | 0.005362707333533834 | 2 | false | 2 | GTP + H2O = GMP + Pyrophosphate |
| 61 | 0.02427660710520141 | 2 | false | 2 | ATP-energy + Uridine = ADP-energy + UMP |
| 62 | 0.009934934521844463 | 2 | false | 2 | ATP-energy + H2O = ADP-energy + Orthophosphate |
| 63 | 0.008655018738876752 | 1 | false | 1 | ATP-metabolism + Ca2+IN + H2O = AMP-energy + Orthophosphate |
| 64 | 0.05689430909157689 | 1 | false | 1 | CDP + Ca2+IN + H2O = CMP + Orthophosphate |
| 65 | 0.017796655470858735 | 2 | false | 2 | ATP-energy + Pyruvate = ADP-energy + Phosphoenolpyruvate |
| 66 | 0.0014726423314010972 | 2 | false | 2 | ADP-ribose + ATP-energy + H2O = 5-Phospho-alpha-D-ribose1-diphosphate + 2 AMP-energy |
| 67 | 0.0042776150948071745 | 2 | false | 2 | 5-Phospho-alpha-D-ribose1-diphosphate + Guanine + H2O = NH3 + Pyrophosphate + Xanthosine5-phosphate |
| 68 | 0.028939063589240477 | 3 | false | 3 | ADP-energy + Pyrophosphate + RNA-C + UDP = ATP-energy + CDP + Orthophosphate + RNA-U |
| 69 | 0.0019344000178397325 | 11 | false | 11 | ATP-energy + DNA-G + NH3 + Uracil = ADP-energy + DNA-C + Guanine + Orthophosphate |
| 70 | 0.004886284569103672 | 6 | false | 6 | ADP-energy + CO2 + H2O + Phosphoenolpyruvate + Pyrophosphate + Xanthosine5-phosphate = (S)-Malate + 5-Phospho-alpha-D-ribose1-diphosphate + ATP-energy + Urate |
| 71 | 0.013029597677572946 | 1 | false | 1 | Ca2+IN + GDP + H2O = GMP + Orthophosphate |
| 72 | 0.011362712812997389 | 1 | false | 1 | ATP-metabolism + Ca2+IN + H2O = ADP-energy + Orthophosphate |
| 73 | 9.490223452437441E-4 | 3 | false | 3 | 5-Phospho-alpha-D-ribose1-diphosphate + H2O = Pyrophosphate + alpha-D-Ribose1-phosphate |
| 74 | 0.008945411265786518 | 2 | false | 2 | ATP-energy + Ca2+IN + H2O = ADP-energy + Orthophosphate |
| 75 | 0.037504588669099004 | 6 | false | 6 | 5-Phospho-alpha-D-ribose1-diphosphate + ADP-energy + CO2 + H+ + H2O + Hypoxanthine + Phosphoenolpyruvate = (S)-Malate + ATP-energy + Pyrophosphate + Xanthosine5-phosphate |
| 76 | 0.021714933706790007 | 2 | false | 2 | ATP-energy + Ca2+IN + H2O = ADP-energy + Orthophosphate |
| 77 | 0.00473388172511402 | 4 | false | 4 | ADP-energy + DNA-G + H2O + Oxidizedthioredoxin + Pyrophosphate = ATP-energy + DNA-extern + GDP + Thioredoxin |
| 78 | 0.0049789841623851805 | 2 | false | 2 | 2 ATP-energy + Sulfate = 3-Phosphoadenylylsulfate + ADP-energy + Pyrophosphate |
| 79 | 0.02462816689965397 | 1 | false | 1 | ATP-energy + Ca2+IN + H2O = ADP-energy + Orthophosphate |
| 80 | 0.05674045026156782 | 1 | false | 1 | Ca2+IN + H2O + ITP-extern = IMP + Pyrophosphate |
| 81 | 0.012680697978049826 | 1 | false | 1 | H2O + UTP = Pyrophosphate + UMP |
| 82 | 0.007168636006845586 | 7 | false | 7 | ADP-energy + CO2 + IMP + NH3 + Phosphoenolpyruvate = (S)-Malate + ATP-energy + GMP |
| 83 | 0.010391719615760908 | 5 | false | 5 | no net reaction |
| 84 | 0.005244773454489482 | 2 | false | 2 | ATP-energy + NH3 + Uridine = ADP-energy + CMP + H2O |
| 85 | 0.03392668547022826 | 2 | false | 2 | GMP + H2O = Guanine + alpha-D-Ribose1-phosphate |
| 86 | 0.001402994837579885 | 2 | false | 2 | DNA-G + IQ-143= DNA-G_blocked + IQ-_used |
| 87 | 0.0027033208390069285 | 4 | false | 4 | AMP-energy + H2O + Pyrophosphate = 5-Phospho-alpha-D-ribose1-diphosphate + Hypoxanthine + NH3 |
| 88 | 0.011498488414621533 | 3 | false | 3 | ATP-energy + NH3 + Uridine = ADP-energy + CMP + H2O |
| 89 | 0.0061472460822511454 | 2 | false | 2 | DNA-A + IQ-143= DNA-A_blocked + IQ-_used |
| 90 | 0.0018108729538574009 | 3 | false | 3 | ADP-energy + IQ-143+ Pyrophosphate + RNA-C + UDP = ATP-energy + CDP + IQ-_used + Orthophosphate + RNA-U_blocked |
| 91 | 0.011049185312248078 | 3 | false | 3 | 5-Phospho-alpha-D-ribose1-diphosphate + H2O = Pyrophosphate + alpha-D-Ribose1-phosphate |
| 92 | 0.028842651739239833 | 1 | false | 1 | ATP-energy + IQ-143+ Sulfate = Adenylylsulfate_blocked + IQ-_used + Pyrophosphate |
| 93 | 0.05461213055700753 | 1 | false | 1 | GTP + Pyruvate = GDP + Phosphoenolpyruvate |
| 94 | 0.014964491850709916 | 1 | false | 1 | Ca2+IN + H2O + UDP = Orthophosphate + UMP |
| 95 | 0.007781740827399131 | 3 | false | 3 | ATP-energy + NH3 + Uridine = ADP-energy + CMP + H2O |
| 96 | 0.06305805710461365 | 1 | false | 1 | Ca2+IN + GDP + H2O = GMP + Orthophosphate |
| 97 | 0.0173973276128907 | 2 | false | 2 | ATP-energy + H2O = ADP-energy + Orthophosphate |
| 98 | 0.04225034421881002 | 1 | false | 1 | H2O + UMP = Orthophosphate + Uridine |
| 99 | 0.021249692031397704 | 2 | false | 2 | ATP-energy + Pyruvate = ADP-energy + Phosphoenolpyruvate |
| 100 | 0.004424086757605639 | 8 | false | 8 | 510-Methylenetetrahydrofolate + DNA-C + H2O = DNA-T + Dihydrofolate + NH3 |
| 101 | 0.005398561772016364 | 3 | false | 3 | ATP-energy + NH3 + Uridine = ADP-energy + CMP + H2O |
| 102 | 3.3045236095607944E-4 | 3 | false | 3 | ATP-energy + H2O = AMP-energy + Pyrophosphate |
| 103 | 0.013104590439268482 | 1 | false | 1 | ATP-energy + NH3 + Xanthosine5-phosphate = AMP-energy + GMP + Pyrophosphate |
| 104 | 0.0015242489203124432 | 2 | false | 2 | GTP + NH3 + Uridine = CMP + GDP + H2O |
| 105 | 6.166920175361135E-4 | 4 | false | 4 | 2 ATP-energy + HCO3- + L-Aspartate + L-Glutamine + Oxygen = 2 ADP-energy + H2O2 + L-Glutamate + Orotate + 2 Orthophosphate |
| 106 | 0.008111785491578982 | 1 | false | 1 | Ca2+IN + GTP + H2O = GDP + Orthophosphate |
| 107 | 0.004761078016804143 | 2 | false | 2 | NH3 + UTP + Uridine = CMP + H2O + UDP |
| 108 | 0.00726859053151796 | 3 | false | 3 | 2 ATP-energy + H2O = 2 ADP-energy + Pyrophosphate |
| 109 | 0.978557363640139 | 3 | false | 3 | ATP-energy + Ca2+IN + H2O = ADP-energy + Orthophosphate |
| 110 | 0.009356081770501179 | 2 | false | 2 | ATP-energy + Uridine = ADP-energy + UMP |
| 111 | 0.007757346401982335 | 2 | false | 2 | ATP-energy + Ca2+IN + H2O = ADP-energy + Orthophosphate |
| 112 | 0.007899099267346443 | 4 | false | 4 | no net reaction |
| 113 | 0.003637222532724582 | 2 | false | 2 | NH3 + UTP = CDP + Orthophosphate |
| 114 | 0.024721054946452714 | 1 | false | 1 | ATP-energy + H2O + L-Glutamine + Xanthosine5-phosphate = AMP-energy + GMP + L-Glutamate + Pyrophosphate |
| 115 | 0.002172243679348207 | 3 | false | 3 | ADP-energy + ADP-metabolism + IQ-143+ Pyrophosphate + RNA-C = ATP-energy + CDP + IQ-_used + Orthophosphate + RNA-A_blocked |
| 116 | 3.426474497347787E-5 | 12 | false | 12 | 5-Phospho-alpha-D-ribose1-diphosphate + ATP-energy + DNA-A + NH3 + Uracil = ADP-energy + AMP-energy + DNA-C + Orthophosphate + Pyrophosphate |
| 117 | 0.018897696087057603 | 2 | false | 2 | 5-Phospho-alpha-D-ribose1-diphosphate + H2O + Hypoxanthine + Oxygen = H2O2 + Pyrophosphate + Xanthosine5-phosphate |
| 118 | 0.008940149045068346 | 1 | false | 1 | ATP-energy + IQ-143= ADP-energy + IQ-_deactivated |
| 119 | 0.00829517643691624 | 1 | false | 1 | GTP + Uridine = GDP + UMP |
| 120 | 1.198011158797252E-4 | 2 | false | 2 | H2O + IMP = Hypoxanthine + alpha-D-Ribose1-phosphate |
| 121 | 0.022565897110051525 | 2 | false | 2 | CMP + 2 H2O = NH3 + Orthophosphate + Uridine |
| 122 | 0.04362319987102781 | 1 | false | 1 | ATP-energy + IQ-143= ADP-energy + IQ-_deactivated |
| 123 | 7.527538197071859E-5 | 2 | false | 2 | ATP-energy + Uridine = ADP-energy + UMP |
| 124 | 0.0054598110854725546 | 1 | false | 1 | ATP-energy + Pyruvate = ADP-energy + Phosphoenolpyruvate |
| 125 | 0.0031234091917802242 | 3 | false | 3 | 2 ATP-energy + H2O = 2 ADP-energy + Pyrophosphate |
| 126 | 0.008061512557000716 | 2 | false | 2 | ATP-energy + H2O = AMP-energy + Pyrophosphate |
| 127 | 0.0010510169904632827 | 3 | false | 3 | ATP-energy + H2O = ADP-energy + Orthophosphate |
| 128 | 7.294308598648769E-4 | 3 | false | 3 | ADP-energy + ADP-metabolism + Pyrophosphate + RNA-C = ATP-energy + CDP + Orthophosphate + RNA-A |
| 129 | 0.025992562132787334 | 1 | false | 1 | ATP-metabolism + H2O = ADP-metabolism + Orthophosphate |
| 130 | 0.015591813645899566 | 1 | false | 1 | ATP-energy + IQ-143= ADP-energy + IQ-_deactivated |
| 131 | 0.019361033089730917 | 3 | false | 3 | ADP-energy + IQ-143+ Pyrophosphate + RNA-C = ATP-energy + IQ-_used + Orthophosphate + RNA-C_blocked |
| 132 | 0.004785085285212376 | 2 | false | 2 | 2 H2O + P1P4-Bis(5-xanthosyl)tetraphosphate = Pyrophosphate + 2 Xanthosine5-phosphate |
| 133 | 0.009332045603517969 | 2 | false | 2 | GTP + H2O = GMP + Pyrophosphate |
| 134 | 0.014022959237212751 | 1 | false | 1 | ATP-energy + IQ-143= ADP-energy + IQ-_deactivated |
| 135 | 0.015626722755678557 | 1 | false | 1 | ATP-energy + Uridine = ADP-energy + UMP |
| 136 | 0.006122072625027286 | 4 | false | 4 | DNA-A + H2O + Oxidizedthioredoxin + Pyrophosphate = ATP-energy + DNA-extern + Thioredoxin |
| 137 | 1.5673110828329229E-4 | 3 | false | 3 | ATP-energy + NH3 + Uridine = ADP-energy + CMP + H2O |
| 138 | 0.0034552749225255086 | 2 | false | 2 | H2O + Oxygen + Pyrophosphate + Xanthosine5-phosphate = 5-Phospho-alpha-D-ribose1-diphosphate + H2O2 + Urate |
| 139 | 0.002503619054793238 | 2 | false | 2 | H2O + L-Glutamine + UTP = CDP + L-Glutamate + Orthophosphate |
| 140 | 0.005954231138678656 | 2 | false | 2 | DNA-T + IQ-143= DNA-T_blocked + IQ-_used |
| 141 | 0.01594636277704542 | 1 | false | 1 | ATP-energy + IQ-143= ADP-energy + IQ-_deactivated |
| 142 | 0.042874775592222525 | 1 | false | 1 | Ca2+IN + H2O + UTP = Orthophosphate + UDP |
| 143 | 0.0025932956925613926 | 7 | false | 7 | ADP-energy + DNA-C + 2 H2O + Oxidizedthioredoxin + Pyrophosphate = ATP-energy + DNA-extern + NH3 + Thioredoxin + UDP |
| 144 | 0.002449448426728229 | 7 | false | 7 | ADP-energy + CO2 + Phosphoenolpyruvate + Thioredoxin = (S)-Malate + ATP-energy + Oxidizedthioredoxin |
| 145 | 0.008968343979364857 | 1 | false | 1 | GTP + H2O = GMP + Pyrophosphate |
| 146 | 0.03491931732673392 | 1 | false | 1 | AMP-energy + H2O = IMP + NH3 |
| 147 | 0.004039751031648775 | 3 | false | 3 | AMP-energy + ATP-energy + Pyrophosphate + alpha-D-Ribose1-phosphate = 5-Phospho-alpha-D-ribose1-diphosphate + ADP-energy + AMP-metabolism + Orthophosphate |
| 148 | 0.010084535436451492 | 3 | false | 3 | ADP-energy + GDP + Pyrophosphate + RNA-C = ATP-energy + CDP + Orthophosphate + RNA-G |
| 149 | 0.005047005030052842 | 1 | false | 1 | UTP + Uridine = UDP + UMP |
| 150 | 0.010647652395587737 | 3 | false | 3 | 2 ATP-energy + H2O = 2 ADP-energy + Pyrophosphate |
| 151 | 0.005301580724417554 | 2 | false | 2 | DNA-C + IQ-143= DNA-C_blocked + IQ-_used |
| 152 | 0.00838356805192797 | 11 | false | 11 | DNA-C + Guanine + H2O = DNA-G + NH3 + Uracil |
| 153 | 0.00599529324361292 | 1 | false | 1 | Allantoate + H2O = Allantoine |
| 154 | 0.004394616104792082 | 32 | false | 19 | 5 ADP-energy + CO2 + H2O + Orthophosphate + 4 Phosphoenolpyruvate + Pyruvate + Ubiquinone = 3 (S)-Malate + 5 ATP-energy + Fumarate + Ubiquinol |
| 155 | 0.06899209623572422 | 1 | false | 1 | Ca2+IN + H2O + UDP = Orthophosphate + UMP |

^1^ This data shows the elementary mode Analysis for *H. sapiens* with 0.16µM IQ-143:

Table S15: Extreme Modes of *H. sapiens* with 1.25µM IQ-143^1^:

| # | Activity | Flux sum | Reversible? | Pathlength | Net reaction |
| --- | --- | --- | --- | --- | --- |
| 1 | 0.04786344329223191 | 1 | true | 1 | H2O + P1P4-Bis(5-adenosyl)tetraphosphate = AMP-energy + ATP-energy |
| 2 | 9.969914267465851E-4 | 2 | true | 2 | ADP-energy + GTP = ATP-energy + GDP |
| 3 | 0.043535723909962964 | 1 | true | 1 | Orthophosphate + Uridine = Uracil + alpha-D-Ribose1-phosphate |
| 4 | 0.0024175638614146733 | 1 | true | 1 | ATP-energy + UMP = ADP-energy + UDP |
| 5 | 0.015989171210082476 | 1 | true | 1 | ATP-energy + GMP = ADP-energy + GDP |
| 6 | 0.02610634288231406 | 3 | true | 3 | ATP-energy + CDP + RNA-U = ADP-energy + RNA-C + UTP |
| 7 | 0.0024562865309492032 | 3 | true | 3 | ATP-energy + CDP + RNA-G = ADP-energy + GTP + RNA-C |
| 8 | 5.19077773227683E-4 | 1 | true | 1 | ATP-energy + UMP = ADP-energy + UDP |
| 9 | 0.017423153536418656 | 1 | true | 1 | ATP-metabolism = ATP-energy |
| 10 | 0.04700909087115568 | 1 | true | 1 | Orotidine5-phosphate = CO2 + UMP |
| 11 | 0.022066422914005845 | 2 | true | 2 | ATP-energy + H2O = ADP-energy + Orthophosphate |
| 12 | 0.01030019410617422 | 1 | true | 1 | AMP-metabolism = AMP-energy |
| 13 | 0.004261111240344895 | 4 | true | 4 | ADP-energy + GTP = ATP-energy + GDP |
| 14 | 0.013080093351799937 | 4 | true | 4 | 2 ATP-energy + HCO3- + Pyruvate = 2 ADP-energy + CO2 + Orthophosphate + Phosphoenolpyruvate |
| 15 | 0.0012275664668326414 | 2 | true | 2 | no net reaction |
| 16 | 0.00433006585860185 | 1 | true | 1 | Pyrophosphate + UMP = 5-Phospho-alpha-D-ribose1-diphosphate + Uracil |
| 17 | 0.019781052892711704 | 1 | true | 1 | ATP-energy + CMP = ADP-energy + CDP |
| 18 | 0.0010147643752992375 | 1 | true | 1 | ATP-energy + UDP = ADP-energy + UTP |
| 19 | 0.006170733778273374 | 1 | true | 1 | H2O + P1P3-Bis(5-adenosyl)triphosphate = ADP-energy + AMP-energy |
| 20 | 0.016076798397335335 | 2 | true | 2 | no net reaction |
| 21 | 0.011940090048190632 | 2 | true | 2 | no net reaction |
| 22 | 0.025841353292343383 | 3 | true | 3 | no net reaction |
| 23 | 0.028192008364815835 | 3 | true | 3 | no net reaction |
| 24 | 0.005615818134375838 | 1 | true | 1 | GMP + Pyrophosphate = 5-Phospho-alpha-D-ribose1-diphosphate + Guanine |
| 25 | 0.007894959793193546 | 3 | true | 3 | ATP-energy + CDP + RNA-A = ADP-energy + ATP-metabolism + RNA-C |
| 26 | 0.041824191955041945 | 1 | true | 1 | (S)-Malate = Fumarate + H2O |
| 27 | 0.04015022028649451 | 1 | true | 1 | H2O + P1P4-Bis(5-guanosyl)tetraphosphate = GMP + GTP |
| 28 | 0.12142369551244281 | 1 | true | 1 | ATP-energy + GDP = ADP-energy + GTP |
| 29 | 0.021965147209823566 | 1 | true | 1 | AMP-metabolism + ATP-energy = 2 ADP-metabolism |
| 30 | 0.030048932370557502 | 1 | true | 1 | Orotidine5-phosphate + Pyrophosphate = 5-Phospho-alpha-D-ribose1-diphosphate + Orotate |
| 31 | 0.002086001036413987 | 2 | true | 2 | GTP + IMP + L-Aspartate = AMP-energy + Fumarate + GDP + Orthophosphate |
| 32 | 0.006743979293639235 | 2 | true | 2 | no net reaction |
| 33 | 0.012944044905924934 | 2 | true | 2 | no net reaction |
| 34 | 0.00441884231341827 | 1 | true | 1 | ADP-metabolism + ATP-energy = ADP-energy + ATP-metabolism |
| 35 | 0.003571153435644736 | 5 | true | 5 | (S)-Malate + ATP-energy + Pyruvate = (S)-Lactate + ADP-energy + CO2 + Phosphoenolpyruvate |
| 36 | 0.004534729677700966 | 1 | true | 1 | ADP-metabolism = AMP-energy |
| 37 | 0.032044253587553806 | 1 | true | 1 | AMP-energy + UTP = ADP-energy + UDP |
| 38 | 7.099999894971676E-4 | 1 | true | 1 | H2O + P1P4-Bis(5-uridyl)tetraphosphate = UMP + UTP |
| 39 | 0.010679959181318699 | 5 | true | 5 | (S)-Malate + ATP-energy + Xanthosine5-phosphate = ADP-energy + CO2 + H2O + IMP + Phosphoenolpyruvate |
| 40 | 0.007486618692184921 | 4 | true | 4 | no net reaction |
| 41 | 0.013612737316568868 | 5 | true | 5 | no net reaction |
| 42 | 0.0016902796065854098 | 4 | true | 4 | 5-Amino-4-imidazolecarboxyamide + 5-Phospho-alpha-D-ribose1-diphosphate + ADP-metabolism + Fumarate + Orthophosphate = ATP-metabolism + Aminoimidazoleribotide + CO2 + L-Aspartate + Pyrophosphate |
| 43 | 0.03245607435050746 | 1 | true | 1 | IMP + Pyrophosphate = 5-Phospho-alpha-D-ribose1-diphosphate + Hypoxanthine |
| 44 | 0.03648286865370309 | 3 | false | 3 | ADP-energy + Pyrophosphate = ATP-energy + Orthophosphate |
| 45 | 0.06904247945993924 | 1 | false | 1 | ADP-energy + Ca2+IN + H2O = AMP-energy + Orthophosphate |
| 46 | 0.003848199268884356 | 4 | false | 4 | AMP-energy + H2O + Pyrophosphate = 5-Phospho-alpha-D-ribose1-diphosphate + Hypoxanthine + NH3 |
| 47 | 0.005319774297396718 | 2 | false | 2 | ATP-energy + H2O = ADP-energy + Orthophosphate |
| 48 | 0.02341366278916257 | 3 | false | 3 | ADP-energy + IQ-143+ GDP + Pyrophosphate + RNA-C = ATP-energy + CDP + IQ-_used + Orthophosphate + RNA-G_blocked |
| 49 | 4.675662664771485E-4 | 4 | false | 4 | ATP-energy + H2O = ADP-energy + Orthophosphate |
| 50 | 0.0053543977035213075 | 2 | false | 2 | ATP-energy + Uridine = ADP-energy + UMP |
| 51 | 0.010394427608318435 | 1 | false | 1 | ATP-energy + IQ-143= ADP-energy + IQ-_deactivated |
| 52 | 0.03227190121903123 | 1 | false | 1 | Ca2+IN + GTP-extern + H2O = GMP + Pyrophosphate |
| 53 | 0.0015538189262196411 | 3 | false | 3 | ATP-energy + Uridine = ADP-energy + UMP |
| 54 | 0.0012552736251809637 | 2 | false | 2 | ATP-energy + H2O = ADP-energy + Orthophosphate |
| 55 | 0.02581182905210888 | 1 | false | 1 | H2O + P1P4-Bis(5-adenosyl)tetraphosphate = 2 ADP-energy |
| 56 | 0.005194653803581528 | 3 | false | 3 | ATP-energy + NH3 + Uridine = ADP-energy + CMP + H2O |
| 57 | 0.005633159716414093 | 2 | false | 2 | ATP-energy + Uridine = ADP-energy + UMP |
| 58 | 0.014057220558407302 | 4 | false | 4 | ATP-energy + NH3 + Uridine = ADP-energy + CMP + H2O |
| 59 | 0.006242782616069031 | 4 | false | 4 | ADP-energy + DNA-C + H2O + Oxidizedthioredoxin + Pyrophosphate = ATP-energy + CDP + DNA-extern + Thioredoxin |
| 60 | 0.004282826338055878 | 2 | false | 2 | GTP + H2O = GMP + Pyrophosphate |
| 61 | 0.001134341575070752 | 2 | false | 2 | ATP-energy + Uridine = ADP-energy + UMP |
| 62 | 0.0010239360615290627 | 2 | false | 2 | ATP-energy + H2O = ADP-energy + Orthophosphate |
| 63 | 0.027356029648537894 | 1 | false | 1 | ATP-metabolism + Ca2+IN + H2O = AMP-energy + Orthophosphate |
| 64 | 0.0030045698055946035 | 1 | false | 1 | CDP + Ca2+IN + H2O = CMP + Orthophosphate |
| 65 | 0.016130726483697178 | 2 | false | 2 | ATP-energy + Pyruvate = ADP-energy + Phosphoenolpyruvate |
| 66 | 0.03019053483656997 | 2 | false | 2 | ADP-ribose + ATP-energy + H2O = 5-Phospho-alpha-D-ribose1-diphosphate + 2 AMP-energy |
| 67 | 0.0053798800333575425 | 2 | false | 2 | 5-Phospho-alpha-D-ribose1-diphosphate + Guanine + H2O = NH3 + Pyrophosphate + Xanthosine5-phosphate |
| 68 | 0.14524612475598087 | 3 | false | 3 | ADP-energy + Pyrophosphate + RNA-C + UDP = ATP-energy + CDP + Orthophosphate + RNA-U |
| 69 | 0.00922704665219487 | 11 | false | 11 | ATP-energy + DNA-G + NH3 + Uracil = ADP-energy + DNA-C + Guanine + Orthophosphate |
| 70 | 0.001072276755925583 | 6 | false | 6 | ADP-energy + CO2 + H2O + Phosphoenolpyruvate + Pyrophosphate + Xanthosine5-phosphate = (S)-Malate + 5-Phospho-alpha-D-ribose1-diphosphate + ATP-energy + Urate |
| 71 | 0.0031409560779063206 | 1 | false | 1 | Ca2+IN + GDP + H2O = GMP + Orthophosphate |
| 72 | 0.008811456227530878 | 1 | false | 1 | ATP-metabolism + Ca2+IN + H2O = ADP-energy + Orthophosphate |
| 73 | 0.007136260273736927 | 3 | false | 3 | 5-Phospho-alpha-D-ribose1-diphosphate + H2O = Pyrophosphate + alpha-D-Ribose1-phosphate |
| 74 | 0.0013534572263430578 | 2 | false | 2 | ATP-energy + Ca2+IN + H2O = ADP-energy + Orthophosphate |
| 75 | 0.008475650468189877 | 6 | false | 6 | 5-Phospho-alpha-D-ribose1-diphosphate + ADP-energy + CO2 + H+ + H2O + Hypoxanthine + Phosphoenolpyruvate = (S)-Malate + ATP-energy + Pyrophosphate + Xanthosine5-phosphate |
| 76 | 0.02931985124428904 | 2 | false | 2 | ATP-energy + Ca2+IN + H2O = ADP-energy + Orthophosphate |
| 77 | 0.012858209684887156 | 4 | false | 4 | ADP-energy + DNA-G + H2O + Oxidizedthioredoxin + Pyrophosphate = ATP-energy + DNA-extern + GDP + Thioredoxin |
| 78 | 0.012414781170910327 | 2 | false | 2 | 2 ATP-energy + Sulfate = 3-Phosphoadenylylsulfate + ADP-energy + Pyrophosphate |
| 79 | 0.02071046953045297 | 1 | false | 1 | ATP-energy + Ca2+IN + H2O = ADP-energy + Orthophosphate |
| 80 | 0.01105236496032247 | 1 | false | 1 | Ca2+IN + H2O + ITP-extern = IMP + Pyrophosphate |
| 81 | 0.016748309469321043 | 1 | false | 1 | H2O + UTP = Pyrophosphate + UMP |
| 82 | 6.818332352701573E-4 | 7 | false | 7 | ADP-energy + CO2 + IMP + NH3 + Phosphoenolpyruvate = (S)-Malate + ATP-energy + GMP |
| 83 | 0.002331856128874543 | 5 | false | 5 | no net reaction |
| 84 | 0.018780980362019584 | 2 | false | 2 | ATP-energy + NH3 + Uridine = ADP-energy + CMP + H2O |
| 85 | 0.006564626807817553 | 2 | false | 2 | GMP + H2O = Guanine + alpha-D-Ribose1-phosphate |
| 86 | 0.9999576902783548 | 2 | false | 2 | DNA-G + IQ-143= DNA-G_blocked + IQ-_used |
| 87 | 0.007141838537199963 | 4 | false | 4 | AMP-energy + H2O + Pyrophosphate = 5-Phospho-alpha-D-ribose1-diphosphate + Hypoxanthine + NH3 |
| 88 | 5.023155754058095E-4 | 3 | false | 3 | ATP-energy + NH3 + Uridine = ADP-energy + CMP + H2O |
| 89 | 0.9404019561579331 | 2 | false | 2 | DNA-A + IQ-143= DNA-A_blocked + IQ-_used |
| 90 | 0.003684070716048704 | 3 | false | 3 | ADP-energy + IQ-143+ Pyrophosphate + RNA-C + UDP = ATP-energy + CDP + IQ-_used + Orthophosphate + RNA-U_blocked |
| 91 | 0.00900774932214976 | 3 | false | 3 | 5-Phospho-alpha-D-ribose1-diphosphate + H2O = Pyrophosphate + alpha-D-Ribose1-phosphate |
| 92 | 0.9052578090052474 | 1 | false | 1 | ATP-energy + IQ-143+ Sulfate = Adenylylsulfate_blocked + IQ-_used + Pyrophosphate |
| 93 | 0.0023795055965034617 | 1 | false | 1 | GTP + Pyruvate = GDP + Phosphoenolpyruvate |
| 94 | 0.02743226904519125 | 1 | false | 1 | Ca2+IN + H2O + UDP = Orthophosphate + UMP |
| 95 | 0.0021222770499396137 | 3 | false | 3 | ATP-energy + NH3 + Uridine = ADP-energy + CMP + H2O |
| 96 | 0.044015805264594254 | 1 | false | 1 | Ca2+IN + GDP + H2O = GMP + Orthophosphate |
| 97 | 0.0030431229120700953 | 2 | false | 2 | ATP-energy + H2O = ADP-energy + Orthophosphate |
| 98 | 0.016630177019761216 | 1 | false | 1 | H2O + UMP = Orthophosphate + Uridine |
| 99 | 0.003106060318267767 | 2 | false | 2 | ATP-energy + Pyruvate = ADP-energy + Phosphoenolpyruvate |
| 100 | 0.023123882315774336 | 8 | false | 8 | 510-Methylenetetrahydrofolate + DNA-C + H2O = DNA-T + Dihydrofolate + NH3 |
| 101 | 0.002200615066273004 | 3 | false | 3 | ATP-energy + NH3 + Uridine = ADP-energy + CMP + H2O |
| 102 | 0.007652047024873321 | 3 | false | 3 | ATP-energy + H2O = AMP-energy + Pyrophosphate |
| 103 | 0.011688263659672793 | 1 | false | 1 | ATP-energy + NH3 + Xanthosine5-phosphate = AMP-energy + GMP + Pyrophosphate |
| 104 | 0.0033953448234627093 | 2 | false | 2 | GTP + NH3 + Uridine = CMP + GDP + H2O |
| 105 | 0.0031390977125216324 | 4 | false | 4 | 2 ATP-energy + HCO3- + L-Aspartate + L-Glutamine + Oxygen = 2 ADP-energy + H2O2 + L-Glutamate + Orotate + 2 Orthophosphate |
| 106 | 0.014404549605998684 | 1 | false | 1 | Ca2+IN + GTP + H2O = GDP + Orthophosphate |
| 107 | 0.023822985645377437 | 2 | false | 2 | NH3 + UTP + Uridine = CMP + H2O + UDP |
| 108 | 0.005334194545468796 | 3 | false | 3 | 2 ATP-energy + H2O = 2 ADP-energy + Pyrophosphate |
| 109 | 0.006813723317181775 | 3 | false | 3 | ATP-energy + Ca2+IN + H2O = ADP-energy + Orthophosphate |
| 110 | 0.0123874464089746 | 2 | false | 2 | ATP-energy + Uridine = ADP-energy + UMP |
| 111 | 0.004462246738856868 | 2 | false | 2 | ATP-energy + Ca2+IN + H2O = ADP-energy + Orthophosphate |
| 112 | 0.00387935641331405 | 4 | false | 4 | no net reaction |
| 113 | 4.530603317655091E-4 | 2 | false | 2 | NH3 + UTP = CDP + Orthophosphate |
| 114 | 0.02115380878234141 | 1 | false | 1 | ATP-energy + H2O + L-Glutamine + Xanthosine5-phosphate = AMP-energy + GMP + L-Glutamate + Pyrophosphate |
| 115 | 0.0013293192726026026 | 3 | false | 3 | ADP-energy + ADP-metabolism + IQ-143+ Pyrophosphate + RNA-C = ATP-energy + CDP + IQ-_used + Orthophosphate + RNA-A_blocked |
| 116 | 0.001546238801111044 | 12 | false | 12 | 5-Phospho-alpha-D-ribose1-diphosphate + ATP-energy + DNA-A + NH3 + Uracil = ADP-energy + AMP-energy + DNA-C + Orthophosphate + Pyrophosphate |
| 117 | 0.006849424772078727 | 2 | false | 2 | 5-Phospho-alpha-D-ribose1-diphosphate + H2O + Hypoxanthine + Oxygen = H2O2 + Pyrophosphate + Xanthosine5-phosphate |
| 118 | 0.04704490543092743 | 1 | false | 1 | ATP-energy + IQ-143= ADP-energy + IQ-_deactivated |
| 119 | 0.009287293737837987 | 1 | false | 1 | GTP + Uridine = GDP + UMP |
| 120 | 0.006619911129784106 | 2 | false | 2 | H2O + IMP = Hypoxanthine + alpha-D-Ribose1-phosphate |
| 121 | 0.02191206586226513 | 2 | false | 2 | CMP + 2 H2O = NH3 + Orthophosphate + Uridine |
| 122 | 0.0158196922577456 | 1 | false | 1 | ATP-energy + IQ-143= ADP-energy + IQ-_deactivated |
| 123 | 0.01415519903558382 | 2 | false | 2 | ATP-energy + Uridine = ADP-energy + UMP |
| 124 | 0.01644168087774822 | 1 | false | 1 | ATP-energy + Pyruvate = ADP-energy + Phosphoenolpyruvate |
| 125 | 7.815613069988014E-4 | 3 | false | 3 | 2 ATP-energy + H2O = 2 ADP-energy + Pyrophosphate |
| 126 | 0.053833488904838744 | 2 | false | 2 | ATP-energy + H2O = AMP-energy + Pyrophosphate |
| 127 | 0.001402091186954224 | 3 | false | 3 | ATP-energy + H2O = ADP-energy + Orthophosphate |
| 128 | 0.2188669341228312 | 3 | false | 3 | ADP-energy + ADP-metabolism + Pyrophosphate + RNA-C = ATP-energy + CDP + Orthophosphate + RNA-A |
| 129 | 0.02926510063971466 | 1 | false | 1 | ATP-metabolism + H2O = ADP-metabolism + Orthophosphate |
| 130 | 0.010179196296325466 | 1 | false | 1 | ATP-energy + IQ-143= ADP-energy + IQ-_deactivated |
| 131 | 0.0111746841194863 | 3 | false | 3 | ADP-energy + IQ-143+ Pyrophosphate + RNA-C = ATP-energy + IQ-_used + Orthophosphate + RNA-C_blocked |
| 132 | 0.021019182823906024 | 2 | false | 2 | 2 H2O + P1P4-Bis(5-xanthosyl)tetraphosphate = Pyrophosphate + 2 Xanthosine5-phosphate |
| 133 | 0.021994073293478134 | 2 | false | 2 | GTP + H2O = GMP + Pyrophosphate |
| 134 | 0.06968296119203132 | 1 | false | 1 | ATP-energy + IQ-143= ADP-energy + IQ-_deactivated |
| 135 | 0.04474208869152774 | 1 | false | 1 | ATP-energy + Uridine = ADP-energy + UMP |
| 136 | 0.03445053484672955 | 4 | false | 4 | DNA-A + H2O + Oxidizedthioredoxin + Pyrophosphate = ATP-energy + DNA-extern + Thioredoxin |
| 137 | 0.006484915953373527 | 3 | false | 3 | ATP-energy + NH3 + Uridine = ADP-energy + CMP + H2O |
| 138 | 0.008035694031373719 | 2 | false | 2 | H2O + Oxygen + Pyrophosphate + Xanthosine5-phosphate = 5-Phospho-alpha-D-ribose1-diphosphate + H2O2 + Urate |
| 139 | 0.004374721814733973 | 2 | false | 2 | H2O + L-Glutamine + UTP = CDP + L-Glutamate + Orthophosphate |
| 140 | 0.9648928850467682 | 2 | false | 2 | DNA-T + IQ-143= DNA-T_blocked + IQ-_used |
| 141 | 0.004871280551854706 | 1 | false | 1 | ATP-energy + IQ-143= ADP-energy + IQ-_deactivated |
| 142 | 0.012602828385737341 | 1 | false | 1 | Ca2+IN + H2O + UTP = Orthophosphate + UDP |
| 143 | 0.010884819015411384 | 7 | false | 7 | ADP-energy + DNA-C + 2 H2O + Oxidizedthioredoxin + Pyrophosphate = ATP-energy + DNA-extern + NH3 + Thioredoxin + UDP |
| 144 | 0.00878438293861894 | 7 | false | 7 | ADP-energy + CO2 + Phosphoenolpyruvate + Thioredoxin = (S)-Malate + ATP-energy + Oxidizedthioredoxin |
| 145 | 0.014298632472019679 | 1 | false | 1 | GTP + H2O = GMP + Pyrophosphate |
| 146 | 0.042079271834483456 | 1 | false | 1 | AMP-energy + H2O = IMP + NH3 |
| 147 | 0.012812062622998388 | 3 | false | 3 | AMP-energy + ATP-energy + Pyrophosphate + alpha-D-Ribose1-phosphate = 5-Phospho-alpha-D-ribose1-diphosphate + ADP-energy + AMP-metabolism + Orthophosphate |
| 148 | 0.536120821107934 | 3 | false | 3 | ADP-energy + GDP + Pyrophosphate + RNA-C = ATP-energy + CDP + Orthophosphate + RNA-G |
| 149 | 0.014607669409517632 | 1 | false | 1 | UTP + Uridine = UDP + UMP |
| 150 | 0.007971935340850722 | 3 | false | 3 | 2 ATP-energy + H2O = 2 ADP-energy + Pyrophosphate |
| 151 | 0.9837572375860835 | 2 | false | 2 | DNA-C + IQ-143= DNA-C_blocked + IQ-_used |
| 152 | 0.005321509682737813 | 11 | false | 11 | DNA-C + Guanine + H2O = DNA-G + NH3 + Uracil |
| 153 | 0.0033649574875739274 | 1 | false | 1 | Allantoate + H2O = Allantoine |
| 154 | 0.003864486961261848 | 32 | false | 19 | 5 ADP-energy + CO2 + H2O + Orthophosphate + 4 Phosphoenolpyruvate + Pyruvate + Ubiquinone = 3 (S)-Malate + 5 ATP-energy + Fumarate + Ubiquinol |
| 155 | 0.014927222858377998 | 1 | false | 1 | Ca2+IN + H2O + UDP = Orthophosphate + UMP |

^1^ This data shows the elementary mode Analysis for *H. sapiens* with 1.25µM IQ-143:

VI. Extreme modes with changed activity after administration of IQ-143

Table S16: Extreme modes (EMs) with a significantly higher activity in *S. epidermidis* after IQ-143 administration^1^.

| EM Number | Pathlength | Reactions |
| --- | --- | --- |
| 17 | 1 | (1 TCA_fumarate-hydratase) |
| 35 | 2 | (1 TCA_citrate-hydro-lyase) (-1 TCA_citrate-hydroxymutase) |
| 36 | 3 | (1 Glyc_lipoic_acetyltransferase) (-1 TCA_citrate-hydro-lyase) (1 TCA_citrate_synthase) |
| 40 | 1 | (1 PurM_IMP_L-aspartate-ligase) |
| 41 | 1 | (1 Glyc_Succinate-CoA-ligase) |
| 48 | 1 | (1 PurM_ATP_dCDP-phosphotransferase) |
| 63 | 1 | (1 PurM_adenylylsulfate-kinase) |
| 74 | 1 | (1 PyrM_uridine-kinase_dTTP) |
| 77 | 2 | (1 PurM_deoxyadenosine-kinase_ATP) (-1 PyrM_deoxyadenosine-phosphorylase) |
| 84 | 2 | (1 Glyc_6-phosphofructokinase) (1 Glyc_fructose-bisphosphatase) |
| 94 | 1 | (1 PurM_thioredoxin-oxidoreductase_dGDP) |
| 96 | 2 | (2 PurM_nucleoside-diphosphate-phosphotransferase_ATP) (1 SERP0688-spermidine/putrescine-transport_import) |
| 97 | 3 | (1 PurM_5-nucleotidase_dCMP) (1 PyrM_Deoxycytidine-aminohydrolase) (1 PyrM_deoxyuridine-phosphorylase) |
| 99 | 1 | (1 PyrM_dUTP-diphosphohydrolase) |
| 103 | 1 | (1 PurM_5-nucleotidase_UMP) |
| 107 | 2 | (1 PurM_5-nucleotidase_dAMP) (1 PyrM_deoxyadenosine-phosphorylase) |
| 112 | 1 | (1 PurM_UTP-diphosphohydrolase) |
| 119 | 1 | (1 AS_Leucine) |
| 139 | 1 | (1 PurM_5-nucleotidase_dTMP) |
| 141 | 1 | (1 PyrM_uridine-kinase_ATP) |
| 143 | 6 | (-1 Glyc_acetaldehyde-dehydrogenase_NAD+) (-1 Glyc_Actetate-CoA-ligase) (2 Glyc_dihydrolipoamide-dehydrogenase) (-1 SERP0389-Glyc_Ethanol_NAD+-oxidoreductase) (-1 TCA_citrate-hydro-lyase) (1 TCA_citrate_synthase) |
| 147 | 3 | (1 AMP-energy_to_AMP-metabolism) (1 PurM_adenylate-kinase_AMP) (2 PurM_thioredoxin-oxidoreductase_dADP) |
| 149 | 1 | (1 PyrM_cytidine-kinase_ATP) |
| 151 | 3 | (1 IQ-143-extern_to_IQ-) (1 SERP0765-Uracil-permease-transport_import) (1 SERP1944-MultiDrug-transport_efflux) |
| 158 | 1 | (1 PurM_GTP-pyrophosphokinase) |
| 172 | 4 | (1 AMP-energy_to_AMP-metabolism) (1 PurM_adenylate-kinase_AMP) (2 PurM_DNA-directed-RNA-polyermase_ATP) (2 PurM_nucleoside-diphosphate-phosphotransferase_ATP) |
| 179 | 1 | (1 PyrM_cytidine-kinase_GTP) |
| 192 | 1 | (1 PurM_5-nucleotidase_CMP) |

^1^ The listed EMs of *S. epidermidis* showed a higher activity when 0,16µM and 1,25µM IQ-143were administered.

Left column: Number of specific mode in the full EM model (Tables S10-S12).

Middle colum: Number of enzymes involved in formation of affected EM.

Table S17: EMs with a significantly lower activity in *S. epidermidis* after IQ-143 administration^1^.

| EM Number | Pathlength | Reactions |
| --- | --- | --- |
| 3 | 1 | (1 PurM_ATP_IDP-phosphotransferase) |
| 19 | 3 | (1 PurM_IMP-pyrophosphorylase) (-1 PurM_nucleotide-phosphatase_Inosine) (1 PurM_nucleotide-phosphatase_Xanthosine) |
| 45 | 1 | (1 PurM_GMP-pyrophosphorylase) |
| 52 | 1 | (1 PyrM_UMP-pyrophosphorylase) |
| 61 | 2 | (-1 AMP-energy_to_AMP-metabolism) (1 PurM_adenylosuccinate-lyase) |
| 79 | 1 | (1 PyrM_dUTP-diphosphatase) |
| 93 | 1 | (1 SERP0841-PurM_PNPase_GDP) |
| 100 | 1 | (1 PyrM_dUTP-diphosphohydrolase) |
| 102 | 2 | (1 PurM_pyruvate-phosphotransferase_GTP) (-1 TCA_PEP-carboxylase) |
| 116 | 2 | (-1 AMP-energy_to_AMP-metabolism) (1 PurM_deoxycytidine-kinase_ATP) |
| 117 | 1 | (1 PurM_thioredoxin-oxidoreductase_dGTP) |
| 122 | 2 | (1 SERP1997-formate/nitrite-transport_efflux/import) (1 SERP2179-choline/betaine/carnitine-transp_efflux) |
| 127 | 1 | (1 PyrM_uridine-kinase_UTP) |
| 133 | 1 | (1 AS_Serine_to_Pyruvate) |
| 140 | 4 | (1 PurM_D-Ribose-1,5-phosphomutase) (-1 PurM_IMP-pyrophosphorylase) (1 PurM_nucleotide-phosphatase_Inosine) (1 PurM_PRPP-synthetase) |
| 148 | 2 | (1 PurM_GDP-reductase) (1 SERP0765-Uracil-permease-transport_import) |
| 150 | 2 | (1 SERP0765-Uracil-permease-transport_import) (1 SERP1997-formate/nitrite-transport_efflux/import) |
| 154 | 7 | (2 Glyc_acetaldehyde-dehydrogenase_NAD+) (2 Glyc_Actetate-CoA-ligase) (1 OP_complex1) (1 OP_complex3) (2 SERP0389-Glyc_Ethanol_NAD+-oxidoreductase) (2 TCA_citrate-hydro-lyase) (-2 TCA_citrate_synthase) |
| 155 | 7 | (3 Glyc_acetaldehyde-dehydrogenase_NAD+) (3 Glyc_Actetate-CoA-ligase) (1 OP_complex2) (1 OP_complex3) (3 SERP0389-Glyc_Ethanol_NAD+-oxidoreductase) (3 TCA_citrate-hydro-lyase) (-3 TCA_citrate_synthase) |
| 161 | 2 | (1 AS_Serine_to_Cysteine) (1 Glyc_Actetate-CoA-ligase) |
| 197 | 1 | (1 PyrM_uridine-kinase_GTP) |

^1^ The listed EMs of *S. epidermidis* showed a lower activity when 0,16µM and 1,25µM IQ-143were administered.

Left column: Number of specific mode in the full EM model (Tables S10-S12).

Middle colum: Number of enzymes involved in formation of affected EM.

Table S18: EMs with a significantly higher activity in *S. aureus* after IQ-143 administration^1^.

| EM Number | Pathlength | Reactions |
| --- | --- | --- |
| 49 | 1 | (1 PyrM_UMP-pyrophosphorylase) |
| 50 | 1 | (1 PyrM_nucleoside-triphosphate-adenylate-kinase) |
| 51 | 1 | (1 PurM_XMP-pyrophosphorylase) |
| 52 | 2 | (1 PyrM_Deoxycytidine-aminohydrolase) (-1 PyrM_Deoxycytidine-deaminase) |
| 53 | 2 | (-1 PurM_nucleotide-phosphatase_Deoxyadenosine) (1 PyrM_deoxyadenosine-phosphorylase) |
| 54 | 1 | (1 PyrM_ATP_dUDP_thymidylate-kinase) |
| 61 | 7 | (-1 AMP-energy_to_AMP-metabolism) (-2 Glyc_alpha-D-Glucose-6-phosphate-ketol-isomerase2) (-2 Glyc_ATP-alpha-D-glucokinase) (2 Glyc_ATP-beta-D-glucokinase) (2 Glyc_beta-D-Glucose-6-phosphate-ketol-isomerase) (2 Glyc_D-Glucose-1-epimerase) (-1 PurM_adenylate-kinase_AMP) |
| 79 | 1 | (1 AS_Aspartate_to_beta-Alanine) |
| 90 | 1 | (1 DNA-extern_to_DNA-intern) |
| 96 | 2 | (2 PurM_nucleoside-diphosphate-phosphotransferase_ATP) (1 SERP0688-spermidine/putrescine-transport_import) |
| 98 | 2 | (1 PurM_nucleoside-diphosphate-phosphotransferase_ATP) (1 SERP0686-spermidine/putrescine-transport_import) |
| 103 | 1 | (1 PurM_5-nucleotidase_UMP) |
| 109 | 1 | (1 PurM_DNA-directed-RNA-polyermase_UTP) |
| 116 | 1 | (1 PurM_thioredoxin-oxidoreductase_dGTP) |
| 122 | 2 | (1 SERP1997-formate/nitrite-transport_efflux/import) (1 SERP2179-choline/betaine/carnitine-transp_efflux) |
| 130 | 1 | (1 PurM_allantoinase) |
| 137 | 1 | (1 PurM_thioredoxin-oxidoreductase_dCDP) |
| 139 | 1 | (1 PurM_5-nucleotidase_dTMP) |
| 140 | 4 | (1 PurM_D-Ribose-1,5-phosphomutase) (-1 PurM_IMP-pyrophosphorylase) (1 PurM_nucleotide-phosphatase_Inosine) (1 PurM_PRPP-synthetase) |
| 141 | 1 | (1 PyrM_uridine-kinase_ATP) |
| 143 | 6 | (-1 Glyc_acetaldehyde-dehydrogenase_NAD+) (-1 Glyc_Actetate-CoA-ligase) (2 Glyc_dihydrolipoamide-dehydrogenase) (-1 SERP0389-Glyc_Ethanol_NAD+-oxidoreductase) (-1 TCA_citrate-hydro-lyase) (1 TCA_citrate_synthase) |
| 146 | 1 | (1 PurM_urea-amidohydrolase) |
| 147 | 3 | (1 AMP-energy_to_AMP-metabolism) (1 PurM_adenylate-kinase_AMP) (2 PurM_thioredoxin-oxidoreductase_dADP) |
| 148 | 1 | (1 AS_Aspartate_to_Asparagine) |
| 149 | 1 | (1 PyrM_cytidine-kinase_ATP) |
| 151 | 3 | (1 IQ-143-extern_to_IQ-) (1 SERP0765-Uracil-permease-transport_import) (1 SERP1944-MultiDrug-transport_efflux) |
| 158 | 1 | (1 PurM_GTP-pyrophosphokinase) |
| 180 | 3 | (1 FA_Deg_C16_to_Acetyl-CoA) (6 TCA_citrate-hydro-lyase) (-6 TCA_citrate_synthase) |
| 198 | 1 | (1 PyrM_uridine-kinase_GTP) |

^1^ The listed EMs of *S. aureus* showed a higher activity after administration of 0,16µM and 1,25µM IQ-143.

Left column: Number of specific mode in the full EM model (Tables S7-S9).

Middle colum: Number of enzymes involved in formation of affected EM.

Table S19: EMs with a significantly lower activity in *S. aureus* after IQ-143 administration^1^.

| EM Number | Pathlength | Reactions |
| --- | --- | --- |
| 19 | 3 | (1 PurM_IMP-pyrophosphorylase) (1 PurM_nucleotide-phosphatase_Guanosine) (-1 PurM_nucleotide-phosphatase_Inosine) |
| 25 | 1 | (1 PyrM_nucleoside-phosphate-kinase_ATP) |
| 58 | 6 | (1 AMP-energy_to_AMP-metabolism) (2 Glyc_alpha-D-Glucose-6-phosphate-ketol-isomerase) (2 Glyc_ATP-alpha-D-glucokinase) (-2 Glyc_ATP-beta-D-glucokinase) (-2 Glyc_D-Glucose-1-epimerase) (1 PurM_adenylate-kinase_AMP) |
| 73 | 1 | (1 SERP0831-PurM_DNA-directed-DNA-polymerase_dATP) |
| 82 | 1 | (1 PurM_thioredoxin-oxidoreductase_dUTP) |
| 93 | 2 | (1 PurM_carbamate-kinase_ATP) (1 PyrM_aspartate-carbamoyltransferase) |
| 102 | 2 | (1 PurM_pyruvate-phosphotransferase_dATP) (-1 TCA_PEP-carboxylase) |
| 133 | 1 | (1 PyrM_uridine-kinase_dATP) |
| 150 | 2 | (1 PurM_GDP-reductase) (1 SERP0765-Uracil-permease-transport_import) |
| 154 | 1 | (1 PurM_XTP-diphosphohydrolase) |
| 161 | 6 | (-3 Glyc_acetaldehyde-dehydrogenase_NAD+) (-3 Glyc_Actetate-CoA-ligase) (2 OP_complex5) (-3 SERP0389-Glyc_Ethanol_NAD+-oxidoreductase) (-3 TCA_citrate-hydro-lyase) (3 TCA_citrate_synthase) |
| 184 | 1 | (1 PurM_thioredoxin-oxidoreductase_dUDP) |
| 196 | 1 | (1 PyrM_cytidine-kinase_dATP) |

^1^ The listed EMs of *S. aureus* showed a lower activity after administration of 0,16µM and 1,25µM IQ-143.

Left column: Number of specific mode in the full EM model (Tables S7-S9).

Middle colum: Number of enzymes involved in formation of affected EM.

**VII: Measured concentrations of nucleotides and NAD(P)H/NAD(P)^+^**

Figure S4: Measured concentrations of NAD(P)H/NAD(P)^+^


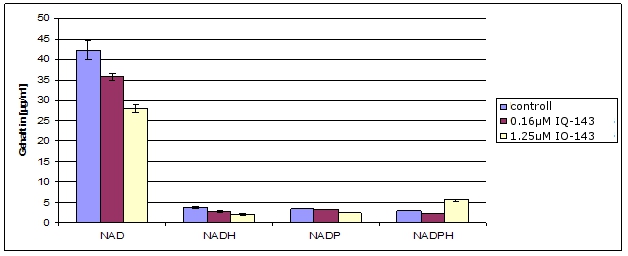


Calibration:

|  | Staph. control | | Staph. 0.16 µm  IQ-143 | | Staph. 1.25 µM  IQ-143 | |
| --- | --- | --- | --- | --- | --- | --- |
| [µg/ml] | MW | sdv | MW | sdv | MW | sdv |
| NAD | 42.19 | 2.45 | 44.08 | 0.93 | 34.38 | 0.06 |
| NADH | 3.71 | 0.25 | 2.63 | 0.04 | 1.95 | 0.09 |
| NADP | 3.47 | 0.06 | 3.24 | 0.11 | 2.42 | 0.12 |
| NADPH | 2.87 | 0.98 | 2.25 | 0.90 | 5.56 | 0.87 |

Figure S5: Measured concentrations of nucleotides.

Calibration:

|  | Staph. Kontrolle | | Staph. 0.16 µm | | Staph. 1.25 µM | | Trimethoprim | |
| --- | --- | --- | --- | --- | --- | --- | --- | --- |
|  | MW | sdv | MW | sdv | MW | sdv | MW | sdv |
| CMP | 21.03 | 0.96 | 24.41 | 0.24 | 3.86 | 0.19 | 7.66 | 0.56 |
| AMP | 0.42 | 0.06 | 0.12 | 0.02 | 20.37 | 0.80 | 3.58 | 0.84 |
| GMP | 1.51 | 0.05 | 1.44 | 0.05 | 3.55 | 0.21 | 5.79 | 0.29 |
| XMP | 2.62 | 0.2 | 3.96 | 0.16 | 3.44 | 0.11 | 4.22 | 0.16 |
| TMP | 1.61 | 0.12 | 1.67 | 0.11 | 8.81 | 0.24 | 3.96 | 0.15 |

**VIII: Calculated enzyme actvities**

Table S20: Calculated enzyme activities for *S. aureus* USA300*.*

|  |  |  |  |  |  |  |
| --- | --- | --- | --- | --- | --- | --- |
| S. AUREUS | 0.00 µM | 0.00 µM | 0.16 µM | 0.16 µM | 1.25 µM | 1.25 µM |
| Name | Raw flux | Normalized flux | Raw flux | Normalized flux | Raw flux | Normalized flux |
| ADP-energy_to_ADP-metabolism | 0.1580 | 0.0791 | 0.1532 | 0.0520 | 0.1331 | 0.0619 |
| AMP-energy_to_AMP-metabolism | 0.2884 | 0.1444 | 0.3869 | 0.1314 | 0.4212 | 0.1960 |
| AS_Acetyl-CoA_to_L-Leucine | 0.0822 | 0.0411 | 0.0781 | 0.0265 | 0.0685 | 0.0319 |
| AS_Acetyl-CoA_to_L-Valine | 0.0790 | 0.0396 | 0.0766 | 0.0260 | 0.0665 | 0.0310 |
| AS_Alanine_to_Pyruvate | 0.0790 | 0.0396 | 0.0766 | 0.0260 | 0.0665 | 0.0310 |
| AS_Aspartate_to_Alanine | 0.0790 | 0.0396 | 0.0766 | 0.0260 | 0.0665 | 0.0310 |
| AS_Aspartate_to_Arginine | 0.0790 | 0.0396 | 0.0766 | 0.0260 | 0.0665 | 0.0310 |
| AS_Aspartate_to_Asparagine | 0.0474 | 0.0237 | 0.0437 | 0.0148 | 0.1770 | 0.0823 |
| AS_Aspartate_to_beta-Alanine | 0.0790 | 0.0396 | 0.0766 | 0.0260 | 0.0665 | 0.0310 |
| AS_Aspartate_to_Homoserine | 0.0790 | 0.0396 | 0.0766 | 0.0260 | 0.0665 | 0.0310 |
| AS_Glutamate_to_Glutamine | 0.0790 | 0.0396 | 0.0766 | 0.0260 | 0.1464 | 0.0681 |
| AS_Glutamate_to_Proline | 0.0790 | 0.0396 | 0.0766 | 0.0260 | 0.0665 | 0.0310 |
| AS_Histidine_to_Glutamate | 0.0790 | 0.0396 | 0.0766 | 0.0260 | 0.0665 | 0.0310 |
| AS_Homoserine_to_Threonine | 0.0790 | 0.0396 | 0.0766 | 0.0260 | 0.0665 | 0.0310 |
| AS_Isoleucine | 0.0790 | 0.0396 | 0.0766 | 0.0260 | 0.0665 | 0.0310 |
| AS_Leucine | 0.0300 | 0.0150 | 0.0000 | 0.0000 | 0.0359 | 0.0167 |
| AS_Phenylalanin_to_Tyrosine | 0.0790 | 0.0396 | 0.0766 | 0.0260 | 0.0665 | 0.0310 |
| AS_Saccharopine_to_Lysine | 0.0790 | 0.0396 | 0.0766 | 0.0260 | 0.0665 | 0.0310 |
| AS_Serine_to_Cysteine | 0.0790 | 0.0396 | 0.0766 | 0.0260 | 0.0665 | 0.0310 |
| AS_Serine_to_Glycine | 0.0790 | 0.0396 | 0.0766 | 0.0260 | 0.0665 | 0.0310 |
| AS_Serine_to_Methionine | 0.0790 | 0.0396 | 0.0766 | 0.0260 | 0.0665 | 0.0310 |
| AS_Serine_to_Pyruvate | 0.0790 | 0.0396 | 0.0766 | 0.0260 | 0.0665 | 0.0310 |
| AS_Threonine | 0.0790 | 0.0396 | 0.0766 | 0.0260 | 0.0665 | 0.0310 |
| AS_Tryptophan_to_Tryptamine | 0.0790 | 0.0396 | 0.0766 | 0.0260 | 0.0665 | 0.0310 |
| AS_Valine | 0.0790 | 0.0396 | 0.0766 | 0.0260 | 0.0665 | 0.0310 |
| ATP-energy_to_ATP-metabolism | 0.0585 | 0.0293 | 0.0567 | 0.0192 | -0.0852 | -0.0396 |
| DNA-extern_to_DNA-intern | 0.0103 | 0.0051 | 0.0766 | 0.0260 | 0.0692 | 0.0322 |
| DNA_to_DNA-blocked | 0.0000 | 0.0000 | 0.0000 | 0.0000 | 0.0000 | 0.0000 |
| FA_Deg_C16_to_Acetyl-CoA | 0.0593 | 0.0297 | 0.0690 | 0.0234 | 0.0532 | 0.0248 |
| FA_Syn_Acetyl-CoA_to_C16 | 0.0790 | 0.0396 | 0.0766 | 0.0260 | 0.0665 | 0.0310 |
| GBAP-extern_to_GBAP | 0.2765 | 0.1385 | 0.3861 | 0.1311 | 0.3234 | 0.1504 |
| Glyc_2-Phospho-D-glycerate-2.3-phosphomutase | -0.4361 | -0.2184 | -0.9255 | -0.3142 | -0.5350 | -0.2489 |
| Glyc_2-phospho-D-glycerate-hydro-lyase | 0.4361 | 0.2184 | 0.9255 | 0.3142 | 0.5350 | 0.2489 |
| Glyc_6-phospho-beta-glucosidase | 0.1375 | 0.0688 | 0.3708 | 0.1259 | 0.3314 | 0.1542 |
| Glyc_6-phosphofructokinase | 0.2378 | 0.1191 | 0.4819 | 0.1636 | 0.2821 | 0.1313 |
| Glyc_acetaldehyde-dehydrogenase_NAD+ | 0.0340 | 0.0170 | -0.2046 | -0.0695 | -0.0912 | -0.0424 |
| Glyc_Actetate-CoA-ligase | 0.1130 | 0.0566 | -0.1279 | -0.0434 | -0.0246 | -0.0115 |
| Glyc_alpha-D-Glucose-6-phosphate-ketol-isomerase | -0.0790 | -0.0396 | 0.0781 | 0.0265 | -0.0665 | -0.0310 |
| Glyc_alpha-D-Glucose-6-phosphate-ketol-isomerase2 | 0.1043 | 0.0522 | 0.3279 | 0.1113 | 0.1451 | 0.0675 |
| Glyc_ATP-alpha-D-glucokinase | -0.0553 | -0.0277 | 0.3141 | 0.1066 | 0.1424 | 0.0662 |
| Glyc_ATP-beta-D-glucokinase | 0.0553 | 0.0277 | -0.3141 | -0.1066 | -0.1424 | -0.0662 |
| Glyc_beta-D-Glucose-6-phosphate-ketol-isomerase | 0.1138 | 0.0570 | 0.1348 | 0.0458 | 0.1224 | 0.0570 |
| Glyc_D-Glucose-1-epimerase | 0.0553 | 0.0277 | -0.3141 | -0.1066 | -0.1424 | -0.0662 |
| Glyc_D-Glucose-1-epimerase-ketol-isomerase | -0.2180 | -0.1092 | -0.4628 | -0.1571 | -0.2675 | -0.1244 |
| Glyc_dihydrolipoamide-dehydrogenase | 0.1580 | 0.0791 | 0.1532 | 0.0520 | 0.2928 | 0.1362 |
| Glyc_fructose-bisphosphat-aldolase | 0.2180 | 0.1092 | 0.4628 | 0.1571 | 0.2675 | 0.1244 |
| Glyc_fructose-bisphosphatase | 0.0198 | 0.0099 | 0.0192 | 0.0065 | 0.0146 | 0.0068 |
| Glyc_glyceraldehyde-3-P-dehydrogenase_NAD+ | 0.3160 | 0.1582 | 0.3065 | 0.1040 | 0.2662 | 0.1238 |
| Glyc_glyceraldehyde-3-P-dehydrogenase_NADP+ | 0.1201 | 0.0601 | 0.6191 | 0.2102 | 0.2688 | 0.1251 |
| Glyc_lipoic_acetyltransferase | 0.0790 | 0.0396 | 0.0766 | 0.0260 | 0.0665 | 0.0310 |
| Glyc_phosphoglycerate-kinase | -0.4361 | -0.2184 | -0.9255 | -0.3142 | -0.5350 | -0.2489 |
| Glyc_PTS-permease1 | 0.0806 | 0.0404 | 0.0919 | 0.0312 | -0.0639 | -0.0297 |
| Glyc_PTS-permease2 | 0.0790 | 0.0396 | 0.0766 | 0.0260 | 0.0665 | 0.0310 |
| Glyc_pyruvate_dehydrogenase | 0.0790 | 0.0396 | 0.0766 | 0.0260 | 0.0665 | 0.0310 |
| Glyc_Succinate-CoA-ligase | 0.0790 | 0.0396 | 0.0766 | 0.0260 | 0.0665 | 0.0310 |
| N-acylneuraminate-9-phosphatase | 0.0000 | 0.0000 | 0.0000 | 0.0000 | 0.0000 | 0.0000 |
| OP_complex1 | 0.0790 | 0.0396 | 0.0766 | 0.0260 | 0.0665 | 0.0310 |
| OP_complex2 | 0.0790 | 0.0396 | 0.0766 | 0.0260 | 0.0665 | 0.0310 |
| OP_complex3 | 0.1580 | 0.0791 | 0.1532 | 0.0520 | 0.1331 | 0.0619 |
| OP_complex4 | 0.0790 | 0.0396 | 0.0766 | 0.0260 | 0.0665 | 0.0310 |
| OP_complex5 | 0.0427 | 0.0214 | 0.0322 | 0.0109 | 0.0067 | 0.0031 |
| PurM_5-Hydroxyisourate-amidohydrolase | 0.0000 | 0.0000 | 0.0000 | 0.0000 | 0.0000 | 0.0000 |
| PurM_5-nucleotidase_AMP | 0.0790 | 0.0396 | 0.0766 | 0.0260 | 0.0665 | 0.0310 |
| PurM_5-nucleotidase_CMP | 0.0790 | 0.0396 | 0.0766 | 0.0260 | 0.0665 | 0.0310 |
| PurM_5-nucleotidase_dAMP | 0.0387 | 0.0194 | 0.0306 | 0.0104 | 0.0938 | 0.0436 |
| PurM_5-nucleotidase_dCMP | 0.0458 | 0.0229 | 0.0467 | 0.0159 | 0.1011 | 0.0471 |
| PurM_5-nucleotidase_dGMP | 0.0790 | 0.0396 | 0.0766 | 0.0260 | 0.0665 | 0.0310 |
| PurM_5-nucleotidase_dTMP | 0.0300 | 0.0150 | 0.0000 | 0.0000 | 0.0393 | 0.0183 |
| PurM_5-nucleotidase_GMP | 0.0790 | 0.0396 | 0.0766 | 0.0260 | 0.0665 | 0.0310 |
| PurM_5-nucleotidase_IMP | 0.0790 | 0.0396 | 0.0766 | 0.0260 | 0.0665 | 0.0310 |
| PurM_5-nucleotidase_UMP | 0.0790 | 0.0396 | 0.0766 | 0.0260 | 0.1597 | 0.0743 |
| PurM_5-nucleotidase_XMP | 0.0790 | 0.0396 | 0.0766 | 0.0260 | 0.0665 | 0.0310 |
| PurM_adenylate-kinase_AMP | 0.1801 | 0.0902 | 0.2819 | 0.0957 | 0.3267 | 0.1520 |
| PurM_adenylate-kinase_dAMP | 0.0577 | 0.0289 | 0.0605 | 0.0205 | 0.0632 | 0.0294 |
| PurM_adenylosuccinate-lyase | 0.0300 | 0.0150 | 0.0291 | 0.0099 | 0.0240 | 0.0111 |
| PurM_adenylosuccinate-lyase2 | 0.0000 | 0.0000 | 0.0000 | 0.0000 | 0.0000 | 0.0000 |
| PurM_adenylylsulfate-kinase | 0.0790 | 0.0396 | 0.0766 | 0.0260 | 0.0665 | 0.0310 |
| PurM_ADP-ribose-ribophosphohydrolase | 0.0198 | 0.0099 | 0.0192 | 0.0065 | 0.0146 | 0.0068 |
| PurM_AICAR-pyrophosphate-phosphoribosyltransferase | 0.0000 | 0.0000 | 0.0000 | 0.0000 | 0.0000 | 0.0000 |
| PurM_AIR-carboxylase | 0.0000 | 0.0000 | 0.0000 | 0.0000 | 0.0000 | 0.0000 |
| PurM_allantoinase | 0.0474 | 0.0237 | 0.1073 | 0.0364 | 0.0932 | 0.0433 |
| PurM_AMP-pyrophosphorylase2 | 0.0569 | 0.0285 | 0.0674 | 0.0229 | 0.0612 | 0.0285 |
| PurM_ATP-phosphohydrolase | 0.0790 | 0.0396 | 0.0766 | 0.0260 | 0.0665 | 0.0310 |
| PurM_ATP_CDP-phosphotransferase | 0.0790 | 0.0396 | 0.0766 | 0.0260 | 0.0665 | 0.0310 |
| PurM_ATP_dADP-phosphotransferase | 0.0790 | 0.0396 | 0.0766 | 0.0260 | 0.0665 | 0.0310 |
| PurM_ATP_dCDP-phosphotransferase | 0.0790 | 0.0396 | 0.0766 | 0.0260 | 0.0665 | 0.0310 |
| PurM_ATP_dGDP-phosphotransferase | 0.0790 | 0.0396 | 0.0766 | 0.0260 | 0.0665 | 0.0310 |
| PurM_ATP_dIDP-phosphotransferase | 0.0790 | 0.0396 | 0.0766 | 0.0260 | 0.0665 | 0.0310 |
| PurM_ATP_dTDP-phosphotransferase | 0.0790 | 0.0396 | 0.0766 | 0.0260 | 0.0665 | 0.0310 |
| PurM_ATP_dUDP-phosphotransferase | 0.0790 | 0.0396 | 0.0766 | 0.0260 | 0.0665 | 0.0310 |
| PurM_ATP_GMP-guanylate-kinase | 0.0790 | 0.0396 | 0.0766 | 0.0260 | 0.0665 | 0.0310 |
| PurM_ATP_GMP_guanylate-kinase | 0.0790 | 0.0396 | 0.0766 | 0.0260 | 0.0665 | 0.0310 |
| PurM_ATP_GTP-phosphotransferase | 0.0790 | 0.0396 | 0.0766 | 0.0260 | 0.0665 | 0.0310 |
| PurM_ATP_IDP-phosphotransferase | 0.0790 | 0.0396 | 0.0766 | 0.0260 | 0.0665 | 0.0310 |
| PurM_ATP_UTP-phosphotransferase | 0.0790 | 0.0396 | 0.0766 | 0.0260 | 0.0665 | 0.0310 |
| PurM_carbamate-kinase_ATP | -0.0055 | -0.0028 | -0.0329 | -0.0112 | -0.0087 | -0.0040 |
| PurM_D-Ribose-1.5-phosphomutase | 0.0095 | 0.0047 | 0.0000 | 0.0000 | 0.0639 | 0.0297 |
| PurM_deoxyadenosine-kinase_ATP | 0.0790 | 0.0396 | 0.0766 | 0.0260 | 0.0665 | 0.0310 |
| PurM_deoxycytidine-kinase_ATP | 0.0790 | 0.0396 | 0.0766 | 0.0260 | 0.0665 | 0.0310 |
| PurM_deoxycytidine-kinase_ATP2 | 0.0790 | 0.0396 | 0.0766 | 0.0260 | 0.0665 | 0.0310 |
| PurM_dGTP-diphosphohydrolase | 0.0790 | 0.0396 | 0.0766 | 0.0260 | 0.0665 | 0.0310 |
| PurM_dITP-diphosphohydrolase | 0.0300 | 0.0150 | 0.1548 | 0.0525 | 0.0313 | 0.0145 |
| PurM_DNA-directed-RNA-polyermase_ATP | 0.1580 | 0.0791 | 0.0000 | 0.0000 | 0.0000 | 0.0000 |
| PurM_DNA-directed-RNA-polyermase_CTP | 0.0790 | 0.0396 | 0.0766 | 0.0260 | 0.0665 | 0.0310 |
| PurM_DNA-directed-RNA-polyermase_GTP | 0.0790 | 0.0396 | 0.0766 | 0.0260 | 0.0665 | 0.0310 |
| PurM_DNA-directed-RNA-polyermase_UTP | 0.0569 | 0.0285 | 0.0674 | 0.0229 | 0.0612 | 0.0285 |
| PurM_GDP-reductase | 0.1825 | 0.0914 | 0.1686 | 0.0572 | 0.0998 | 0.0464 |
| PurM_GMP-pyrophosphorylase2 | 0.0790 | 0.0396 | 0.0766 | 0.0260 | 0.0665 | 0.0310 |
| PurM_GTP-diphosphohydrolase | 0.0790 | 0.0396 | 0.0766 | 0.0260 | 0.0665 | 0.0310 |
| PurM_GTP-pyrophosphokinase | 0.0174 | 0.0087 | 0.0766 | 0.0260 | 0.1371 | 0.0638 |
| PurM_IMP-cyclohydrolase | 0.0000 | 0.0000 | 0.0000 | 0.0000 | 0.0000 | 0.0000 |
| PurM_IMP-dehydrogenase | 0.0980 | 0.0491 | 0.0881 | 0.0299 | 0.0838 | 0.0390 |
| PurM_IMP-pyrophosphorylase | 0.4645 | 0.2326 | 0.4597 | 0.1561 | 0.2994 | 0.1393 |
| PurM_IMP_L-aspartate-ligase | 0.0790 | 0.0396 | 0.0766 | 0.0260 | 0.0665 | 0.0310 |
| PurM_ITP-diphosphohydrolase | 0.0790 | 0.0396 | 0.0766 | 0.0260 | 0.0665 | 0.0310 |
| PurM_metaphosphatase | 0.0790 | 0.0396 | 0.0766 | 0.0260 | 0.0665 | 0.0310 |
| PurM_nucleoside-diphosphate-phosphotransferase_ATP | 0.5688 | 0.2848 | 0.4122 | 0.1399 | 0.5536 | 0.2576 |
| PurM_nucleotide-phosphatase_Adenine | 0.0790 | 0.0396 | 0.0766 | 0.0260 | 0.0665 | 0.0310 |
| PurM_nucleotide-phosphatase_Deoxyadenosine | 0.0261 | 0.0131 | -0.0299 | -0.0101 | -0.0266 | -0.0124 |
| PurM_nucleotide-phosphatase_Deoxyguanosine | -0.0790 | -0.0396 | -0.0766 | -0.0260 | -0.0665 | -0.0310 |
| PurM_nucleotide-phosphatase_Deoxyinosine | -0.0790 | -0.0396 | -0.0766 | -0.0260 | -0.0665 | -0.0310 |
| PurM_nucleotide-phosphatase_Deoxyuridine | 0.1051 | 0.0526 | 0.1019 | 0.0346 | 0.0885 | 0.0412 |
| PurM_nucleotide-phosphatase_Guanosine | 0.0790 | 0.0396 | 0.0766 | 0.0260 | 0.0306 | 0.0142 |
| PurM_nucleotide-phosphatase_Inosine | -0.3855 | -0.1931 | -0.3831 | -0.1301 | -0.2329 | -0.1083 |
| PurM_nucleotide-phosphatase_Xanthosine | 0.0790 | 0.0396 | 0.0766 | 0.0260 | 0.0665 | 0.0310 |
| PurM_PRPP-synthetase | 0.0292 | 0.0146 | 0.0192 | 0.0065 | 0.0785 | 0.0365 |
| PurM_pyruvate-phosphotransferase_ATP | 0.0790 | 0.0396 | 0.0766 | 0.0260 | 0.0665 | 0.0310 |
| PurM_pyruvate-phosphotransferase_dATP | 0.0458 | 0.0229 | 0.0475 | 0.0161 | 0.0106 | 0.0050 |
| PurM_pyruvate-phosphotransferase_dGTP | 0.0790 | 0.0396 | 0.0766 | 0.0260 | 0.0665 | 0.0310 |
| PurM_pyruvate-phosphotransferase_GTP | 0.0790 | 0.0396 | 0.0766 | 0.0260 | 0.0665 | 0.0310 |
| PurM_SAICAR-synthetase | 0.0000 | 0.0000 | 0.0000 | 0.0000 | 0.0000 | 0.0000 |
| PurM_thioredoxin-oxidoreductase_dADP | 0.1580 | 0.0791 | 0.1532 | 0.0520 | 0.2928 | 0.1362 |
| PurM_thioredoxin-oxidoreductase_dATP | 0.1580 | 0.0791 | 0.1532 | 0.0520 | 0.1331 | 0.0619 |
| PurM_thioredoxin-oxidoreductase_dCDP | 0.0435 | 0.0218 | 0.0437 | 0.0148 | 0.0566 | 0.0263 |
| PurM_thioredoxin-oxidoreductase_dCTP | 0.0790 | 0.0396 | 0.0766 | 0.0260 | 0.0665 | 0.0310 |
| PurM_thioredoxin-oxidoreductase_dGDP | 0.0126 | 0.0063 | 0.0176 | 0.0060 | 0.0260 | 0.0121 |
| PurM_thioredoxin-oxidoreductase_dGTP | 0.0095 | 0.0047 | 0.0000 | 0.0000 | 0.0240 | 0.0111 |
| PurM_thioredoxin-oxidoreductase_dUDP | 0.0213 | 0.0107 | 0.0161 | 0.0055 | 0.0033 | 0.0015 |
| PurM_thioredoxin-oxidoreductase_dUTP | 0.0790 | 0.0396 | 0.0766 | 0.0260 | 0.0665 | 0.0310 |
| PurM_urea-amidohydrolase | 0.0790 | 0.0396 | 0.0766 | 0.0260 | 0.1464 | 0.0681 |
| PurM_UTP-diphosphohydrolase | 0.0790 | 0.0396 | 0.0766 | 0.0260 | 0.1910 | 0.0888 |
| PurM_xanthosine-phosphoribosyltransferase | 0.0790 | 0.0396 | 0.0766 | 0.0260 | 0.0665 | 0.0310 |
| PurM_XMP-ligase | 0.0790 | 0.0396 | 0.0766 | 0.0260 | 0.0665 | 0.0310 |
| PurM_XMP-pyrophosphorylase | -0.0261 | -0.0131 | 0.0299 | 0.0101 | 0.0266 | 0.0124 |
| PurM_XMP_L-glutamine-amide-ligase | 0.0790 | 0.0396 | 0.0766 | 0.0260 | 0.0665 | 0.0310 |
| PurM_XTP-diphosphohydrolase | 0.0450 | 0.0225 | 0.0414 | 0.0140 | 0.0000 | 0.0000 |
| PyrM_2.3-cyclic-nucleotidase_CMP | 0.0198 | 0.0099 | 0.0192 | 0.0065 | 0.0186 | 0.0087 |
| PyrM_2.3-cyclic-nucleotidase_UMP | 0.0790 | 0.0396 | 0.0766 | 0.0260 | 0.0665 | 0.0310 |
| PyrM_AMP-pyrophosphorylase | 0.1011 | 0.0506 | 0.0858 | 0.0291 | 0.0719 | 0.0334 |
| PyrM_aspartate-carbamoyltransferase | 0.0300 | 0.0150 | 0.0000 | 0.0000 | 0.0013 | 0.0006 |
| PyrM_ATP_dTDP_thymidylate-kinase | 0.0790 | 0.0396 | 0.0766 | 0.0260 | 0.0665 | 0.0310 |
| PyrM_ATP_dUDP_thymidylate-kinase | -0.0261 | -0.0131 | 0.0299 | 0.0101 | 0.0266 | 0.0124 |
| PyrM_CO2_L-glutamine-amido-ligase | 0.0356 | 0.0178 | 0.0329 | 0.0112 | 0.0100 | 0.0046 |
| PyrM_CTP-synthase | 0.0790 | 0.0396 | 0.0766 | 0.0260 | 0.0665 | 0.0310 |
| PyrM_cytidilate-kinase_CTP | 0.0790 | 0.0396 | 0.0383 | 0.0130 | 0.0665 | 0.0310 |
| PyrM_cytidilate-kinase_dCMP | 0.0790 | 0.0396 | 0.0766 | 0.0260 | 0.0665 | 0.0310 |
| PyrM_cytidine-aminohydrolase | 0.0790 | 0.0396 | 0.0766 | 0.0260 | 0.0665 | 0.0310 |
| PyrM_cytidine-kinase | 0.0790 | 0.0396 | 0.0766 | 0.0260 | 0.0665 | 0.0310 |
| PyrM_cytidine-kinase_ATP | 0.0324 | 0.0162 | 0.0207 | 0.0070 | 0.0845 | 0.0393 |
| PyrM_cytidine-kinase_dATP | 0.0853 | 0.0427 | 0.0421 | 0.0143 | 0.0346 | 0.0161 |
| PyrM_cytidine-kinase_dCTP | 0.0790 | 0.0396 | 0.0766 | 0.0260 | 0.0665 | 0.0310 |
| PyrM_cytidine-kinase_dGTP | 0.1011 | 0.0506 | 0.0858 | 0.0291 | 0.0719 | 0.0334 |
| PyrM_cytidine-kinase_dTTP | 0.0790 | 0.0396 | 0.0766 | 0.0260 | 0.0665 | 0.0310 |
| PyrM_cytidine-kinase_dUTP | 0.0790 | 0.0396 | 0.0766 | 0.0260 | 0.0665 | 0.0310 |
| PyrM_cytidine-kinase_GTP | 0.0790 | 0.0396 | 0.0766 | 0.0260 | 0.0665 | 0.0310 |
| PyrM_cytidine-kinase_ITP | 0.0790 | 0.0396 | 0.0766 | 0.0260 | 0.0665 | 0.0310 |
| PyrM_cytidine-ribohydrolase | 0.0000 | 0.0000 | 0.0000 | 0.0000 | 0.0000 | 0.0000 |
| PyrM_dCMP-aminohydrolase | 0.0790 | 0.0396 | 0.0766 | 0.0260 | 0.0665 | 0.0310 |
| PyrM_deoxyadenosine-phosphorylase | -0.0664 | -0.0332 | -0.0161 | -0.0055 | 0.0539 | 0.0251 |
| PyrM_Deoxycytidine-aminohydrolase | -0.0593 | -0.0297 | 0.0000 | 0.0000 | 0.0612 | 0.0285 |
| PyrM_Deoxycytidine-deaminase | 0.0261 | 0.0131 | -0.0299 | -0.0101 | -0.0266 | -0.0124 |
| PyrM_deoxyguanosine-phosphorylase | 0.1580 | 0.0791 | 0.1532 | 0.0520 | 0.1331 | 0.0619 |
| PyrM_deoxyinosine-phosphorylase | 0.0790 | 0.0396 | 0.0766 | 0.0260 | 0.0665 | 0.0310 |
| PyrM_deoxyuridine-phosphorylase | -0.0593 | -0.0297 | -0.0552 | -0.0187 | 0.0126 | 0.0059 |
| PyrM_dihydroorotase | -0.0790 | -0.0396 | -0.0766 | -0.0260 | -0.0665 | -0.0310 |
| PyrM_dihydroorotate-oxidase | 0.0790 | 0.0396 | 0.0766 | 0.0260 | 0.0665 | 0.0310 |
| PyrM_dUMP-phosphotransferase | 0.0790 | 0.0396 | 0.0766 | 0.0260 | 0.0665 | 0.0310 |
| PyrM_dUTP-diphosphatase | 0.0419 | 0.0210 | 0.0391 | 0.0133 | 0.0180 | 0.0084 |
| PyrM_dUTP-diphosphohydrolase | 0.0790 | 0.0396 | 0.0766 | 0.0260 | 0.1783 | 0.0830 |
| PyrM_GMP-pyrophosphorylase | -0.0514 | -0.0257 | -0.0506 | -0.0172 | -0.0439 | -0.0204 |
| PyrM_nucleoside-phosphate-kinase_ATP | 0.0790 | 0.0396 | -0.0437 | -0.0148 | -0.0393 | -0.0183 |
| PyrM_nucleoside-phosphate-kinase_ATP2 | 0.0790 | 0.0396 | 0.0766 | 0.0260 | 0.0665 | 0.0310 |
| PyrM_nucleoside-triphosphate-adenylate-kinase | -0.0261 | -0.0131 | 0.0299 | 0.0101 | 0.0266 | 0.0124 |
| PyrM_OMP-decarboxylase | 0.0790 | 0.0396 | 0.0766 | 0.0260 | 0.0665 | 0.0310 |
| PyrM_orotate-phosphoribosyltransferase | 0.0790 | 0.0396 | 0.0766 | 0.0260 | 0.0665 | 0.0310 |
| PyrM_pyrimidine-nucleoside-phosphorylase | 0.0790 | 0.0396 | 0.0766 | 0.0260 | 0.0665 | 0.0310 |
| PyrM_thioredoxin-reductase | 0.1580 | 0.0791 | 0.1532 | 0.0520 | 0.1331 | 0.0619 |
| PyrM_thymidilate-synthase | 0.0000 | 0.0000 | 0.0000 | 0.0000 | 0.0000 | 0.0000 |
| PyrM_thymidine-kinase_dTMP | 0.0790 | 0.0396 | 0.0766 | 0.0260 | 0.0665 | 0.0310 |
| PyrM_thymidine-kinase_dUMP | -0.0790 | -0.0396 | -0.0766 | -0.0260 | -0.0665 | -0.0310 |
| PyrM_thymidine-phosphorylase | 0.0790 | 0.0396 | 0.0766 | 0.0260 | 0.0665 | 0.0310 |
| PyrM_UMP-pyrophosphorylase | -0.0687 | -0.0344 | -0.0299 | -0.0101 | -0.0240 | -0.0111 |
| PyrM_uridine-kinase_ATP | 0.0300 | 0.0150 | 0.0000 | 0.0000 | 0.0759 | 0.0353 |
| PyrM_uridine-kinase_dATP | 0.0790 | 0.0396 | 0.0360 | 0.0122 | 0.0299 | 0.0139 |
| PyrM_uridine-kinase_dCTP | 0.0593 | 0.0297 | 0.0575 | 0.0195 | 0.0479 | 0.0223 |
| PyrM_uridine-kinase_dGTP | 0.0790 | 0.0396 | 0.0766 | 0.0260 | 0.0665 | 0.0310 |
| PyrM_uridine-kinase_dTTP | 0.0790 | 0.0396 | 0.0766 | 0.0260 | 0.0665 | 0.0310 |
| PyrM_uridine-kinase_dUTP | 0.0790 | 0.0396 | 0.0766 | 0.0260 | 0.0233 | 0.0108 |
| PyrM_uridine-kinase_GTP | 0.0790 | 0.0396 | 0.0766 | 0.0260 | 0.1876 | 0.0873 |
| PyrM_uridine-kinase_ITP | 0.0790 | 0.0396 | 0.0766 | 0.0260 | 0.0665 | 0.0310 |
| PyrM_uridine-kinase_UTP | 0.1185 | 0.0593 | 0.1149 | 0.0390 | 0.0998 | 0.0464 |
| PyrM_uridine-phosphorylase | 0.0790 | 0.0396 | 0.0766 | 0.0260 | 0.0665 | 0.0310 |
| PyrM_uridine-ribohydrolase | 0.0000 | 0.0000 | 0.0000 | 0.0000 | 0.0000 | 0.0000 |
| PyrM_UTP_L-glutamine-amido-ligase | 0.0198 | 0.0099 | 0.0192 | 0.0065 | 0.0146 | 0.0068 |
| SERP0290-zinc-transport_efflux | 0.0790 | 0.0396 | 0.0766 | 0.0260 | 0.0665 | 0.0310 |
| SERP0291-zinc-transporter_import | 0.0790 | 0.0396 | 0.0766 | 0.0260 | 0.0665 | 0.0310 |
| SERP0292-iron-dicitrate-transporter_import | 0.0790 | 0.0396 | 0.0414 | 0.0140 | 0.0286 | 0.0133 |
| SERP0389-Glyc_Ethanol_NAD+-oxidoreductase | 0.0340 | 0.0170 | -0.2046 | -0.0695 | -0.0912 | -0.0424 |
| SERP0653-PurM_FGAM-synthethase | 0.0000 | 0.0000 | 0.0000 | 0.0000 | 0.0000 | 0.0000 |
| SERP0655-PurM_amidophosphoribosyltransferase | 0.0000 | 0.0000 | 0.0000 | 0.0000 | 0.0000 | 0.0000 |
| SERP0656-PurM_AIR_synthetase | 0.0000 | 0.0000 | 0.0000 | 0.0000 | 0.0000 | 0.0000 |
| SERP0657-PurM_GAR-formyltransferase | 0.0000 | 0.0000 | 0.0000 | 0.0000 | 0.0000 | 0.0000 |
| SERP0658-PurM_AICAR-formyltransferase | 0.0000 | 0.0000 | 0.0000 | 0.0000 | 0.0000 | 0.0000 |
| SERP0659-PurM_phosphoribosylamine-glycine-ligase | 0.0000 | 0.0000 | 0.0000 | 0.0000 | 0.0000 | 0.0000 |
| SERP0686-spermidine/putrescine-transport_import | 0.0790 | 0.0396 | 0.0766 | 0.0260 | 0.0665 | 0.0310 |
| SERP0687-spermidine/putrescine-transport_import | 0.0126 | 0.0063 | 0.0176 | 0.0060 | 0.0353 | 0.0164 |
| SERP0688-spermidine/putrescine-transport_import | 0.0245 | 0.0123 | 0.0253 | 0.0086 | 0.0326 | 0.0152 |
| SERP0765-Uracil-permease-transport_import | 0.1936 | 0.0969 | 0.1854 | 0.0629 | 0.1697 | 0.0789 |
| SERP0831-PurM_DNA-directed-DNA-polymerase_dATP | 0.0790 | 0.0396 | 0.0766 | 0.0260 | 0.0260 | 0.0121 |
| SERP0831-PurM_DNA-directed-DNA-polymerase_dCTP | 0.0790 | 0.0396 | 0.0766 | 0.0260 | 0.0665 | 0.0310 |
| SERP0831-PurM_DNA-directed-DNA-polymerase_dGTP | 0.0790 | 0.0396 | 0.0766 | 0.0260 | 0.0665 | 0.0310 |
| SERP0831-PurM_DNA-directed-DNA-polymerase_dTTP | 0.0790 | 0.0396 | 0.0766 | 0.0260 | 0.0665 | 0.0310 |
| SERP0841-PurM_PNPase_ADP | 0.1580 | 0.0791 | 0.1532 | 0.0520 | 0.1331 | 0.0619 |
| SERP0841-PurM_PNPase_GDP | 0.0529 | 0.0265 | 0.0513 | 0.0174 | 0.0446 | 0.0207 |
| SERP1403-MultiDrug-transport_efflux | 0.0988 | 0.0495 | 0.0843 | 0.0286 | 0.0798 | 0.0371 |
| SERP1802-cobalt/nickel-transport_efflux | 0.0790 | 0.0396 | 0.0766 | 0.0260 | 0.0665 | 0.0310 |
| SERP1803-cobalt/nickel-transport_efflux | 0.0790 | 0.0396 | 0.0766 | 0.0260 | 0.0665 | 0.0310 |
| SERP1944-MultiDrug-transport_efflux | 0.0988 | 0.0495 | 0.2406 | 0.0817 | 0.1903 | 0.0885 |
| SERP1951-lipoprotein-transport_efflux/import | 0.0790 | 0.0396 | 0.0766 | 0.0260 | 0.0665 | 0.0310 |
| SERP1952-macrolide-transport_efflux | 0.0790 | 0.0396 | 0.0766 | 0.0260 | 0.1530 | 0.0712 |
| SERP1997-formate/nitrite-transport_efflux/import | 0.1161 | 0.0582 | 0.1724 | 0.0585 | 0.2163 | 0.1006 |
| SERP2060-glyerol-transport_import | 0.0790 | 0.0396 | 0.0766 | 0.0260 | 0.0665 | 0.0310 |
| SERP2156-Glyc_L-lactate-dehydrogenase | 0.1580 | 0.0791 | 0.1532 | 0.0520 | 0.1331 | 0.0619 |
| SERP2179-choline/betaine/carnitine-transp_efflux | 0.1059 | 0.0530 | 0.3080 | 0.1046 | 0.2528 | 0.1176 |
| SERP2186-PurM_ATP_sulfate-adenylyltransferase | 0.0790 | 0.0396 | 0.0766 | 0.0260 | 0.0665 | 0.0310 |
| SERP2283-phopsphonate-transport_import | 0.0790 | 0.0396 | 0.0766 | 0.0260 | 0.0665 | 0.0310 |
| SERP2289-MultiDrug-transport_efflux | 0.0790 | 0.0396 | 0.0613 | 0.0208 | 0.0532 | 0.0248 |
| TCA_citrate-hydro-lyase | -0.2489 | -0.1246 | -0.4068 | -0.1381 | -0.3081 | -0.1433 |
| TCA_citrate-hydroxymutase | -0.0758 | -0.0380 | -0.0751 | -0.0255 | -0.0645 | -0.0300 |
| TCA_citrate_synthase | 0.3247 | 0.1626 | 0.4819 | 0.1636 | 0.3726 | 0.1734 |
| TCA_fumarate-hydratase | 0.0758 | 0.0380 | 0.0751 | 0.0255 | 0.0645 | 0.0300 |
| TCA_isocitrate-hydro-lyase | 0.0790 | 0.0396 | 0.0766 | 0.0260 | 0.0665 | 0.0310 |
| TCA_lipoic-transsuccinylase | -0.2370 | -0.1187 | -0.2298 | -0.0780 | -0.1996 | -0.0929 |
| TCA_Oxidoreductase | 0.1580 | 0.0791 | 0.1532 | 0.0520 | 0.1331 | 0.0619 |
| TCA_oxoglutarate-dehydrogenase-complex1 | 0.2370 | 0.1187 | 0.2298 | 0.0780 | 0.1996 | 0.0929 |
| TCA_oxoglutarate-dehydrogenase-complex2 | 0.2370 | 0.1187 | 0.2298 | 0.0780 | 0.1996 | 0.0929 |
| TCA_oxoglutarate-synthase | 0.0790 | 0.0396 | 0.0766 | 0.0260 | 0.0665 | 0.0310 |
| TCA_PEP-carboxylase | -0.7189 | -0.3600 | -1.2029 | -0.4084 | -0.7452 | -0.3467 |
| TCA_Pyruvate_CO2-ligase | -0.0356 | -0.0178 | -0.0329 | -0.0112 | -0.0100 | -0.0046 |
| TCA_pyruvate_dehydrogenase | 0.0790 | 0.0396 | 0.0766 | 0.0260 | 0.0665 | 0.0310 |

Table S21: Calculated enzyme activities of *S. epidermidis* RP62A*.*

| S. EPIDERMIDIS | 0.00 µM | 0.00 µM | 0.16 µM | 0.16 µM | 1.25 µM | 1.25 µM |
| --- | --- | --- | --- | --- | --- | --- |
| Name | Raw flux | Normalized flux | Raw flux | Normalized flux | Raw flux | Normalized flux |
| ADP-energy_to_ADP-metabolism | 0.1581 | 0.0669 | 0.1581 | 0.0669 | 0.1317 | 0.0585 |
| AMP-energy_to_AMP-metabolism | -0.0293 | -0.0124 | -0.0293 | -0.0124 | -0.0191 | -0.0085 |
| AS_Acetyl-CoA_to_L-Leucine | 0.0791 | 0.0334 | 0.0791 | 0.0334 | 0.0658 | 0.0292 |
| AS_Acetyl-CoA_to_L-Valine | 0.0395 | 0.0167 | 0.0395 | 0.0167 | 0.0494 | 0.0219 |
| AS_Alanine_to_Pyruvate | 0.0791 | 0.0334 | 0.0791 | 0.0334 | 0.0658 | 0.0292 |
| AS_Aspartate_to_Alanine | 0.0791 | 0.0334 | 0.0791 | 0.0334 | 0.0658 | 0.0292 |
| AS_Aspartate_to_Arginine | 0.0791 | 0.0334 | 0.0791 | 0.0334 | 0.0658 | 0.0292 |
| AS_Aspartate_to_Asparagine | 0.0791 | 0.0334 | 0.0791 | 0.0334 | 0.1449 | 0.0643 |
| AS_Aspartate_to_beta-Alanine | 0.0791 | 0.0334 | 0.0791 | 0.0334 | 0.0658 | 0.0292 |
| AS_Aspartate_to_Homoserine | 0.0791 | 0.0334 | 0.0791 | 0.0334 | 0.0658 | 0.0292 |
| AS_Glutamate_to_Glutamine | 0.0791 | 0.0334 | 0.0791 | 0.0334 | 0.0658 | 0.0292 |
| AS_Glutamate_to_Proline | 0.0791 | 0.0334 | 0.0791 | 0.0334 | 0.0658 | 0.0292 |
| AS_Histidine_to_Glutamate | 0.0791 | 0.0334 | 0.0791 | 0.0334 | 0.0658 | 0.0292 |
| AS_Homoserine_to_Threonine | 0.0791 | 0.0334 | 0.0791 | 0.0334 | 0.0658 | 0.0292 |
| AS_Isoleucine | 0.0791 | 0.0334 | 0.0791 | 0.0334 | 0.0658 | 0.0292 |
| AS_Leucine | 0.0285 | 0.0120 | 0.0285 | 0.0120 | 0.0586 | 0.0260 |
| AS_Phenylalanin_to_Tyrosine | 0.0198 | 0.0084 | 0.0198 | 0.0084 | 0.0165 | 0.0073 |
| AS_Saccharopine_to_Lysine | 0.0791 | 0.0334 | 0.0791 | 0.0334 | 0.0658 | 0.0292 |
| AS_Serine_to_Cysteine | 0.0522 | 0.0221 | 0.0522 | 0.0221 | 0.0171 | 0.0076 |
| AS_Serine_to_Glycine | 0.0791 | 0.0334 | 0.0791 | 0.0334 | 0.0658 | 0.0292 |
| AS_Serine_to_Methionine | 0.0791 | 0.0334 | 0.0791 | 0.0334 | 0.0658 | 0.0292 |
| AS_Serine_to_Pyruvate | 0.0791 | 0.0334 | 0.0791 | 0.0334 | 0.0290 | 0.0129 |
| AS_Threonine | 0.0791 | 0.0334 | 0.0791 | 0.0334 | 0.0658 | 0.0292 |
| AS_Tryptophan_to_Tryptamine | 0.0791 | 0.0334 | 0.0791 | 0.0334 | 0.0658 | 0.0292 |
| AS_Valine | 0.0380 | 0.0161 | 0.0380 | 0.0161 | 0.0975 | 0.0433 |
| ATP-energy_to_ATP-metabolism | 0.0791 | 0.0334 | 0.0791 | 0.0334 | 0.0658 | 0.0292 |
| DNA-extern_to_DNA-intern | 0.0791 | 0.0334 | 0.0791 | 0.0334 | 0.0658 | 0.0292 |
| DNA_to_DNA-blocked | 0.0000 | 0.0000 | 0.0000 | 0.0000 | 0.0000 | 0.0000 |
| FA_Deg_C16_to_Acetyl-CoA | 0.0791 | 0.0334 | 0.0791 | 0.0334 | 0.0658 | 0.0292 |
| FA_Syn_Acetyl-CoA_to_C16 | 0.0198 | 0.0084 | 0.0198 | 0.0084 | 0.0165 | 0.0073 |
| GBAP-extern_to_GBAP | 0.2594 | 0.1097 | 0.2594 | 0.1097 | 0.3615 | 0.1605 |
| Glyc_2-Phospho-D-glycerate-2.3-phosphomutase | -0.2151 | -0.0910 | -0.2151 | -0.0910 | -0.1831 | -0.0813 |
| Glyc_2-phospho-D-glycerate-hydro-lyase | 0.2151 | 0.0910 | 0.2151 | 0.0910 | 0.1831 | 0.0813 |
| Glyc_6-phospho-beta-glucosidase | 0.0285 | 0.0120 | 0.0285 | 0.0120 | 0.0257 | 0.0114 |
| Glyc_6-phosphofructokinase | 0.1273 | 0.0538 | 0.1273 | 0.0538 | 0.1080 | 0.0479 |
| Glyc_acetaldehyde-dehydrogenase_NAD+ | -0.0221 | -0.0094 | -0.0221 | -0.0094 | -0.2799 | -0.1243 |
| Glyc_Actetate-CoA-ligase | 0.0300 | 0.0127 | 0.0300 | 0.0127 | -0.2627 | -0.1167 |
| Glyc_alpha-D-Glucose-6-phosphate-ketol-isomerase | 0.0791 | 0.0334 | 0.0791 | 0.0334 | 0.0658 | 0.0292 |
| Glyc_alpha-D-Glucose-6-phosphate-ketol-isomerase2 | -0.7639 | -0.3230 | -0.7639 | -0.3230 | -0.6862 | -0.3047 |
| Glyc_ATP-alpha-D-glucokinase | -0.7639 | -0.3230 | -0.7639 | -0.3230 | -0.6862 | -0.3047 |
| Glyc_ATP-beta-D-glucokinase | 0.7639 | 0.3230 | 0.7639 | 0.3230 | 0.6862 | 0.3047 |
| Glyc_beta-D-Glucose-6-phosphate-ketol-isomerase | 0.8714 | 0.3685 | 0.8714 | 0.3685 | 0.7777 | 0.3453 |
| Glyc_D-Glucose-1-epimerase | 0.7639 | 0.3230 | 0.7639 | 0.3230 | 0.6862 | 0.3047 |
| Glyc_D-Glucose-1-epimerase-ketol-isomerase | -0.1075 | -0.0455 | -0.1075 | -0.0455 | -0.0915 | -0.0406 |
| Glyc_dihydrolipoamide-dehydrogenase | 0.1581 | 0.0669 | 0.1581 | 0.0669 | 0.1317 | 0.0585 |
| Glyc_fructose-bisphosphat-aldolase | 0.1075 | 0.0455 | 0.1075 | 0.0455 | 0.0915 | 0.0406 |
| Glyc_fructose-bisphosphatase | 0.0198 | 0.0084 | 0.0198 | 0.0084 | 0.0165 | 0.0073 |
| Glyc_glyceraldehyde-3-P-dehydrogenase_NAD+ | 0.1581 | 0.0669 | 0.1581 | 0.0669 | 0.1317 | 0.0585 |
| Glyc_glyceraldehyde-3-P-dehydrogenase_NADP+ | 0.0569 | 0.0241 | 0.0569 | 0.0241 | 0.0514 | 0.0228 |
| Glyc_lipoic_acetyltransferase | 0.0720 | 0.0304 | 0.0720 | 0.0304 | 0.0658 | 0.0292 |
| Glyc_phosphoglycerate-kinase | -0.2151 | -0.0910 | -0.2151 | -0.0910 | -0.1831 | -0.0813 |
| Glyc_PTS-permease1 | 0.0791 | 0.0334 | 0.0791 | 0.0334 | 0.0658 | 0.0292 |
| Glyc_PTS-permease2 | 0.0593 | 0.0251 | 0.0593 | 0.0251 | 0.0738 | 0.0327 |
| Glyc_pyruvate_dehydrogenase | 0.0380 | 0.0161 | 0.0380 | 0.0161 | 0.1054 | 0.0468 |
| Glyc_Succinate-CoA-ligase | 0.0395 | 0.0167 | 0.0395 | 0.0167 | 0.0494 | 0.0219 |
|  |  |  |  |  |  |  |
| OP_complex1 | 0.0474 | 0.0201 | 0.0474 | 0.0201 | 0.0283 | 0.0126 |
| OP_complex2 | 0.0380 | 0.0161 | 0.0380 | 0.0161 | 0.0112 | 0.0050 |
| OP_complex3 | 0.0854 | 0.0361 | 0.0854 | 0.0361 | 0.0395 | 0.0175 |
| OP_complex4 | 0.0791 | 0.0334 | 0.0791 | 0.0334 | 0.0658 | 0.0292 |
| OP_complex5 | 0.1581 | 0.0669 | 0.1581 | 0.0669 | 0.1317 | 0.0585 |
|  |  |  |  |  |  |  |
| PurM_5-nucleotidase_AMP | 0.0791 | 0.0334 | 0.0791 | 0.0334 | 0.0658 | 0.0292 |
| PurM_5-nucleotidase_CMP | 0.0791 | 0.0334 | 0.0791 | 0.0334 | 0.0658 | 0.0292 |
| PurM_5-nucleotidase_dAMP | 0.0791 | 0.0334 | 0.0791 | 0.0334 | 0.0658 | 0.0292 |
| PurM_5-nucleotidase_dCMP | 0.0791 | 0.0334 | 0.0791 | 0.0334 | 0.0658 | 0.0292 |
| PurM_5-nucleotidase_dGMP | 0.0791 | 0.0334 | 0.0791 | 0.0334 | 0.0658 | 0.0292 |
| PurM_5-nucleotidase_dTMP | 0.0791 | 0.0334 | 0.0791 | 0.0334 | 0.0658 | 0.0292 |
| PurM_5-nucleotidase_GMP | 0.0380 | 0.0161 | 0.0380 | 0.0161 | 0.0362 | 0.0161 |
| PurM_5-nucleotidase_IMP | 0.0791 | 0.0334 | 0.0791 | 0.0334 | 0.0658 | 0.0292 |
| PurM_5-nucleotidase_UMP | 0.0791 | 0.0334 | 0.0791 | 0.0334 | 0.0658 | 0.0292 |
| PurM_5-nucleotidase_XMP | 0.0791 | 0.0334 | 0.0791 | 0.0334 | 0.0658 | 0.0292 |
| PurM_adenylate-kinase_AMP | -0.2238 | -0.0946 | -0.2238 | -0.0946 | -0.2114 | -0.0939 |
| PurM_adenylate-kinase_dAMP | 0.0727 | 0.0308 | 0.0727 | 0.0308 | 0.0606 | 0.0269 |
| PurM_adenylosuccinate-lyase | -0.0514 | -0.0217 | -0.0514 | -0.0217 | -0.0606 | -0.0269 |
| PurM_adenylosuccinate-lyase2 | 0.0000 | 0.0000 | 0.0000 | 0.0000 | 0.0000 | 0.0000 |
| PurM_adenylylsulfate-kinase | 0.0791 | 0.0334 | 0.0791 | 0.0334 | 0.0658 | 0.0292 |
| PurM_ADP-ribose-ribophosphohydrolase | 0.0791 | 0.0334 | 0.0791 | 0.0334 | 0.0658 | 0.0292 |
| PurM_AICAR-pyrophosphate-phosphoribosyltransferase | 0.0000 | 0.0000 | 0.0000 | 0.0000 | 0.0000 | 0.0000 |
| PurM_AIR-carboxylase | 0.0000 | 0.0000 | 0.0000 | 0.0000 | 0.0000 | 0.0000 |
|  |  |  |  |  |  |  |
| PurM_AMP-pyrophosphorylase | 0.0727 | 0.0308 | 0.0727 | 0.0308 | 0.0606 | 0.0269 |
| PurM_AMP-pyrophosphorylase2 | 0.0854 | 0.0361 | 0.0854 | 0.0361 | 0.0711 | 0.0316 |
| PurM_ATP-phosphohydrolase | 0.1581 | 0.0669 | 0.1581 | 0.0669 | 0.1317 | 0.0585 |
| PurM_ATP_CDP-phosphotransferase | 0.0791 | 0.0334 | 0.0791 | 0.0334 | 0.0658 | 0.0292 |
| PurM_ATP_dADP-phosphotransferase | 0.0791 | 0.0334 | 0.0791 | 0.0334 | 0.0658 | 0.0292 |
| PurM_ATP_dCDP-phosphotransferase | 0.0791 | 0.0334 | 0.0791 | 0.0334 | 0.0658 | 0.0292 |
| PurM_ATP_dGDP-phosphotransferase | 0.0791 | 0.0334 | 0.0791 | 0.0334 | 0.0658 | 0.0292 |
| PurM_ATP_dIDP-phosphotransferase | 0.0791 | 0.0334 | 0.0791 | 0.0334 | 0.0658 | 0.0292 |
| PurM_ATP_dTDP-phosphotransferase | 0.0791 | 0.0334 | 0.0791 | 0.0334 | 0.0658 | 0.0292 |
| PurM_ATP_dUDP-phosphotransferase | 0.0791 | 0.0334 | 0.0791 | 0.0334 | 0.0658 | 0.0292 |
| PurM_ATP_GMP-guanylate-kinase | 0.0791 | 0.0334 | 0.0791 | 0.0334 | 0.0658 | 0.0292 |
| PurM_ATP_GMP_guanylate-kinase | 0.0791 | 0.0334 | 0.0791 | 0.0334 | 0.0658 | 0.0292 |
| PurM_ATP_GTP-phosphotransferase | 0.0791 | 0.0334 | 0.0791 | 0.0334 | 0.0658 | 0.0292 |
| PurM_ATP_IDP-phosphotransferase | 0.0554 | 0.0234 | 0.0554 | 0.0234 | -0.0441 | -0.0196 |
| PurM_ATP_UTP-phosphotransferase | 0.0791 | 0.0334 | 0.0791 | 0.0334 | 0.0658 | 0.0292 |
| PurM_carbamate-kinase_ATP | -0.0467 | -0.0197 | -0.0467 | -0.0197 | 0.0059 | 0.0026 |
| PurM_D-Ribose-1.5-phosphomutase | 0.0150 | 0.0064 | 0.0150 | 0.0064 | 0.0000 | 0.0000 |
| PurM_deoxyadenosine-kinase_ATP | 0.0791 | 0.0334 | 0.0791 | 0.0334 | 0.0658 | 0.0292 |
| PurM_deoxycytidine-kinase_ATP | 0.0150 | 0.0064 | 0.0150 | 0.0064 | 0.0000 | 0.0000 |
| PurM_deoxycytidine-kinase_ATP2 | 0.0791 | 0.0334 | 0.0791 | 0.0334 | 0.0658 | 0.0292 |
| PurM_dGTP-diphosphohydrolase | 0.0380 | 0.0161 | 0.0380 | 0.0161 | 0.0316 | 0.0140 |
| PurM_dITP-diphosphohydrolase | 0.0791 | 0.0334 | 0.0791 | 0.0334 | 0.0658 | 0.0292 |
| PurM_DNA-directed-RNA-polyermase_CTP | 0.0791 | 0.0334 | 0.0791 | 0.0334 | 0.0658 | 0.0292 |
| PurM_DNA-directed-RNA-polyermase_GTP | 0.0285 | 0.0120 | 0.0285 | 0.0120 | 0.0981 | 0.0436 |
| PurM_DNA-directed-RNA-polyermase_UTP | 0.0791 | 0.0334 | 0.0791 | 0.0334 | 0.0658 | 0.0292 |
| PurM_DNA-directed-RNA-polymerase_ATP | 0.0791 | 0.0334 | 0.0791 | 0.0334 | 0.0658 | 0.0292 |
| PurM_GDP-reductase | 0.2088 | 0.0883 | 0.2088 | 0.0883 | 0.1317 | 0.0585 |
| PurM_GMP-pyrophosphorylase | 0.0577 | 0.0244 | 0.0577 | 0.0244 | 0.0441 | 0.0196 |
| PurM_GMP-pyrophosphorylase2 | 0.0791 | 0.0334 | 0.0791 | 0.0334 | 0.0658 | 0.0292 |
| PurM_GTP-diphosphohydrolase | 0.0791 | 0.0334 | 0.0791 | 0.0334 | 0.0658 | 0.0292 |
| PurM_GTP-pyrophosphokinase | 0.0791 | 0.0334 | 0.0791 | 0.0334 | 0.0658 | 0.0292 |
| PurM_IMP-cyclohydrolase | 0.0000 | 0.0000 | 0.0000 | 0.0000 | 0.0000 | 0.0000 |
| PurM_IMP-dehydrogenase | 0.0988 | 0.0418 | 0.0988 | 0.0418 | 0.1238 | 0.0550 |
| PurM_IMP-pyrophosphorylase | 0.4523 | 0.1913 | 0.4523 | 0.1913 | 0.3556 | 0.1579 |
| PurM_IMP_L-aspartate-ligase | 0.0791 | 0.0334 | 0.0791 | 0.0334 | 0.0658 | 0.0292 |
| PurM_ITP-diphosphohydrolase | 0.0237 | 0.0100 | 0.0237 | 0.0100 | 0.0316 | 0.0140 |
| PurM_metaphosphatase | 0.0791 | 0.0334 | 0.0791 | 0.0334 | 0.0658 | 0.0292 |
| PurM_nucleoside-diphosphate-phosphotransferase_ATP | -0.0759 | -0.0321 | -0.0759 | -0.0321 | 0.0593 | 0.0263 |
| PurM_nucleotide-phosphatase_Adenine | 0.0791 | 0.0334 | 0.0791 | 0.0334 | 0.0658 | 0.0292 |
| PurM_nucleotide-phosphatase_Deoxyadenosine | -0.0419 | -0.0177 | -0.0419 | -0.0177 | -0.0349 | -0.0155 |
| PurM_nucleotide-phosphatase_Deoxyguanosine | -0.0791 | -0.0334 | -0.0791 | -0.0334 | -0.0658 | -0.0292 |
| PurM_nucleotide-phosphatase_Deoxyinosine | -0.0791 | -0.0334 | -0.0791 | -0.0334 | -0.0658 | -0.0292 |
| PurM_nucleotide-phosphatase_Deoxyuridine | -0.0791 | -0.0334 | -0.0791 | -0.0334 | -0.0658 | -0.0292 |
| PurM_nucleotide-phosphatase_Guanosine | 0.0791 | 0.0334 | 0.0791 | 0.0334 | 0.0658 | 0.0292 |
| PurM_nucleotide-phosphatase_Inosine | -0.3732 | -0.1578 | -0.3732 | -0.1578 | -0.2897 | -0.1286 |
| PurM_nucleotide-phosphatase_Xanthosine | 0.0791 | 0.0334 | 0.0791 | 0.0334 | 0.0257 | 0.0114 |
| PurM_PRPP-synthetase | 0.0941 | 0.0398 | 0.0941 | 0.0398 | 0.0658 | 0.0292 |
| PurM_pyruvate-phosphotransferase_ATP | 0.1581 | 0.0669 | 0.1581 | 0.0669 | 0.1317 | 0.0585 |
| PurM_pyruvate-phosphotransferase_dATP | 0.0791 | 0.0334 | 0.0791 | 0.0334 | 0.1515 | 0.0672 |
| PurM_pyruvate-phosphotransferase_dGTP | 0.0411 | 0.0174 | 0.0411 | 0.0174 | 0.0547 | 0.0243 |
| PurM_pyruvate-phosphotransferase_GTP | 0.0277 | 0.0117 | 0.0277 | 0.0117 | 0.0053 | 0.0023 |
| PurM_SAICAR-synthetase | 0.0000 | 0.0000 | 0.0000 | 0.0000 | 0.0000 | 0.0000 |
| PurM_thioredoxin-oxidoreductase_dATP | 0.1581 | 0.0669 | 0.1581 | 0.0669 | 0.1317 | 0.0585 |
| PurM_thioredoxin-oxidoreductase_dCDP | 0.0791 | 0.0334 | 0.0791 | 0.0334 | 0.0658 | 0.0292 |
| PurM_thioredoxin-oxidoreductase_dCTP | 0.0791 | 0.0334 | 0.0791 | 0.0334 | 0.0658 | 0.0292 |
| PurM_thioredoxin-oxidoreductase_dGDP | 0.0791 | 0.0334 | 0.0791 | 0.0334 | 0.0658 | 0.0292 |
| PurM_thioredoxin-oxidoreductase_dGTP | 0.0150 | 0.0064 | 0.0150 | 0.0064 | 0.0000 | 0.0000 |
| PurM_thioredoxin-oxidoreductase_dUDP | 0.0324 | 0.0137 | 0.0324 | 0.0137 | 0.0270 | 0.0120 |
| PurM_thioredoxin-oxidoreductase_dUTP | 0.0791 | 0.0334 | 0.0791 | 0.0334 | 0.0658 | 0.0292 |
| PurM_thioredoxin-oxidoreductased_dADP | 0.1581 | 0.0669 | 0.1581 | 0.0669 | 0.1317 | 0.0585 |
| PurM_urea-amidohydrolase | 0.0791 | 0.0334 | 0.0791 | 0.0334 | 0.1449 | 0.0643 |
| PurM_UTP-diphosphohydrolase | 0.0791 | 0.0334 | 0.0791 | 0.0334 | 0.0658 | 0.0292 |
| PurM_xanthosine-phosphoribosyltransferase | 0.0791 | 0.0334 | 0.0791 | 0.0334 | 0.0658 | 0.0292 |
| PurM_XMP-pyrophosphorylase | 0.0419 | 0.0177 | 0.0419 | 0.0177 | 0.0349 | 0.0155 |
| PurM_XMP_L-glutamine-amide-ligase | 0.0791 | 0.0334 | 0.0791 | 0.0334 | 0.0658 | 0.0292 |
| PurM_XTP-diphosphohydrolase | 0.0791 | 0.0334 | 0.0791 | 0.0334 | 0.0658 | 0.0292 |
|  |  |  |  |  |  |  |
| PyrM_2.3-cyclic-nucleotidase_CMP | 0.0198 | 0.0084 | 0.0198 | 0.0084 | 0.0184 | 0.0082 |
| PyrM_2.3-cyclic-nucleotidase_UMP | 0.0759 | 0.0321 | 0.0759 | 0.0321 | 0.0632 | 0.0281 |
|  |  |  |  |  |  |  |
| PyrM_aspartate-carbamoyltransferase | 0.0324 | 0.0137 | 0.0324 | 0.0137 | 0.0718 | 0.0319 |
| PyrM_ATP_dTDP_thymidylate-kinase | -0.0411 | -0.0174 | -0.0411 | -0.0174 | -0.0342 | -0.0152 |
| PyrM_ATP_dUDP_thymidylate-kinase | 0.0419 | 0.0177 | 0.0419 | 0.0177 | 0.0349 | 0.0155 |
| PyrM_CO2_L-glutamine-amido-ligase | 0.0791 | 0.0334 | 0.0791 | 0.0334 | 0.0658 | 0.0292 |
| PyrM_CTP-synthase | 0.0791 | 0.0334 | 0.0791 | 0.0334 | 0.0658 | 0.0292 |
| PyrM_cytidilate-kinase_CTP | 0.0830 | 0.0351 | 0.0830 | 0.0351 | 0.0731 | 0.0325 |
| PyrM_cytidilate-kinase_dCMP | 0.0791 | 0.0334 | 0.0791 | 0.0334 | 0.0658 | 0.0292 |
| PyrM_cytidine-aminohydrolase | 0.0791 | 0.0334 | 0.0791 | 0.0334 | 0.0658 | 0.0292 |
| PyrM_cytidine-kinase_ATP | 0.0791 | 0.0334 | 0.0791 | 0.0334 | 0.1449 | 0.0643 |
| PyrM_cytidine-kinase_dATP | 0.0443 | 0.0187 | 0.0443 | 0.0187 | 0.0369 | 0.0164 |
| PyrM_cytidine-kinase_dCTP | 0.0791 | 0.0334 | 0.0791 | 0.0334 | 0.0658 | 0.0292 |
| PyrM_cytidine-kinase_dGTP | 0.0791 | 0.0334 | 0.0791 | 0.0334 | 0.0658 | 0.0292 |
| PyrM_cytidine-kinase_dTTP | 0.0791 | 0.0334 | 0.0791 | 0.0334 | 0.1357 | 0.0602 |
| PyrM_cytidine-kinase_dUTP | 0.0380 | 0.0161 | 0.0380 | 0.0161 | 0.0658 | 0.0292 |
| PyrM_cytidine-kinase_GTP | 0.0791 | 0.0334 | 0.0791 | 0.0334 | 0.0658 | 0.0292 |
| PyrM_cytidine-kinase_ITP | 0.0198 | 0.0084 | 0.0198 | 0.0084 | 0.0184 | 0.0082 |
| PyrM_cytidine-kinase_UTP | 0.0791 | 0.0334 | 0.0791 | 0.0334 | 0.1765 | 0.0784 |
| PyrM_cytidine-ribohydrolase | 0.0000 | 0.0000 | 0.0000 | 0.0000 | 0.0000 | 0.0000 |
| PyrM_dCMP-aminohydrolase | 0.0791 | 0.0334 | 0.0791 | 0.0334 | 0.0658 | 0.0292 |
| PyrM_deoxyadenosine-phosphorylase | 0.0419 | 0.0177 | 0.0419 | 0.0177 | 0.0349 | 0.0155 |
| PyrM_Deoxycytidine-aminohydrolase | 0.0419 | 0.0177 | 0.0419 | 0.0177 | 0.0349 | 0.0155 |
| PyrM_Deoxycytidine-deaminase | -0.0419 | -0.0177 | -0.0419 | -0.0177 | -0.0349 | -0.0155 |
| PyrM_deoxyguanosine-phosphorylase | 0.1581 | 0.0669 | 0.1581 | 0.0669 | 0.1317 | 0.0585 |
| PyrM_deoxyinosine-phosphorylase | 0.0791 | 0.0334 | 0.0791 | 0.0334 | 0.0658 | 0.0292 |
| PyrM_deoxyuridine-phosphorylase | -0.0261 | -0.0110 | -0.0261 | -0.0110 | -0.0217 | -0.0096 |
| PyrM_dihydroorotase | -0.0791 | -0.0334 | -0.0791 | -0.0334 | -0.0658 | -0.0292 |
| PyrM_dihydroorotate-oxidase | 0.0791 | 0.0334 | 0.0791 | 0.0334 | 0.0658 | 0.0292 |
| PyrM_dUMP-phosphotransferase | 0.0791 | 0.0334 | 0.0791 | 0.0334 | 0.0658 | 0.0292 |
| PyrM_dUTP-diphosphatase | 0.0435 | 0.0184 | 0.0435 | 0.0184 | 0.0184 | 0.0082 |
| PyrM_dUTP-diphosphohydrolase | 0.0285 | 0.0120 | 0.0285 | 0.0120 | 0.0007 | 0.0003 |
|  |  |  |  |  |  |  |
| PyrM_nucleoside-phosphate-kinase_ATP | 0.0791 | 0.0334 | 0.0791 | 0.0334 | 0.0658 | 0.0292 |
| PyrM_nucleoside-phosphate-kinase_ATP2 | 0.0791 | 0.0334 | 0.0791 | 0.0334 | 0.0658 | 0.0292 |
| PyrM_nucleoside-triphosphate-adenylate-kinase | 0.0625 | 0.0264 | 0.0625 | 0.0264 | 0.0520 | 0.0231 |
| PyrM_OMP-decarboxylase | 0.0791 | 0.0334 | 0.0791 | 0.0334 | 0.1890 | 0.0839 |
| PyrM_orotate-phosphoribosyltransferase | 0.0791 | 0.0334 | 0.0791 | 0.0334 | 0.0658 | 0.0292 |
| PyrM_pyrimidine-nucleoside-phosphorylase | 0.0791 | 0.0334 | 0.0791 | 0.0334 | 0.0658 | 0.0292 |
| PyrM_thioredoxin-reductase | 0.1581 | 0.0669 | 0.1581 | 0.0669 | 0.1317 | 0.0585 |
| PyrM_thymidilate-synthase | 0.0000 | 0.0000 | 0.0000 | 0.0000 | 0.0000 | 0.0000 |
| PyrM_thymidine-kinase_dTMP | 0.0791 | 0.0334 | 0.0791 | 0.0334 | 0.0658 | 0.0292 |
| PyrM_thymidine-kinase_dUMP | 0.1052 | 0.0445 | 0.1052 | 0.0445 | 0.0876 | 0.0389 |
| PyrM_thymidine-phosphorylase | 0.0791 | 0.0334 | 0.0791 | 0.0334 | 0.0658 | 0.0292 |
| PyrM_UMP-pyrophosphorylase | -0.1060 | -0.0448 | -0.1060 | -0.0448 | -0.1146 | -0.0509 |
| PyrM_uridine-kinase_ATP | 0.0285 | 0.0120 | 0.0285 | 0.0120 | 0.0507 | 0.0225 |
| PyrM_uridine-kinase_dATP | 0.0791 | 0.0334 | 0.0791 | 0.0334 | 0.0658 | 0.0292 |
| PyrM_uridine-kinase_dCTP | 0.0593 | 0.0251 | 0.0593 | 0.0251 | 0.0474 | 0.0211 |
| PyrM_uridine-kinase_dGTP | 0.0791 | 0.0334 | 0.0791 | 0.0334 | 0.0658 | 0.0292 |
| PyrM_uridine-kinase_dTTP | 0.0791 | 0.0334 | 0.0791 | 0.0334 | 0.0658 | 0.0292 |
| PyrM_uridine-kinase_dUTP | 0.0791 | 0.0334 | 0.0791 | 0.0334 | 0.0658 | 0.0292 |
| PyrM_uridine-kinase_GTP | 0.0791 | 0.0334 | 0.0791 | 0.0334 | 0.0323 | 0.0143 |
| PyrM_uridine-kinase_ITP | 0.0791 | 0.0334 | 0.0791 | 0.0334 | 0.0658 | 0.0292 |
| PyrM_uridine-kinase_UTP | 0.1384 | 0.0585 | 0.1384 | 0.0585 | 0.0738 | 0.0327 |
| PyrM_uridine-phosphorylase | 0.0720 | 0.0304 | 0.0720 | 0.0304 | 0.0665 | 0.0295 |
| PyrM_uridine-ribohydrolase | 0.0000 | 0.0000 | 0.0000 | 0.0000 | 0.0000 | 0.0000 |
| PyrM_UTP_L-glutamine-amido-ligase | 0.0403 | 0.0171 | 0.0403 | 0.0171 | 0.1040 | 0.0462 |
| SERP0290-zinc-transport_efflux | 0.0791 | 0.0334 | 0.0791 | 0.0334 | 0.0658 | 0.0292 |
| SERP0291-zinc-transporter_import | 0.0791 | 0.0334 | 0.0791 | 0.0334 | 0.0658 | 0.0292 |
| SERP0292-iron-dicitrate-transporter_import | 0.0791 | 0.0334 | 0.0791 | 0.0334 | 0.2022 | 0.0898 |
| SERP0389-Glyc_Ethanol_NAD+-oxidoreductase | -0.0221 | -0.0094 | -0.0221 | -0.0094 | -0.2799 | -0.1243 |
| SERP0653-PurM_FGAM-synthethase | 0.0000 | 0.0000 | 0.0000 | 0.0000 | 0.0000 | 0.0000 |
| SERP0655-PurM_amidophosphoribosyltransferase | 0.0000 | 0.0000 | 0.0000 | 0.0000 | 0.0000 | 0.0000 |
| SERP0656-PurM_AIR_synthetase | 0.0000 | 0.0000 | 0.0000 | 0.0000 | 0.0000 | 0.0000 |
| SERP0657-PurM_GAR-formyltransferase | 0.0000 | 0.0000 | 0.0000 | 0.0000 | 0.0000 | 0.0000 |
| SERP0658-PurM_AICAR-formyltransferase | 0.0000 | 0.0000 | 0.0000 | 0.0000 | 0.0000 | 0.0000 |
| SERP0659-PurM_phosphoribosylamine-glycine-ligase | 0.0000 | 0.0000 | 0.0000 | 0.0000 | 0.0000 | 0.0000 |
| SERP0686-spermidine/putrescine-transport_import | 0.1581 | 0.0669 | 0.1581 | 0.0669 | 0.2594 | 0.1152 |
| SERP0687-spermidine/putrescine-transport_import | 0.0791 | 0.0334 | 0.0791 | 0.0334 | 0.0658 | 0.0292 |
| SERP0688-spermidine/putrescine-transport_import | 0.0277 | 0.0117 | 0.0277 | 0.0117 | 0.0454 | 0.0202 |
| SERP0765-Uracil-permease-transport_import | 0.1605 | 0.0679 | 0.1605 | 0.0679 | 0.1771 | 0.0786 |
| SERP0831-PurM_DNA-directed-DNA-polymerase_dATP | 0.0791 | 0.0334 | 0.0791 | 0.0334 | 0.1725 | 0.0766 |
| SERP0831-PurM_DNA-directed-DNA-polymerase_dCTP | 0.0530 | 0.0224 | 0.0530 | 0.0224 | 0.0441 | 0.0196 |
| SERP0831-PurM_DNA-directed-DNA-polymerase_dGTP | 0.0791 | 0.0334 | 0.0791 | 0.0334 | 0.0658 | 0.0292 |
| SERP0831-PurM_DNA-directed-DNA-polymerase_dTTP | 0.1107 | 0.0468 | 0.1107 | 0.0468 | 0.0922 | 0.0409 |
| SERP0841-PurM_PNPase_ADP | 0.1581 | 0.0669 | 0.1581 | 0.0669 | 0.1317 | 0.0585 |
| SERP0841-PurM_PNPase_GDP | 0.0285 | 0.0120 | 0.0285 | 0.0120 | 0.0112 | 0.0050 |
| SERP1403-MultiDrug-transport_efflux | 0.0791 | 0.0334 | 0.0791 | 0.0334 | 0.0658 | 0.0292 |
| SERP1802-cobalt/nickel-transport_efflux | 0.0727 | 0.0308 | 0.0727 | 0.0308 | 0.0632 | 0.0281 |
| SERP1803-cobalt/nickel-transport_efflux | 0.0791 | 0.0334 | 0.0791 | 0.0334 | 0.0658 | 0.0292 |
| SERP1944-MultiDrug-transport_efflux | 0.1170 | 0.0495 | 0.1170 | 0.0495 | 0.2430 | 0.1079 |
| SERP1951-lipoprotein-transport_efflux/import | 0.0791 | 0.0334 | 0.0791 | 0.0334 | 0.0658 | 0.0292 |
| SERP1952-macrolide-transport_efflux | 0.0791 | 0.0334 | 0.0791 | 0.0334 | 0.1363 | 0.0605 |
| SERP1997-formate/nitrite-transport_efflux/import | 0.1297 | 0.0548 | 0.1297 | 0.0548 | 0.0711 | 0.0316 |
| SERP2060-glyerol-transport_import | 0.0791 | 0.0334 | 0.0791 | 0.0334 | 0.0658 | 0.0292 |
| SERP2156-Glyc_L-lactate-dehydrogenase | -0.1581 | -0.0669 | -0.1581 | -0.0669 | 0.1317 | 0.0585 |
| SERP2179-choline/betaine/carnitine-transp_efflux | 0.1961 | 0.0829 | 0.1961 | 0.0829 | 0.1449 | 0.0643 |
| SERP2186-PurM_ATP_sulfate-adenylyltransferase | 0.0791 | 0.0334 | 0.0791 | 0.0334 | 0.0658 | 0.0292 |
| SERP2283-phopsphonate-transport_import | 0.0791 | 0.0334 | 0.0791 | 0.0334 | 0.0658 | 0.0292 |
| SERP2289-MultiDrug-transport_efflux | 0.0633 | 0.0268 | 0.0633 | 0.0268 | 0.0527 | 0.0234 |
| TCA_citrate-hydro-lyase | 0.2222 | 0.0940 | 0.2222 | 0.0940 | -0.0988 | -0.0439 |
| TCA_citrate-hydroxymutase | -0.0791 | -0.0334 | -0.0791 | -0.0334 | -0.0658 | -0.0292 |
| TCA_citrate_synthase | -0.1431 | -0.0605 | -0.1431 | -0.0605 | 0.1646 | 0.0731 |
| TCA_fumarate-hydratase | 0.0791 | 0.0334 | 0.0791 | 0.0334 | 0.0658 | 0.0292 |
| TCA_isocitrate-hydro-lyase | 0.0791 | 0.0334 | 0.0791 | 0.0334 | 0.0658 | 0.0292 |
| TCA_lipoic-transsuccinylase | -0.1676 | -0.0709 | -0.1676 | -0.0709 | -0.2278 | -0.1012 |
| TCA_Oxidoreductase | 0.0886 | 0.0375 | 0.0886 | 0.0375 | 0.1620 | 0.0719 |
| TCA_oxoglutarate-dehydrogenase-complex1 | 0.1676 | 0.0709 | 0.1676 | 0.0709 | 0.2278 | 0.1012 |
| TCA_oxoglutarate-dehydrogenase-complex2 | 0.1676 | 0.0709 | 0.1676 | 0.0709 | 0.2278 | 0.1012 |
| TCA_oxoglutarate-synthase | 0.0791 | 0.0334 | 0.0791 | 0.0334 | 0.0658 | 0.0292 |
| TCA_PEP-carboxylase | -0.5211 | -0.2204 | -0.5211 | -0.2204 | -0.5261 | -0.2336 |
| TCA_Pyruvate_CO2-ligase | -0.0791 | -0.0334 | -0.0791 | -0.0334 | -0.0658 | -0.0292 |
| TCA_pyruvate_dehydrogenase | 0.0380 | 0.0161 | 0.0380 | 0.0161 | 0.1054 | 0.0468 |
| TCA_succinate-dehydrogenase | 0.0419 | 0.0177 | 0.0419 | 0.0177 | 0.0349 | 0.0155 |

**IX: PCR results**

Figure S6: Results for PCR detection of *in silico* predicted additional enzymes for *S. epidermidis*^1^*.*

*
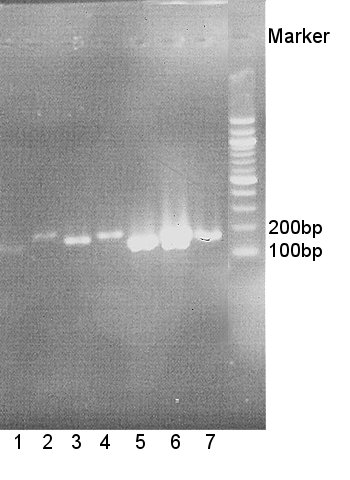
*

^1^ Lanes 1-7: *S. epidermidis* (1 = nucleoside-triphosphate diphosphatase, 2 = adenosinetriphosphatase, 3 = deoxycytidine kinase, 4 = 5'-nucleotidase, 5 = xanthine phosphoribosyltransferase, 6 = thymidine phosphorylase, 7 = epoxyadenosine kinase). Lanes 8-13: *C. albicans.* For more details of enzymes listed in the legend, see also Table 1S.

**X: Mode of action: Complex 1&3 of the oxidative phosphorylation**

Figure S7: Mode of action of complex 1 of the oxidative phosphorylation.

**
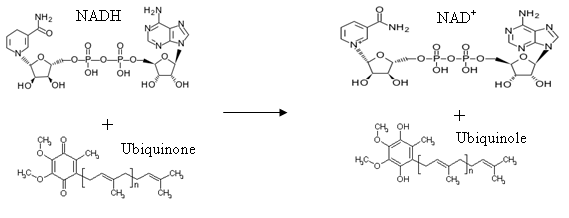
**

Figure S8: Mode of action of complex 3 of the oxidative phosphorylation.


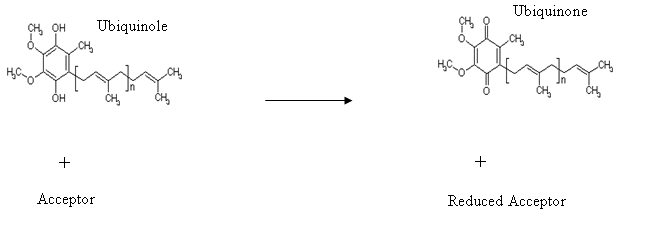

Supplement: Additional file 1 — Supplementary materials. Additional file 1 is a Word document containing additional data on sequence comparisons, pathway models, synthesis and effects of the IQ-143 compound, gene expression data, and nucleotide and NAD measurements, as reported in the manuscript. [file gb-2011-12-3-r24-S1.DOC]
